# Comparative genomic analysis of a Shiga toxin-producing *Escherichia coli* (STEC) O145:H25 associated with a severe pediatric case of hemolytic uremic syndrome in Davidson County, Tennessee, US

**DOI:** 10.1186/s12864-020-06967-3

**Published:** 2020-08-17

**Authors:** Julio A. Guerra, Chengxian Zhang, Jonathan E. Bard, Donald Yergeau, Natasha Halasa, Oscar G. Gómez-Duarte

**Affiliations:** 1 International Enteric Vaccine Research Program, Division of Infectious Diseases, Department of Pediatrics, University at Buffalo, The State University of New York (SUNY), Jacobs School of Medicine and Biomedical Sciences, 875 Ellicott St. Office 6090, Buffalo, NY 14203 USA; 2 Division of Pediatric Infectious Diseases, Department of Pediatrics, Vanderbilt University School of Medicine, Nashville, TN USA; 3 UB Genomics and Bioinformatics Core, Center of Excellence in Bioinformatics, University at Buffalo, The State University of New York, Buffalo, NY USA

**Keywords:** STEC, Diarrhea, Children, Gastroenteritis, Hemolytic-uremic syndrome

## Abstract

**Background:**

Shiga toxin-producing *E. coli* (STECs) are foodborne pathogens associated with bloody diarrhea and hemolytic uremic syndrome (HUS). Although the STEC O157 serogroup accounts for the highest number of infections, HUS-related complications and deaths, the STEC non-O157, as a group, accounts for a larger proportion of STEC infections and lower HUS cases. There is limited information available on how to recognize non-O157 serotypes associated with severe disease. The objectives of this study were to describe a patient with STEC non-O157 infection complicated with HUS and to conduct a comparative whole genome sequence (WGS) analysis among the patient’s STEC clinical isolate and STEC O157 and non-O157 strains.

**Results:**

The STEC O145:H25 strain EN1I-0044-2 was isolated from a pediatric patient with diarrhea, HUS and severe neurologic and cardiorespiratory complications, who was enrolled in a previously reported case-control study of acute gastroenteritis conducted in Davidson County, Tennessee in 2013. The strain EN1I-0044-2 genome sequence contained a chromosome and three plasmids. Two of the plasmids were similar to those present in O145:H25 strains whereas the third unique plasmid EN1I-0044-2_03 shared no similarity with other STEC plasmids, and it carried 23 genes of unknown function. Strain EN1I-0044-2, compared with O145:H25 and O157 serogroup strains shared chromosome- and plasmid-encoded virulence factors, including Shiga toxin, LEE type III secretion system, LEE effectors, SFP fimbriae, and additional toxins and colonization factors.

**Conclusions:**

A STEC O145:H25 strain EN1I-0044-2 was isolated from a pediatric patient with severe disease, including HUS, in Davidson County, TN. Phylogenetic and comparison WGS analysis provided evidence that strain EN1I-0044-2 closely resembles O145:H25, and confirmed an independent evolutionary path of STEC O145:H25 and O145:H28 serotypes. The strain EN1I-0044-2 virulence make up was similar to other O145:H25 and O157 serogroups. It carried *stx2* and the LEE pathogenicity island, and additional colonization factors and enterotoxin genes. A unique feature of strain EN1I-0044-2 was the presence of plasmid pEN1I-0044-2_03 carrying genes with functions to be determined. Further studies will be necessary to elucidate the role that newly acquired genes by O145:H25 strains play in pathogenesis, and to determine if they may serve as genetic markers of severe disease.

## Background

Shiga toxin-producing *Escherichia coli* (STEC), also known as Vero toxin-producing *E. coli* [1, 2], are defined as strains that express one or two bacteriophage-encoded Shiga toxins Stx1 and Stx2 [3]. STECs, a cause diarrhea and hemolytic uremic syndrome (HUS) in children and adults worldwide, may present in the form of sporadic cases or outbreaks [4, 5]. O157 is the most common serogroup associated with diarrhea and HUS in the US. Nevertheless, non-O157 STEC serogroups are surpassing the number O157 STECs infections and have the potential for large outbreaks [4–16]. Up to 52% of all STEC associated disease is due to non-O157 STEC, which corresponds to more than 37,000 illnesses annually in the US [17]. Although O157 serogroup leads to more severe disease, the increasing number of non-O157 infections is of public health concern, first, because it is difficult to discern among those associated with more severe disease and also because virulence markers for detection are currently unknown [7, 13, 18].

There are over 400 non-O157 STEC serotypes, of which more than 100 are reported to cause gastrointestinal disease in humans [8, 9]. Strains from serogroups O26, O45, O103, O111, O121, and O145, also known as the “big six”, are most frequently associated with human illness [13, 19–21]. A relevant non-O157 STEC is the O104:H4 serotype which emerged from an enteroaggregative *E. coli* (EAEC) by acquiring a stx2 phage that caused a large outbreak bloody diarrhea in Europe in 2011 with a high rate of HUS and mortality [22].

STEC serogroup O145 has been associated with outbreaks of diarrhea and HUS worldwide [21, 23–28]. Among the main serotypes within this serogroup, O145:H28 is the most frequently detected, with outbreaks reported in the US [26, 29] and Belgium [25, 30]. Serotype O145:H25 is less frequently detected yet, a larger proportion of reported cases are associated with HUS, highlighting the clinical significance of this serotype [24, 27, 31, 32]. Despite the importance of this serotype, there is limited information on the evolutionary path and the genomic composition, including the traits associated with virulence and colonization of this serotype. Furthermore, data are missing regarding whether O145:H25 has a distinct evolutionary lineage compared with O145:H28 [32]. Robust genomics integrated with epidemiology information may answer key questions about the virulence profile, disease severity, epidemic risk assessment, and genetic origin of these poorly characterized strains.

The objectives of this study were to describe a case of acute gastroenteritis associated with HUS in a Tennessean child and to conduct a comparative analysis of the whole genome sequence of this STEC O145:H25 clinical isolate with previously reported STEC O145:H25 and other STEC genomes. The child was enrolled as a participant in a National Vaccine Surveillance Network (NVSN) study in Davidson County, TN [33]. This STEC O145:H25 strain was identified in 2013, 1 year after a US multistate outbreak of STEC O145:H25 in 2012, that resulted in 18 infections, 4 hospitalizations and one death [34]. This study compared the genomics of the STEC O145:H25 strain EN1I-0044-2 with previously reported O145:H25 [32], O145:H28 [35] and other important STEC serotypes.

## Results and discussion

### Clinical presentation and course

We isolated a STEC O145:H25 strain from a child with acute gastroenteritis complicated with HUS. A 30-month-old previously healthy Hispanic female, fully immunized, developed a non-bloody, mucus-containing diarrhea 5 days prior to hospital admission. Two days prior to admission, she complained of abdominal pain, nausea and multiple episodes of vomiting. Despite oral hydration with electrolyte solution administered at home, her condition continued to worsen due to persistent diarrhea and vomiting. Patient was brought to an Emergency Department (ED) by her parents, where she was initially diagnosed with a viral illness, given sublingual ondansetron and discharged to home. The following day, the diarrhea and vomiting persisted, and she returned to the ED where intravenous fluids (IVF) were initiated. Complete blood cell count (CBC) revealed a white blood cell count of 29,000/μL, platelet count 220,000/μL, and creatinine at 1.4 mg/dl. On the next day, the CBC was abnormal with a platelet count of only 102,000/μL and creatinine increased to 2.4 mg/dl. The patient was diagnosed with bloody diarrhea and hemolytic uremic syndrome (HUS), presumptively secondary to STEC diarrhea, and transferred to a tertiary care children’s hospital in Nashville, TN.

Upon arrival to the hospital, the patient had nausea, vomiting, diarrhea and altered mental status. She was directly admitted to the pediatric intensive care unit (PICU) for diagnosis and management. She was intubated due to impending respiratory failure and was mechanical ventilated. She received IVF and required several packed red blood cell transfusions due to HUS-associated anemia. She tested negative for rotavirus and positive for STEC by stool culture and molecular testing. Neurological evaluation, confirmed by magnetic resonance imaging (MRI), revealed extensive basal ganglia and thalamic restricted diffusion consistent with ischemic injury likely associated with HUS. The EEG showed frequent subclinical unilateral occipital lobe seizures and she was started on anti-convulsant medications. Cardiology evaluation reported increasing tachycardia, narrow pulse pressure, and the echocardiogram revealed pericardial effusion with tamponade as well as bilateral pleural effusion. An emergent pericardial drain was placed as well as a pleural catheter to drain pericardial fluid and pleural fluid, respectively. She also received milrinone and nicardipine to improve left ventricular function. HUS-associated renal failure in the presence of cardiac insufficiency and pericardial and pleural extravascular fluid prompted initiation of hemodialysis management. Patient’s clinical condition improved over the following 4 weeks after admission and she was discharged to home with instructions for outpatient follow up.

### General genomic features of STEC O145:H25 strain EN1I-0044-2

Despite the epidemiological importance of O145:H25 serotype STECs, there is limited information on the evolutionary path and the genomic composition, including the traits associated with virulence and colonization. Only two complete genomes of two O145:H25 strains associated with HUS have been previously described, and only one study has performed comparative genomic analysis [32].

Initially, the *E. coli* strain EN1I-0044-2 obtained from patient’s stool sample tested positive for both *eae* and *stx*2 genes by polymerase chain reaction (PCR) and serotyping results reported O145:H25 [33]. The STEC O145:H25 strain EN1I-0044-2 was further evaluated by whole genome sequence analysis that revealed a 5276,111 bp genome consisting of 5417 coding DNA sequences (CDS), 22 rRNAs, 89 tRNAs and 54 sRNAs. The backbone chromosome consisted of 5149 CDS, interrupted by mobile genetics elements (MGEs), including prophage/prophage-like elements (*n* = 13), integrate elements (*n* = 8), and insertion sequences (*n* = 62). The *stx2* gene encoding Shiga toxin-2 was located in a prophage of about 31.8 Kb in size. The LEE pathogenicity island locus was integrated at the tRNA *pheU* locus. The STEC O145:H25 strain EN1I-0044-2 carries three plasmids, the pEHEC-like plasmid pEN1I-0044-2_01 (49,096 bp) and two additional plasmids pEN1I-0044-2_02 (31,963 bp) and pEN1I-0044-2_03 (63,546 bp) (Table 1).

**Table 1:** Genome characteristics of STEC EN1I-0044-2 compared with other highly pathogenic STECs

Chromosome	O145:H25 str. EN1I-0044-2	O145:H25 str. CFSAN004177	O145:H28 str. RM13514	O26:H11 str. 11,368	O103:H2 str. 12,009	O111:H- str. 11,128	O157:H7 str. Sakai
Size (kb)	5131	5191	5586	5697	5449	5371	5498
GC (%)	50.5	50.5	50.7	50.7	50.7	50.6	50.5
CDSs	5149	5193	5613	5364	5054	4972	5230
tRNA	99	96	104	101	98	107	105
Prophages	13	14	20	21	15	17	18
*stx* genes	*stx2a*	*stx2a*	*stx2a*	*stx1*	*stx1 + 2a*	*stx1 + 2a*	*stx1 + 2a*
Number of IS^a^	55	73	53	62	50	56	37
Integrate elements (IE)	8	9	7	9	6	7	6
LEE-island integration locus	*pheU*	*pheU*	*selC*	*pheU*	*pheV*	*pheV*	*selC*
**pEHEC-like plasmid**							
Size (kb)	50	52	87	85	76	78	93
GC (%)	47	47	47.6	47.5	49.1	50	47.6
CDSs	79	58	94	98	67	72	85
Number of IS^a^	4	4	4	6	6	8	3
**Other plasmid(s)**							
Size (kb)	32/63	35/96	65	63/6/4	N/A	205/98/8/7	3
GC (%)	49/51	49/48	53	52/46/44	N/A	47/48/50/50	43
CDSs	48/86	46/120	69	93/10/3	N/A	222/121/10/10	3
Number of IS^a^	4/0	5/0	6	1/0/0	N/A	14/1/0/0	0
**Total genome size (bp)**	**5276**	**5374**	**5738**	**5855**	**5525**	**5767**	**5594**

^a^Insertion sequences (coverage ≥90%, identity ≥90%)

**Fig. 1:**
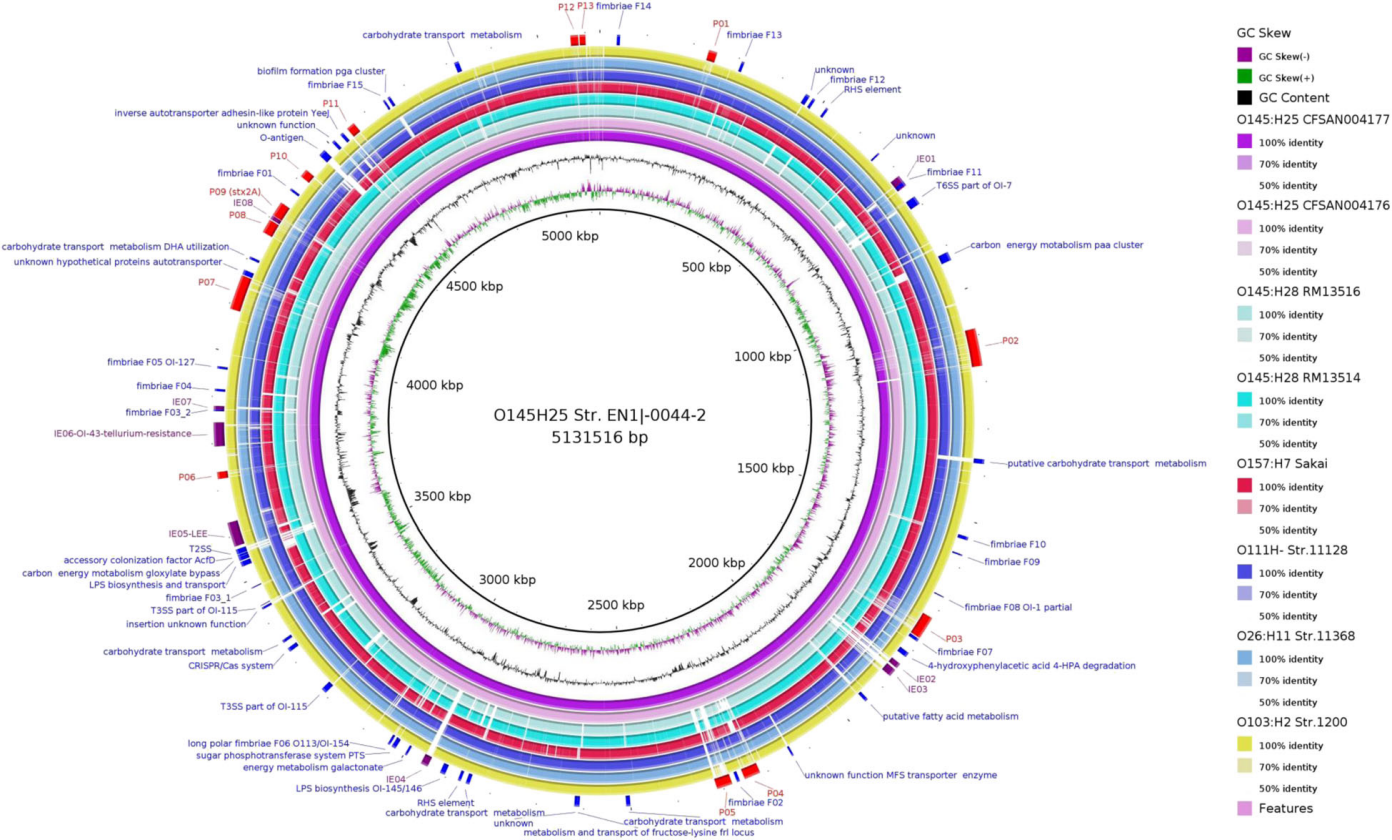
Circular map and genome features of STEC O145:H25 strain EN1I-0044-2 in comparison to other STEC strains. The circular map was generated using BLAST Ring Image Generator (BRIG) software [36]

In silico serotyping analysis confirmed that STEC strain EN1I-0044-2 belong to serotype O145:H25. Multilocus sequencing typing (MLST) showed that STEC strain EN1I-0044-2 belong to sequence type (ST) 5309. In contrast, STEC O145:H25 strain CFSAN004177 belong to ST 7061 (Table 2). Only *mdh* allele was dissimilar among O145:H25 strains, differing in a single nucleotide polymorphism (SNP) (A431G). The WGS of reference STEC strains, showed significant conservation of the chromosomal backbone among STEC strains (Fig. 1). However, differences in the number and size of acquired MGEs such as prophages, IEs, and plasmids were identified among O145:H25 strains in comparison with other STEC strains (Table 1). Previously reported non-homologous regions, detected in O145:H25 strain CFSAN004177 and evaluated in strain EN1I-0044-2 [32] using Blast analysis, showed that these regions are present or partially present in strain EN1I-0044-2 (Additional file 1: Table S1).

**Table 2:** MLST analysis of the STEC strains

Serotype	strain	Seven ***E. coli*** MLST gene loci allele profile							ST	ST Cplx
		***adk***	***fumC***	***gyrB***	***icd***	***mdh***	***purA***	***recA***		
O145:H25	EN1I-0044-2^a^	437	46	69	1	20^b^	34	50	5309	none
O145:H25	CFSAN004176	437	46	69	1	600	34	50	7061	none
O145:H25	CFSAN004177	437	46	69	1	600	34	50	7061	none
O145:H28	RM13514	19	23	18	24	21	2	16	32	32
O26:H11	11,368	16	4	12	16	9	7	7	21	29
O111:HNM	11,128	6	4	12	16	9	7	12	16	29
O103:H2	12,009	6	4	3	17	7	7	6	17	20
O157:H7	Sakai	12	12	8	12	15	2	2	11	11

^a^Study strain: STEC O145:H25 strain EN1I-0044-2. ^b^The *mdh* allele was dissimilar among O145:H25 strains in a single nucleotide polymorphism (SNP). The SNP in strain EN1I-0044-2 is A431G

### Phylogenetic analyses demonstrated clustering of O145:H25 strains, including EN1I-0044-2

**Fig. 2:**
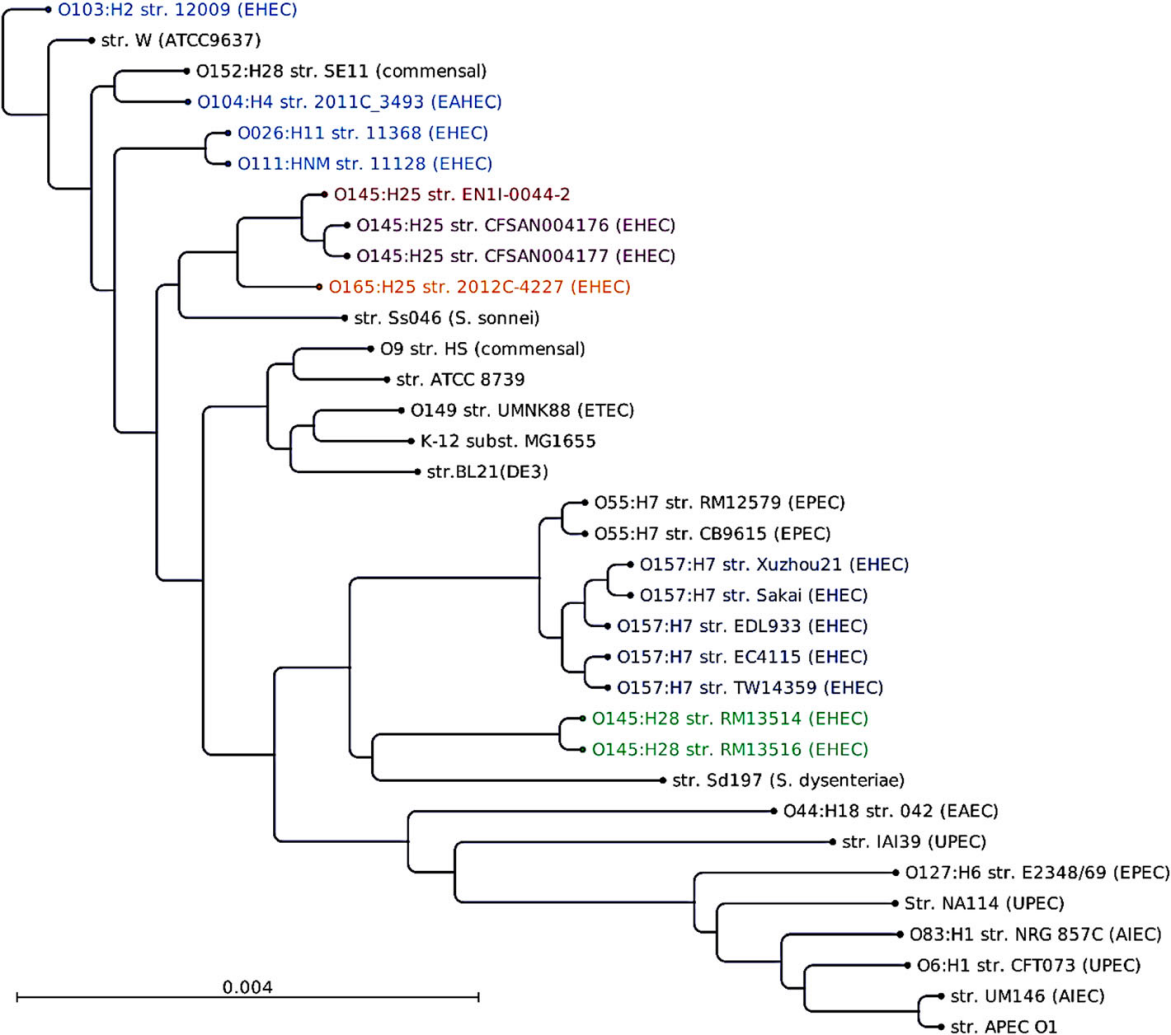
Genome-wide phylogenetic analysis of *E. coli* and *Shigella* strains. The maximum likelihood (ML)-based phylogenetic tree based on the concatenated nucleotide sequences of 341 orthologous CDSs was constructed as described previously [35, 37]. Pairwise comparisons of all genome sequences were carried out using NUCmer from the MUMer package [38] and highly similar regions (repeated sequences) were removed from the analysis. Genomes were downloaded from GenBank, including 13 STEC strains, the German outbreak EHAEC strain, 17 other *E. coli* and 2 *Shigella* strains

In this study, phylogenetic analysis indicated that STEC O145:H25 strain EN1I-0044-2 has a common evolutionary lineage with two recently described O145:H25 strains, and a different evolutionary lineage from STEC O145:H28 strains which has been previously reported [32]. The maximum-likelihood tree constructed showed that strain EN1I-0044-2 was clustered with other O145:H25 strains. STEC O165:H25 strain 2012C-4227 was clustered with other O145:H25 strains. *S. sonnei* strain Ss046 was also clustered with O145:H25 and O165:H25 strains. In contrast, O145:H28 strains were distant from O145:H25 strains, and were clustered with *S. dysenteriae* strain Sd197, some STEC O157:H7 and EPEC O55:H7 strains. O145:H25 strains were also distant from important non-O157 STEC strains, including O103:H2 strain 12,009, O26:H11 strain 11,368, O111:HNM strain 11,128 and the German outbreak O104:H4 strain 2011C_3493 (Fig. 2).

### Mobile genetic elements identified in STEC O145:H25 strain EN1I-0044-2

Comparative genomic analysis of STEC O145:H25 strain EN1I-0044-2 against the chromosome sequence of reference STEC strains revealed that there was significant conservation of the chromosomal backbone among STEC strains, as previously described [32, 35, 37, 39]. Similarities in the number and size of acquired MGEs such as plasmids, prophages, IEs, and IS were identified between strain EN1I-0044-2 and the other two STEC O145:H25 strains. However, dissimilarities of acquired MGEs were found between O145:H25 strains and other important STEC serotypes, which may explain the differences in the genome size among STEC strains [32]. Acquired MGEs are known to play an important role in driving genome and virulence evolution of STEC strains [35].

#### Plasmids

Previous studies have demonstrated that STEC strains differs considerably in the number and composition of plasmids [32, 35, 37] yet, O145:H25 strain EN1I-0044-2 carry two plasmids that are similar to other O145:H25 strains [32]. Subsequent DNA sequence analysis showed that the main pEN1I-0044-2_01 plasmid (49,096 bp) present in strain EN1I-0044-2 is similar to pEHEC-like virulence plasmids carried by others O145:H25 strains, including the virulence gene composition (99% sequence identity with 98% sequence coverage). The second plasmid pEN1I-0044-2_02 is similar to pCFSAN004176P_01 [CP012492.1] and pCFSAN004177P_01 [CP012496.1] present in O145:H25 strains CFSAN004176 and CFSAN004177 (99% sequence identity with 99 and 100% sequence coverage, respectively). This plasmid carries the *sfp* fimbrial gene cluster [32] which is also present among STEC O157 and O165 strain plasmids [40, 41].

The third plasmid pEN1I-0044-2_03 (63,546 bp) seems to be unique to STEC strain EN1I-0044-2 as it did not share homology with any of the additional plasmids present among STEC strains, including the two O145:H25 strains. In fact, pEN1I-0044-2_03 is similar to plasmids pM110_FII DNA [AP018140.1] from *E. coli* clinical isolate M110 isolated from blood specimen in a tertiary care hospital in Yangon, Myanmar [42] and pEC974–3 [CP021843.1] from *E. coli* clinical isolate EC974 from a women with urinary tract infection (UTI) [43]. However, pEN1I-0044-2_03 does not harbor resistance genes against quinolones and tetracycline present in pM110_FII DNA [42] or the DNA segment containing the class A broad-spectrum beta-lactamase TEM-1 gene and IS elements/transposase genes such as IS1, IS6 and Tn3 family transposase harboring by pEC974–3 [43]. pEN1I-0044-2_03, an IncF conjugative plasmid carries genes encoding conjugative transfer proteins and, in addition, it carries 23 genes of unknown function that deserved to be analyzed at the molecular level to discern their possible role in pathogenesis (Additional file 1, Table S4). Further studies will be necessary to determine if virulence phenotypes are associated to these plasmid-encoded genes. The sequence of this plasmid was annotated using RAST webserver (https://rast.nmpdr.org) [44–46].

#### Prophages and integrate elements (IE)

STEC strain EN1I-0044-2 has similar number and type of prophage/prophage-like elements to other O145:H25 strains [32]. STEC O145:H25 strain EN1I-0044-2 has 13 prophage/prophage-like elements and lambda-like phages were predominant among them (Table 3). The lambda-like family Stx2a phage in strain EN1I-0044-2 (31.8 kb) was smaller than Stx2a phages carried by O145:H25 strains CFSAN004176 (44.7 kb) and CFSAN004177 (44.7 kb) (Additional file 6: Figure S1, panel A). The missing DNA region of phage Stx2a in strain EN1I-0044-2 carries 11 ORFs genes most of which encode phage-related proteins (Additional file 6: Figure S1, panel A). Comparison and phylogenetic analysis showed that the Stx2a phage of strain EN1I-0044-2 was highly related to O145:H25 phages and unrelated to O145:H28, O157, O111, and O103 phages (Additional file 6: Figure S1, panel B).

**Table 3:** Identified prophages and integrative elements (IE) in STEC O145:H25 str. EN1I-0044-2

Name	Contig	Contig position (start)	Contig position (end)	Size (Kb)	GC%	Insertion site	Most common Phage Type	T3SS effector proteins
**Prophages**								
P01	1	234,098	252,152	22.3	48	ybhB	Stx2 converting phage vB_EcoP_24B (Podoviridae-like)	*traR, nleG, espJ*
P02	2	277,808	366,514	88.7	50.1	ybhB	Enterobacteria phage YYZ-2008 (unclassified)	tRNA’s (Arg, Met), *nleG*
P03	3	254,241	303,995	49.8	49	*yjbN*	Enterobacteria phage mEp460 (lambda-like)	*fosA*
P04	4	202,949	238,006	35.1	52.4	*yhdJ*	**1** Enterobacteria phage P88 (Myoviridae)	
P05	4	266,538	299,976	33.4	45	*tRNA (argW)*	**2** Salmonella Phage 103203_sal5 (Podoviridae)	
P06	7	249,027	263,648	14.6	48.1	*yjbN*	**3** Enterobacteria phage mEp460 (lambda-like)	
P07	9	307	75,279	75	51.2	*tRNA (serU)*	Enterobacteria phage lambda NC_001416 (lambda-like)	*traR, hydrolas*
P08	9	177,135	206,738	29.6	53.5	*ompW*	Enterobacteria phage BP-4795 (lambda-like)	*terB,* tRNA’s *(Met, Arg),* colonization factor AcfC
P09	9	218,608	250,415	31.8	51	*yciE*	Enterobacteria phage phiP27 (lambda-like)	*lom*, peptidase S14, ***stx2a****,* serine protease
P10	10	69,262	87,022	17.7	43.6	*yegL*	Enterobacteria phage BP-4795 (lambda-like)	nleH, espJ, cif*
P11	11	1	18,207	18.2	48.7		Enterobacteria phage mEp237 (lambda-like)	*terB*
P12	19	402	16,927	16.5	48.9	*ompW*	Phage Gifsy-2 (unclassified)	*tRNA’s (Arg, Met)*
P13	20	2665	15,249	12.5	56.7		Enterobacteria phage BP-4795 (lambda-like)	
**Integrative elements**								
IE01	1	721,994	747,268	25.2	48	tRNA (*thrW*)		Intimin-like adhesin *fdeC*, fimbrial operon
IE02	3	372,461	383,975	11.5	47	*yjjG*		restriction endonuclease
IE03	3	388,759	405,824	17	48	tRNA (*leuX*)		RM system, peptidase S8
IE04	5	390,657	406,347	15.7	44	tRNA (*selC*)		Putative two-component abortive infection system (Abi), RM system
IE05	7	102,015	153,886	51.9	38	tRNA (*pheU*)	LEE insertion site	*espA*, *espB*, *espD*, *espF*, *espG, espH*, *espL*, *espS*, *espZ*, *map*, *tir*, *eae*, adhesin *efa*1, *nleE*, *nleB*
IE06	8	1	51,161	51.1	49	tRNA (*metX*)	OI-43 insertion site	Urease operon, tellurium resistance, *espI*
IE07	8	78,288	86,298	8	42	*viuB*-*fimD*		
IE08	9	209,198	217,645	8.4	41			*nleG*, *nleG*, *espO*, *nleF*, *nleH*, *espM*

STEC strain EN1I-0044-2 has 8 IE, two of them EI3 and EI4 carrying restriction-modification (RM) systems (phage defense mechanism) (Table 3), which have been described among EI of other STEC O145:H25 strains [32]. RM system was identified by the detection of methyltransferase (MTase) and restriction endonuclease (REase) genes by BLASTN comparison with other STEC O145:H25 strains. In addition, IE4 also carries a phage defense mechanism called Abortive infection (Abi) system. Abi is activated once a phage, evading restriction by host RM systems and by CRISPR, enters the host cell, leading to self-death (“suicide”) to prevent spreading of phages to other cells [47].

#### Non-homologous regions

In contrast to prophages and IEs, which encode integrases that catalyze their excision and integration [48], non-homologous regions do not contain integrases or transposon genes. BLAST analysis of these regions previously detected in STEC O145:H25 strains [32] showed that all of them are present and/or partially present in strain EN1I-0044-2 (Additional file 1: Table S1). Dissimilarities among these regions has been previously detected in O145:H25 and other STEC strains [32]. These regions contain gene clusters associated with type II secretion system (T2SS), type VI secretion system (T6SS), CRISPR (clustered regularly interspersed palindromic repeats) loci, metabolism and fimbrial biosynthesis.

#### Insertion sequences (IS)

IS elements identified in strain EN1I-0044-2 were similar to those present in STEC O145:H25 strains and different from other STEC. The small differences in the distribution of IS elements among O145:H25 strains may be explained by differences at insertion sites since most of them are located in MGEs such as prophages, prophages like elements, IEs and plasmids. In fact, significant differences in the types and copy number of IS elements has been described among STEC strains [32, 49]. It’s believed that IS elements not only play important roles in bacterial genome evolution and diversification [37, 39], but also participate in the immobilization of MGEs, resulting in their fixation in the genome [39]. A total of 15 types of IS elements with a total of 62 copies producing significant alignment were identified in the chromosome and plasmids sequences of STEC strain EN1I-0044-2. IS600 was the most prevalent IS in the chromosome of O145:H25 strains, in contrast to other STEC strains such as O145:H28 strain RM13514 and O26:H11 in which a few copies were detected, or not detected, including O103:H2 strain 12,009, O111: HNM strain 11,128 and O157:H7 strain Sakai. IS629 was absent in O145:H25 strains but it was commonly found in the chromosome of STEC O157:H7 strain Sakai and non-O157 strains (Additional file 2: Table S1). In pEN1I-0044-2_01 and other pEHEC-like plasmids a few copies of IS were detected with no prevalence of a particular one. Secondary plasmid pEN1I-0044-2_02 carry four IS while pEN1I-0044-2_03 does not contain any IS (Additional file 2: Table S2).

### O-islands analysis

A total of 177 genomic islands identified in O157:H7 strain EDL933 carry genes involved in metabolic, fitness and pathogenicity [50]. In this study, 47 of 177 (43%) complete or partially present O-islands were detected in O145:H25 strains EN1I-0044-2 (Additional file 1: Table S2). The LEE (OI-148) is similar in size and integration site to previously described O145:H25 strains, and the *eae* gene encoding intimin has the same size and subtype (beta) [32]. In contrast, LEE islands in O145:H28 strains are generally smaller in size and integrated at different tRNA, and carrying a different intimin type (gamma) [32, 35]. In strain EN1I-0044-2, a partial OI-122 was found outside the LEE sequence. The OI-122 among O145:H25 strains contains the *efa1* adhesin gene and three type III secretion system (T3SS) effector protein genes, and it is considered a LEE accessory region [32]. STEC O145:H25 strains carrying a second OI-122 related to T3SS is located outside the LEE island and partially conserved among STEC O145:H28 strains [35].

OI-7 (T6SS) and OI-115 (T3SS) are partially present in all three O145:H25 strains and others STEC strains. However, O145:H28 strains only have OI-115 [32, 35]. O145:H25 strains carries only OI-43 (including the urease gene cluster). In contrast, O145:H28 strain RM13514 carries both OI-43 and OI-48 and strain RM13516 only one (OI-43) [32, 35]. OI-43 and OI-48 (known as tellurian resistance islands) contain tellurian resistance and urease gene cluster [32, 35]. Urease has been suggested to play a role in cell acid resistance and in the gastrointestinal tract of the host [35, 51].

### Chromosomal and plasmid virulence factors identified

The number of LEE and non-LEE encoded effectors present in strain EN1I-0044-2 is similar to other O145:H25 strains and it is different from other STEC serotypes [32]. LEE encodes a T3SS containing structural and effectors proteins. Among STEC serotypes O145:H25, O26:H11 and O103:H2, the EspA, B, and D proteins and the adhesin intimin receptor Tir are subtype β, while O145:H28 and O157:H7 are subtype γ. Non-LEE-encoded effectors presented a similar pattern in O145:H25, except in the number of copies of the *nleG* gene; There were only 3 copies in strain pEN1I-0044-2 instead of the 9 described in O145:H25 strain CFSAN004177 (Additional file 2: Table S3).

Plasmid-encoded virulence genes previously reported in others pEHEC-like plasmids were evaluated in pEN1I-0044-2_01 [32, 52]. pEN1I-0044-2_01 carry *ehxA*, *hlyA* and *sta1* genes and, lacks of *cba*, *cma ecf*-cluster, *espP*, *toxB*, *katP* and *stcE* and *toxB* virulence genes (Table 4). Specifically, *cba* and *cma* gene sequence were disrupted by IS in strain EN1I-0044-2. The *ecf*-cluster has been associated with bacterial persistence in the bovine gastrointestinal tract [32]. No antimicrobial resistance genes were detected in the pEN1I-0044-2_01 or the other two additional plasmids present in O145:H25 strain EN1I-0044-2 (Table 4).

**Table 4:** Virulence genes in STEC O145:H25 strain EN1I-0044-2

		O145:H25			O145:H28		O157
		EN1I-0044-2	CFSAN004176	CFSAN004177	RM13514	RM13516	Sakai
Virulence gene	Product	Copy (Type)	Copy (Type)	Copy (Type)	Copy (Type)	Copy (Type)	Copy (Type)
Chromosome-encoded							
*bor*	Bor protein	1 ^a^	1	1	1	1	2
*eae*	Intimin β type	1 (β)^b^	1 (β)	1 (β)	1 (γ)	1 (γ)	1 (γ)
*efa1*	EHEC factor for adherence	1	2	2	+/−	1	+/−
*ehaA*	Autotransporter protein	1	1	1	1	1	1
*espI*	Serine protease autotransporter	(1)^c^	1	1	1	–	–
*gad*	Serum resistance gene	2	1	1	1	–	1
*lpf cluster*	Long Polar fimbriae	1	1	1	1	1	2
*sod (Cu/Zn)*	Superoxide dismutase	1	1	1	2	2	2
*Stx2 genes*	Shiga toxin 2	1	1	1	1	1	2 (*stx1*, *stx2*)
Plasmid-encoded							
*ehxA*	Enterohemolysin	1 (E)	1 (E)	1 (E)	1 (C)	1 (C)	1 (B)
*hlyA*	Hemolysin A	1	–	–	1	1	1
*sfp cluster*	SFP fimbria	1	1	1	–	–	-^d^
*sta1*	Stable enterotoxin STIa	1	1	1	–	–	–
*espI*	Serine protease autotransporter	1	1	1	–	–	–

^a^The number represents gene copy number. ^b^Character or letter in parenthesis corresponds to gene type. ^c^ Number in parenthesis represents pseudogenes. ^d^ *sfp* cluster is absent in the O157 Sakai strain, yet it was initially described among STEC O157 strains

Chromosome-encoded virulence gene analysis showed that strain EN1I-0044-2 carries a β type intimin. Besides LEE and non-LEE encoded effectors and intimin, we detected *Efa1* encoding gene, previously reported to mediate intestinal colonization in calves [53]. Long Polar fimbriae cluster [54] was also detected in our strain EN1I-0044-2 and the other two O145:H25 strains CFSAN004176 and CFSAN004177 [32]. EhaA is a novel autotransporter protein of STEC O157:H7 that contributes to adhesion and biofilm formation that was also detected in our strain [55]. The *EspI* gene, a member of the SPATE family [56], is partially present in the chromosome of strain EN1I-0044-2 (only covers 52.7% of the gene sequence) yet, a complete copy of the gene was found in the plasmid pEN1I-0044-2_01. Strain EN1I-0044-2 lacked the *iha* and *pagC* genes sequences but carry the resistance genes: *gad*, involved in serum resistance [57]; *bor*, involved in acid resistance [58]; and *sodC*, involved in defense against extracellular phagocyte-derived reactive oxygen species (*sodC* encodes superoxide dismutase) [59]. Two copies of *gad* gene were found in EN1I-0044-2. (Table 4).

### Fimbriae gene cluster analysis

Fimbriae facilitate the initial attachment of STEC to intestinal cells and subsequence colonization of the host gut. The genome of STEC O157:H7 strains contain 16 loci-encoding genes putatively involved in pili biosynthesis [60, 61]. We identified that the number of fimbrial gene clusters in strain EN1I-0044-2 was the same in other O145:H25 strains, and similar to STEC O157 strains described previously described. 15/19 (78.9%) of the fimbrial gene clusters evaluated were present in strain EN1I-0044-2. Fourteen of them were detected in the chromosome sequence and one (Sfp fimbria) in plasmid pEN1I-0044-2_02. Fimbrial gene clusters F17 and F19 were absent in O145:H25 strain EN1I-0044-2 while F08 and F16 are partially present (Additional file 1: Table S3). This data suggests that the hypervirulence phenotype of the STEC O145:H25 strains may require fimbrial-dependent adherence, yet it may not depend on additional adherence-mediating genes such *toxB*, *iha*, or *ecf* [32].

The higher severity of STEC infections observed among patients infected with non-O157 strain including O145:H25 serotype strains remains unclear. Clinical severity of STEC O157 strains may be the result of the virulence make up. STEC non-O157 strains with similar virulence make may explain the clinical severity of some of these non-O157 serotype. Our study strain shared a number of virulence gene with O157 and non-O157 strain (Table 4). Major virulence genes in common, included *stx2*, type III secretion LEE and LEE effector genes. Other genes of interest shared by O145 and O157 serogroup strains tested were *bor*, *efa1*, *ehaA*, *gad*, *lpf cluster*, *sod*, and *ehxA*. Gen present in the study strain, O145:H25 strains and absent in O145:H28 include *sfp* fimbrial cluster, *sta1*, and *espI.* The *sfp* finbrial cluster was detected in O157, O165, and O156 strains most of them isolated from patients who suffered HUS [40, 62]. Although more epidemiological studies may be necessary to establish the association between *sfp* cluster and higher HUS risk, the presence of this cluster may have epidemiological relevance as a marker to recognize STEC strains with high virulence potential. The association *sfp* and additional O145:H45 unique genes may open the way to the development of unique genetic markers for the identification of hypervirulent O145:H25 strains associated with severe disease and life-threatening complications.

## Conclusion

We describe detailed genomic information of a STEC O145:H25 strain associated with bloody diarrhea and HUS in a child in Davidson County, TN in 2013. Whole genome sequencing analysis demonstrated that strain EN1I-0044-2 is related to previously described O145:H25 strains that were associated with HUS cases in the US in 2003 and 2004. Phylogenic analysis showed that strain EN1I-0044-2 belongs to the same lineage to O145:H25 strains CFSAN004176 and CFSAN004177 and support the hypothesis that this serotype evolved independently from serotype O145:H28. Comparative analysis of EN1I-0044-2 and the other two O145:H25 strains showed small differences in the number and topology of MGEs, including prophages, IS and plasmids. O145:H25 serotypes and O157 serogroup share important virulence genes in addition to *stx2*, among them LEE T3SS, LEE effectors, fimbrial genes, non-fimbrial colonization factors and enterotoxins. Strain EN1I-0044-2 carries also the *sfp* fimbrial cluster, shared by O157 serogroup strains, and the pEN1I-0044-2_03 plasmid that contained a number of genes whose role in pathogenesis is yet to be determined. Further studies directed to elucidate the function of genes unique among O145:H25 strains may improve our understanding of the role these genes may play in the pathogenesis of STEC disease and its severity. Unique virulence genes among O145:H25 strains may lead to the development of genetic markers for the detection of non-O157 STECs, and specifically O145:H45 strains associated with life-threatening STEC infections.

## Methods

### Bacterial strain

STEC O145:H25 strain EN1I-0044-2 was used in this study. This strain was isolated from a 30 month-old Hispanic female previously healthy with bloody diarrhea in Davison County, Tennessee, USA, while conducting active gastroenteritis surveillance under the New Vaccine Surveillance Network (NVSN) study (July 1, 2012, to June 30, 2013). The isolate EN1I-0044-2 obtained from patient’s stool sample was positive for both *eae* and *stx*2 genes by PCR and reported as serotype O145:H25 [33].

### Whole genome sequencing, assembly, and annotation

The STEC O145:H25 strain EN1I-0044-2 was cultured overnight in Luria broth at 37 °C and 200 rpm. Genomic DNA (gDNA) was isolated using GenElute Bacterial Genomic DNA Kit (Sigma-Aldrich) according to manufacturer’s instructions. The EN1I-0044-2 strain genome was sequenced by BGI Americas Corporation (Cambridge, MA) and was processed for de novo assembly, and comparative analysis. The libraries were prepared for 500 bp and 2 kb inserts paired-end sequencing on Illumina HiSeq 2000 sequencing platform. A total of 8,688,160 and 9,588,868 reads were generated from 500 bp and 2 kb libraries. Short reads were assembled into genome sequence using SOAPdenovo Version: 2.04 (http://sourceforge.net/projects/soapdenovo2/files/SOAPdenovo2/) [63]. The final assembly comprised 40 scaffolds composed of 64 contigs, resulting in a final assembly size of 5276,096 bp.

Genome annotation was conducted by the National Center for Biotechnology Information (NCBI) Prokaryotic Genome Annotation Pipeline (PGAAP) (http://www.ncbi.nlm.nih.gov/genome/annotation_prok/). This Whole Genome Shotgun project has been deposited at the NCBI GenBank under the accession numbers QQVX00000000 and PRJNA448001 (https://www.ncbi.nlm.nih.gov/bioproject/PRJNA448001).

### Comparative analysis of STEC genomes

The circular map for genome comparison of STEC strains EN1I-0044-2 was generated by BLAST Ring Image Generator (BRIG) software (http://sourceforge.net/projects/brig) using BRIG default settings [36]. The EN1I-0044-2 strain genome was set as reference and BLASTed against eight STEC genomes. The accession number of these strains are described in Additional file 3: Table S1. Non-homologous regions previously detected in O145:H25 CFSAN004177 strain (Location and size of each non-homologous region are described in Additional file 1: Table S1) [32] were first BLASTed by Basic Local Alignment against strain EN1I-0044-2. Subsequently, these regions, as well as other dissimilarities identified by BRIG image, were manually examined by CLC Genomics Workbench 11.0.1 (CLC Bio, Qiagen, Aarhus, Denmark).

### Whole-genome based phylogenetic analysis

The maximum-likelihood tree was constructed using the concatenated nucleotide sequences in FASTA format of 341 orthologous CDSs from *E. coli* subst. MG1655 [NC_000913] as reference (Additional file 4). To compare these sequences, our strain and 33 available genome sequences that were downloaded from genbank, were converted into searchable blast databases. The NC_000913 concatenated conserved fasta sequences were then compared to each database using blastn, extracting the top corresponding CDS for each WGS. These sequences were then aggregated for each strain, and compared using the MAFFT alignment tool. Subsequently, the maximum likelihood (ML)-based phylogenetic trees was built as described before [35, 37]. Pairwise comparisons of all genome sequences were carried out using NUCmer from the MUMer package [38] and highly similar regions (repeated sequences) were removed from the analysis. Genomes were downloaded from GenBank, including 13 STEC strains, the German outbreak EHAEC strain, 17 other *E. coli* and 2 *Shigella* strains, accession number for strains used are included in Additional file 3: Table S2. The concatenated nucleotide sequences of 341 orthologous CDSs from 34 strains genomes used are included in Additional file 5.

### MLST, serotyping, virulence factors and antimicrobial resistance by WGS analysis

MLST confirmation was performed by using the WGS and compared with the merged alleles sequences of each genes from the University of Warwick website (http://mlst.warwick.ac.uk/mlst/dbs/Ecoli). Allele comparison among STEC strains was performed by using DNA Dynamo sequence analysis software (Copyright© BlueTractorSoftware Ltd). In silico serotyping for previously described O and H type genes was conducted by using whole-genome sequencing (WGS) data using the BLAST tool SerotypeFinder 1.1 [64]. Virulence Factors of Pathogenic Bacteria database (VFDB) [65] was used for virulence factors screening. In addition, we used VirulenceFinder 1.5; which contain the *E. coli* virulence gene database [66]. The identification of acquired antimicrobial resistance genes was performed using a ResFinder 3.0 [67]. SerotypeFinder 1.1, VirulenceFinder 1.5 and ResFinder 3.0 web servers can be access at Center for Genomic Epidemiology (CGE) database (http://www.genomicepidemiology.org/).

### Plasmid identification

Initial identification of plasmids in O145:H25 strain EN1I-0044-2 genome was achieved using the PlasmidFinder 1.3 tool available on the CGE webserver [68]. Nucleotide sequence of the identified contigs with high probability coming from plasmid were used for a BLAST search in NCBI website (https://blast.ncbi.nlm.nih.gov/Blast.cgi). Progressive Mauve was used to generate alignment and perform comparison analysis with plasmids sequences producing significant alignments obtained from NCBI [69].

### Prophages, integrated elements and genomic island identification

Initial identification and annotation of prophage sequences within O145:H25 strain EN1I-0044-2 genome was performed using PHASTER web server [70]. EIs such as genomic islands (GIs) were initially predicted by an integrated interface for computational identification and visualization of genomic islands “Islandviewer4” [71]. All prophages and EIs were examined manually for accuracy of the prediction using Mauve by locating integrases and potential integration sites [69] or by CLC Genomics Workbench 11.0.1 (CLC Bio, Qiagen, Aarhus, Denmark) (Table 3). Phylogenetic tree of the stx2a prophage of O145:H25 strain EN1I-0044-2 and other STEC strains was performed using by CLC Genomics Workbench 11.0.1 (CLC Bio, Qiagen, Aarhus, Denmark).

Genomic island detection was performed by BLAST analysis by using genomic islands sequences previously identified in STEC O157:H7 strains EDL933 (Additional file 1: Table S2) [50]. Fimbrial gene cluster were identified by BLAST analysis using the locus tag of each gene in each gene clusters present in O145:H25 strain CFSAN004176 and other STEC as previously described [32].

### Insertion sequence (IS) identification

Initial IS elements identification and location was conducted using ISfinder webserver database [72]. Number of copies of each one of the identified IS elements were detected by nucleotide BLAST of the IS element and the genome of O145:H25 strain EN1I-0044-2 using Blastn suit from NCBI (https://blast.ncbi.nlm.nih.gov/Blast.cgi). Only highly similar sequences with ≥90% of coverage and ≥ 90% of identity to the identified IS elements were considered for the analysis. We used the same parameters to identify IS elements previously described for O145:H25 strains CFSAN004176 and CFSAN004177 [32]. IS elements identified either in our strain and the other two O145:H25 strains were used for comparative analysis (Additional file 2: Table S2).

## Supplementary information

**Additional file 1: Table S1.** Non-homologous region analysis. **Table S2.** O-islands analysis. **Table S3.** Fimbrial gene cluster analysis. Table S4. Sequence annotation of pEN1I-0044-2_03 plasmid by RAST.

**Additional file 2: Table S1.** Insertion sequence analysis. **Table S2.** LEE- and non-LEE-encoded effectors analysis.

**Additional file 3: Table S1**. Strains used for comparative analysis. **Table S2.** Strains used for phylogenetic analysis.

**Additional file 4: **List of the 341 conserved CDSs from *E. coli* K-12 MG1655 strain.

**Additional file 5.** Concatenated nucleotide sequences of 341 orthologous CDSs from 34 bacterial strains.

**Additional file 6: Figure S1.** Stx2a prophages gene alignment and evolutionary relationships. **Panel A**, Diagram of gene alignment of Stx2a phage from STEC O145:H25 CFASN004177 and phage P09 from study strain STEC O145:H25 EN1I-0044-2. Annotated sequence of Stx2a prophage from strain CFSAN004177 was taken from GenBank: [CP014670.1], position start: 4,500,642 and position end: 4,545,386. Prophage alignment was performed using Mauve (Darling AE, Mau B, Perna NT., 2010). Numbers indicate ORFs. Numbers in read correspond to virulence genes, among them the stx2A and B genes. **Panel B**, Phylogenetic tree of P09 phage sequence compared with stx2a prophages from O145:H25, O145:H28, O157, and additional non-O157 serogroups. The maximum likelihood (ML)-based phylogenetic tree based on phage sequence comparisons.

## Data Availability

This Whole Genome Shotgun project for the STEC O145:H25 strain EN1I-0044-2 has been deposited at the NCBI GenBank under the accession numbers QQVX00000000 and PRJNA448001 (https://www.ncbi.nlm.nih.gov/bioproject/PRJNA448001). All the other supporting data are included as additional files. Non-homologous regions are described in Additional file 1: Table S1. The accession number of strains used for the comparative analysis are included in Additional file 3: Table S1. The accession number of strains used for the whole-genome based phylogenetic analysis are included in Additional file 3: Table S2. The 341 non-recombinogenic CDSs from *E. coli* K-12 MG1655 strain used for phylogenetic analysis are included in Additional file 4. Concatenated nucleotide sequences of 341 orthologous CDSs from 34 strains used for phylogenetic analysis are included in Additional file 5.

## Abbreviations

STEC: Shiga toxin-producing *Escherichia coli*
EHEC: Enterohemorrhagic *Escherichia coli*
NVSN: National Vaccine Surveillance Network
HUS: Hemolytic uremic syndrome
LEE: Locus of enterocyte effacement
CDS: Coding DNA sequence
T3SS: Type III secretion system
MGEs: Mobile genetics elements
O-islands: Genomics islands
