# Supplementary material for: Comparative genomic analysis of a Shiga toxin-producing Escherichia coli (STEC) O145:H25 associated with a severe pediatric case of hemolytic uremic syndrome in Davidson County, Tennessee, US
Source: BMC Genomics. 2020 Aug 17;21:564. doi: 10.1186/s12864-020-06967-3 (PMC7437938; doi:10.1186/s12864-020-06967-3)
Supplement: Supplementary file 2 — Additional file 2: Table S1. Insertion sequence analysis. Table S2. LEE- and non-LEE-encoded effectors analysis. [file 12864_2020_6967_MOESM2_ESM.docx]

**Additional file 2.**

**Table S1: IS element producing significant alignments present on STEC chromosome (coverage ≥ 90%, identity ≥ 90%)**

| **IS element** | **IS**  **family** | **size (bp)** | **O145:H25**  **str. EN1I-0044-2** | **O145:H25**  **str. CFSAN004177** | **O145:H28**  **str. RM13514** | **O26:H11**  **str. 11368** | **O103:H2**  **str. 12009** | **O111: HNM**  **str. 11128** | **O157:H7**  **str. Sakai** |
| --- | --- | --- | --- | --- | --- | --- | --- | --- | --- |
| IS1H | IS1 | 764 | 1 | 1 | 0 | 1 | 1 | 1 | 1 |
| IS1F | IS1 | 768 | 0 | 0 | 2 | 4 | 1 | 2 | 1 |
| IS2 | IS3 | 1331 | 0 | 0 | 0 | 0 | 1 | 1 | 1 |
| IS3 | IS3 | 1258 | 0 | 0 | 0 | 2 | 1 | 1 | 0 |
| **IS600** | **IS3** | **1264** | **38** | **49** | **8** | **6** | **0** | **0** | **0** |
| **IS629** | **IS3** | **1310** | **0** | **0** | **29** | **6** | **23** | **34** | **16** |
| IS911 | IS3 | 1250 | 4 | 4 | 1 | 1 | 0 | 0 | 0 |
| ISCfr6 | IS3 | 1258 | 2 | 2 | 1 | 0 | 1 | 4 | 0 |
| ISEc16 | IS3 | 1244 | 0 | 0 | 0 | 1 | 0 | 0 | 0 |
| ISEc31 | IS3 | 1258 | 0 | 0 | 0 | 0 | 0 | 0 | 1 |
| ISEc48 | IS3 | 1336 | 1 | 1 | 0 | 0 | 0 | 0 | 0 |
| IS30 | IS30 | 1221 | 0 | 0 | 0 | 0 | 0 | 0 | 1 |
| IS682 | IS66 | 2533 | 0 | 0 | 1 | 1 | 0 | 1 | 1 |
| ISEc8 | IS66 | 2442 | 0 | 2 | 1 | 2 | 1 | 0 | 8 |
| ISEc22 | IS66 | 2454 | 0 | 0 | 0 | 6 | 0 | 0 | 0 |
| ISEc23 | IS66 | 2532 | 1 | 6 | 0 | 0 | 0 | 1 | 0 |
| ISCro1 | IS66 | 2699 | 0 | 0 | 0 | 9 | 1 | 0 | 0 |
| IS91 | IS91 | 1829 | 0 | 0 | 0 | 0 | 0 | 2 | 0 |
| IS621 | IS110 | 1425 | 0 | 0 | 0 | 13 | 12 | 0 | 0 |
| ISEc20 | IS110 | 1459 | 0 | 0 | 7 | 0 | 1 | 1 | 0 |
| ISEc38 | ISL3 | 1722 | 1 | 2 | 1 | 2 | 1 | 0 | 0 |
| IS609 | IS200/IS605 | 1748 | 1 | 1 | 2 | 3 | 2 | 3 | 2 |
| ISEc1 | ISAs1 | 1291 | 5 | 5 | 0 | 4 | 3 | 4 | 4 |
| ISEc26 | ISAs1 | 1305 | 0 | 0 | 0 | 1 | 1 | 1 | 1 |
| **Total** |  |  | **54** | **73** | **53** | **62** | **50** | **56** | **37** |

| **IS element** | **IS**  **family** | **size (bp)** | **O145:H25**  **str. EN1I-0044-2** | **O145:H25**  **str. CFSAN004177** | **O145:H28**  **str. RM13514** | **O26:H11**  **str. 11368** | **O103:H2**  **str. 12009** | **O111: HNM**  **str. 11128** | **O157:H7**  **str. Sakai** |
| --- | --- | --- | --- | --- | --- | --- | --- | --- | --- |
| **pEHEC-like plasmid** | | |  |  |  |  |  |  |  |
| IS600 | IS3 | 1264 | 0 | 1 | 1 | 2 | 0 | 0 | 0 |
| IS629 | IS3 | 1310 | 0 | 0 | 2 | 1 | 6 | 3 | 1 |
| IS911 | IS3 | 1250 | 0 | 0 | 1 | 1 | 0 | 0 | 1 |
| IS1203E | IS3 | 1032 | 0 | 0 | 0 | 0 | 0 | 0 | 1 |
| ISCfr6 | IS3 | 1258 | 0 | 0 | 0 | 0 | 0 | 0 | 0 |
| ISEc8 | IS66 | 2442 | 0 | 0 | 0 | 0 | 0 | 2 | 0 |
| ISEc23 | IS66 | 2532 | 1 | 0 | 0 | 0 | 0 | 0 | 0 |
| IS91 | IS91 | 1829 | 1 | 1 | 2 | 2 | 0 | 3 | 0 |
| ISSbo1 | IS91 | 1709 | 1 | 1 | 0 | 0 | 0 | 0 | 0 |
| ISEc76 | IS110 | 1368 | 1 | 1 | 0 | 0 | 0 | 0 | 0 |
| **Secondary plasmids** | | |  |  |  |  |  |  |  |
| IS1R | IS1 | 768 | 0 | 0 | 0 | 0 | NA | 4 | 0 |
| IS600 | IS3 | 1264 | 1 | 2 | 0 | 0 | NA | 0 | 0 |
| IS629 | IS3 | 1264 | 0 | 0 | 2 | 0 | NA | 1 | 0 |
| IS1203 | IS3 | 1310 | 0 | 0 | 0 | 0 | NA | 5 | 0 |
| ISEc25 | IS3 | 1310 | 1 | 1 | 0 | 0 | NA | 0 | 0 |
| IS10L | IS4 | 1329 | 0 | 0 | 0 | 0 | NA | 1 | 0 |
| IS26 | IS6 | 820 | 0 | 0 | 3 | 0 | NA | 4 | 0 |
| ISEc23 | IS66 | 2532 | 1 | 1 | 0 | 0 | NA | 0 | 0 |
| IS1294 | IS91 | 1689 | 0 | 0 | 0 | 1 | NA | 0 | 0 |
| ISSbo1 | IS91 | 1709 | 1 | 1 | 0 | 0 | NA | 0 | 0 |
| ISVsa3 | IS91 | 977 | 0 | 0 | 1 | 0 | NA | 0 | 0 |
| **Total** |  |  | **8** | **9** | **12** | **7** | **6** | **23** | **3** |

**Table S2: Comparison of IS elements present on STEC plasmids using BLASTN (coverage ≥ 90%, identity ≥ 90%)**

**Table S3: LEE- and non-LEE-encoded effectors present on STEC strains**

| **Effector** | **O145:H25**  **str. EN1I-0044-2** | **O145:H25**  **str. CFSAN004177** | **O145:H28**  **str. RM13514** | **O26:H11**  **str. 11368** | **O103:H2**  **str. 12009** | **O111: HNM**  **str. 11128** | **O157:H7**  **str. Sakai** |
| --- | --- | --- | --- | --- | --- | --- | --- |
| *espA* (variant) | 1 (β) | 1 (β) | 1 (γ) | 1 (β) | 1 (β) | 1 (α) | 1 (γ) |
| *espB* (variant) | 1 (β) | 1 (β) | 1 (γ) | 1 (β) | 1 (β) | 1 (α) | 1 (γ) |
| *espD* (variant) | 1 (β) | 1 (β) | 1 (γ) | 1 (β) | 1 (β) | 1 (α) | 1 (γ) |
| *espF* | 1 | 1 | 1 | 1 | 1 | 1 | 1 |
| *espG* | 1 | 1 | 1 | 1 | 1 | 1 | 1 |
| *espH* | 1 | 1 | 1 | 1 | 1 | 1 | 1 |
| *espJ* | 2 | 2 | 1 | 1 | 0 | 1 | 1 |
| *espK* | 1 | 1 | 1 | 2 | 2 | 1 | 1 |
| *espL* | 1 | 2 | 1 | 1 | 2 | 2 | 1 |
| *espM* | 1 | 1 | 1 | 1 | 1 | 1 | 1 |
| *espN* | 1 | 1 | 1 | 1 | 1 | 1 | 1 |
| *espO* | 1 | 1 | 2 | 2 | 1 | 2 | 2 |
| *espR* | 1 | 1 | 1 | 1 | 1 | 1 | 1 |
| *espS* | 2 (1) | 2 | 2 | 2 (1) | 2 | 1 | 0 |
| *espV* | 1 (1) | 1 (1) | 2 (1) | 1 (1) | 1 (1) | 1 (1) | 1 (1) |
| *espW* | 1 | 1 | 0 | 1 | 1 | 1 | 1 |
| *espX* | 2 | 2 | 1 | 1 | 1 | 1 | 1 |
| *espZ* (*sepZ*) | 1 | 1 | 1 | 1 | 1 | 1 | 1 |
| *Map* | 1 | 1 | 1 | 1 | 1 | 1 | 1 |
| *nleA* | 1 | 1 | 1 | 2 | 1 | 1 | 1 |
| *nleB* | 1 | 2 | 3 (1) | 1 | 4 | 3 (1) | 3 (1) |
| *nleC* | 3 | 3 | 2 (1) | 1 | 2 (2) | 2 | 1 |
| *nleD* | 0 | 0 | 0 | 0 | 0 | 0 | 1 |
| *nleE* | 2 | 2 | 1 | 1 | 2 | 2 | 1 |
| *nleF* | 1 | 1 | 1 | 1 | 1 | (1) | 1 |
| *nleG* | 4 | 9 | 5 | 13 | 7 (1) | 10 | 13 (2) |
| *nleH* | 2 | 2 | 3 (1) | 2 | 2 | 2 | 2 |
| *tccp* | 1 | 1 | 1 | 1 | 1 | 1 | 1 (1) |
| *tir* (variant) | 1 (β) | 1 (β) | 1 (γ) | 1 (β) | 1 (β) | 1 (α) | 1 (γ) |
| *cif* | 1 (1) | 1 (1) | 1 | 1 (1) | 1 (1) | 1 (1) | 0 |
| **Total** | 40 (3) | 46 (2) | 40 (4) | 46 (3) | 43 (5) | 45 (4) | 45 (5) |

Gene presence (Coverage ≥ 80%, identity ≥ 90%); numbers of pseudogenes are shown in parentheses
