# Supplementary material for: Comparative genomic analysis of a Shiga toxin-producing Escherichia coli (STEC) O145:H25 associated with a severe pediatric case of hemolytic uremic syndrome in Davidson County, Tennessee, US
Source: BMC Genomics. 2020 Aug 17;21:564. doi: 10.1186/s12864-020-06967-3 (PMC7437938; doi:10.1186/s12864-020-06967-3)
Supplement: Supplementary file 3 — Additional file 3: Table S1. Strains used for comparative analysis. Table S2. Strains used for phylogenetic analysis. [file 12864_2020_6967_MOESM3_ESM.docx]

**ADDITIONAL FILE 3.**

**Table S1. Strains used for STEC comparative analysis**

| **Serotype** | **Strain** | **GenBank** |
| --- | --- | --- |
| O145:H25 (EcO145) | EN1I-0044-2 | This study |
|  | CFSAN004176 | NZ_CP014583 |
|  | CFSAN004177 | CP014670 |
| O145:H28 (EcO145) | RM13514 | CP006027 |
|  | RM13516 | CP006262 |
| O157:H7 (EcO157) | Sakai | NC_002695 |
| O103:H2 (EcO103) | 12009 | NC_013353 |
| O26:H11 (EcO26) | 11368 | NC_013361 |
| O111:HNM (EcO111) | 11128 | NC_013364 |
| O152:H28 (commensal) | SE11 | NC_011415 |

**Table S2. Reference strains used for Whole-genome based phylogenetic analysis**

| **Serotype** | **Strain** | **GenBank** | **ID for phylogenetic analysis** |
| --- | --- | --- | --- |
| O145:H25 (EcO145) | EN1I-0044-2 | This study | O145:H25 str. EN1I-0044-2 (STEC) |
|  | CFSAN004176 | NZ_CP014583 | O145:H25 str. CFSAN004176 (STEC) |
|  | CFSAN004177 | CP014670 | O145:H25 str. CFSAN004177 (STEC) |
| O145:H28 (EcO145) | RM13514 | CP006027 | O145:H28 str. RM13514 (STEC) |
|  | RM13516 | CP006262 | O145:H28 str. RM13516 (STEC) |
| O157:H7 (EcO157) | EDL933 | NC_002655 | O157:H7 str. EDL933 (STEC) |
|  | Sakai | NC_002695 | O157:H7 str. Sakai (STEC) |
|  | EC4115 | NC_011353 | O157:H7 str. EC4115 (STEC) |
|  | TW14359 | NC_013008 | O157:H7 str. TW14359 (STEC) |
|  | Xuzhou21 | NC_017906 | O157:H7 str. Xuzhou21 (STEC) |
| O103:H2 (EcO103) | 12009 | NC_013353 | O103:H2 str. 12009 (STEC) |
| O26:H11 (EcO26) | 11368 | NC_013361 | O26:H11 str. 11368 (STEC) |
| O111:HNM (EcO111) | 11128 | NC_013364 | O111:HNM str. 11128 (STEC) |
| O104 | German outbreak str. 2011C-3493 | NC_018658 | O104:H4 str. 2011C_3493 |
| O165:H25 | 2012C-4227 | CP013029 | O165:H25 str. 2012C-4227 (EHEC) |
| Other *E. coli* | W | NC_017635 | str. W (ATCC9637) |
|  | SE11 | NC_011415 | O152:H28 str. SE11 (commensal) |
|  | ATCC 8739 | NC_010468 | str. ATCC 8739 |
|  | HS | NC_009800 | O9 str. HS (commensal) |
|  | BL21(DE3) | NC_012971 | str.BL21(DE3) |
|  | K12 subst. MG1655 | NC_000913 | K-12 subst. MG1655 |
|  | UMNK88 | NC_017641 | O149 str. UMNK88 (ETEC) |
|  | *E. coli* O55 str. CB9615 | NC_013941 | O55:H7 str. CB9615 (EPEC) |
|  | *E. coli* O55 str. RM12579 | NC_017656 | O55:H7 str. RM12579 (EPEC) |
|  | 42 | NC_017626 | str. 042 (EAEC) |
|  | IAI39 | NC_011750 | str. IAI39 (UPEC) |
|  | *E. coli* O127 str. E2348/69 | NC_011601 | O127:H6 str. E2348/69 (EPEC) |
|  | NA114 | NC_017644 | Str. NA114 (UPEC) |
|  | *E. coli* O83 str. NRG 857C | NC_017634 | O83:H1 str. NRG 857C (AIEC) |
|  | CFT073 | NC_004431 | O6:H1 str. CFT073 (UPEC) |
|  | APEC O1 | NC_008563 | str. APEC O1 |
|  | UM146 | NC_017632 | str. UM146 (AIEC) |
| *Shigella* | *S. sonnei* Ss046 | NC_007384 | str. Ss046 (*S. sonnei*) |
|  | *S. dysenteriae* Sd197 | NC_007606 | str. Sd197 (*S. dysenteriae*) |
