# Supplementary material for: Comparative genomic analysis of a Shiga toxin-producing Escherichia coli (STEC) O145:H25 associated with a severe pediatric case of hemolytic uremic syndrome in Davidson County, Tennessee, US
Source: BMC Genomics. 2020 Aug 17;21:564. doi: 10.1186/s12864-020-06967-3 (PMC7437938; doi:10.1186/s12864-020-06967-3)
Supplement: Supplementary file 4 — Additional file 4: List of the 341 conserved CDSs from E. coli K-12 MG1655 strain. [file 12864_2020_6967_MOESM4_ESM.docx]

**Additional file 4:** List of the 341 conserved CDSs from *E. coli* K-12 MG1655 strain

>lcl|NC_000913.3_gene_3_gene=thrB_locus_tag=b0003_location=2801..3733

>lcl|NC_000913.3_gene_10_gene=satP_locus_tag=b0010_location=complement(9928..10494)

>lcl|NC_000913.3_gene_13_gene=dnaK_locus_tag=b0014_location=12163..14079

>lcl|NC_000913.3_gene_27_gene=lspA_locus_tag=b0027_location=25207..25701

>lcl|NC_000913.3_gene_28_gene=fkpB_locus_tag=b0028_location=25826..26275

>lcl|NC_000913.3_gene_46_gene=kefF_locus_tag=b0046_location=47246..47776

>lcl|NC_000913.3_gene_51_gene=rsmA_locus_tag=b0051_location=complement(51609..52430)

>lcl|NC_000913.3_gene_80_gene=cra_locus_tag=b0080_location=88028..89032

>lcl|NC_000913.3_gene_83_gene=ftsL_locus_tag=b0083_location=91032..91397

>lcl|NC_000913.3_gene_90_gene=murG_locus_tag=b0090_location=99644..100711

>lcl|NC_000913.3_gene_93_gene=ftsQ_locus_tag=b0093_location=103155..103985

>lcl|NC_000913.3_gene_96_gene=lpxC_locus_tag=b0096_location=106557..107474

>lcl|NC_000913.3_gene_101_gene=zapD_locus_tag=b0102_location=complement(111856..112599)

>lcl|NC_000913.3_gene_102_gene=coaE_locus_tag=b0103_location=complement(112599..113219)

>lcl|NC_000913.3_gene_108_gene=ampD_locus_tag=b0110_location=118733..119284

>lcl|NC_000913.3_gene_109_gene=ampE_locus_tag=b0111_location=119281..120135

>lcl|NC_000913.3_gene_119_gene=speE_locus_tag=b0121_location=complement(135598..136464)

>lcl|NC_000913.3_gene_124_gene=can_locus_tag=b0126_location=complement(142008..142670)

>lcl|NC_000913.3_gene_126_gene=yadH_locus_tag=b0128_location=143702..144472

>lcl|NC_000913.3_gene_129_gene=panD_locus_tag=b0131_location=complement(146314..146694)

>lcl|NC_000913.3_gene_144_gene=sfsA_locus_tag=b0146_location=complement(160782..161486)

>lcl|NC_000913.3_gene_155_gene=yadS_locus_tag=b0157_location=complement(177001..177624)

>lcl|NC_000913.3_gene_157_gene=mtn_locus_tag=b0159_location=complement(178455..179153)

>lcl|NC_000913.3_gene_161_gene=yaeH_locus_tag=b0163_location=complement(183709..184095)

>lcl|NC_000913.3_gene_163_gene=dapD_locus_tag=b0166_location=complement(185123..185947)

>lcl|NC_000913.3_gene_169_gene=pyrH_locus_tag=b0171_location=191855..192580

>lcl|NC_000913.3_gene_179_gene=lpxA_locus_tag=b0181_location=202560..203348

>lcl|NC_000913.3_gene_180_gene=lpxB_locus_tag=b0182_location=203348..204496

>lcl|NC_000913.3_gene_195_gene=rcsF_locus_tag=b0196_location=complement(219591..219995)

>lcl|NC_000913.3_gene_198_gene=metN_locus_tag=b0199_location=complement(221614..222645)

>lcl|NC_000913.3_gene_207_gene=yafC_locus_tag=b0208_location=complement(229967..230881)

>lcl|NC_000913.3_gene_221_gene=ivy_locus_tag=b0220_location=240343..240816

>lcl|NC_000913.3_gene_239_gene=gpt_locus_tag=b0238_location=255977..256435

>lcl|NC_000913.3_gene_241_gene=crl_locus_tag=b0240_location=join(257829..257899,258676..259006)

>lcl|NC_000913.3_gene_386_gene=yaiY_locus_tag=b0379_location=complement(399025..399333)

>lcl|NC_000913.3_gene_395_gene=aroL_locus_tag=b0388_location=406405..406929

>lcl|NC_000913.3_gene_407_gene=brnQ_locus_tag=b0401_location=419591..420910

>lcl|NC_000913.3_gene_412_gene=tgt_locus_tag=b0406_location=426137..427264

>lcl|NC_000913.3_gene_419_gene=nrdR_locus_tag=b0413_location=433002..433451

>lcl|NC_000913.3_gene_439_gene=ampG_locus_tag=b0433_location=complement(452070..453545)

>lcl|NC_000913.3_gene_449_gene=fadM_locus_tag=b0443_location=464402..464800

>lcl|NC_000913.3_gene_474_gene=ybaN_locus_tag=b0468_location=490882..491259

>lcl|NC_000913.3_gene_478_gene=recR_locus_tag=b0472_location=494405..495010

>lcl|NC_000913.3_gene_481_gene=hemH_locus_tag=b0475_location=498055..499017

>lcl|NC_000913.3_gene_613_gene=entH_locus_tag=b0597_location=629300..629713

>lcl|NC_000913.3_gene_622_gene=ahpC_locus_tag=b0605_location=638945..639508

>lcl|NC_000913.3_gene_640_gene=flc_locus_tag=b0624_location=complement(657555..657938)

>lcl|NC_000913.3_gene_658_gene=ybeL_locus_tag=b0643_location=675018..675500

>lcl|NC_000913.3_gene_673_gene=ybeX_locus_tag=b0658_location=complement(690906..691784)

>lcl|NC_000913.3_gene_674_gene=ybeY_locus_tag=b0659_location=complement(691874..692341)

>lcl|NC_000913.3_gene_688_gene=nagA_locus_tag=b0677_location=complement(701603..702751)

>lcl|NC_000913.3_gene_694_gene=fur_locus_tag=b0683_location=complement(710200..710646)

>lcl|NC_000913.3_gene_736_gene=sdhD_locus_tag=b0722_location=755560..755907

>lcl|NC_000913.3_gene_738_gene=sdhB_locus_tag=b0724_location=757689..758405

>lcl|NC_000913.3_gene_740_gene=sucB_locus_tag=b0727_location=761522..762739

>lcl|NC_000913.3_gene_749_gene=ybgE_locus_tag=b0735_location=774309..774602

>lcl|NC_000913.3_gene_750_gene=ybgC_locus_tag=b0736_location=774752..775156

>lcl|NC_000913.3_gene_752_gene=tolR_locus_tag=b0738_location=775849..776277

>lcl|NC_000913.3_gene_787_gene=ybhB_locus_tag=b0773_location=complement(807433..807909)

>lcl|NC_000913.3_gene_796_gene=moaB_locus_tag=b0782_location=818055..818567

>lcl|NC_000913.3_gene_797_gene=moaC_locus_tag=b0783_location=818570..819055

>lcl|NC_000913.3_gene_825_gene=glnH_locus_tag=b0811_location=complement(847258..848004)

>lcl|NC_000913.3_gene_831_gene=mntR_locus_tag=b0817_location=853183..853650

>lcl|NC_000913.3_gene_833_gene=ldtB_locus_tag=b0819_location=complement(854824..855744)

>lcl|NC_000913.3_gene_862_gene=ybjL_locus_tag=b0847_location=complement(888134..889819)

>lcl|NC_000913.3_gene_863_gene=ybjM_locus_tag=b0848_location=890089..890466

>lcl|NC_000913.3_gene_865_gene=ybjC_locus_tag=b0850_location=890913..891200

>lcl|NC_000913.3_gene_867_gene=rimK_locus_tag=b0852_location=891967..892869

>lcl|NC_000913.3_gene_876_gene=artM_locus_tag=b0861_location=complement(900866..901534)

>lcl|NC_000913.3_gene_877_gene=artQ_locus_tag=b0862_location=complement(901534..902250)

>lcl|NC_000913.3_gene_881_gene=ybjQ_locus_tag=b0866_location=904593..904916

>lcl|NC_000913.3_gene_925_gene=cmk_locus_tag=b0910_location=961201..961884

>lcl|NC_000913.3_gene_926_gene=rpsA_locus_tag=b0911_location=961995..963668

>lcl|NC_000913.3_gene_934_gene=ycbJ_locus_tag=b0919_location=971752..972645

>lcl|NC_000913.3_gene_942_gene=ycbL_locus_tag=b0927_location=983650..984297

>lcl|NC_000913.3_gene_943_gene=aspC_locus_tag=b0928_location=complement(984519..985709)

>lcl|NC_000913.3_gene_952_gene=ssuE_locus_tag=b0937_location=complement(996937..997512)

>lcl|NC_000913.3_gene_976_gene=yccF_locus_tag=b0961_location=complement(1023902..1024348)

>lcl|NC_000913.3_gene_979_gene=yccT_locus_tag=b0964_location=complement(1027111..1027773)

>lcl|NC_000913.3_gene_985_gene=yccA_locus_tag=b0970_location=complement(1030759..1031418)

>lcl|NC_000913.3_gene_1013_gene=torR_locus_tag=b0995_location=complement(1057262..1057954)

>lcl|NC_000913.3_gene_1017_gene=cbpM_locus_tag=b0999_location=complement(1062550..1062855)

>lcl|NC_000913.3_gene_1018_gene=cbpA_locus_tag=b1000_location=complement(1062855..1063775)

>lcl|NC_000913.3_gene_1050_gene=ycdY_locus_tag=b1035_location=1099640..1100194

>lcl|NC_000913.3_gene_1060_gene=ymdB_locus_tag=b1045_location=1105820..1106353

>lcl|NC_000913.3_gene_1076_gene=pyrC_locus_tag=b1062_location=complement(1121561..1122607)

>lcl|NC_000913.3_gene_1078_gene=grxB_locus_tag=b1064_location=complement(1123407..1124054)

>lcl|NC_000913.3_gene_1080_gene=rimJ_locus_tag=b1066_location=1125562..1126146

>lcl|NC_000913.3_gene_1106_gene=fabH_locus_tag=b1091_location=1148759..1149712

>lcl|NC_000913.3_gene_1116_gene=ptsG_locus_tag=b1101_location=1157869..1159302

>lcl|NC_000913.3_gene_1119_gene=ycfL_locus_tag=b1104_location=1162247..1162624

>lcl|NC_000913.3_gene_1121_gene=thiK_locus_tag=b1106_location=1163260..1164084

>lcl|NC_000913.3_gene_1144_gene=phoQ_locus_tag=b1129_location=complement(1188316..1189776)

>lcl|NC_000913.3_gene_1145_gene=phoP_locus_tag=b1130_location=complement(1189776..1190447)

>lcl|NC_000913.3_gene_1192_gene=minE_locus_tag=b1174_location=complement(1224279..1224545)

>lcl|NC_000913.3_gene_1193_gene=minD_locus_tag=b1175_location=complement(1224549..1225361)

>lcl|NC_000913.3_gene_1198_gene=ycgM_locus_tag=b1180_location=1228079..1228738

>lcl|NC_000913.3_gene_1201_gene=umuD_locus_tag=b1183_location=1230767..1231186

>lcl|NC_000913.3_gene_1221_gene=ychF_locus_tag=b1203_location=complement(1256721..1257812)

>lcl|NC_000913.3_gene_1223_gene=ychH_locus_tag=b1205_location=1258791..1259069

>lcl|NC_000913.3_gene_1226_gene=ispE_locus_tag=b1208_location=complement(1262026..1262877)

>lcl|NC_000913.3_gene_1230_gene=prmC_locus_tag=b1212_location=1266094..1266927

>lcl|NC_000913.3_gene_1231_gene=ychQ_locus_tag=b1213_location=1266924..1267316

>lcl|NC_000913.3_gene_1243_gene=ychN_locus_tag=b1219_location=complement(1273246..1273599)

>lcl|NC_000913.3_gene_1256_gene=purU_locus_tag=b1232_location=complement(1287782..1288624)

>lcl|NC_000913.3_gene_1259_gene=rssB_locus_tag=b1235_location=1290242..1291255

>lcl|NC_000913.3_gene_1261_gene=hns_locus_tag=b1237_location=complement(1292509..1292922)

>lcl|NC_000913.3_gene_1267_gene=oppB_locus_tag=b1244_location=1302899..1303819

>lcl|NC_000913.3_gene_1268_gene=oppC_locus_tag=b1245_location=1303834..1304742

>lcl|NC_000913.3_gene_1277_gene=yciA_locus_tag=b1253_location=complement(1311848..1312246)

>lcl|NC_000913.3_gene_1278_gene=yciB_locus_tag=b1254_location=complement(1312351..1312890)

>lcl|NC_000913.3_gene_1279_gene=yciC_locus_tag=b1255_location=complement(1312920..1313663)

>lcl|NC_000913.3_gene_1305_gene=yciS_locus_tag=b1279_location=1340243..1340551

>lcl|NC_000913.3_gene_1310_gene=yciT_locus_tag=b1284_location=complement(1343597..1344346)

>lcl|NC_000913.3_gene_1332_gene=pspA_locus_tag=b1304_location=1368079..1368747

>lcl|NC_000913.3_gene_1352_gene=tpx_locus_tag=b1324_location=complement(1388305..1388811)

>lcl|NC_000913.3_gene_1373_gene=ydaN_locus_tag=b1342_location=1408050..1409033

>lcl|NC_000913.3_gene_1415_gene=ldhA_locus_tag=b1380_location=complement(1441854..1442843)

>lcl|NC_000913.3_gene_1642_gene=pntB_locus_tag=b1602_location=complement(1674972..1676360)

>lcl|NC_000913.3_gene_1646_gene=folM_locus_tag=b1606_location=1680976..1681698

>lcl|NC_000913.3_gene_1660_gene=malI_locus_tag=b1620_location=complement(1698152..1699180)

>lcl|NC_000913.3_gene_1663_gene=add_locus_tag=b1623_location=1702233..1703234

>lcl|NC_000913.3_gene_1668_gene=rsxA_locus_tag=b1627_location=1705767..1706348

>lcl|NC_000913.3_gene_1669_gene=rsxB_locus_tag=b1628_location=1706348..1706926

>lcl|NC_000913.3_gene_1671_gene=rsxD_locus_tag=b1630_location=1709142..1710200

>lcl|NC_000913.3_gene_1672_gene=rsxG_locus_tag=b1631_location=1710204..1710824

>lcl|NC_000913.3_gene_1673_gene=rsxE_locus_tag=b1632_location=1710828..1711523

>lcl|NC_000913.3_gene_1674_gene=nth_locus_tag=b1633_location=1711523..1712158

>lcl|NC_000913.3_gene_1679_gene=pdxH_locus_tag=b1638_location=complement(1717351..1718007)

>lcl|NC_000913.3_gene_1682_gene=slyB_locus_tag=b1641_location=1719876..1720343

>lcl|NC_000913.3_gene_1692_gene=gloA_locus_tag=b1651_location=1727837..1728244

>lcl|NC_000913.3_gene_1693_gene=rnt_locus_tag=b1652_location=1728347..1728994

>lcl|NC_000913.3_gene_1695_gene=grxD_locus_tag=b1654_location=complement(1733754..1734101)

>lcl|NC_000913.3_gene_1697_gene=sodB_locus_tag=b1656_location=1735378..1735959

>lcl|NC_000913.3_gene_1702_gene=ydhC_locus_tag=b1660_location=1739911..1741122

>lcl|NC_000913.3_gene_1721_gene=sufE_locus_tag=b1679_location=complement(1758874..1759290)

>lcl|NC_000913.3_gene_1729_gene=menI_locus_tag=b1686_location=complement(1765222..1765632)

>lcl|NC_000913.3_gene_1747_gene=ppsR_locus_tag=b1703_location=1787445..1788278

>lcl|NC_000913.3_gene_1752_gene=nlpC_locus_tag=b1708_location=complement(1792267..1792731)

>lcl|NC_000913.3_gene_1768_gene=ydiZ_locus_tag=b1724_location=1807400..1807690

>lcl|NC_000913.3_gene_1769_gene=yniA_locus_tag=b1725_location=1807796..1808656

>lcl|NC_000913.3_gene_1770_gene=yniB_locus_tag=b1726_location=complement(1808697..1809233)

>lcl|NC_000913.3_gene_1771_gene=yniC_locus_tag=b1727_location=1809380..1810048

>lcl|NC_000913.3_gene_1783_gene=osmE_locus_tag=b1739_location=complement(1821918..1822256)

>lcl|NC_000913.3_gene_1826_gene=mipA_locus_tag=b1782_location=complement(1865726..1866472)

>lcl|NC_000913.3_gene_1859_gene=nudL_locus_tag=b1813_location=1896170..1896748

>lcl|NC_000913.3_gene_1863_gene=manX_locus_tag=b1817_location=1902048..1903019

>lcl|NC_000913.3_gene_1868_gene=rlmA_locus_tag=b1822_location=complement(1906251..1907060)

>lcl|NC_000913.3_gene_1871_gene=yebO_locus_tag=b1825_location=complement(1908261..1908548)

>lcl|NC_000913.3_gene_1888_gene=yebY_locus_tag=b1839_location=complement(1923365..1923706)

>lcl|NC_000913.3_gene_1899_gene=eda_locus_tag=b1850_location=complement(1932115..1932756)

>lcl|NC_000913.3_gene_1907_gene=znuC_locus_tag=b1858_location=1942662..1943417

>lcl|NC_000913.3_gene_1962_gene=pgsA_locus_tag=b1912_location=complement(1992269..1992817)

>lcl|NC_000913.3_gene_2118_gene=dcd_locus_tag=b2065_location=complement(2141634..2142215)

>lcl|NC_000913.3_gene_2184_gene=yehW_locus_tag=b2128_location=complement(2215745..2216476)

>lcl|NC_000913.3_gene_2200_gene=sanA_locus_tag=b2144_location=2232878..2233597

>lcl|NC_000913.3_gene_2206_gene=mglB_locus_tag=b2150_location=complement(2239350..2240348)

>lcl|NC_000913.3_gene_2209_gene=folE_locus_tag=b2153_location=complement(2242984..2243652)

>lcl|NC_000913.3_gene_2224_gene=fruK_locus_tag=b2168_location=complement(2261427..2262365)

>lcl|NC_000913.3_gene_2225_gene=fruB_locus_tag=b2169_location=complement(2262365..2263495)

>lcl|NC_000913.3_gene_2232_gene=mepS_locus_tag=b2175_location=2269979..2270545

>lcl|NC_000913.3_gene_2253_gene=ccmE_locus_tag=b2197_location=complement(2294901..2295380)

>lcl|NC_000913.3_gene_2258_gene=napC_locus_tag=b2202_location=complement(2297657..2298259)

>lcl|NC_000913.3_gene_2261_gene=napG_locus_tag=b2205_location=complement(2299565..2300260)

>lcl|NC_000913.3_gene_2292_gene=yfaE_locus_tag=b2236_location=2348514..2348768

>lcl|NC_000913.3_gene_2313_gene=arnD_locus_tag=b2256_location=2370018..2370908

>lcl|NC_000913.3_gene_2320_gene=menB_locus_tag=b2262_location=complement(2375962..2376819)

>lcl|NC_000913.3_gene_2321_gene=menH_locus_tag=b2263_location=complement(2376834..2377592)

>lcl|NC_000913.3_gene_2324_gene=elaB_locus_tag=b2266_location=complement(2380722..2381027)

>lcl|NC_000913.3_gene_2337_gene=nuoK_locus_tag=b2279_location=complement(2395043..2395345)

>lcl|NC_000913.3_gene_2339_gene=nuoI_locus_tag=b2281_location=complement(2395908..2396450)

>lcl|NC_000913.3_gene_2343_gene=nuoE_locus_tag=b2285_location=complement(2401552..2402052)

>lcl|NC_000913.3_gene_2345_gene=nuoB_locus_tag=b2287_location=complement(2403951..2404613)

>lcl|NC_000913.3_gene_2346_gene=nuoA_locus_tag=b2288_location=complement(2404629..2405072)

>lcl|NC_000913.3_gene_2348_gene=alaA_locus_tag=b2290_location=2407561..2408778

>lcl|NC_000913.3_gene_2353_gene=yfbV_locus_tag=b2295_location=complement(2412677..2413132)

>lcl|NC_000913.3_gene_2354_gene=ackA_locus_tag=b2296_location=2413470..2414672

>lcl|NC_000913.3_gene_2357_gene=yfcD_locus_tag=b2299_location=complement(2418634..2419176)

>lcl|NC_000913.3_gene_2360_gene=yfcG_locus_tag=b2302_location=2420621..2421268

>lcl|NC_000913.3_gene_2371_gene=cvpA_locus_tag=b2313_location=complement(2430275..2430763)

>lcl|NC_000913.3_gene_2383_gene=yfcL_locus_tag=b2325_location=complement(2443891..2444169)

>lcl|NC_000913.3_gene_2386_gene=mepA_locus_tag=b2328_location=complement(2445560..2446384)

>lcl|NC_000913.3_gene_2489_gene=murQ_locus_tag=b2428_location=2545773..2546669

>lcl|NC_000913.3_gene_2496_gene=amiA_locus_tag=b2435_location=2552352..2553221

>lcl|NC_000913.3_gene_2533_gene=yffB_locus_tag=b2471_location=2591247..2591603

>lcl|NC_000913.3_gene_2543_gene=bcp_locus_tag=b2480_location=2600478..2600948

>lcl|NC_000913.3_gene_2556_gene=yfgO_locus_tag=b2493_location=complement(2614820..2615881)

>lcl|NC_000913.3_gene_2563_gene=purN_locus_tag=b2500_location=2622234..2622872

>lcl|NC_000913.3_gene_2576_gene=yfgM_locus_tag=b2513_location=complement(2638663..2639283)

>lcl|NC_000913.3_gene_2581_gene=ndk_locus_tag=b2518_location=complement(2644433..2644864)

>lcl|NC_000913.3_gene_2593_gene=iscU_locus_tag=b2529_location=complement(2659903..2660289)

>lcl|NC_000913.3_gene_2595_gene=iscR_locus_tag=b2531_location=complement(2661643..2662131)

>lcl|NC_000913.3_gene_2597_gene=suhB_locus_tag=b2533_location=2663442..2664245

>lcl|NC_000913.3_gene_2617_gene=hmp_locus_tag=b2552_location=2685835..2687025

>lcl|NC_000913.3_gene_2633_gene=recO_locus_tag=b2565_location=complement(2701741..2702469)

>lcl|NC_000913.3_gene_2635_gene=rnc_locus_tag=b2567_location=complement(2703383..2704063)

>lcl|NC_000913.3_gene_2639_gene=rseB_locus_tag=b2571_location=complement(2707798..2708754)

>lcl|NC_000913.3_gene_2650_gene=trxC_locus_tag=b2582_location=2718735..2719154

>lcl|NC_000913.3_gene_2663_gene=rluD_locus_tag=b2594_location=complement(2735031..2736011)

>lcl|NC_000913.3_gene_2664_gene=bamD_locus_tag=b2595_location=2736146..2736883

>lcl|NC_000913.3_gene_2682_gene=nadK_locus_tag=b2615_location=2750831..2751709

>lcl|NC_000913.3_gene_2687_gene=smpB_locus_tag=b2620_location=2754896..2755378

>lcl|NC_000913.3_gene_2726_gene=ygaU_locus_tag=b2665_location=complement(2796337..2796786)

>lcl|NC_000913.3_gene_2728_gene=ygaV_locus_tag=b2667_location=2797211..2797510

>lcl|NC_000913.3_gene_2730_gene=stpA_locus_tag=b2669_location=complement(2798091..2798495)

>lcl|NC_000913.3_gene_2731_gene=alaE_locus_tag=b2670_location=2799164..2799613

>lcl|NC_000913.3_gene_2742_gene=ygaZ_locus_tag=b2682_location=2809617..2810354

>lcl|NC_000913.3_gene_2743_gene=ygaH_locus_tag=b2683_location=2810344..2810679

>lcl|NC_000913.3_gene_2747_gene=luxS_locus_tag=b2687_location=complement(2814218..2814733)

>lcl|NC_000913.3_gene_2759_gene=recX_locus_tag=b2698_location=complement(2822139..2822639)

>lcl|NC_000913.3_gene_2789_gene=hypC_locus_tag=b2728_location=2851864..2852136

>lcl|NC_000913.3_gene_2807_gene=ispF_locus_tag=b2746_location=complement(2871301..2871780)

>lcl|NC_000913.3_gene_2809_gene=ftsB_locus_tag=b2748_location=complement(2872509..2872820)

>lcl|NC_000913.3_gene_2810_gene=ygbE_locus_tag=b2749_location=complement(2873014..2873337)

>lcl|NC_000913.3_gene_2811_gene=cysC_locus_tag=b2750_location=complement(2873387..2873992)

>lcl|NC_000913.3_gene_2859_gene=sdaC_locus_tag=b2796_location=2928229..2929518

>lcl|NC_000913.3_gene_2863_gene=fucA_locus_tag=b2800_location=complement(2933041..2933688)

>lcl|NC_000913.3_gene_2867_gene=fucU_locus_tag=b2804_location=2938888..2939310

>lcl|NC_000913.3_gene_2895_gene=mutH_locus_tag=b2831_location=2969662..2970351

>lcl|NC_000913.3_gene_2896_gene=ygdQ_locus_tag=b2832_location=2970420..2971133

>lcl|NC_000913.3_gene_2959_gene=fldB_locus_tag=b2895_location=3039855..3040376

>lcl|NC_000913.3_gene_3015_gene=yggS_locus_tag=b2951_location=3095098..3095802

>lcl|NC_000913.3_gene_3016_gene=yggT_locus_tag=b2952_location=3095820..3096386

>lcl|NC_000913.3_gene_3018_gene=rdgB_locus_tag=b2954_location=3096681..3097274

>lcl|NC_000913.3_gene_3024_gene=trmI_locus_tag=b2960_location=complement(3102133..3102852)

>lcl|NC_000913.3_gene_3060_gene=hybA_locus_tag=b2996_location=complement(3144154..3145140)

>lcl|NC_000913.3_gene_3062_gene=yghW_locus_tag=b2998_location=complement(3146450..3146737)

>lcl|NC_000913.3_gene_3067_gene=exbD_locus_tag=b3005_location=complement(3150818..3151243)

>lcl|NC_000913.3_gene_3070_gene=yghB_locus_tag=b3009_location=3153563..3154222

>lcl|NC_000913.3_gene_3072_gene=yqhD_locus_tag=b3011_location=3155355..3156518

>lcl|NC_000913.3_gene_3075_gene=yqhH_locus_tag=b3014_location=3158627..3158884

>lcl|NC_000913.3_gene_3077_gene=ftsP_locus_tag=b3017_location=complement(3161257..3162669)

>lcl|NC_000913.3_gene_3078_gene=plsC_locus_tag=b3018_location=complement(3162744..3163481)

>lcl|NC_000913.3_gene_3091_gene=yqiA_locus_tag=b3031_location=complement(3175425..3176006)

>lcl|NC_000913.3_gene_3094_gene=nudF_locus_tag=b3034_location=complement(3177281..3177910)

>lcl|NC_000913.3_gene_3100_gene=ribB_locus_tag=b3041_location=complement(3183813..3184466)

>lcl|NC_000913.3_gene_3122_gene=plsY_locus_tag=b3059_location=3204694..3205311

>lcl|NC_000913.3_gene_3138_gene=ebgR_locus_tag=b3075_location=3221466..3222449

>lcl|NC_000913.3_gene_3158_gene=yqjA_locus_tag=b3095_location=3247773..3248435

>lcl|NC_000913.3_gene_3161_gene=yqjD_locus_tag=b3098_location=3249375..3249680

>lcl|NC_000913.3_gene_3169_gene=yhaK_locus_tag=b3106_location=3254319..3255020

>lcl|NC_000913.3_gene_3224_gene=nlpI_locus_tag=b3163_location=complement(3308040..3308924)

>lcl|NC_000913.3_gene_3228_gene=truB_locus_tag=b3166_location=complement(3311833..3312777)

>lcl|NC_000913.3_gene_3241_gene=rlmE_locus_tag=b3179_location=complement(3327035..3327664)

>lcl|NC_000913.3_gene_3246_gene=yhbE_locus_tag=b3184_location=complement(3331770..3332735)

>lcl|NC_000913.3_gene_3249_gene=ispB_locus_tag=b3187_location=3333710..3334681

>lcl|NC_000913.3_gene_3250_gene=sfsB_locus_tag=b3188_location=3334909..3335187

>lcl|NC_000913.3_gene_3251_gene=murA_locus_tag=b3189_location=complement(3335235..3336494)

>lcl|NC_000913.3_gene_3256_gene=mlaE_locus_tag=b3194_location=complement(3338466..3339248)

>lcl|NC_000913.3_gene_3257_gene=mlaF_locus_tag=b3195_location=complement(3339256..3340065)

>lcl|NC_000913.3_gene_3261_gene=lptC_locus_tag=b3199_location=3342836..3343411

>lcl|NC_000913.3_gene_3263_gene=lptB_locus_tag=b3201_location=3343944..3344669

>lcl|NC_000913.3_gene_3267_gene=yhbJ_locus_tag=b3205_location=3347115..3347969

>lcl|NC_000913.3_gene_3268_gene=npr_locus_tag=b3206_location=3347966..3348238

>lcl|NC_000913.3_gene_3292_gene=sspA_locus_tag=b3229_location=complement(3376782..3377420)

>lcl|NC_000913.3_gene_3300_gene=argR_locus_tag=b3237_location=3384703..3385173

>lcl|NC_000913.3_gene_3302_gene=yhcO_locus_tag=b3239_location=complement(3385857..3386129)

>lcl|NC_000913.3_gene_3310_gene=yhdE_locus_tag=b3248_location=complement(3397785..3398378)

>lcl|NC_000913.3_gene_3311_gene=mreD_locus_tag=b3249_location=complement(3398387..3398875)

>lcl|NC_000913.3_gene_3315_gene=acuI_locus_tag=b3253_location=3403484..3404458

>lcl|NC_000913.3_gene_3318_gene=accB_locus_tag=b3255_location=3405436..3405906

>lcl|NC_000913.3_gene_3322_gene=prmA_locus_tag=b3259_location=3409070..3409951

>lcl|NC_000913.3_gene_3349_gene=def_locus_tag=b3287_location=3433690..3434199

>lcl|NC_000913.3_gene_3352_gene=trkA_locus_tag=b3290_location=3436518..3437894

>lcl|NC_000913.3_gene_3358_gene=rpoA_locus_tag=b3295_location=complement(3440040..3441029)

>lcl|NC_000913.3_gene_3363_gene=secY_locus_tag=b3300_location=complement(3442766..3444097)

>lcl|NC_000913.3_gene_3364_gene=rplO_locus_tag=b3301_location=complement(3444105..3444539)

>lcl|NC_000913.3_gene_3366_gene=rpsE_locus_tag=b3303_location=complement(3444726..3445229)

>lcl|NC_000913.3_gene_3368_gene=rplF_locus_tag=b3305_location=complement(3445607..3446140)

>lcl|NC_000913.3_gene_3372_gene=rplX_locus_tag=b3309_location=complement(3447453..3447767)

>lcl|NC_000913.3_gene_3383_gene=rplC_locus_tag=b3320_location=complement(3452297..3452926)

>lcl|NC_000913.3_gene_3416_gene=yheS_locus_tag=b3352_location=3481289..3483202

>lcl|NC_000913.3_gene_3421_gene=crp_locus_tag=b3357_location=3486120..3486752

>lcl|NC_000913.3_gene_3449_gene=rpe_locus_tag=b3386_location=complement(3514382..3515059)

>lcl|NC_000913.3_gene_3450_gene=dam_locus_tag=b3387_location=complement(3515077..3515913)

>lcl|NC_000913.3_gene_3452_gene=aroB_locus_tag=b3389_location=complement(3517398..3518486)

>lcl|NC_000913.3_gene_3460_gene=nudE_locus_tag=b3397_location=complement(3525589..3526149)

>lcl|NC_000913.3_gene_3463_gene=hslR_locus_tag=b3400_location=3529348..3529749

>lcl|NC_000913.3_gene_3468_gene=ompR_locus_tag=b3405_location=complement(3535865..3536584)

>lcl|NC_000913.3_gene_3491_gene=glgA_locus_tag=b3429_location=complement(3566600..3568033)

>lcl|NC_000913.3_gene_3493_gene=glgX_locus_tag=b3431_location=complement(3569346..3571319)

>lcl|NC_000913.3_gene_3510_gene=yhhA_locus_tag=b3448_location=3586943..3587383

>lcl|NC_000913.3_gene_3521_gene=panM_locus_tag=b3459_location=3597984..3598367

>lcl|NC_000913.3_gene_3527_gene=rsmD_locus_tag=b3465_location=3604393..3604989

>lcl|NC_000913.3_gene_3537_gene=acpT_locus_tag=b3475_location=3612969..3613556

>lcl|NC_000913.3_gene_3556_gene=uspB_locus_tag=b3494_location=complement(3639385..3639720)

>lcl|NC_000913.3_gene_3557_gene=uspA_locus_tag=b3495_location=3640111..3640545

>lcl|NC_000913.3_gene_3572_gene=dctR_locus_tag=b3507_location=3654683..3655213

>lcl|NC_000913.3_gene_3575_gene=hdeA_locus_tag=b3510_location=complement(3656408..3656740)

>lcl|NC_000913.3_gene_3583_gene=gadX_locus_tag=b3516_location=complement(3664986..3665810)

>lcl|NC_000913.3_gene_3595_gene=dctA_locus_tag=b3528_location=complement(3682161..3683447)

>lcl|NC_000913.3_gene_3729_gene=rpoZ_locus_tag=b3649_location=3822106..3822381

>lcl|NC_000913.3_gene_3751_gene=uhpA_locus_tag=b3669_location=complement(3850136..3850726)

>lcl|NC_000913.3_gene_3759_gene=yidG_locus_tag=b3675_location=complement(3855608..3855970)

>lcl|NC_000913.3_gene_3814_gene=glmU_locus_tag=b3730_location=complement(3913830..3915200)

>lcl|NC_000913.3_gene_3816_gene=atpD_locus_tag=b3732_location=complement(3915993..3917375)

>lcl|NC_000913.3_gene_3817_gene=atpG_locus_tag=b3733_location=complement(3917402..3918265)

>lcl|NC_000913.3_gene_3826_gene=mioC_locus_tag=b3742_location=complement(3926012..3926455)

>lcl|NC_000913.3_gene_3827_gene=asnC_locus_tag=b3743_location=complement(3926545..3927003)

>lcl|NC_000913.3_gene_3847_gene=yifE_locus_tag=b3764_location=3948086..3948424

>lcl|NC_000913.3_gene_3858_gene=ppiC_locus_tag=b3775_location=complement(3959532..3959813)

>lcl|NC_000913.3_gene_3865_gene=wecA_locus_tag=b3784_location=3967916..3969019

>lcl|NC_000913.3_gene_3872_gene=wecE_locus_tag=b3791_location=3975146..3976276

>lcl|NC_000913.3_gene_3887_gene=hemD_locus_tag=b3804_location=complement(3989088..3989828)

>lcl|NC_000913.3_gene_3890_gene=cyaY_locus_tag=b3807_location=complement(3993739..3994059)

>lcl|NC_000913.3_gene_3915_gene=ubiE_locus_tag=b3833_location=4018855..4019610

>lcl|NC_000913.3_gene_3918_gene=ubiB_locus_tag=b3835_location=4020226..4021866

>lcl|NC_000913.3_gene_3931_gene=hemG_locus_tag=b3850_location=4034608..4035153

>lcl|NC_000913.3_gene_3970_gene=dtd_locus_tag=b3887_location=4077015..4077452

>lcl|NC_000913.3_gene_3974_gene=fdhE_locus_tag=b3891_location=complement(4080299..4081228)

>lcl|NC_000913.3_gene_4002_gene=yiiQ_locus_tag=b3920_location=complement(4111615..4112214)

>lcl|NC_000913.3_gene_4005_gene=uspD_locus_tag=b3923_location=4113293..4113721

>lcl|NC_000913.3_gene_4006_gene=fpr_locus_tag=b3924_location=complement(4113726..4114472)

>lcl|NC_000913.3_gene_4046_gene=yijD_locus_tag=b3964_location=4161771..4162130

>lcl|NC_000913.3_gene_4062_gene=secE_locus_tag=b3981_location=4177358..4177741

>lcl|NC_000913.3_gene_4063_gene=nusG_locus_tag=b3982_location=4177743..4178288

>lcl|NC_000913.3_gene_4078_gene=rsd_locus_tag=b3995_location=complement(4196332..4196808)

>lcl|NC_000913.3_gene_4082_gene=yjaG_locus_tag=b3999_location=4199504..4200094

>lcl|NC_000913.3_gene_4126_gene=dgkA_locus_tag=b4042_location=4256637..4257005

>lcl|NC_000913.3_gene_4127_gene=lexA_locus_tag=b4043_location=4257115..4257723

>lcl|NC_000913.3_gene_4141_gene=yjbQ_locus_tag=b4056_location=4270238..4270654

>lcl|NC_000913.3_gene_4142_gene=yjbR_locus_tag=b4057_location=4270658..4271014

>lcl|NC_000913.3_gene_4203_gene=adiY_locus_tag=b4116_location=complement(4337168..4337929)

>lcl|NC_000913.3_gene_4227_gene=dcuA_locus_tag=b4138_location=complement(4365472..4366773)

>lcl|NC_000913.3_gene_4240_gene=blc_locus_tag=b4149_location=complement(4377189..4377722)

>lcl|NC_000913.3_gene_4243_gene=frdC_locus_tag=b4152_location=complement(4379377..4379772)

>lcl|NC_000913.3_gene_4260_gene=tsaE_locus_tag=b4168_location=4395585..4396046

>lcl|NC_000913.3_gene_4264_gene=hfq_locus_tag=b4172_location=4400288..4400596

>lcl|NC_000913.3_gene_4266_gene=hflK_locus_tag=b4174_location=4402038..4403297

>lcl|NC_000913.3_gene_4267_gene=hflC_locus_tag=b4175_location=4403300..4404304

>lcl|NC_000913.3_gene_4270_gene=nsrR_locus_tag=b4178_location=4406190..4406615

>lcl|NC_000913.3_gene_4314_gene=ytfP_locus_tag=b4222_location=4447894..4448235

>lcl|NC_000913.3_gene_4317_gene=ppa_locus_tag=b4226_location=complement(4449122..4449652)

>lcl|NC_000913.3_gene_4336_gene=pyrB_locus_tag=b4245_location=complement(4471460..4472395)

>lcl|NC_000913.3_gene_4344_gene=rraB_locus_tag=b4255_location=4478473..4478889

>lcl|NC_000913.3_gene_4348_gene=holC_locus_tag=b4259_location=complement(4483837..4484280)

>lcl|NC_000913.3_gene_4454_gene=dnaC_locus_tag=b4361_location=complement(4600238..4600975)

>lcl|NC_000913.3_gene_4467_gene=rimI_locus_tag=b4373_location=4608185..4608631

>lcl|NC_000913.3_gene_4491_gene=rob_locus_tag=b4396_location=complement(4634441..4635310)

>lcl|NC_000913.3_gene_4492_gene=creA_locus_tag=b4397_location=4635521..4635994

>lcl|NC_000913.3_gene_4496_gene=arcA_locus_tag=b4401_location=complement(4639590..4640306)
