# Supplementary material for: Comparative genomic analysis of a Shiga toxin-producing Escherichia coli (STEC) O145:H25 associated with a severe pediatric case of hemolytic uremic syndrome in Davidson County, Tennessee, US
Source: BMC Genomics. 2020 Aug 17;21:564. doi: 10.1186/s12864-020-06967-3 (PMC7437938; doi:10.1186/s12864-020-06967-3)
Supplement: Supplementary file 5 — Additional file 5. Concatenated nucleotide sequences of 341 orthologous CDSs from 34 bacterial strains. [file 12864_2020_6967_MOESM5_ESM.docx]

**Additional file 5.** Concatenated nucleotide sequences of 341 orthologous CDSs from 34 bacterial strains.

>CP006027.fa.conservedCDS.fa

atggttaaagtttatgccccggcttccagtgccaatatgagcgtcgggtttgatgtgctc

ggggcggcggtgacacccgttgatggtgcattgctcggagatgtagtcacggttgaggcg

gcagagacattcagtctcaacaacctaggacgctttgccgataagctgccgtcagaacca

cgggaaaatatcgtttatcagtgctgggagcgtttttgccaggagcttgggaagcaaatt

ccagtggcgatgactctggaaaagaatatgccgatcggttcgggcttaggctccagcgcc

tgttcggtggtcgcggcgctgatggcgatgaatgaacactgtggcaagccgcttaatgac

actcgtttgctggctttgatgggcgagctggaaggacgaatctccggcagcatacattac

gacaacgtggcaccgtgttttcttggtggtatgcagttgatgatcgaagaaaacgacatc

atcagccagcaagtgccagggtttgatgagtggctgtgggtgctggcgtatccggggatt

aaagtctcgacggcagaagcccgggctattttaccggcgcagtatcgccgccaggattgc

attgcgcacgggcgacatctggcaggcttcattcacgcctgctattcccgtcagcctgag

cttgccgcgaagctgatgaaagatgttatcgcagaaccctaccgtgaacggttactgcct

ggcttccggcaggcgcggcaggcggtcgcggaaatcggcgcggtagcgagcggtatctcc

ggctccggcccgaccttgttcgcgctgtgtgacaagccggataccgcccagcgcgttgcc

gactggttgggtaagaactacctgcaaaatcaggaaggttttgttcatatttgccggctg

gatacggcgggcgcacgagtactggaaaactaaatgggcaacactaagttggctaatccg

gcaccgctgggcctgatgggcttcggcatgaccaccattctgcttaacctgcacaacgtg

ggttatttcgctctggacggtattattcttgccatgggcattttctacggcggcatcgcg

caaatttttgccggtctgctggagtacaaaaaaggcaacactttcggtttaaccgcattc

acctcttacggttctttctggctgacgctggttgcgattctgctgatgccgaaactgggt

ctgaccgatgcgccaaatgcacagttccttggtgtctacctgggtctgtggggcgtattt

acgctgtttatgttcttcggcacgctgaaaggcgcacgcgttctgcaattcgttttcttt

agcctgaccgtgctgtttgccctgctggcgatcggtaacattgccggtaacgccgcaatc

atccactttgccggctggattggtctgatctgcggtgccagcgcaatctatctggcgatg

ggtgaagtactgaacgagcagtttggtcgcaccgttctgccgattggtgaatcccactaa

atgggtaaaataattggtatcgacctgggtactaccaactcttgtgtagcgattatggat

ggcaccactcctcgcgtgctggagaacgccgaaggcgatcgcaccacgccttctattatt

gcctatacccaggatggtgaaactctggttggtcagccggctaaacgtcaggcagtgacg

aacccgcaaaacaccctgtttgcgattaaacgcctgattggccgccgcttccaggacgaa

gaagtacagcgtgatgtttccatcatgccgttcaaaattattgctgctgataacggcgac

gcatgggtcgaagttaaaggccagaaaatggcaccgccgcagatttctgctgaagtgctg

aaaaaaatgaagaaaaccgctgaagattacctgggtgaaccggtaactgaagctgttatc

accgtaccggcatactttaacgatgctcagcgtcaggcaaccaaagacgcaggccgtatc

gctggtctggaagtaaaacgtatcatcaacgaaccgaccgcagctgcgctggcttacggt

ctggacaaaggtactggcaaccgtactatcgcggtttatgacctgggtggtggtactttc

gatatttctattatcgaaatcgacgaagttgacggcgaaaaaaccttcgaagttctggca

accaacggtgatacccacctgggtggtgaagacttcgacagccgtctgatcaactatctg

gttgaagaattcaagaaagatcagggcattgacctgcgcaacgatccgctggcaatgcag

cgcctgaaagaagcggcagaaaaagcgaaaatcgaactgtcttccgctcagcagaccgac

gttaacctgccgtacatcactgcagatgcgaccggtccgaaacacatgaacatcaaagtg

actcgtgcgaaactggaaagcctggttgaagatctggtaaaccgttccattgagccgctg

aaagttgcactgcaggacgctggcctgtccgtatctgatatcgacgacgttatcctcgtt

ggtggtcagactcgcatgccaatggttcagaagaaagttgctgagttctttggtaaagag

ccgcgtaaagacgttaacccggacgaagctgtagcaatcggtgctgctgttcagggtggt

gttctgactggtgacgtaaaagacgtactgctgctggacgttaccccgctgtctctgggt

atcgaaaccatgggcggtgtgatgacgacgctgatcgcgaaaaacaccactatcccgacc

aagcacagccaggtgttctctaccgctgaagacaaccagtctgcggtaaccatccatgtg

ctgcagggtgaacgtaaacgtgcggctgataacaaatctctgggtcagttcaacttggat

ggtatcaacccggcaccgcgcggcatgccgcagatcgaagttaccttcgatatcgatgct

gacggtatcctgcacgtttccgcgaaagataaaaacagcggtaaagagcagaagatcacc

atcaaagcttcttctggtctgaacgaagatgaaatccagaaaatggtacgcgacgcagaa

gctaacgccgaagctgaccgtaagtttgaagagctggtacagactcgcaaccagggcgac

catctgctgcacagcacccgtaagcaggttgaagaagcaggcgacaaactgccggctgac

gacaaaactgctatcgagtctgcactgactgcactggaaactgctctgaaaggtgaagac

aaagccgctatcgaagcgaaaatgcaggagctggcacaggtttcccagaaactgatggaa

atcgcccagcagcaacatgcccagcagcagactgccggtgctgatgcttctgcaaacaac

gcgaaagatgacgatgttgtcgacgctgaatttgaagaagtcaaagacaaaaaataaatg

agtcaatcgatctgttcaacagggctacgctggctgtggctggtggtagtcgtgctgatt

atcgatctgggcagcaaatacctgatcctccagaactttgctctgggggatacggtcccg

ctgttcccgtcgcttaatctgcattatgcgcgtaactatggcgcggcgtttagtttcctt

gccgatagcggcggctggcagcgttggttctttgccggtattgcgattggtattagcgtg

atcctggcagtgatgatgtatcgctcgaaggccacgcagaagctaaacaatatcgcttat

gcgctgattattggcggcgcgctgggcaacctgttcgaccgcctgtggcacggcttcgtt

gtcgatatgatcgacttctacgtcggcgactggcacttcgccaccttcaaccttgccgat

actgccatttgtgtcggtgcggcactgattgtgctggaaggttttttgccttctaaagcg

aaaaaacaataaatgtctgaatctgtacagagcaatagcgccgtcctggtgcacttcacg

ctaaaactcgacgatggcaccaccgccgagtctacccgcaacaacggtaaaccggcgctg

ttccgcctgggcgatgcttctctttctgaagggctggagcaacacctgctggggctgaaa

gtgggcgataaaaccaccttctcactggagcccgatgcggcgtttggcgtgccgtcaccg

gacctgattcagtacttctcccgccgtgaatttatggatgcaggcgagccagaaattggc

gcaatcatgctttttaccgcaatggatggcagtgagatgcctggtgtgatccgcgaaatt

aacggcgactccattaccgttgatttcaaccatccgctggccgggcagaccgttcatttt

gatattgaagtgctggaaatcgatccggcactggaggcgtaaatgatcctcataatttat

gcgcatccgtatccgcatcattcccatgcgaataaacggatgcttgaacaggcaaggacg

ctggaaggcgtcgaaattcgctctctttatcaactctatcctgacttcaatatcgatatt

gccgccgagcaggaggcgctgtctcgcgccgatctgatcgtctggcagcacccgatgcag

tggtacagcattcctccgcttcttaaactttggatcgataaagttttctcgcacggctgg

gcttacggtcacggcggcacggcgctgcatggcaaacatttgctgtgggcggtgacgacc

ggcggcggggaaagccattttgaaattggtgcgcatccgggctttgatgtgctgtcgcag

ccgctacaggcgacggcaatctactgcgggctgaactggctgcctccgtttgccatgcac

tgcacctttatttgtgacgacgaaaccctcgaagggcaggcgcgccactataagcaacgt

ctgctggaatggcaggaggcacatcatggatagatgaataatcgagtccaccagggccac

ttagcccgtaaacgcttcgggcaaaactttctcaacgatcagttcgtgatcgacagcatt

gtttctgccattaacccgcagaagggtcaggcgatggtcgaaatcggccccggtctggct

gcattgaccgaaccggtcggcgaacgtctggaccaactgacggttatcgaacttgaccgc

gatctggcggcacgtctgcaaacgcatccattcttaggcccgaaactgacgatttatcag

caggatgcgatgacctttaactttggcgaactggccgagaaaatgggtcagccgctgcgt

gttttcggcaacctgccttataacatctccacgccgttgatgttccatctgtttagctat

actgatgccattgccgacatgcactttatgttgcaaaaagaggtggtgaatcgtctggtt

gcaggaccgaacagcaaggcgtatggtcgattaagcgtcatggcgcaatactattgcaac

gtgatcccagtgctggaagtacctccgtcagcctttacaccaccacccaaagtggattcc

gccgtcgtgcgcctggttcctcatgcaacgatgcctcacccggttaaagatgttcgcgtg

ttgagccgcatcaccaccgaagcctttaaccagcgtcgtaaaaccattcgtaacagcctc

ggcaacctgtttagcgtcgaggtgttaacgggaatggggatcgacccggcgatgcgagcg

gaaaatatctctgtcgcgcaatattgccagatggcgaactatctggcggagaacgcgcct

ttgcaggagagttaagtgaaactggatgaaatcgctcggctggcgggagtgtcgcggacc

actgcaagctatgttattaacggcaaagcgaagcaataccgtgtgagcgacaaaaccgtt

gaaaaagtcatggctgtggtgcgtgagcacaattaccacccgaacgccgtggcagctggg

cttcgtgctggacgcacacgttctattggtcttgtgatccccgatctggagaacaccagc

tatacccgcatcgctaactatcttgaacgccaggcgcggcaacggggttatcaactgctg

attgcctgctcagaagatcagccagacaacgaaatgcgctgcattgagcaccttttacag

cgtcaggttgatgccattattgtttcgacgtcattgccacctgagcatcctttttatcaa

cgctgggctaacgacccgttcccgattgtcgcgctggaccgcgccctcgatcgtgaacac

tttaccagcgtggttggtgccgatcaggatgatgccgaaatgctggcggaagagttacgt

aagtttcccgccgagacggtgctttatcttggcgcgctgccggagctttctgtcagcttc

ctgcgtgaacaaggtttccgtactgcctggaaagatgatccgcgcgaagtgcatttcctg

tatgccaacagctatgagcgggaggcggctgcccagttattcgaaaaatggctggaaacg

catccgatgccgcaggcgctgttcacaacgtcgtttgcgttgttgcaaggagtaatggat

gtcacgctgcgtcgcgacggcaaactgccttctgacctggcaattgccacctttggcgat

aatgaactgctcgacttcttacagtgcccggtgctggcagtggctcaacgtcaccgcgat

gtcgcagagcgtgtgctggagattgtcctggcaagcctggacgaaccgcgtaagccaaaa

cctggtttaacgcgcattaaacgtaatctctatcgccgcggtgtgctcaaccgtagctaa

atgatcagcagagtgacagaagctctaagcaaagttaaaggatcgatgggaagccacgag

cgccatgcattgcctggtgttattggtgacgatcttttgcgatttgggaagctgccactc

tgcctgttcatttgcattattttgacggcggtgaccgtggtaaccacggcgcaccatacc

cgtttactgaccgctcagcgcgaacaactggtgctggagcgagatgctttagacattgaa

tggcgcaacctgatccttgaagagaatgcgctcggcgaccatagccgggtggaaaggatc

gccacggaaaagctgcaaatgcagcatgttgatccgtcacaagaaaatatcgtagtgcaa

aaataaatgagtggtcaaggaaagcgattaatggtgatggcaggcggaaccggtggacat

gtattcccgggactggcggttgcgcaccatctaatggctcagggttggcaagttcgctgg

ctggggactgccgaccgtatggaagcggacttagtgccaaaacatggcatcgaaattgat

ttcattcgtatctctggtctgcgtggaaaaggtataaaagcactgatagctgcgccgctg

cgtatcttcaatgcctggcgtcaggcgcgggcgattatgaaagcgtacaaacctgacgtg

gtgctcggtatgggcggctacgtatcaggtcctggtggtctggccgcgtggtcgttaggc

attccggttgtacttcatgaacaaaacggtattgcgggcttaaccaataaatggctggcg

aagattgccaccaaagtgatgcaggcgtttccaggtgctttccccaatgcggaagtggtg

ggtaacccggtgcgtaccgatgtgttggcgctaccgttgccgcagcaacgtttggctgga

cgtgaaggtccggttcgtgtgctggtagtgggtggttcccagggcgcacgcattcttaac

cagacaatgccgcaggttgctgcgaaactgggtgattcagtcactatctggcatcagagc

ggcaaaggttcgcaacaatccgttgaacaggcgtatgccgaagcggggcaaccgcagcat

aaagtgactgaatttattgatgatatggcggcggcgtatgcgtgggcggatgtcgtcgtt

tgccgctccggtgctttaacggtgagtgaaatcgccgcggcaggactaccggcgttgttt

gtgccgtttcaacataaagaccgccagcaatactggaatgcgctaccgctggaaaaagcg

ggcgcagccaaaattatcgagcagccacagcttagcgtggatgctgtcgccaacaccctg

gccgggtggtcgcgagaaaccttattaaccatggcagaacgcgcccgcgctgcatccatt

ccggatgccaccgagcgagtggcaaatgaagtgagccgggctgcccgggcgtaaatgtcg

caggctgctctgaacacgcgaaacagcgaagaagaggtttcttctcgccgcaataatgga

acgcgtctggcggggatccttttcctgctgaccgttttaacgacagtgttggtgagcggc

tgggtcgtgttgggctggatggaagatgcgcaacgcctgccgctctcaaagctggtgttg

accggtgaacgccattacacgcgtaatgacgatatccggcagtcgatcctggcattgggt

gagccgggtacctttatgacccaggatgtcaacatcatccagacgcaaatagaacaacgc

ctgccgtggattaagcaggtgagcgtcagaaagcagtggcctgatgaattgaagattcat

ctggttgaatatgtgccgattgcgcggtggaatgatcaacacatggtagacgcggaagga

aataccttcagcgtgccgccagatcgcaccagcaagcaggtgcttccaatgctgtatggc

ccggaaggcagcgccaatgaagtgttgcagggctatcgcgaaatggggcagatgctggca

aaggacagatttactctgaaggaagcggcgatgaccgcgcggcgttcctggcagttgacg

ctgaataacgatattaagctcaatcttggccggggcgatacgatgaaacgtttggctcgc

tttgtagaactttatccggttttacagcagcaggcgcaaaccgatggcaaacggattagc

tacgttgatttgcgttatgactctggagcggcagtaggctgggcgcccttgccgccagag

gaatctactcagcaacaaaatcaggcacaggcagaacaacaatgaatgatcaaacaaagg

acacttaaacgtatcgttcaggcgacgggtgtcggtttacataccggcaagaaagtcacc

ctgacgttacgccctgcgccggccaacaccggggtcatctatcgtcgcaccgacttgaat

ccaccggtagatttcccggccgatgccaaatctgtgcgtgataccatgctctgtacgtgt

ctggtcaacgagcatgatgtacggatttcaaccgtagagcacctcaatgctgctctcgcg

ggcctgggcatcgataacattgttatcgaagttaacgcgccggaaatcccgatcatggac

ggcagcgccgctccgtttgtatacctgctgcttgacgccggtatcgacgagttgaactgc

gccaagaaatttgttcgcatcaaagagactgttcgtgtcgaagatggcgataagtgggct

gaatttaagccgtacaatggtttttcgctggatttcaccatcgattttaaccatccggct

attgattccagcaaccagcgctatgcgatgaacttctccgctgatgcgtttatgcgccag

atcagccgtgcgcgtacgttcggtttcatgcgtgatatcgaatatctgcagtcccgtggt

ttgtgcctgggcggcagcttcgattgtgccatcgttgttgacgattatcgcgtactgaac

gaagacggcctgcgttttgaagacgaatttgtgcgtcacaaaatgcttgatgcgatcggt

gacttgttcatgtgtggtcacaatattattggtgcatttaccgcttataaatccggtcat

gcactgaataacaaactgctgcaggctgtcctggcgaaacaggaagcctgggaatatgtg

accttccaggacgacgcagaactgccgttggccttcaaagcgccttcagccgtactggca

taaatgcagacccaggtcctttttgaacatccactaaatgaaaaaatgcgtacatggctg

cgcattgagtttttgactcagcaactcaccgttaatttacccatcgttgaccacactggc

gcgctgcatttcttccgtaatgtcagtgaattactggatgttttcgagcgcggcgaagtc

cgcactgagctgttgaaagaacttgaccggcagcaacgtaaactccagacctggattggc

gtgcctggcgtggaccagagccgtattgaagcattaattcagcagttaaaagcggcgggg

agcgtattaatttccgcgccgcgtatcgggcaatttctgcgtgaagatcgtttgattgct

ctggtgcgtcagcgactgagcatcccaggcggctgttgcagctttgatttacctacattg

cacatttggctgcatctaccccaggcgcagcgcgacagccaggtagaaacctggattgcc

agcctgaacccgctcacccaggcgcttaccatggtgctggatttaattcgccagtcggcc

cccttccgtaaacaaaccagcctgaatggtttttatcaggataacggtggcgatgccgac

ttgctgcgcctgaatctgtcgctcgattcacagctttatccgcaaatttccggtcataag

agccgttttgccattcgttttatgccgctggacagtgaaaacggacaggtaccggaacgt

ctggatttcgaactggcctgttgctaaatgaggtatatagttgccttaacgggaggcatt

ggcagtggcaagagtaccgttgccaatgcgtttgctgatctcggaattaacgtcattgat

gccgatattattgcgcgtcaggtggttgaaccaggtgcacctgcgctacatgccattgct

gatcactttggcgctaacatgattgctgctgatggaacattgcagcgccgggccttgcgc

gagcggatcttcgccaacccggaagagaaaaactggcttaacgacctgctgcatccgctg

attcagcaagagacgcaacaccagatccagcaagcaacctccccctatgtactgtgggtt

gtgccattgctggtagaaaactcactgtataaaaaagcgaatcgagtgctggtggtggat

gtcagcccagaaacgcaacttaagcgcaccatgcagcgcgatgatgtaactcgcgagcat

gtcgaacaaatccttgctgctcaggcaacgcgcgaagcccgccttgccgtggcagatgac

gtcattgataataacggcgcaccggatgctatcgcatcggatgttgcccgcctgcacgca

cactatttgcagcttgcgtcgcagtttgtctcacaggaaaaaccgtaaatgttgttagaa

caggggtggctggttggcgcgcgccgcgttccctcaccacattacgattgccgcccggat

gacgaaacacccaccctgctggtggtgcacaatattagcctaccgccaggcgagtttggc

ggtccgtggatcgacgcattattcactggaactattgatccgcaggcacatcctttcttt

gctgagatcgcccatttgcgcgtctccgctcactgtttgattcgccgtgatggtgaaata

gtccagtatgttcctttcgataaacgtgcatggcatgcgggagtctctcagtatcagggg

cgcgaacgctgcaatgatttttctattgggattgagcttgaaggcaccgatacgctggcg

tataccgatgcgcagtatcaacagcttgcggcggttacgcgggcactgattgattgctat

ccggatatcgctaataacatgacgggccattgtgatattgcgccggatcggaaaaccgat

cccggtcctgcatttgattgggcacggtttcgtgcgctggtcagcaaggagacaacatga

atgacgctatttacaaccttactggtgttaattttcgagcgcctgtttaagttgggcgag

cactggcagcttgatcatcgtcttgaagcgttctttcggcgggtgaaacatttttctctc

gggcgcacgttaggcatgaccattattgcgatgggcgtgacttttttactgttacgcgca

ttgcagggagtattgttcaacgttcccacgctgctggtgtggctgctgattggtttgctg

tgtattggcgcaggtaaagttcgtcttcattatcatgcttatctgacagctgcttcacgt

aatgatagccatgcccgtgccacgatggctggcgaactcaccatgattcacggcgtcccg

gcaggctgcgacgaacgtgagtatttgcgtgagctgcaaaatgcattgctgtggattaac

tttcgtttttatcttgcaccgctgttctggctgattgtggggggaacctggggacccgtt

acgctgatggggtatgcgttcttgcgtgcatggcaatactggctggcacgatatcagacg

ccgcatcatcgtttacagtccggcattgatgccgtgcttcatgtactggattgggtgccg

gttcgtcttgcgggtgtggtatatgccttgatcggtcatggtgagaaagcgttaccggcc

tggtttgcttcgctgggtgatttccatacttcgcagtatcaggtgttaacgcgtctggcg

cagttctctctggcgcgtgaaccgcatgtcgataaggtggagacgccgaaggcagcggtt

tcaatggcgaagaaaacctcgttcgtggtcgtggtggtgattgcgctactgacgatttac

ggggcgttggtgtaaatggccgaaaaaaaacagtggcatgaaacgctacacgaccagttt

gggcagtactttgcggtagataacgttctgtatcatgaaaagaccgatcaccaggatctg

atcatttttgagaacgctgcatttggtcgcgtaatggcgctggatggcgtagtacaaacc

accgagcgcgacgagtttatctatcatgagatgatgacccatgttccgctactggcccac

ggtcacgcgaaacatgtgctgattatcggcggcggcgacggtgccatgctgcgtgaagta

acccgacataaaaacgttgagtcaatcacgatggtggaaatcgatgcgggtgtcgtgtcg

ttctgccgtcagtatctacccaaccataacgccggtagctacgacgatccgcgctttaag

ctggtgatcgacgatggcgtcaatttcgttaatcaaaccagccagacctttgatgtcatt

atctccgactgcaccgatcctatcggtcccggcgaaagccttttcacttcggcattttat

gaaggctgcaaacgttgcctgaatcctggcggtatcttcgtcgcacaaaacggcgtctgc

tttttacagcaggaagaagccatcgacagccatcgcaaactcagccattacttcagcgac

gttggcttttatcaggcggcgatcccgacctattacggcggtatcatgacttttgcatgg

gcgacagataacgacgccttacgccatctctcaaccgaaattattcaggcgcgttttctc

gcctctggcctgaaatgccgttattacaatccggcagtccatacggcagcttttgcctta

ccccagtatctgcaagacgcactggcttcacagccgtcctaaatgaaagacatagataca

ctcatcagcaacaatgcactatggtcaaaaatgctggtggaagaggatcccgggtttttt

gagaaactggcacaagcgcaaaaaccgcgctttctatggattggatgttccgacagtcgc

gttcctgcagaacgtttaaccggtcttgagccgggcgaactctttgttcaccgtaatgtt

gctaacctggtcattcacaccgacctgaactgcctttccgtggttcagtatgcagtggat

gtactcgaagttgaacacattattatctgtggccactacggttgcggcggcgtacaagcc

gcagttgaaaacccggaactggggcttatcaacaactggctgctgcatatccgcgatatc

tggttcaaacatagctcattgctcggcgaaatgccgcaagagcgccgtctggataccttg

tgtgaactgaacgtcatggaacaggtgtataacctgggccactccaccattatgcaatca

gcgtggaaacgcgggcagaaagttaccattcacggctgggcctatggcattcacgacggc

ttgctgcgtgatctggatgttactgccaccaaccgcgaaacccttgagcaacgttaccgt

catgggatttccaacctcaagctgaaacacgccaaccacaaataaatgatgcatctttac

tgggtggcgctaaaaagcatctgggcgaaagagatccatcgctttatgcgtatctgggtg

cagacgctggtgccgccagttatcaccatgaccctttactttattattttcggtaacctg

attggttcgcgtattggcgatatgcatggcttcagctacatgcagttcatcgtaccgggg

ctgatcatgatgtcggtgatcaccaacgcctacgccaacgtcgcgtcatcattttttggt

gccaaattccagcgtaatattgaagagctgctggtagcgccggttccgactcacgtcatt

attgccggatatgtcggcggtggcgtggcgcgtggtctgtttgttggcattctggtgacg

gcaatttcactgttttttgtgccgtttcaggtgcattcgtgggtattcgttgccttaacc

ctggtgctcacggcggtgttgttctcccttgcgggtttgctgaacggcgtgtttgccaaa

acgttcgatgacatcagcctggtgccaacctttgtgttaacgccactcacgtatttgggt

ggggtcttttactcactgactttgttgccgccgttctggcaagggctgtcgcacctgaac

ccaatcgtttatatgatcagtggtttccgctacggcttcctcggtatcaatgatgttccg

ctggtcactacctttggcgtactggtggtctttattgtggcgttttatttgatctgttgg

tcgctgatccaacgtggacgtggtttgcgtagctaaatgattcgcacgatgctgcagggc

aaactccaccgcgtgaaagtgactcatgcggacctgcactatgaaggttcttgcgccatt

gaccaggattttcttgacgcagccggtattctcgaaaacgaagccattgatatctggaat

gtcaccaacggcaagcgtttctccacttatgccatcgcggcagaacgcggttcgagaatt

atttctgttaacggtgcggcggcccactgcgccagtgtcggcgatattgtcatcatcgcc

agcttcgttaccatgccagatgaagaagctcgcacctggcgacccaacgtcgcctatttt

gaaggcgacaatgaaatgaaacgtaccgcgaaagcgattccggtacaggttgcttgaatg

gaattctctccccctctacagcgcgcgacgctaattcagcgttacaaacgttttttagcc

gatgtgatcacacccgatggtcgcgaattaacgctacactgcccgaatacgggtgcgatg

accggttgtgcaacgcctggcgataccgtctggtattcgacttcagacaacaccaaacgg

aaatacccacacacctgggaattaactcaaagccagagcggcgcatttatttgcgtcaac

acgttttgggctaacaggttgacgaaagaggctatccttaatgaatcaatttcagaactg

tcaggctatagctcgctgaaaagcgaagtaaaatacggcgcagaacgcagccgtattgac

tttatgttgcaggcggattcgcgtccagactgctatattgaagtgaaatcggttacgtta

gcggagaacgaacagggatattttcccgatgcggtcactgaacgaggtcagaaacacctt

cgggagttgatgagcgtagcggctgaaggccagcgtgcggttatctttttcgccgtgctg

cattcagccattacacggttttcacccgcgcgccacatcgatgagaaatacgcgcaacta

ttgtcagaagctcaacagaggggggtagaaattctggcttacaaagcggaaatttctgct

gaaggcatggctcttaaaaaatcactgccggttacattgtagatgctcgtctattggctg

gatatagtcggcacagcggtatttgccatctccggcgttttgttagccggaaaattgcgt

atggacccttttggtgttctggtactgggcgtggttaccgctgtaggcggcgggacaatt

cgcgacatggcgctggatcacggcccggtattttgggtgaaagatcccaccgatctggtc

gttgcaatggtaaccagcatgctgaccatcgtgctggtgcgccagccaagacgcttacca

aaatggatgttgccggtgctggacgccgttggtctggcggtgtttgtcggcattggcgtg

aataaagcctttaatgcggaagccggtccgttaatcgcggtttgtatgggcgtcattact

ggcgttggcggcgggatcattcgtgatgttctggcccgcgaaatccccatgattttacgt

acagaaatctacgcaactgcctgtattatcggcggtattgtccacgctacggcttattac

acattttccgtaccactggaaacagccagtatgatgggcatggtcgtgacgctattgatt

cggctggcggctattcgttggcatcttaagctaccgacgtttgcgctggatgagaatggg

cgttgaatgaaaatcggcatcattggtgcaatggaagaagaagttacgctgctgcgtgac

aaaatcgaaaaccgtcaaactatcagtctcggcggttgcgaaatctataccggccaactg

aatggaaccgaggttgcgcttctgaaatcgggcatcggtaaagtcgctgcggcgctgggt

gccactttgctgttggaacactgcaagccagatgtgattattaacaccggttctgccggt

ggcctggcaccaacgctgaaagtgggcgatatcgttgtctcggacgaagcacgttatcac

gacgcggatgtcacggcatttggttatgaatacggtcagttaccaggctgtccggcaggc

tttaaagctgacgataaactgatcgctgccgctgaggcctgcattgccgaactgaatctt

aacgctgtacgtggcctgattgttagcggcgacgctttcatcaacggttctgttggtctg

gcgaaaatccgccacaatttcccacaggccattgctgtagagatggaagcgacggcaatc

gcccatgtctgccacaatttcaacgtcccgtttgtcgtagtacgcgccatctccgacgtg

gccgatcaacagtctcatcttagcttcgatgagttcctggctgttgccgctaaacagtcc

agcctgatggttgagtcactggtgcagaaactggcacatggctaaatgtacgacaatctg

aaaagtctgggtattaccaatcctgaagaaattgatcgttacagcctccggcaggaagcc

aacaacgatattctgaaaatctatttccagaaagacaaaggcgagtttttcgccaagagc

gttaagtttaaatatccgcgtcagcgtaaaacggtcgtcgctgatggtgtgggtcagggt

tataaagaagtccaggaaatcagcccgaatctacggtatatcattgatgagcttgatcaa

atctgccagcgtgaccgcagcgaagttgatcttaagcgtaagatcctcgacgacttacgt

cacctggagtcagtcgtaaccaataagatcagcgagattgaagccgatctggaaaaacta

acgcgtaaataaatgcagcagttacagaacattattgaaaccgcttttgaacgccgtgcc

gagatcacgccagccaatgcagacaccgttacccgcgaagcggtaaatcaggtgatcgcc

ctgctggattccggcgcactgcgtgtagcggaaaaaattgacggtcagtgggtgacgcat

cagtggttgaaaaaagcggtactgctctctttccgtattaatgataatcaggtgatcgaa

ggggcagaaagccgctacttcgataaagtgccgatgaaattcgctgattacgacgaagca

cgtttccagaaagaaggcttccgtgttgtgccaccagcggcggtacgtcagggcgcgttc

attgcccgtaacaccgtgctgatgccgtcttacgtcaacatcggcgcatatgttgatgaa

ggcaccatggttgatacctgggcgaccgtcggttcctgtgcgcagattggtaaaaacgtc

cacctttccggtggcgtgggtatcggcggcgtgctggagccgctgcaggctaacccgacc

attattgaagataactgcttcatcggcgcgcgctctgaagtggttgaaggggtgattgtc

gaagaaggttccgtcatttccatgggcgtatacattggtcagagcacccgtatttacgac

cgtgaaaccggcgaaatccactacggtcgcgttccggcggggtctgtggttgtttcaggt

aatctgccgtcgaaagatggcaaatacagcctctactgtgcggttatcgttaagaaagtt

gacgcgaaaactcgcggcaaagtcggcattaacgaactgctgcgtaccatcgactaaatg

gctaccaatgcaaaacccgtctataaacgcattctgcttaagttgagtggcgaagctctg

cagggcactgaaggcttcggtattgatgcaagcatactggatcgcatggctcaggaaatc

aaagaactggttgaactgggtattcaggttggtgtggtgattggtgggggtaacctgttc

cgtggcgctggtctggcgaaagcgggtatgaaccgcgttgtgggcgaccacatggggatg

ctggcgaccgtaatgaacggcctggcaatgcgtgatgcactgcaccgcgcctatgtgaac

gctcgcctgatgtccgctattccattgaatggcgtgtgcgacagctacagctgggcagaa

gctatcagcctgttgcgcaacaaccgtgtggtgatcctctccgccggtacaggtaacccg

ttctttaccaccgactcagcagcttgcctgcgtggtatcgaaattgaagccgatgtggtg

ctgaaagcaaccaaagttgacggcgtgtttaccgctgatccggcgaaagatccaaccgca

accatgtacgagcaactgacttacagcgaagtgctggaaaaagagctgaaagtcatggac

ctggcggccttcacgctggctcgtgaccataaattaccgattcgtgttttcaacatgaac

aaaccgggtgcgctgcgccgtgtggtaatgggtgaaaaagaagggactttaatcacggaa

taagtgattgataaatccgcctttgtgcatccaaccgccattgtggaagagggcgcgtcg

attggcgcgaacgcacacattggtcctttttgtatcgttggaccccatgtcgaaattggt

gagggtaccgtactgaaatctcacgttgtcgtgaatggtcatactaaaattggccgcgat

aatgagatttatcagttcgcctccatcggcgaagttaaccaggatttgaaatatgctggc

gaaccgacccgtgtggaaatcggcgatcgtaaccgcattcgcgaaagcgtcaccattcat

cgtggcacagtccagggcggtggattgacgaaggtgggcagcgacaacttactgatgatc

aacgcgcacattgcgcacgattgtacggtaggtaaccgctgtattctcgccaacaacgca

acgctggcgggtcacgtatcggttgacgacttcgcgatcatcggcggcatgaccgcagtc

catcagttctgcatcattggtgcgcatgtgatggttggcggctgctccggtgtggcgcag

gacgtccctccttatgtcattgcgcagggtaaccacgccacgccgttcggtgttaatatc

gaagggctgaagcgccgcggattcagccgtgaggcgattaccgctatccgcaatgcgtat

aagctgatttatcgtagcggtaaaacgctcgatgaagtgaaaccggaaattgctgaactg

gcggaaacatatccggaagtgaaagcctttaccgatttctttgcacgctcaacgcgcggt

ctgattcgttaaatgactgaacagcgtccattaacgattgccctggtcgccggagaaacc

tccggcgatatcctgggggccggtttaatccgcgctctgaaagaacgtgtgcccaacgcc

cgctttgttggtgttgccgggccacgaatgcaggctgaaggctgcgaagcctggtacgaa

atggaagaactggcggtgatgggcattgttgaagtgctcggtcgtctgcgtcgcttactg

catattcgtgccgatctgacaaagcgttttggtgaactaaagccagatgtttttgttggc

attgatgcgcctgacttcaatattaccctcgaaggtaaccttaaaaagcagggtatcaaa

accattcattatgtcagtccgtccgtctgggcgtggcgacagaaacgcgttttcaaaata

ggcagagccaccgatctggtgctcgcatttctgcctttcgaaaaagcgttttatgacaaa

tacaacgtaccgtgccgctttatcggtcataccatggctgatgccatgccattagatcca

gataaaaatgccgcccgtgatgtgctggggatcccgcacaatacccactgtctggcattg

ttgccgggaagtcgtggcgcggaagttgagatgcttagcgccgatttcctgaaaacggcc

cagcttttgcgccagacgtaccctgatctggagatcgtggtgccgctggtgaatgccaaa

cgccgcgagcagtttgaacgcatcaaagctgaagtcgcgccagacctttcagttcatttg

ctggatgggatgggccgtgaggcgatggtcgccagcgatgcggcactactggcatcgggg

acggcagccctggagtgtatgctggcgaaatgcccgatggtggtgggatatcgcatgaag

ccttttaccttctggttggcgaagcggctggtgaaaactgattatgtctcgctgccaaat

ctgctggcgggcagagagttagtcaaagagttattgcaggaagagtgtgagccgcaaaaa

ctggctgaggcgctgttaccgctgctggcgaacgggaaaaccagccacgcgatgcacgat

accttccgtgaactgcatcagcagatccgctgcaatgccgatgagcaggcggcacaagcc

gttctggagttagcacaatgaatgcgtgctttaccgatctgtttagtagcactcatgcta

agcggctgttccatgttaagcagatcccctgtcgaacccgttcaaagcactgcaccccag

ccgaaagcggagcctgcaaaaccgaaagcgccgcgcgccacgccggtccgaatttatacc

aatgcagaagaattagtcggcaaaccgttccgcgatctcggtgaagtcagtggcgactct

tgccaggcctctaatcaggactctccgccgagcattccaaccgcacgtaagcggatgcaa

atcaacgcctctaaaatgaaagccaatgctgtattactgcatagctgcgaagtcaccagc

ggtacgccaggctgctatcgtcaggctgtatgtatcggttctgcgcttaacattacggcg

aaatgaatgataaaactttcgaatatcaccaaagtgttccaccagggcacccgcaccatc

caggcgttgaacaacgtcagcctgcatgtgccagctgggcaaatttatggcgttatcggt

gcctcaggcgcgggtaagagtacgcttatacgttgtgtaaacctgctggagcgcccaacc

gagggtagtgtgctggtcgatggccaggaactgaccacgctgtcagaatccgagttgacc

aaagctcgccgccagattggtatgattttccagcattttaacctgctctcttcgcgtact

gtttttggcaacgtggctctgccgctggagctggacaacacaccgaaagacgagatcaaa

cgtcgcgtgacggaattgctgtcattagttggtcttggcgataagcatgatagctacccg

tcgaatctttccggtgggcagaaacaacgtgtggcgattgcccgtgcattagccagcaat

cccaaagtattgctgtgtgatgaagccaccagcgcgctggacccggcaacgacacgttct

attctcgaactgctgaaagacatcaaccgccgtctgggtttgacgattctgttgatcact

cacgaaatggacgttgtgaagcgcatttgtgattgcgtggcggtcatcagcaatggcgaa

ctgatcgagcaggacacggtaagtgaagtgttctcgcatccgaaaacgccgctggcgcag

aagtttattcagtcaaccctgcatctggatatcccggaagattaccaggaacgtctgcaa

gcggagccattcactgactgcgtcccgatgctgcgtctggagtttaccggtcaatcggtc

gatgccccactgctttctgaaaccgcgcgtcgtttcaacgtcaacaacaacattattagc

gcgcagatggattacgccggtggcgtgaagttcggcatcatgctgactgaaatgcacggc

acacaacaagatacgcaagccgccattgcctggctgcaggaacaccatgtaaaagtagag

gtactgggttatgtctgaatgaaagccacgtcggaagaactcgccatttttgtttcggtc

gtagaaagcggcagctttagccgggcagcggaacaattagggcaagcaaactcagcggta

agccgggcggtgaaaaagctggagatgaaacttggcgttagcctgcttaatcggaccacg

cgacaacttagcctgacggaagaaggcgagcgttatttccgtcgcgtacagtcaattttg

caggagatggcagcggcagaatcagaaattatggagacgcgtaatacaccgcgtggactg

ttacggatcgatgccgcaactccagtggtgctgcactttctgatgccgttaattaagcct

ttccgtgaacgctatccggaagtcactttgtcgctagtctcctccgaaacgattattaat

ttgatcgaaagaaaagtggatgtcgcgatacgcgctggtacgttaacggattccagctta

cgtgccaggccgttatttaacagttatcgaaaaattatcgcctcccccgattatatttcc

cgctacgggaagccagaaacgatcgacgatttaaagcaacatgtttgcctgggattcact

gaacccgcttccctcaatacctggccgatagcctgtagcgatggacaattacatgaggtg

aagtacggtttgtcatccaatagtggggaaacactgaaacagctttgcctgagtgggaac

gggattgcgtgtttgtccgactatatgatcgacagagaaatcgctcgcggagaattggtg

gagttaatggcagataaagtgttgccagtggaaatgccattcagtgcagtctattacagc

gaccgtgcggtaagtacgcgcatccgggcttttatcgatttccttagcgagcatgtaaaa

ac-------------------------------atgggcaggataagctcgggaggaatg

atgtttaaggcaataacgacagtcgccgcactggtcatcgccaccagtgcaatggcgcag

gatgatttaaccattagcagccttgcaaagggcgaaaccaccaaagctgcatttaatcag

atggtgcaagggcataagctgcctgcctgggtgatgaaaggcggtacttatacccctgca

caaaccgtgacattgggagatgagacgtatcaggtgatgagcgcgtgcaaaccgcatgac

tgtggctcgcaacgtatcgctgtgatgtggtccgagaaatctaatcagatgacggggctg

ttctcgactattgatgagaaaacgtcgcaagagaaactcacctggttgaatgtgaacgat

gcgctttcgattgatggtaaaacggtgctgttcgcggcgttgaccggcagcctggaaaac

catccggatggctttaattttaaataaatgagcgaaaaatacatcgtcacctgggacatg

ttgcagatccatgcacgtaaactcgcaagccgactgatgccttctgaacaatggaaaggc

attattgccgtaagccgtggcggtctggtaccgggtgcgttactggcgcgtgaactgggt

attcgtcatgtcgataccgtttgtatttccagctacgatcacgacaaccagcgcgagctt

aaagtgctgaaacgcgcagaaggcgatggcgaaggcttcatcgttattgatgacctggtg

gataccggtggtactgcggttgcgattcgtgaaatgtatccaaaagcgcactttgtcacc

atctttgcaaaaccggctggtcgtccgctggttgatgattatgttgttgatatcccgcaa

gatacctggatcgaacagccgtgggatatgggcgtcgtattcgtcccgccaatctccggt

cgctaaatgacgttaccgagtggacacccgaaaagcagattgatcaaaaaatttaccgca

ctaggcccatatattcgtgaaggtaagtgcgaagataatcgattctttttcgattgtctg

gctgtatgcgtcaacgtgaaaccggcaccggaagtgcgtgagttctggggctggtggatg

gagcttgaagcacaggaatcccgttttacatacagttaccagtttggtctgttcgataaa

gcaggcgactggaagagtgttccggtaaaagacactgaagtggttgaacgactggagcac

accctgcgtgagttccacgagaagctgcgtgagctgctgacgacgctgaatctgaagctg

gaaccggcggatgattttcgtgatgaaccggtgaagttaacggcgtgaatggctgatttc

accctgtcaaaatcgctgtttagcggaaaatatcgcaatgcctcttcaacgcctggcaac

attgcctatgcgttgtttgtgctgttttgcttttgggctggggcgcaattgctgaacctg

ttagtgcatgcgcccggcgtctatgagcgtttaatgcaggtccaggaaacaggtcgccca

cgggtggaaattggtttaggtgtcggcaccattttcgggctgatcccgtttttagtaggc

tgcctcatttttgcagtggtggcgctatggctgcactggcgacatcgccgccagtaaatg

acacaacctctttttctgatcgggcctcggggctgtggtaaaacaacggtcggaatggcc

cttgccgattcgcttaaccgtcggtttgtcgataccgatcagtggttgcaatcacagctc

aatatgacggtcgcggagatcgtcgaaagggaagagtgggcgggatttcgcgccagagaa

acggcggcgctggaagcggtaactgcgccatccaccgttatcgctacaggcggcggcatt

attctgacggaatttaatcgtcacttcatgcaaaataacgggatcgtggtttatttgtgt

gcgccagtatcagtcctggttaaccgactgcaagctgcgccggaagaagatttacggcca

accttaacgggaaaaccgctgagcgaagaagttcaggaagtgctggaagaacgcgatgcg

ctatatcgcgaagttgcgcatattatcatcgacgcaacaaacgaacccagccaggtgatt

tctgaaattcgcagcgccctggcacagacgatcaattgttgaatgacccatcaattaaga

tcgcgcgatatcatcgctctgggctttatgacatttgcgttgttcgtcggcgcaggtaac

attatttttcctccaatggtcggcttacaggcaggcgaacacgtctggactgcggcattc

ggcttcctcattactgccgttggtctgccggtattaacggtagtggcgctggcaaaagtt

ggcggcggtgttgacagcctcagcacgccaatcggtaaagtcgctggcgtactgctggca

acggtttgttacctggcggtggggccgcttttcgctacgccgcgtacagctaccgtttct

tttgaagtggggattgcgccgctgacgggtgattccgcgctgccgctgtttatctacagc

ctggtctatttcgctatcgttattctggtttcgctctatccgggcaagctgctggatacc

gtgggcaacttccttgcgccgctgaaaattatcgcgctggtcatcctgtctgttgccgcg

attgtctggccggcgggttctatcagcacggcgactgaggcttatcaaaacgctgcgttt

tctaacggcttcgttaacggctatctgaccatggatacgctgggcgcaatggtgtttggt

atcgttattgttaacgcggcgcgttctcgtggcgttaccgaagcgcgtctgctgacccgt

tataccgtctgggctggcctgatggcgggtgttggtctgactctgctgtacctggcgctg

ttccgtctgggttcagacagcgcgtcgctggtcgatcagtctgcaaacggcgctgctatt

ctgcatgcttacgttcagcacacctttggcggcggcggtagcttcctgctggcggcgtta

atcttcatcgcctgcctggtaacggcagttggcctgacctgtgcttgtgcagaattcttt

gcccagtacgtaccgctctcttatcgtacgctggtgtttatcctcggcggcttctcgatg

gtggtttctaacctcggcttaagccagctgattcagatctccgtaccggtgctgaccgct

atttatccgccgtgtatcgcactggttgtattaagttttacacgctcatggtggcataat

tcgtcccgcgtgattgctccgccgatgtttatcagcctgctttttggtattctcgacggg

atcaaagcatctgcattcagcgatatcttaccgtcctgggcgcagcgtttaccgctggcc

gaacaaggtctggcgtggttaatgccaacagtggtgatggtggttctggccattatctgg

gatcgcgcggcaggtcgtcaggtgacctccagcgctcactaaatgaaatttgaactggac

accaccgacggtcgcgcacgccgtggccgcctggtctttgatcgtggcgtagtggaaacg

ccttgttttatgcctgttggcacctacggcaccgtaaaagggatgacgccggaagaagtt

gaagccactggcgcgcaaattatcctcggcaacaccttccacctgtggctgcgcccgggt

caggaaatcatgaaactgcacggcgatctgcacgattttatgcagtggaagggaccgatc

ctcaccgactccggcggcttccaggtcttcagccttggtgatattcgtaaaatcaccgaa

cagggcgttcacttccgtaacccgatcaacggcgacccgattttcctcgacccggaaaag

tcgatggagattcagtacgatcttggttcggatatcgtcatgatctttgatgagtgtacg

ccgtatcctgctgattgggattacgcaaaacgctccatggagatgtctctgcgttgggcg

aagcgtagccgtgagcgttttgacagtctcggaaacaaaaatgcgctgtttggtatcatt

cagggcagcgtttacgaagatttacgtgatatttctgttaaaggtctggtagatatcggt

tttgatggctacgctgtcggcggtctggctgtgggtgagccgaaagcagatatgcaccgc

attctggagcatgtatgcccgcaaattccggcagacaaaccgcgttacctgatgggcgtt

ggtaaaccagaagacctggttgaaggcgtacgtcgtggtatcgatatgtttgactgcgta

atgccaacccgcaacgcccgaaatggtcatttgttcgtgaccgatggcgtggtgaaaatc

cgcaatgcgaagtataagagcgatactggcccactcgatcctgagtgtgattgctacacc

tgtcgcaattattcacgcgcttacttgcatcatcttgaccgttgcaacgaaatattaggc

gcgcgactcaataccattcataaccttcgttactaccagcgtttgatggcgggtttacgc

aaggctattgaagagggtaaattagagagcttcgtaactgatttttaccagcgtcagggg

cgagaagtaccacctttgaacgttgattaaatgcattgcccattctgtttcgccgtggac

actaaggtaattgactctcgtctcgtgggcgagggttcatccgtacgccgccgtcggcag

tgtctggtgtgtaatgaacgtttcaccacctttgaagtggcggagctggttatgccgcgt

gttgtaaaaagcaacgacgtgcgtgaaccgtttaatgaagaaaaattacgtagcggaatg

ctgcgggcgctggaaaaacgtccggtgagttccgatgacgtcgaaatggcaatcaatcat

attaaatcgcagctgcgcgccaccggtgagcgcgaagtgccgagcaagatgattggcaat

ctggtgatggagcaattgaaaaagctcgataaagtcgcctatatccgttttgcctctgtc

taccgcagtttcgaagatatcaaagaatttggcgaagagatcgcgcgcctggaggactaa

atgtccagtcaatatttacgtatttttcaacagccgcgttcagccatattgctgatcctg

ggttttgcttccgggctaccgctcgccctgacatccggcaccttacaggcctggatgacg

gtcgagaatatcgatctcaaaaccattggtttcttctctctggtaggccaggcttacgtt

tttaaattcctctggtcaccgctgatggaccgctacacgcccccattttttgggcggcgg

cgcggttggctgctcgccacgcaaatcctgttattagtcgccattgcggcgatgggtttt

ctcgaaccaggcacccaactccgctggatggcggcgctggcggtggtgatcgctttttgc

tctgcctcccaggatattgtcttcgatgcgtggaaaaccgatgtgctcccggcagaagaa

cgtggtgcgggcgcggcaatcagcgtgctgggttaccgtttagggatgctggtttccggc

ggcctagccctgtggctggcagataaatggctgggctggcagggcatgtactggttgatg

gcggcgctgttgatcccctgtattatcgcgacgttgcttgcaccagaaccaaccgacacc

attcctgtgccaaaaacgctggaacaagcggttgttgcacctctgcgagatttctttggt

cgcaataatgcctggcttattttgcttcttatcgtgctgtataagctgggcgacgcattc

gccatgagcctgacaaccacgtttttgattcgcggcgtcgggtttgatgcgggtgaagta

ggcgtggttaacaaaacgcttggcttactagcgaccattgttggcgcattgtacggtggg

attttgatgcagcgcctgtcactgttccgggcactgctgattttcggcattttacaaggt

gcgtctaacgctggttactggctgctgtcgattactgataagcatctctacagcatgggc

gcagccgtctttttcgaaaacctctgtggcgggatgggcacatcagcctttgtcgcgctg

ttaatgacactatgtaataagtcattttccgctactcaatttgccctgctctcagcgctt

tctgctgtagggcgagtttatgtcggccccgtggcgggttggtttgttgaagcacacggc

tggtcgacattctatctattctccgtcgccgctgccgtaccagggcttattttgctgctg

gtttgccgccagacgcttgaatatacacgagtaaatgacaactttatctcccgtaccgca

tatccggcaggttatgcctttgccatgtggacactggcggcgggcgtcagcctgttggcc

gtgtggttactgctgttgacgatggacgcgctggatttgacgcacttctctttcctgcct

gctctgctggaagtcggggttttagtcgccctttctggcgtcgtgcttggtggtttgctg

gattatctggcgctacgaaaaacgcatctgacgtaaatgcaaacacaaatcaaagttcgt

ggatatcatctcgacgtttaccagcacgtaaacaacgctcgctaccttgaattcctcgaa

gaagcccgctgggatgggttggaaaatagcgacagttttcagtggatgacggcccataac

atcgctttcgtggtggtgaatatcaatattaactatcgtcgtccggcggtattaagcgac

ttgttaaccattacgagccagttacagcaattaaacggtaaaagcggcatcttaagccag

gtcattacactggagccggaagggcaggtggtagcggatgcgcttattacgtttgtttgt

attgatcttaaaacgcagaaagcattagctctggaaggggaattgcgcgaaaagctggag

cagatggttaagtaaatgcaacgaatcattttaatcatcattggctggctggcggtagtg

ctgggtacgctgggcgtggtattaccggtattaccgacgacgccgtttatcctgctggcg

gcctggtgctttgcccgttcttccccgcgctttcacgcctggttgctgtaccgctcatgg

tttggcagctatctacgtttctggcagaaacatcatgcgatgccgcgcggtgtcaaaccg

cgggcgattttgcttattttgctcacgtttgccatttctctgtggttcgtccagatgcca

tgggtgcgcatcatgttgctggtaattctcgcctgtttgcttttctatatgtggcgaatt

ccggtgattgatgaaaagcaagaaaagcactgaatgcaaaccagcccgctgttaacacag

cttatggaagcactgcgctgtctgccgggcgttggcccgaagtcggcgcagcgtatggcg

ttcacgctgcttcagcgcgatcgtagcggcgggatgcgtctggcgcaggcgctcacccgg

gcgatgtcggaaatcggccactgcgccgattgccgcaccttcaccgaacaggaagtctgt

aacatctgttcgaatccgcgtcgtcaggaaaacggtcaaatctgcgtggtggagagtccg

gcggacatttacgccattgagcagacggggcagttttcaggtcgttattttgtgttgatg

ggacatctgtcaccgctggacggcatcggtccggatgatatcggccttgatcgtctggaa

cagcgtctggcagaggaaaaaatcactgaagtgatcctcgccaccaaccctacggttgaa

ggtgaagctaccgctaactacattgccgagctttgcgcgcaatatgacgtggaagccagc

cgaatcgctcatggcgtaccggttggcggcgaactggaaatggtcgatggcaccacgctg

tcacactcccttgccgggcgtcataagattcgtttttaaatgcgtcagactaaaaccggt

atcctgctggcaaacctgggtacgcccgatgcccccacacctgaagcggtaaaacgctat

ctgaaacaatttttaagcgacagacgcgtggttgatacctcacggttgttatggtggcca

ttgctgcgcggcgtgattttgccgctgcgctcgccgcgtgtggcgaagctgtatgcctct

gtctggatggaaggtggctcgccgctgatggtttacagccgccagcaacagcaggcgctg

gcacaacgtttaccggagacgcccgtagcgctgggaatgagctacggctcgccatcactg

gaaagcgccgtagatgaactcctggcagagcatgtagatcatattgtggtgctgccgctt

tatccgcaattctcctgttctacggtcggtgcggtatgggatgaactggcacgcattctg

gcgcgcaaacgtagcattccggggatatcgtttatacgtgattacgccgataaccacgat

tacattaatgcactggcgaacagcgtacgcgcttcttttgccaaacatggcgaaccggat

ctgctgctgctctcttatcatggcattccccagcgttatgcagatgaaggcgatgattac

ccgcaacgttgccgcacaacgactcgtgaactggcttccgcattggggatggcaccggaa

aaagtgatgatgacctttcagtcgcgctttggtcgggaaccctggctgatgccttatacc

gacgaaacgctgaaaatgctcggagaaaaaggcgtaggtcatattcaggtgatgtgcccg

ggctttgctgcggattgtctggagacgctggaagagattgccgagcaaaaccgtgaggtc

ttcctcggtgccggcgggaaaaaatatgaatatattccggcgcttaatgccacgccggaa

catatcgaaatgatggctaatcttgttgccgcgtatcgctaaatgatctggaaacgccat

ttaacgctcgacgaactgaacgccaccagcgataacacaatggtggcgcatctgggaatt

gtgtatacccgtctgggcgatgatgtgctggaagccgaaatgccggttgatacccgtact

catcagccgtttggcctgctgcatggcggcgcgtcggcggcgctggctgaaacgctggga

tcgatggccggatttatgatgacccgtgacgggcagtgtgtggtgggcacggaacttaac

gccacccatcatcgcccggtgtctgaaggcaaggtacgcggcgtctgccagccgctgcat

cttgggcggcaaaatcagagctgggaaatcgtcgttttcgatgaacaggggcggcgttgc

tgcacttgtcggctgggtacggcagt----------atgtccttgattaacaccaaaatt

aaaccttttaaaaaccaggcattcaaaaacggcgaattcatcgaaatcaccgaaaaagat

accgaaggccgctggagcgtcttcttcttctacccggctgactttactttcgtatgcccg

accgaactgggtgacgttgctgaccactacgaagaactacagaaactgggcgtagacgta

tacgcagtatctaccgatactcacttcacccacaaagcatggcacagcagctctgaaacc

atcgctaaaatcaaatatgcgatgatcggcgacccgactggcgccctgacccgtaacttc

gacaacatgcgtgaagatgaaggtctggctgaccgtgcgaccttcgttgttgacccgcag

ggtatcatccaggcaatcgaagttaccgctgaaggcattggccgtgacgcgtcagacctg

ctgcgtaaaatcaaagcagcacagtacgtagcttctcacccaggtgaagtttgcccggct

aaatggaaagaaggtgaagcaactctggctccgtctctggacctggttggtaaaatctaa

gtgttacaacttcttttagcagtttttattggcggtggtacgggaagcgtggcgagatgg

ctgttaagtatgcgatttaacccgctgcatcaggcgattccgttggggacgctggctgca

aatctgattggggcattcatcataggaatgggattcgcctggttcagcaggatgacgaac

attgatccagtgtggaaagtattaatcaccaccggattttgtggcggtctaacaaccttc

tcaacattttcggcagaagtggtgtttttgttacaagagggccgctttggctgggcatta

ctgaacgttttcgtcaaccttctggggtcttttgccatgaccgcactggcattctggctg

ttttcggcctcaaccgcacactaaatgaacaaggttgctcaatattaccgtgaactggtt

gcgtcactgagcgaacgcctgcgcaatggcgaacgtgatatcgacgcactggtggaacag

gcgcgcgagcgcgtaataaaaacaggggagttaacgcgaaccgaggtcgatgagctgacg

cgagctgtcagacgtgacctggaagagttcgccatgagctatgaagagagcctgaaagaa

gaatctgacagcgtctttatgcgggtgattaaagaaagcttgtggcaggagctggcagac

atcaccgataaaacgcagcttgaatggcgcgaagttttccaggacctcaatcatcatggg

gtttatcacagcggagaagtggtcgggctgggaaatctggtctgcgagaaatgtcacttc

catctcccgatctacacaccggaagtgctgacgctatgcccgaaatgtggtcatgaccag

ttccagagacgcccgtttgagccgtaaatgagcgacgacaattcacacagtagtgacacg

ataagcaacaagaagggatttttctccctgttactcagccaacttttccacggtgaaccg

aaaaaccgtgacgaactgctggcgctgatccgtgattccgggcagaacgaccttatcgac

gaagatacgcgcgatatgctcgaaggggtgatggacatcgcagaccaacgcgtccgcgac

atcatgatcccccgctcccagatgattaccctgaaacgcaaccagacgctggacgaatgc

cttgatgtcatcatcgagtccgcccactcacgtttcccggtgattagcgaagacaaagat

cacattgaagggattctgatggcgaaagacttgctgccgtttatgcgcagcgatgctgaa

gccttcagcatggacaaagtgttacgtcaggcggttgtcgttcctgaaagtaagcgcgta

gaccggatgctgaaagagtttcgctctcagcgttaccacatggcgatcgttattgacgaa

ttcggtggggtttccggtctggtaaccattgaagacatcctggaactgattgttggtgag

attgaagacgaatatgacgaagaagatgatatcgacttccgtcagctgagtcgtcatacc

tggaccgtgcgcgcactggcttccattgaagacttcaacgaagcgttcggcacccacttt

agcgatgaagaagtcgacactatcggtggtctggtgatgcaggcatttgggcatcttccg

gcacgtggcgaaaccatcgacatcgacggttaccagttcaaagtggcgatggccgacagt

cggcgtattattcaggttcatgtcaaaatcccggatgactcaccccagccgaagctggat

gaataaatgagtcaggtgatcctcgatttacaactggcatgtgaagataattccgggtta

ccggaagagagccagtttcagacatggctgaatgcggtgatcccgcagtttcaggaagag

tcggaagtgacgattcgcgtggtcgataccgccgaaagccacagtctcaatctgacctat

cgcggtaaggataagccgaccaacgtgctctccttcccgtttgaagtgccgcccggcatg

gagatgtcgctactgggcgatctggttatctgccgtcaggtggttgagaaggaagcacag

gagcaaggcaaaccactggaggcgcactgggcgcatatggtggtgcacggcagtctgcat

ttgttaggttacgatcacatcgaagatgacgaagcagaagaaatggaagccctcgaaaca

gagattatgcttgctctgggctatgaggatccgtacattgccgagaaagaataaatgtat

gcattaacccagggccggatctttaccggccacgaatttcttgatgaccacgcggttgtt

atcgctgatggcctgattaaaagcgtctgtccggtagcggaactgccgccagagatcgaa

caacgttcactgaacggggccattctctcccccggttttatcgatgtgcagttaaacggc

tgcggcggcgtacagtttaacgacaccgctgaagcggtcagcgtggaaacgctggaaatc

atgcagaaagccaatgagaaatcaggctgtactaactatctgccgacgcttatcaccacc

agcgatgagctgatgaaacagggcgtgcgcgttatgcgcgagtacctggcaaaacatccg

aatcaggcgttaggtctgcatctggaaggtccgtggctgaatctggtaaaaaaaggcacc

cataatccgaattttgtgcgtaagcctgatgccgcgctggtcgatttcctgtgtgaaaac

gccgacgtcattaccaaagtgaccctggcaccggaaatggttcctgcggaagtcatcagc

aaactggcaaatgccgggattgtggtttctgccggtcactccaacgcgacgttgaaagaa

gcaaaagccggtttccgcgcggggattacctttgccacccatctgtacaacgcgatgccg

tatattaccggtcgtgaaccgggcctggcgggcgcgatcctcgacgaagctgacatttat

tgcggtattatcgctgatggcctgcatgttgattacgccaacattcgtaacgctaaacgc

cttaaaggcgacaaactgtgtctggttaccgacgccaccgcgccagcaggtgccaacatt

gaacagttcatttttgcgggtaaaacaatatactaccgtaacggactttgtgtggatgag

aacggtacgttaagcggttcatccttaaccatgattgaaggcgtgcgtaatctggtcgaa

cattgcggtatcgcactggatgaagtgctgcgtatggcgacgctctatccggcgcgtgcg

attggcgttgagaaacgtctcggcacactcgccgcaggtaaagtagccaacctgaccgca

ttcacacctgattttaaaatcaccaagaccatcgttaacggtaacgaggtcgtaactcaa

taaatgactgataacaataccgccctaaagaaagctggcctgaaagtaacgcttccacgt

ttaaaaatcctggaagttcttcaggagccggacaaccatcacgtcagtgcggaagattta

tacaaacgtctgatcgatatgggtgaagaaattggtctggctacggtatatcgcgtactg

aaccagtttgacgacgctggtatcgtcacccgccacaattttgaaggcggtaaatccgta

tttgaactgacacagcaacatcaccacgatcacctgatctgcctcgactgcggcaaggtt

atcgaatttagtgatgattcaatcgaagcgcgtcagcgtgaaattgccgcaaaacatggc

attcgcctgactaaccacagtctctatctttacggtcactgtgccgaaggcgattgccgc

gaagatgagcacgcgcacgaaggcaaataaatggtaagcaacgcctccgcattaggacgc

aatggcgtacatgatttcatcctcgttcgcgctaccgctatcgtcctgacgctctacatc

atttatatggtcggttttttcgccaccagtggcgagctgacatatgaagtctggatcggt

ttcttcgcctctgcgttcaccaaagtgttcaccctgctggcgctgttttctatcttgatc

catgcctggatcggcatgtggcaggtgttgaccgactacgttaaaccgctggccttgcgc

ctgatgctgcaactggtgattgtcgttgcactggtggttgacgtgatttatggattcgtt

gtggtgtggggtgtgtgaatgagactcgagttttcaatttatcgctataacccggatgtt

gatgatgctccgcgtatgcaggattacaccctggaagcggatgaaggtcgcgacatgatg

ctgctggatgcgcttatccagctgaaagagaaagatcccagcctgtcgttccgtcgctcc

tgtcgtgaaggtgtgtgcggttccgacggtctgaacatgaacggcaagaatggtctggcc

tgtattaccccgatttcggcactcaaccagccgggcaagaagattgtgattcgccctctg

ccaggtttaccggtgatccgcgatttggtggtagacatgggacaattctatgcgcaatat

gagaaaattaagccttacctgttgaataatgaacaaaatccgccagctcgcgagcattta

cagatgccagagcagcgcgaaaaactcgacgggctgtatgaatgtattctctgcgcatgt

tgttcaacctcttgtccgtctttctggtggaatcccgataagtttatcggcccggcaggc

ttgttagcggcatatcgtttcctgatcgatagccgtgataccgagactgacagccgcctc

gacggtttgagcgatgcattcagtgtattccgctgtcacagcatcatgaactgcgtcagt

gtatgtccgaaggggctgaacccgacgcgcgccatcggccatatcaagtcgatgttgttg

caacgtaatgcgtaaatgagtagcgtagatattctggtccctgacctgcctgaatccgta

gccgatgccaccgtcgcaacctggcataaaaaacccggcgacgcagtcgtacgtgatgaa

gtgctggtagaaatcgaaactgacaaagtggtactggaagtaccggcatcagcagacggc

attctggatgcggttctggaagatgaaggtacaacggtaacgtctcgtcagatccttggt

cgcctgcgtgaaggcaacagcgccggtaaagaaaccagcgccaaatctgaagagaaagcg

tccactccggcgcaacgccagcaggcgtctctggaagagcaaaacaacgatgcgttaacc

ccggcgatccgtcgcttgctggctgaacataacctcgacgccagcgccattaaaggcacc

ggcgtgggtggtcgtctgacccgtgaagatgtggaaaaacatctggcgaaatccccggcg

aaagagtctgcaccggcagcggctgctccggcggcgcaaccggctctggctgcacgtagt

gaaaaacgtgtcccgatgactcgcctgcgtaagcgtgtggcagagcgtctgctggaagcg

aaaaactccaccgccatgctgaccacgttcaacgaagtcaacatgaagccgattatggat

ctgcgtaagcagtacggtgaagcgtttgaaaaacgccacggcatccgtctgggctttatg

tccttctacgtgaaagcggtggttgaagccctgaaacgttacccggaagtgaatgcttct

atcgacggcgatgacgtggtttaccacaactatttcgacgtcagcatggcggtttctacg

ccgcgcggcctggtgacgccggttctgcgtgatgtcgataccctcggcatggcagacatc

gagaagaaaatcaaagagctggcagtcaaaggccgtgatggcaagctgacggttgaagat

ctgaccggtggtaacttcaccatcaccaacggtggtgtgttcggttccctgatgtctacg

ccgatcatcaacccgccgcagagcgcaattctgggtatgcacgctatcaaagatcgtccg

atggcggtgaatggtcaggttgagatcctgccgatgatgtacctggcgctgtcctacgat

caccgtctgatcgatggtcgcgaatccgtgggcttcctggtaacaatcaaagagttgctg

gaagatccgacgcgtctgctgctggacgtgtagatgagtaagattatcgcgaccttgtat

gcggtaatggacaagcgccccctgcgggcgctttccttcgtgatggcgcttctgttagca

ggatgtatgttttgggacccatcacgtttcgccgcgaagaccagtgatctggaaatctgg

catggtttattgctgatgtgggccgtctgtgctggtgtgattcacggcgtgggctttcgt

ccgcagaaggttctttggcaagggattttttgcccattgcttgccgatattgttctcatt

gtcgggctgattttcttcttcttttaagtgaatacaacgctgtttcgatggccggttcgc

gtctactatgaagataccgatgccggtggtgtggtgtaccacgccagttacgtcgctttt

tatgaaagagcacgcacagagatgctgcgtcatcatcacttcagtcaacaggcgctgatg

gctgaacgcgttgcctttgtggtacgtaaaatgacggtggaatattacgcacctgcgcgg

cttgacgatatgctcgaaatacagactgaaataacatcaatgcgtggcacctctttggtt

ttcacgcaacgtattgtcaacgccgagaatactttgctgaatgaagcagaggttctggtt

gtttgcgttgacccactcaaaatgaagcctcgtgcgcttcccaagtctattgtcgcggag

tttaagcagtgaatggccagagcgcgtggacgaggtcgtcgcgatctcaagtccgaaatc

aacattgtaccgttgctggacgtactgctggtgctgttgctgatctttatggcgacagcg

cccatcatcacccagagcgtggaggtcgatctgccagacgctactgaatcacaggcggtg

agcagtaacgataatccgccagtgattgttgaagtgtctggtattggtcagtacaccgtg

gtggttgagaaagatcgcctggagcgtttaccaccagagcaggtggtggcggaagtgtcc

agccgtttcaaggccaacccgaaaacggtctttctgatcggtggcgcaaaagatgtgcct

tacgatgaaataattaaagcactgaacttgttacatagtgcgggtgtgaaatcggttggt

ttaatgacgcagcctatctaaatgaaactcatcagtaatgatctgcgcgatggcgataag

ttgccacatcgtcatgtctttaacggcatgggttacgatggcgataatatttcaccgcat

ctggcgtgggatgatgttcctgcgggaacgaaaagttttgttgtcacctgctacgacccg

gatgcgccaaccggctccggctggtggcactgggtagttgttaatttacccgctgatacc

cgcgtattaccgcaagggtttggctctggtctggtagcaatgccagacggcgttttgcag

acgcgtaccgactttggtaaaaccgggtacgatggtgcagcgccgccgaaaggcgaaacc

catcgctacatttttaccgttcacgcgctggatgtagagcgtattgatgtcgatgaagga

gccagcggcgcgatggtcgggtttaacgttcatttccactctctggcaagcgcatcgatt

accgcgatgttcagttaaatgagtcaggtaagcactgaatttatcccgacccgtattgct

attcttacggtttctaatcggcgcggtgaagaagacgatacctccggtcactatttgcgc

gattcggcgcaagaagcgggccatcacgttgtcgataaagccattgtgaaagaaaaccgc

tacgctattcgcgctcaggtatctgcgtggatcgccagcgacgatgtacaagtggtgttg

attacggggggtactggcctgacggaaggtgatcaggctcccgaagcattgctgccgttg

ttcgaccgtgaagttgaaggttttggtgaagtgttccgtatgttgtcgtttgaagagatt

ggcacttccacgttgcaatctcgtgcggtagcgggcgtcgctaacaaaacgctgattttc

gccatgccgggttcgaccaaagcgtgccgtaccgcatgggaaaatatcatcgcgccgcag

ctggatgcccgtacgcgtccgtgtaatttccatccacatttgaagaaataaatgtcgcaa

ctgacccatatcaacgccgctggcgaagcgcacatggtggatgtctccgccaaagcggaa

accgtgcgtgaagcgcgcgccgaagcctttgtcaccatgcgcagcgagacgctggcgatg

attattgatggtcgccaccacaaaggcgacgtatttgccactgcgcgtattgccggtatt

caggcggcaaaacgcacgtgggatctgatcccgctgtgtcatccgctgatgctcagcaaa

gttgaagtcaatttacaggccgagccggagcacaatcgggtgcgtatagaaaccttatgc

cgcctgaccgggaaaaccggtgtcgaaatggaagcgttaaccgcggcctccgtggcggcg

ctgaccatttatgacatgtgcaaagcggtgcaaaaagatatggtgattggtccggtacgt

ttgctggcgaagagcggcggcaagtcgggtgactttaaggtggaagcggatgattaaatg

aagtctgtattaaaagtttcactggctgcactgaccctggcttttgcggtttcttctcat

gccgcggataaaaaattagttgtcgcgacggataccgccttcgttccgtttgaatttaaa

cagggcgataaatatgtgggctttgacgttgatctgtgggctgccatcgctaaagagctg

aagctggattacgaactgaagccgatggatttcagtgggatcattccggcactgcaaacc

aaaaacgtcgatctggcgctggcgggcattaccatcaccgacgagcgtaaaaaagcgatc

gatttctctgacggctactacaaaagcggcctgttagtgatggtgaaagctaacaataac

gatgtgaaaagcgtgaaagatctcgacgggaaagtggttgctgtgaagagcggtactggc

tccgttgattacgcgaaagcaaacatcaaaactaaagatctgcgtcagttcccgaacatc

gataacgcctatatggaactgggcaccaaccgcgcagacgccgttctgcacgatacgcca

aacattctgtacttcatcaaaaccgccggtaacggtcagttcaaagcggtaggtgactct

ctggaagcgcagcaatacggtatagcgttcccgaaaggtagcgacgagctgcgtgacaaa

gtcaacggcgcgttgaaaaccctgcgcgagaacggaacttacaacgaaatctacaaaaaa

tggttcggtactgaaccgaaataaatgagtcgtcgcgcaggtacgccaacagcaaaaaaa

gtgacgcagttagtgaacgtggaagagcacgttgaagggttccgccaggtcagagaggcg

catcggcgcgagcttattgatgattacgttgagctgatttctgacttgatcagggaagtg

ggggaagctcgccaggtagacatggctgctcgtttgggagtttcgcaaccgacggtggct

aaaatgcttaagcgactggcaactatggggctgattgaaatgatcccctggcgaggcgtg

tttttaacggcagaaggagagaagctggcgcaggaaagccgcgagcgacatcagatagtc

gaaaatttcttattggtgttgggcgtcagtccggaaatcgcccgtcgcgacgcggaaggc

atggagcaccatgttagtgaagagacgctggatgcctttcgtttgtttacccagaaacac

ggtgccaaatgaatgaatatgaaattgaaaacattattcgcagcggccttcgctgttgtc

ggcttttgcagtaccgcctctgcggtaacttatcctctgccaaccgacgggagtcgcctg

gttggtcagaatcaggtgatcaccattcctgaaggtaacactcagccgctggagtatttt

gccgcggagtaccagatggggctttccaatatgatggaagcgaacccgggtgtggatacc

ttcctgccgaaaggcggtactgtcctgaacattccgcagcagctgatcctgccggatacc

gttcatgaaggcatcgtcattaacagtgcagagatgcgtctgtattactatccgaaaggg

accaacaccgttatcgtgctgccgatcggcattggtcagttaggcaaagatacgcctatc

aactggaccaccaaagttgagcgtaagaaagcaggcccgacctggacgccgaccgccaaa

atgcacgcagagtaccgcgctgcgggcgaaccgcttccggctgtcgttccggcaggtccg

gataacccgatggggctgtatgcactctacatcggtcgcctgtatgctatccatggcacc

aacgccaacttcggtatcggcctgcgtgtaagtcatggttgtgtgcgtctgcgtaacgaa

gacatcaaattcctgttcgagaaagtaccggtcggtacccgcgtacagtttattgatgag

ccggtaaaagcgaccaccgagccagacggcagccgttatattgaagtccataatccgctg

tctaccaccgaagcccagtttgaaggtcaggaaattgtgccaattaccctgacgaagagc

gtgcagacagtgaccggtcagccagatgttgaccaggttgttcttgatgaagcgattaaa

aaccgctccgggatgccggttcgtctgaattaagtgaatataaacgtcgccgaattgtta

aatgggaattacattctgttattatttgtggtcctcgcgcttgggctatgtctcggaaag

ttacgacttggttcgatccaactgggtaattccattggcgttttagtcgtatcgctgtta

ttaggccaacaacatttcagcattaacaccgacgcgcttaatcttggctttatgctgttt

attttctgcgttggggttgaagccggaccgaactttttttccattttttttcgcgatgga

aaaaattacctaatgttagcactggtgatggttggcagtgcgctggtgatcgccttaggg

ttaggtaagctgtttggctgggatattggcctgacggccggtatgttagcaggctctatg

acgtcgacaccggttctggtcggtgctggcgatacactgcgtcattccggcatggaaagc

aggcagctctcactggcactggataatctgagcctcgggtatgccttaacctatttaatc

ggtctggtgagtttgattgttggtgcgcgttacttgccgaaattgcagcatcaggactta

cagaccagcgcccagcaaatcgcccgcgaacgtggcctggacactgatgccaaccgtaag

gtttatttaccggtgatccgcgcctatcgcgtcggcccggagctggtggcctggaccgac

ggcaaaaatctgcgtgaactgggtatttatcgacaaaccggctgctacattgaacgtatt

cgacgtaacgggattctggcaaatccagacggtgatgccgtgctacaaatgggcgatgaa

atagcgttggtaggctatcccgacgcccatgcccgactcgatcccagcttccgtaacggc

aaagaagttttcgatcgtgaccttctcgatatgcgtatcgtcactgaagaagtggtcgtt

aaaaaccataacgccgttggcaaacgtctcgcacaactgaagttgaccgaccacggttgc

ttccttaaccgcgtcattcgtagccagattgagatgccgatagatgacaacgtcgtgctt

aacaaaggtgacgttttacaagtcagcggcgatgcccgtcgcgtaaaaaccatcgccgat

cgcattggctttatctcgattcacagccaggtcactgacttgctggcattctgcgccttc

tttgttattgggctgatgatcgggatgatcacattccagttcagcacattcagtttcggc

atggggaacgctgccgggttgttattcgccggaattatgctgggctttatgcgtgctaac

cacccgaccttcggttacattccgcaaggtgcattaagcatggtgaaagagttcggcttg

atggtgtttatggcaggcgttggtctgagcgccggtagcggtattaataacggcctgggc

gcgattggcggtcaaatgttgattgccggattgattgtcagtctggtgcccgtggttatc

tgtttcttgttcggtgcttatgtattgcgaatgaaccgcgcactgttgttcggcgcaatg

atgggcgcacgcacctgcgcgccggcaatggagatcatcagtgatacagctcgcagtaac

atcccggcgctgggctatgcgggcacctacgcaatcgccaacgttctgctgacgctggca

gggacaatcatcgtcatggtatggccaggattaggataagtgaaacataaacaacgttgg

gcgggggcaatctgctgttttgtcctcttcattgtggtgtgcctttttctggcgacgcac

atgaaaggcgcttttcgggctgccgggcatcctgaaatcggcttgctgtttttcattctt

cctggagcagttgccagtttcttttcacagcgtagagaagtcctgaaacctctgtttggc

gcaatgctggcggcaccctgttcaatgcttattatgcggctgtttttttcaccgacccgc

tcattctggcaagagctggcatggttactaagcgcggtgttctggtgtgcgctgggggca

ctgtgtttcttatttatcagtagtttgtttaaaccacagcacagaaaaaatcagtaaatg

cgcgcgatcggtaaattgcctaagagcgtgttgatactggaatttatcggaatgatgttg

ctggcggtggcgctgctgtcggtaagcgactccctgtcgctgcctgagccattttctcgg

ccagaagtgcagattctgatgatttttctcggtgttttgctcatgcttcccgctgcggtg

gtggttattcttcaggtggcaaaacgtcttgccccacagctgatgaaccgtccaccgcaa

tattcacgttcagaaagagaaaaagataatgacgccaaccattgagtgaaaattgccata

ttgtcccgggatggaacgctctattcgtgtaagcggctgcgtgaagccgctatacagcgc

ggtcacctggttgaaattcttgatccgctttcttgctacatgaacataaatcctgcggcg

tcttctattcactacaaaggccgcaagttaccccattttgacgcagtgatcccgcgtatt

ggcaccgccattaccttttatgggacggcggcactgcgccagttcgagatgctggggagc

tatccgctcaatgagtcggtcgccattgcccgggcgcgtgacaaattgcgttccatgcaa

ctgctggcgcgtcagggcatcgacctgcctgtcacgggcattgcgcattcgccggatgat

accagcgatttaatcgacatggtcggtggtgcgccgctggtggtcaagttggttgaaggc

acgcagggaattggcgtcgtgctggcggagacgcgtcaggcggcggaaagcgtgattgac

gctttccgcggtctgaacgcgcatattctggtgcaggaatatatcaaagaggcgcaaggg

tgcgatatccgctgtctggttgttggcgatgaagtggtcgctgcgattgaacggcgggcg

aaagagggcgattttcgttccaatttgcatcgtggcggcgcggcaagtgtcgccagtatc

acaccacaggagcgtgaaatcgcgataaaagccgcgcgaacgatggcgctggacgttgct

ggtgtggatattctgcgtgctaatcgcgggccgttggtgatggaagtgaatgcgtcgccg

gggctggaaggaatagaaaaaaccaccggtatcgacatcgcgggtaaaatgatccgctgg

atcgaacgctacgctacgacagaatattgcctgaaaacgggtggttagatgtttgagtat

ttacccgaactgatgaaagggctgcacaccagcctgacgctaaccgttgcctcgctgatt

gtggcactgattctggcgttgatttttaccatcatcctgacgctgaaaacgccggtgctg

gtgtggctggtgcggggttatatcacgctgtttaccggtacaccgctgctggtgcagatc

ttcctgatttattacgggccgggtcagttcccgacgttgcaggagtatccggcactgtgg

catttgttgtcagaaccgtggttatgtgcgctgattgcgttgtcgctgaacagtgcggcg

tataccacgcagctgttttacggtgcaattcgtgcgatcccggaaggtcagtggcagtcc

tgtagcgccctgggaatgagcaaaaaagatacgctggcgatcctgctgccgtatgccttt

aaacgctcgctctcttcttattccaacgaagtggtgctggtgttcaaaagtacctctctg

gcttacaccattacgctgatggaagtgatgggatacagccagttgttgtacggacgcacc

tacgatgtaatggtgttcggcgcggcagggattatttatctggtcgtcaacggcctgctg

acgctgatgatgcgtctgatcgagcgcaaagcactggcatttgaacggcggaattaaatg

aatgaattttttcctttagcaagcgccgccgggatgaccgtcggccttgccgtttgtgca

ttgattgtcggactggcgctggcgatgttctttgcggtatgggagtcggcaaaatggcgt

cctgtcgcgtgggcaggttcagcgctggtaaccattctgcgtggcctgccagaaattctg

gtggtgctgtttatctattttggttcctcgcagctgctgctgacgctttcggatggcttc

actatcaatcttgggttcgtgcagatcccggtgcagatggacattgagaacttcgacgtg

agtccgttcctttgtggtgtcatcgctctgtcactgctgtatgccgcctatgcctcgcaa

acgctgcgcggcgcgttgaaagcggtgccgatgggtcagtgggaatccggccaggcgctg

gggctgtcgaaatcggctatctttttccgtctggtgatgccgcagatgtggcgtcatgcg

ctgcctggcctcggtaaccagtggctggtgctgctgaaagataccgcgctggtcagtttg

attagtgtgaatgatttaatgctgcaaaccaaaagcatcgctactcgtacccaggaacca

tttacctggtacattgtggcggcggcgatttacctggtgatcaccctgctcagtcagtac

attctcaaacgcattgacctgcgcgcgacacgttttgagcggaggcccagctaaatgcaa

ttttccacaactccaactctggaaggccagaccatcgttgaatattgcggtgtggtgact

ggcgaagcgattttaggcgcgaatattttccgtgatttctttgccggtatccgcgatatc

gttggcggacgctccggtgcctacgaaaaagagctacgtaaagcgcgggagatcgccttt

gaggagcttggctcccaggcgcgggcgctgggggccgatgccgtagtcggtattgatatc

gactacgaaacggtcgggcaaaacggcagtatgctgatggttagcgtcagcggtacggcg

gtgaaaacgcgtcgatgaatgacggcaattgccccggttattaccattgatggcccaagc

ggtgcagggaaaggcaccttgtgtaaggctatggcggaagcgttgcaatggcatctgctg

gattcgggtgcaatttatcgcgtactggcattggcggcattacatcaccatgttgatgtt

gcgtcggaagacgcgctggtgccgctggcatcccatctggatgtacgttttgtgtcaacc

aatggcaatctggaagtgatcctcgaaggggaagatgtcagcggcgaaattcgtactcag

gaagtggcgaatgcagcttcacaagtcgcggcattcccacgcgttcgtgaagcattattg

cgtcgccaacgcgcgtttcgcgaattaccaggcctgattgccgatggccgcgacatggga

acggtggtattccctgatgcgccagtgaaaattttccttgacgcctcctcggaagaacgt

gcgcatcgccgcatgctacagttgcaggagaagggctttagtgttaactttgagcgcctt

ttggccgagatcaaagaacgcgacgaccgcgatcgtaaccgagcggtagcgccactggtt

ccggcagccgatgctttagtgttggattccaccaccttaagcattgagcaagtgattgaa

aaagcgctacaatacgcgcgccagaaattggctctcgcataaatgactgaatcttttgct

caactctttgaagagtccttaaaagaaatcgaaacccgcccgggttctatcgttcgtggc

gttgttgttgctatcgacaaagacgtagtactggttgacgctggtctgaaatctgagtcc

gccatcccggctgagcagttcaaaaacgcccagggcgagctggaaatccaggtaggtgac

gaagttgacgttgctctggacgcagtagaagacggcttcggtgaaactctgctgtcccgt

gagaaagctaaacgtcacgaagcctggatcacgctggaaaaagcttacgaagatgctgaa

actgttaccggtgttatcaacggcaaagttaagggcggcttcactgttgagctgaacggt

attcgtgcgttcctgccaggttctctggtagacgttcgtccggtgcgtgacactctgcac

ctggaaggcaaagagcttgaattcaaagtaatcaagctggatcagaagcgcaacaacgtt

gttgtttctcgtcgtgccgttatcgaatccgaaaacagcgcagagcgcgatcagctgctg

gaaaacctgcaggaaggcatggaagttaaaggtatcgttaagaacctcactgactacggt

gcattcgttgatctgggcggcgttgacggcctgctgcacatcactgacatggcctggaaa

cgcgttaagcatccgagcgaaatcgtcaacgtgggcgacgaaatcactgttaaagtgctg

aagttcgaccgcgaacgtacccgtgtatccctgggcctgaaacagctgggcgaagatccg

tgggtagctatcgctaaacgttatccggaaggtaccaaactgactggtcgcgtgaccaac

ctgaccgactacggctgcttcgttgaaatcgaagaaggcgttgaaggcctggtacacgtt

tccgaaatggattggaccaacaaaaacatccacccgtccaaagttgttaacgttggcgat

gtagtggaagttatggttctggatatcgacgaagaacgtcgtcgtatctccctgggtctg

aaacagtgcaaagctaacccgtggcagcagttcgcggaaacccacaacaagggcgaccgt

gttgaaggtaaaatcaagtctatcactgacttcggtatcttcatcggcctggacggcggc

atcgacggcctggttcacctgtctgacatctcctggaacgttgcaggcgaagaagcagtt

cgtgaatacaaaaaaggcgacgaaatcgctgcagttgttctgcaggttgacgcagaacgt

gaacgtatctccctgggcgttaaacagctcgcagaagatccgttcaacaactgggttgct

ctgaacaagaaaggcgctatcgtaaccggtaaagtgactgcagttgacgctaaaggcgca

accgtagaactggctgatggcgttgaaggttacctgcgtgcttctgaagcatcccgtgac

cgcgttgaagacgctaccctggttctgagcgttggcgacgaagttgaagctaaattcacc

ggcgttgatcgtaaaaaccgcgcaatcagcctgtctgtacgtgcgaaagacgaagctgac

gagaaagatgcaatcgcaactgttaacaaacaggaagatgcaaacttctccaacaacgca

atggctgaagctttcaaagcagctaaaggcgagtaaatggaacagctgcgtgccgaatta

agccatttactgggcgaaaaactcagtcgtattgagtgcgtcaatgaaaaagcggatacg

gcgttgtgggctttgtatgacagccagggaaacccaatgccgttaatggcaagaagcttt

agtacgcccggaaaagcccgacaactggcatggaaaaccaccatgctggcaagaagtggg

actgtccgtatgccgactatttatggtgtgatgacgcatgaagaacaccccggccccgat

gtcctgctactggagcggatgcgtggtgtttcggtggaggcaccagcccgaacaccagaa

cgctgggaacaactcaaagaccaaatcgttgaagccttactggcctggcaccgtcaggac

agtcgcggttgcgtcggcgcggtcgacaatactcaggaaaatttctggccctcatggtac

cggcaacatgttgaagtgctatggaccacgctcaatcagttcaataacaccggtctgacg

atgcaggataagcggatcctgtttcgcactcgcgaatgtctcccggcattatttgaaggc

tttaacgacaatagtgtgttgattcacggtaacttctgtttacgcagcatgctgaaagat

tcgcgcagcgatcagttactggcgatggtcgggccgggactaatgctttgggcaccgcga

gaatacgaactgttccgactaatggataattctctggcggaagatttgctctggagttac

ctgcaacgcgcgccagtggcggagtcgttcatctggcggcgttggttgtatgtgttatgg

gatgaagttgcgcaactggttaataccggacgatttagtcggcgcaacttcgatctggca

tcaaaatcactcttgccgtggctcgcctgaatgaactatcgtattattccggtcaccgca

ttctcccagaactgttcattgatctggtgtgaacaaacccgcctggccgcactggtcgat

cctggcggcgatgcggaaaaaatcaaacaggaagttaatgacagcggcctgacactgatg

cagatcctgctgacgcatggtcatctggaccacgttggcgcagcggcggaactggcgcaa

cattacggcgtgccggtttttggcccggaaaaagaagatgagttctggctgcaaggcttg

cctgcgcaaagtcgtatgtttggtctggaagagtgtcagccgctgacgccagatcgttgg

ctgaaagaaggcgataccatcagcatagggaatgtgactttacaggtgttacattgccct

gggcatacgccgggtcatgtcgtgttttttgatgatcgggcaaagctgctgatttctggc

gatgttattttcaaaggcggagtagggcgcagtgacttcccgcgtggcgatcataatcaa

ctgatttcttcaatcaaagataaattgctgccactgggggatgacgtgacatttattccg

ggtcacggaccattatccacacttggttatgaacgcctgcataatcccttcctgcaagac

gaaatgcccgtctggtaaatgtttgagaacattaccgccgctcctgccgacccgattctg

ggcctggccgatctgtttcgtgccgatgaacgtcccggcaaaattaacctcgggattggt

gtctataaagatgagacgggcaaaaccccggtactgaccagcgtgaaaaaggctgaacag

tatctgctcgaaaatgaaaccaccaaaaattacctcggcattgacggcatccctgaattt

ggtcgctgcactcaggaactgctgtttggtaaaggtagcgccctgatcaatgacaaacgt

gctcgcacggcacagactccgggtggcactggcgcactacgcgtagctgccgatttcctg

gcaaaaaataccagcgttaagcgagtgtgggtgagcaacccaagctggccgaaccataag

agcgtctttaactctgcaggtctggaagttcgtgaatacgcttattatgatgcggaaaac

cacacccttgacttcgatgcactgattaacagcctgaacgaagctcaggctggcgacgta

gtgctgttccatggctgctgccacaacccaaccggtatcgaccctacgctggaacaatgg

cagacactggcacaactctccgttgagaaaggctggttaccgctgtttgacttcgcttac

cagggttttgcccgtggtctggaagaagatgctgaaggactgcgcgctttcgcggctatg

cataaagagctgattgttgccagttcctactctaaaaactttggcctgtacaacgagcgt

gttggcgcttgtactctggttgctgccgacagtgaaaccgttgatcgcgcattcagccaa

atgaaagcggcgattcgcgctaactactctaacccaccagcacacggcgcttctgttgtt

gccaccatcctgagcaacgatgcgttacgtgcgatttgggaacaagagctgactgatatg

cgccagcgtattcagcgtatgcgtcagttgttcgtcaatacgctgcaggaaaaaggcgca

aaccgcgacttcagctttatcatcaaacagaacggcatgttctccttcagtggcctgaca

aaagaacaagtgctgcgtctgcgcgaagagtttggcgtgtatgctgttgcttctggtcgc

gtaaacgtggccgggatgacaccagataacatggctccgctgtgcgaagcgattgtggca

gtgctgtaaatgcgtgtcatcaccctggcgggtagtcctcgctttccttctcgctccagc

tccttgctggaatatgcgcgggaaaaactaaatggcctggatgtagaggtttatcactgg

aatctgcaaaacttcgccgcggaagatctgctttatgctcgtttcgatagtccggcactc

aataccttcaccgaacagttgcaacaggctgacgggctgattgtcgccacgcctgtgtat

aaagccgcttattccggtgcgctgaaaaccctgctcgacctgctgccagaacgtgcgttg

caaggcaaagtggtgctaccgctggcgacgggcggtacggtggcccatctgctggcggtc

gattatgcccttaagccagttttaagtgcgctgaaagcccaggagatcctgcacggcgtg

tttgccgatgactcacaagtaattgattaccatcacaaaccccagttcacgccgaattta

caaacccgtcttgataccgcgctagaaactttctggcaggcattgcaccgccgcgatgtt

caggttcctgatcttcattctctgcgaggtaatgcccatgcgtaaatgcgtaccgttttg

aacattctgaactttgtgcttggcggatttgccaccactctgggctggttgttggcgact

ctggtcagtattgtgctgatttttaccttaccgctgacacgatcctgctgggagatcact

aaactgtctctggtgccttatggcaatgaagctattcatgtcgatgaactgaacccggct

ggcaaaaatgtgctgctgaatactggcggtacggtattgaatattttctggctgattttc

tttggctggtggttatgcctgatgcacattgcaacgggcatcgcacaatgtatttcaatc

attggcattcctgtcggcattgcgaactttaaaattgccgctattgcactatggccggtt

ggtcgtcgcgtggtatcggtagaaacagcgcaagctgcgcgtgaagccaatgcacgtcgt

cgttttgaataaatgaaaaccggcatcgtgaccaccttgattgcattatgtttgccggta

tccgtttttgccaccacgctgcggctttcaaccgatgtcgatcttctggtactcgacggc

aagaaggtttccagctctctgctgcgcggtgcggacagcattgaactggataatggaccg

catcagttagtgtttcgcgttgagaagacgattcatctttccaatagtgaagaacggctg

tatatctccccaccgctggtggtcagttttaatacccagctcatcaaccaggtcaatttt

cgcctgcctcgcctggagaatgagcgggaagctaaccattttgatgccgcgccgcgcctt

gaattgttggatggcgatgcgacgccgattccggtaaagctggatattctcgccattacc

tcaacggcaaaaacgattgattatgaggtcgaggttgaacgctataacaaatccgcgaaa

cgcgcttcactaccgcaatttgccacgatgatggcagatgacagtacgctgctttcgggt

gtttccgagctggatgctattccaccgcagtctcaggtgctcacagaacaacggctgaag

tattggtttaaactggctgacccacaaacgcgaaatactttcctgcaatgggcggaaaaa

caaccatcttcctgaatggatcgtattgttagttcttcacatgaccgtacatcactgctt

agcacccataaagtgctgcgtaatacctattttctgctgagcctgacgctggccttttcg

gcgattaccgcaactgccagtacggtgctgatgctgccatctccgggtctgattctgacg

ctggtgggtatgtatggtttgatgttcctgacctataaaacggcgaataagccgaccggg

attatctccgcattcgcctttaccggttttctgggttatatcctcggaccaattctgaac

acctatctgtctgccggaatgggtgacgtaatcgctatggcactgggcggaacggcgtta

gtgttcttctgctgctctgcatatgtgctgaccacccgcaaagatatgtcgttcctcggc

ggtatgctgatggcgggtattgtggtggtgctgattggtatggttgctaatatcttcctg

cagctgcctgctctgcatctggcgatcagcgcggtcttcattttgatctcctctggcgct

attttgtttgaaaccagcaacatcattcatggcggtgaaacgaactatattcgtgccacg

gttagcctgtatgtttctctgtacaacatcttcgtcagcctgctgagcattctgggcttc

gccagccgcgattaaatgccacatcacattgttattgttgaagatgagccggttacccag

gcgcgattacaatcctacttcactcaggaggggtataccgtttccgttacggcgagcggt

gccgggctgcgggaaattatgcagaatcagccggtggatttaattctgctggatatcaac

ttgcccgatgaaaatggcctgatgttaacccgcgccctgcgagaacgctcaacggtgggg

attattctggttaccggacgcagcgatcggattgaccgtattgttgggctggaaatgggc

gcagacgattacgtcaccaaaccgctggaactgcgcgaactggtagtacgggtaaaaaat

ctgctctggcgaatcgacctcgcgcgacaagttcaaccgctcactcaggacaactgctat

cgctttgcgggttattgcctgaatgtgtcgcgccatacgctggagcgggatggtgagccg

attaaactgacccgcgcagagtatgaaatgttggtggcatttgtgacgaatccgggagaa

attctcagccgtgaacgtctgctgcgtatgctctctgcgcgtcgggtggaaaaccctgac

ctgcgcaccgtcgatgtgttaattcgtcgtttacgccataaactcagcgcggatttactg

gtgacgcaacatggcgaaggttatttcttagccgctgatgtgtgctgaatggctaatgtt

acggtgacttttactattaccgaattttgcctgcataccggcatctctgaagaggaattg

aatgaaattgtcggtttgggggtggttgaaccgcgtgagattcaggaaacaacctgggta

tttgacgaccatgccgccatcgtggtgcaacgcgcggtgcgcctgcgtcatgaactggct

ctggactggccggggatcgcggtggcgctgacgttaatggatgatattgcgcacctgaag

caggaaaaccgcctgctgcgccagcggctttcccggtttgtagctcatccgtgaatggaa

ttaaaggattattacgccatcatgggcgtgaaaccgacggacgatctcaagacaatcaag

accgcctatcgtcgacttgcccgcaaataccatcctgatgtcagcaaagaaccggatgcc

gaagcccgcttcaaagaggtcgctgaagcctgggaagtgttaagtgatgaacaacgtcgc

gctgagtatgatcagatgtggcaacatcgcaacgatccgcaatttaaccgtcagttccat

catggcgacggacagagttttaacgccgaagattttgacgatatcttctcgtcaattttc

ggtcagcatgcccgccagagccgtcaacgccccgccacacgcggccacgatattgaaatc

gaagtggcggtattcctcgaagaaacgcttactgagcataagcgtaccatcagctataac

ctgccggtttataacgcctttggcatgatcgaacaggaaatcccgaaaacgctgaatgtg

aagatcccggcgggcgtcggcaatggtcaacgtatccgcctgaaaggccaggggacgccg

ggcgaaaacggcggtccaaatggcgacctgtggctggtgattcatattgcgccacatccg

ctgtttgatattgtcggccaggatctggaaattgtggtgccggttagcccgtgggaagcg

gcgctgggtgctaaagtcaccgttccaacactgaaagaaagcattttgctgactatcccg

cctggcagccaggccgggcaacgattgcgcgttaaaggcaaaggtctggtgagcaaaaaa

cagaccggcgatctgtatgcggtactgaaaatcgtgatgccgccgaaaccggatgaaaac

actgccgcgctgtggcagcaactggcagacgcccagtcgtcttttgatccacgtaaagat

tgggggaaagcataaatgaacgagttttctatcctctgtcgtgtgctgggttcgctctat

taccgccaaccacaagatcctttactggtgccgctgtttactctgattcgtgaggggaaa

ctggctgcgaactggccactggagcaggatgagttactgacgcgtttacagaaaagttgt

gatatgacgcaagtctctgccgattacaatgcgttgtttatcggcgatgaatgtgccgtg

ccgccatatcgtagcgcatgggttgaggacgcaacggaagcggaagtgcgcgcttttctt

tccgaacgagggatgccactggcggatacgccagccgatcacatcggcacattgctgctt

gcggcttcctggctggaagatcagtcaacggaagatgagagcgaagcactggaaacactg

ttcagtgagtatctgttaccctggtgtggcgcgttccttggcaaagtggaggcccatgca

accacgcctttctggcgcaccatggcaccgctaacccgtgatgccattagtgcaatgtgg

gacgagctggaagaggattctgaagagtaaatgaaaacgcgtattcatgttgtgcagggt

gatattaccaaactggccgttgatgtgattgtgaatgcggctaatccgtcattaatggga

ggcggcggcgtcgatggggccattcatcgcgcagcgggtccggccctgctggatgcctgt

ttaaaagtcaggcaacagcagggcgattgccctacggggcatgccgttattacgcttgca

ggcgatcttcccgctaaagccgtagtgcacaccgtcgggccagtctggcgtggtggtgaa

caaaacgaagaccagcttttgcaggatgcctatctcaatagcctacgactggtggcggca

aacagctatacgtcagtggcttttactgcaatcagtactggggtttatggttaccctcgt

gcggcagcggctgaaatcgcagtaaaaaccgtttcagaatttattacccgtcacgcttta

cccgaacaggtatactttgtctgttatgatgaagaaaacgcccacctctacgaaagactc

cttacccaacaaggagatgaatgaatgactgcaccatcccaggtattaaagatccgccgc

ccagacgactggcaccttcacctccgcgatggcgacatgttaaaaactgtcgtgccgtat

accagcgaaatttatggacgggctatcgtaatgcccaatctggctccgcccgtgaccacc

attgaggctgccgtggcgtatcgccaacgtattcttgacgccgtacctgccgggcacgat

ttcaccccattgatgacctgttatttaacagattcgctggatcctaatgagctggagcgc

ggatttaacgaaggcgtgttcaccgctgcaaaactttacccggcaaacgcaaccactaac

tccagccacggcgtgacgtcaattgacgcaatcatgccggtacttgagcgcatggaaaaa

atcggtatgccgctactggtgcatggtgaagtgacacatgcagatatcgacatttttgat

cgtgaagcgcgctttatagaaagcgtgatggaacctctgcgccagcgcctgactgcgctg

aaagtcgtttttgagcacatcaccaccaaagatgctgccgactatgtccgtgacggaaat

gaacggctggctgccaccatcactccgcagcatctgatgtttaaccgcaaccatatgctg

gttggaggcgtgcgtccgcacctgtattgtctacccatcctcaaacgcaatattcaccaa

caggcattgcgtgaactggtcgccagcggttttaatcgaatattcctcggtacggattct

gcgccacatgcacgtcatcgcaaagagagcagttgcggctgcgcgggctgcttcaacgcc

ccaaccgcgctgggaagttacgctaccgtctttgaagagatgaatgctctgcagcacttt

gaagcattctgttctgtaaatggcccgcagttctatgggttgccggtcaacgacacattc

atcgaactggtacgtgaagagcatcaggttgctgaaagcatcgcactgactgatgacacg

ctggtgccattcctcgccggggaaacggtacgctggtccgttaaacaataagtgaagcta

tacatttacgatcactgcccttactgcctcaaagcccgcatgattttcggcctgaaaaat

atccccgtcgaattacatgttctgctcaacgacgacgcagaaacacccacccggatggtc

ggtcaaaaacaggttcccattctgcaaaaagatgacagccgctatatgccagaaagcatg

gatatcgttcactatgtcgataaactcgacggcaaaccgttactgaccggcaaacgttcc

cctgcaattgaagagtggctgcgcaaggtcaatggctacgccaacaaactgctgttgccg

cgttttgccaaatcggcatttgatgagttttctactcccgccgcgcgcaaatatttcgtc

gacaagaaagaggccagcgcgggtaattttgccgacctgctggcccactctgacggtctg

attaagaatatcagcgatgatttacgtgcgctggacaaactgatcgtcaaaccgaacgcc

gtgaatggcgaactttcggaagatgatattcagctattcccgctactgcgtaatctgacg

ctggtagccggaattaactggccaagccacgttgctgattaccgcgataatatggcgaaa

cagacacaaatcaatttgttatcatcaatggcgatttaaatgtttggctatcgcagtaac

gtgccaaaagtgcgcttaaccacagaccgactggtcgtgcgtctggtgcatgatcgtgat

gcctggcgtcttgcggattattacgcagagaatcgccatttcctcaagccctgggagcca

gtgcgcgacgaaagccactgctatccatcaggctggcaggccaggctggggatgattaac

gaatttcataaacaaggttcagctttctactttggcttattcgacccggacgaaaaagag

attattggcgttgccaatttttccaatgttgttcgcggctcttttcatgcctgctatctc

ggttattcgattgggcaaaaatggcagggcaaaggactcatgtttgaagccctgaccgca

gccattcgttatatgcagcgcacccaacatattcatcgcattatggctaattatatgccg

cacaataaacgtagcggtgatttactggcgcgactgggttttgaaaaagaaggctatgcg

aaagactatctgttgattgatggacaatggcgcgatcacgtactgacggcattaactacc

ccagactggacgcccggccgctaaatgtatacgaagattattggtactggcagctatctg

cccgaacaagtgcggacaaacgccgatttggaaaaaatggtggacacctctgacgagtgg

attgtcactcgtaccggtatccgcgaacgccacattgccgcgccaaacgaaaccgtttca

accatgggctttgaagcggcgacacgcgcaattgagatggcgggcattgagaaagaccag

attggcctgatcgttgtggcaacgacttctgctacgcacgctttcccgagcgcagcttgt

cagattcaaagcatgctgggcattaaaggttgcccggcatttgacgttgcagcagcctgc

gcaggtttcacctatgcattaagcgtagccgatcaatacgtgaaatctggggcggtgaag

tatgctctggtcgtcggttccgatgtactggcgcgcacctgcgatccaacagatcgtggg

actattattatttttggcgatggcgcgggcgctgcggtgctggctgcctctgaagagccg

ggaatcatctccacccatctgcatgccgacggtagctatggtgagttgctgacgctgcct

aatgctgaccgtgtgaatccagagaattcaattcatctgacgatggcgggcaacgaagtc

ttcaaggttgcggtaacggaactggcgcacatcgttgatgagacgctggcggcgaataat

cttgaccgttctcaactggactggctggttccgcatcaggctaacttgcgtattatcagt

gcaacggcgaaaaaactcggtatgtcgatggacaatgtcgtggtgacgctggatcgccac

ggtaatacctctgcggcctctgtcccgtgcgcgctggatgaagctgtacgcgacgggcgc

attaagccggggcagttggttctgcttgaagcctttggcggtggattcacctggggctcc

gcgctggttcgtttctagatgtttaagaatgcatttgctaacctgcaaaaggtcggtaaa

tcgctgatgctgccggtatccgtactgcctatcgcaggtattctgctgggcgtcggttcc

gcgaatttcagctggctgcccgccgttgtatcgcatgttatggcagaagcaggcggttcc

gtctttgcaaacatgccactgatttttgcgatcggtgtcgccctcggctttaccaataac

gatggcgtatccgcgctggccgcagttgttgcctatggcatcatggttaaaaccatggcc

gtggttgcgccactggtactgcatttacctgctgaagaaatcgcctctaaacacctggcg

gatactggcgtactcggagggattatctccggtgcgatcgcagcgtacatgtttaaccgt

ttctaccgtattaagctgcctgagtatcttggcttctttgccggtaaacgctttgtgccg

atcatttctggcctggctgccatctttactggcgttgtgctgtccttcatttggccgccg

attggttctgcaatccagaccttctctcagtgggctgcttaccagaacccggtagttgcg

tttggcatttacggtttcatcgaacgttgcctggtaccgtttggtctgcaccacatctgg

aacgtacctttccagatgcagattggtgaatacaccaacgcagcaggtcaggttttccac

ggcgacattccgcgttatatggcgggtgacccgactgcgggtaaactgtctggtggcttc

ctgttcaaaatgtacggtctgccagctgccgcaattgctatctggcactctgctaaacca

gaaaaccgcgcgaaagtgggcggtattatgatctccgcggcgctgacctcgttcctgacc

ggtatcaccgagccgatcgagttctccttcatgttcgttgcgccgatcctgtacatcatc

cacgcgattctggcaggcctggcattcccaatctgtattcttctggggatgcgtgacggt

acgtcgttctcgcacggtctgatcgacttcattgttctgtctggtaacagcagcaaactg

tggttgttcccgatcgtcggtatcggttatgcgattgtttactacaccatcttccgcgtg

ctgattaaagcactggatctgaaaacgccgggtcgtgaagatgcgactgaagatgcaaaa

gcaacaggtaccagcgaaatggcaccggctctggttgctgcatttggtggtaaagaaaac

attactaacctcgacgcatgtattacccgtctgcgcgtcagcgttgctgatgtgtctaaa

gtggatcaggctggcctgaagaaactgggcgcagcgggcgtagtggttgctggttctggt

gttcaggcgattttcggtactaaatccgataacctgaaaaccgagatggatgagtacatc

cgtaaccactaaatgagaaaaggatgctttgggctgatgtctctggcgttgttactgctg

gtgggctgtcgttcacatccggaaattccggtgaatgatgagcaatcgctggtgatggag

tcatctttattggctgcgggcatcagtgcagaaaagcccgtcctttcgacgtctgatatt

caaccttcagcatcctcaacgctttataacgaaaggcaagaacccgttaccgttcattat

cgtttttactggtatgacgccagagggctggagatgcatcctctggaaaggccacgcagc

gttaccattcccgcacattcggcggtaacgctgtacggcagcgccaattttctgggggcg

cacaaagtcagactttatctatatttgtaagtgccgtttcgcagcaataatcccatcacg

cgcgacgaattgctgtcgcgctttttcccgcagtttcatcccgtcacgacgtttaatagt

gggcttagtggcgggagttttctcattgaacatcagggccagcgttttgttgtgcgtcag

ccgcacgatcctgatgcgccgcagtccgcgttcttgcgccagtatcgggctttatcacaa

ctacccgcatgcattgcaccgaagccgcatttatatctccgtgactggatggtagtcgac

tatctgcccggcgcggtaaaaacgtatttgccggataccaacgaactggcaggcttgctg

tattatctacatcaacaaccacgttttggctggcgaataacgctgttgccgttactggaa

ctgtactggcagcaaagcgatccggcgcggcggacagtgggttggctgcgaatgttaaaa

tgtctgcgcaaagcgcgggaaccacggcctttacgcttaagtccattgcatatggatgtc

cacgccggaaatttagtgcatagcgcgtcagggttaaaactcatcgactgggagtatgca

ggagatggtgatatcgcgctggaactggcggcggtgtgggtggaaaatactgaacagcac

cggcaattggtcaatgactatgccactcgcgcgaagatttatccggcgcaattatggcgt

caggtcaggcgatggtttccctggctgctgatgctcaaagcagggtggtttgagtaccgc

tggcgacaaaccggcgatcaacaatttatcaggctggccgatgacacctggcggcagcta

ttaataaaacaataaatgaaaaaattactgcgtctttttttcccgctctcgctgcgggta

cgttttctgttggcaacggcagcggtagtactggtgctttcgcttgcctacggaatggtc

gcgctgatcggttatagcgtcagtttcgataaaactacgtttcggctgttacgtggcgag

agcaatctgttctatacccttgcgaagtgggaaaacaataagttgcatgtcgagttaccc

gaaaatatcgacaagcaaagccccaccatgacgctaatttatgatgagaacgggcagctt

ttatgggcgcaacgtgacgtgccctggctgatgaagatgatccagcctgactggctgaaa

tcgaatggttttcatgaaattgaagcggatgttaacgataccagcctcttgctgagtgga

gatcattcgatacagcaacagttgcaggaagtgcgggaagatgatgacgacgcggagatg

acccactcggtggcggtaaacgtctacccggcaacatcgcggatgccaaagttaaccatt

gtggtggtggataccattccggtggagctaaaaagttcctatatggtctggagctggttt

atctatgtgctctcagccaatctgctgttagtgatcccgctgctgtgggtcgccgcctgg

tggagtttacgccccatcgaagccctggcaaaagaagtccgcgaactggaagaacataac

cgcgaattgctcaatccagccacaacgcgagaactgaccagtctggtacgaaacctgaac

cgattgttaaaaagcgaacgcgaacgttacgacaaataccgtacaacgctcaccgacctg

acccatagtctgaaaacgccactggcggtgctgcaaagtacgctgcgttctctgcgtagt

gaaaagatgagcgtcagtgatgctgaaccggtaatgctggagcaaatcagccgcatttca

cagcaaattggctactacctgcatcgtgccagtatgcgcggcgggacattgctcagccgc

gagctgcatccggtcgccccactgctggacaatctcacctcggcgctgaacaaagtgtat

caacgcaaaggggtcaatatctctctcgatatttcgccagagatcagctttgtcggtgag

cagaacgattttgtcgaggtgatgggcaatgtgctggataatgcctgtaaatattgcctc

gagtttgtcgaaatttctgcaaggcaaaccgacgagcatctctatattgtggtcgaggat

gatggacccggtattccattaagcaagcgagaggtcattttcgaccgtggtcaacgggtt

gatactttacgccctgggcaaggtgtggggctggcggtagcccgcgaaatcaccgagcaa

tatgagggtaaaatcgtcgccggagagagcatgctgggcggtgcgcggatggaggtgatt

tttggtcgccagcattctgcgccgaaagatgaataaatgcgcgtactggttgttgaagac

aatgcgttgttacgtcaccaccttaaagttcagattcaggatgctggtcatcaggtcgat

gatgcagaagatgccaaagaagccgattattatctcaatgaacatttaccggatattgcg

attgtcgatctcggattgccagacgaggacggtctgtcactgattcgccgctggcgtagc

aatgatgtttcactgccgattctggtattaaccgcccgtgaaagctggcaggacaaagtc

gaagtattaagtgccggtgctgatgattacgtgactaaaccgtttcatattgaagaggtg

atggcgcgaatgcaggcattaatgcggcgtaatagcggcctggcttcacaggtcatttcg

ctccccccgtttcaggttgatctctctcgccgtgaattatctattaatgacgaagtgatc

aaactgaccgcgttcgaatacaccattatggaaacgttgatacgcaataatggcaaagtg

gtgagcaaagattcgttaatgctccaactctatccggatgcggagctgcgggaaagccat

accattgatgtactgatgggacgtctgcgcaaaaaaattcaggcacaatatccccaagaa

gtgattaccaccgttcgcggccagggctatctgttcgaattgcgctgaatggcattactc

gatttctttctctcgcggaagaaaaacacagccaacattgcaaaagaacggctgcagatt

attgttgctgaacgccgtcgcagcgatgcagaaccgcattatctgccgcagttgcgtaaa

gatattcttgaggtcatttgtaaatacgtacaaattgatcctgagatggtaaccgtacag

cttgagcaaaaagatggcgatatttctattcttgagctgaacgtgaccttaccggaagca

gaagagctgaaataaatggcacgcattattgttgttacttcgggcaaagggggtgttggt

aagacaacctccagcgcggccatcgccactggtttggcccagaagggaaagaaaactgtc

gtgatagattttgatatcggcctgcgtaatctcgacctgattatgggttgcgaacgccgg

gtcgtttacgatttcgtcaacgtcattcagggcgatgcaacgctaaatcaggcgttaatt

aaagataagcgtactgaaaatctctatattctgccggcatcgcaaacacgcgataaagat

gccctcacccgtgaaggggtcgctaaagttcttgatgatctgaaagcgatggattttgaa

tttatcgtttgtgactccccggcagggattgaaaccggtgcgttaatggcactctatttt

gcagacgaagccattattaccaccaacccggaagtctcttcagtacgcgactctgaccgt

attttaggcattctggcgtcaaaatcacgccgcgcagaaaatggcgaagagcctattaaa

gagcacctgctgttaacgcgctataacccaggccgcgtaagcagaggtgacatgctgagc

atggaagatgtgctggagatcctgcgcatcaaactcgtcggcgtgatcccagaggatcaa

tcagtattgcgcgcctctaaccagggtgaaccggtcattctcgacattaacgccgatgcg

ggtaaagcctacgcagataccgtagaacgtctgttgggagaagaacgtcctttccgcttc

attgaagaagagaagaaaggcttcctcaaacgcttgttcggaggataaatgtatcaacat

cacaactggcaaggtgcgctgctggattatccggtgagtaaagtagtctgtgttggcagt

aactatgccaaacatattaaagagatgggcagcgcagtgcccgaagagccagtgctgttt

attaaaccagaaacggcactgtgcgatctgcggcagccattggcgatcccatccgatttc

ggttcagttcatcatgaagtcgaactggcggtgttgattggcgcgacgctgcgtcaggct

acggaagagcatgtccgcaaagccattgccggttacggcgtggcgctcgatctgacgttg

cgtgatgttcagggaaaaatgaagaaagccgggcagccgtgggaaaaggctaaagcgttt

gataactcttgtccgctttccgggtttattcccgcggcggaattcaccggcgatccgcaa

aatacaacgctgggcctgagcgtaaacggcgaacaacgccagcaaggtacgactgcggac

atgatccataaaatcgttccgctgatcgcttatatgagcaagttttttaccctcaaggcc

ggtgacgttgtgctgacaggcacgcctgatggcgtcggcccgttgcaaagcggtgatgag

ctgacagtcactttcgatgggcattctttgacaactcgcgttttgtaaatgttgtttatc

aagcctgcggatctccgcgaaattttgacttttccgctatttagcgatcttgttcagtgt

ggctttccttcaccggccgcagattacgttgaacagcgcatcgatctgaatcaactgttg

atccagcatcccagcgcgacttacttcgtcaaagcaagtggtgattctatgattgatggt

ggaattagtgacggtgatttactgattgtcgatagcgctattaccgccagccatggtgat

attgtcatcgctgctgttgacggcgagtttacggtgaaaaaattgcaactacgcccgacg

gtacagcttattcccatgaacagcgcgtactcgcccattaccatcagtagtgaagatacg

ctggatgtctttggtgtggtgatccacgtcgttaaggcgatgcgctgaatgggattcaaa

tgcggtatcgtcggtttgcccaacgtcgggaaatctaccctgttcaacgcgctgaccaaa

gccggtattgaagcggccaactttccattctgcaccattgagccgaacacaggcgtcgta

ccaatgcctgaccctcgcctggatcaactggctgaaatcgtaaaaccgcagcgtacgctt

cccacgaccatggaatttgtcgatatcgccggtctggtaaaaggcgcatcgaaaggcgaa

ggtctgggtaaccagttcctgaccaacatccgtgaaaccgaagcgatcggtcacgttgtt

cgctgctttgaaaatgacaacatcattcacgtttccggcaaagttaacccggctgacgat

attgaagttatcaataccgaactggcgctggcagacctcgacacctgcgaacgtgcgatt

catcgcgtacagaagaaagccaaaggtggcgataaagacgcgaaagctgagctggcggtc

ctggaaaaatgcctgccccagctggaaaacgcaggtatgctgcgcgcgctggatttaagc

gctgaagagaaagcggctattcgttacctgagcttcctgacgctaaaaccaacaatgtac

atcgccaacgtcaacgaagacggttttgaaaacaacccatatcttgaccaggtgcgtgaa

atcgcggcgaaagaaggttctgttgtggttccggtttgtgctgctgttgaagcagacatt

gccgaactggacgacgaagaacgtgacgagtttatgcaggagcttgggcttgaagagccg

ggcctgaaccgtgtgatccgtgccggttataagctgctgaacctgcaaacttacttcacc

gctggggtgaaagaagtgcgtgcatggaccattccggttggagcaaccgcgccgcaggca

gcgggcaaaatccatactgattttgaaaaaggctttatccgtgcacaaaccatctcgttt

gaagatttcatcacttacaaaggtgaacaaggcgcgaaagaagcaggcaaaatgcgtgca

gaaggtaaagattacatcgttaaagatggcgatgtgatgaacttccttttcaacgtctaa

atgaaacgcaaaaacgcttcgttactcggtaacgtgctcatggggttgggtctggtggta

atggtggttggcgtggggtattcaatcctcaaccagttaccacagtttaatatgccccag

tatttcgcacatggtgcagtgctaagtattttcgtcggtgccattctctggctggcgggt

gcccgtgttggcgggcatgaacaggtgtgcgaccgttactggtgggttcgccactatgac

aaacgttgccgccgtagcgataatcgccgtcatagctaaatgcggacacagtggccctct

ccggcaaaacttaatctgtttttatacattaccggtcagcgtgcggatggttaccacacg

ctgcaaacgctgtttcagtttcttgattacggcgacaccatcagcattgagcttcgtgac

gatggggatattcgtctgttaacgcccgttgaaggcgtggaacatgaagataacctgatc

gttcgcgcagcgcggttattgatgaaaactgcggcagacagcgggcgtcttccgacggga

agcggtgcgaatatcagcattgacaagcgtttgccgatgggcggcggtctcggcggtggt

tcatccaatgccgcgacggtcctggtggcattaaatcatctctggcaatgcggactaagc

atggatgagctggcggaaatggggctgacgctgggcgcagatgttcctgtctttgttcgg

gggcatgccgcgtttgccgaaggcgttggtgaaatactaacgccggtggctccgccagag

aagtggtatctggtggcgcaccctggtgtaagtattccgactccggtgatttttaaagat

cctgaactcccgcgcaatacgccaaaaaggtcaatagaaacgttgctaaaatgtgaattc

agcaatgattgcgaggttatcgcaagaaaacgttttcgcgaggttgatgcggtgctttcc

tggctgttagaatacgccccgtcgcgcctgactgggacaggggcctgtgtctttgctgaa

tttgatacagagtctgaagcccgccaggtgctagagcaagccccggaatggctcaatggc

tttgtggcgaaaggcgctaatctttccccattgcacagagccatgctttaaatggaatat

caatactggttacgtgaagcaataaaccaacttcaggcgagcgaaagcccgcggcgtgat

gctgaaatcctgctggagcatgttaccggcagagggcgtacttttattctcgcctttggt

gagacgcagctgactgacgaacaatgtcagcaacttgatgcgctactgacacgtcgtcgc

gatggtgaacccattgctcatttaaccggggtgcgagaattctggtcgttgccgttattt

gtttcgccagcgaccttaattccgcgcccggatacggagtgtctggtggagcaggcactg

gcgcggttgcctgaacaaccttgccgtattctcgatctcgggacgggtaccggggcgatt

gcgcttgcgctggctagcgagcgcccggactgcgaaattatcgctgtagatcgtatgcct

gatgctgtctccctggcacaacgtaatgcccagcatctggcgatcaaaaatatccacatt

ctgcaaagcgactggtttagcgcgctagccgggcagcagtttgcgatgattgtcagcaat

ccgccgtatattgacgagcaggacccacatcttcaacaaggcgatgtccgctttgagccg

ctcactgcgctggttgcggcagacagtggaatggcagacatcgtgcatatcatcgaacag

tcgcgtaacgcgctggtatccggcggctttctgcttctggaacatggctggcagcagggc

gaagcggtgcgacaggcatttatcctcgcggggtatcatgacgtcgaaacctgccgtgac

tatggtgataacgagcgtgtaacgctcggccgctattatcaatgaatgacaagtttttct

acactgcttagtgttcatcttattagtatcgcgctttctgttgggctattaaccttacgt

ttctggctacgttatcagaagcatcctcaggcatttgcgcgctggacgcgcattgtgccg

ccggttgtcgatacggtgttattgttaagcggcattgcgttgatggctaaagcgcacatc

ctgccattttccgggcaggcacagtggctgactgaaaagctgtttggagttatcatttat

atcgttttgggttttattgcactcgattatcgtcgtatgcacagtcagcaggcgcgcatt

attgccttcccgctggcgttggtggtgctgtacatcatcattaaactcgccaccacaaaa

gtaccgttactggggtaaatgcaaaaaatcgtgatcgttgccaatggcgcaccttacggg

agcgaatccttgtttaacagcttgcggctggccattgcgttacgagagcaggagagcaat

ctggatctgcgtctgttcctgatgtctgatgcggtcacagccgggttgcgcgggcaaaaa

ccaggggaaggctacaacattcagcaaatgctggagatccttaccgctcagaatgtaccg

gtgaaattgtgcaaaacctgtaccgacgggcgcgggattagtacacttcctctgattgat

ggggtggaaatcggtactcttgtggaactggcgcaatggacgctgtcagccgataaagtg

ctcacattttaaatgcattcactccaacgtaaagttctgcgtactatttgtccggaccaa

aaaggtctgatcgcacgtattaccaatatttgctacaagcacgagttaaatatcgtacag

aacaatgaatttgttgatcaccgtaccgggcgcttttttatgcgcacggaactggaaggg

atttttaatgattccaccctgctggcggatctctatagcgcattgccagaaggctccgtg

cgtgagctgaatcctgccggtcgtcgccggatagtgattctggtcactaaagaagcgcat

tgccttggcgatttgttgatgaaagccaattatggcggcctggatgtcgaaatcgcggca

gtgattggtaaccacgatactttacgttctctggttgagcgttttgatataccgtttgag

ctggtaagccatgaagggttaagccgcaacgagcacgatcaaaagatggcggatgccatt

gatgcttatcaacctgactacgtggtgctggcgaagtatatgcgggtattaacaccggaa

tttgtgtcacgcttcccgaataagatcatcaatattcaccattccttcctgccagcgttt

atcggcgcacgtccttatcaccaggcctatgaacgtggcgtgaagattattggcgcaacc

gctcactatgtgaatgacaatctggacgaaggcccaatcatcatgcaggacgttattcat

gtcgatcatacctacacagctgaagatatgatgcgcgcaggtcgtgacgtcgagaaaaac

gtcttaagtcgcgcgctctacaaagtactggcgcagcgcgtctttgtttacggtaatcgg

acgattattctttaaatgacgcagccattggtcggaaaacagattctcattgttgaagat

gagcaggtatttcgctcgcttctggattcgtggttttcctcattgggagcgacaacggta

ctggcggctgatggggtggatgcccttgagttgctgggaggtttcactccagacctgatg

atatgtgatatcgcgatgccacgaatgaacgggcttaaactgctggagcatatacgtaac

agaggcgaccagaccccagttctggtgatatctgccactgaaaatatggcagatattgcc

aaagcgttacgtctgggcgttgaagatgttttgctgaaaccagttaaagatctgaatcgc

ttgcgcgagatggtttttgcctgtctctatcccagcatgtttaattcgcgcgttgaggaa

gaggaaaggctttttcgcgactgggatgcaatggttgaaaaccctgccgcagcggcgaaa

ttattacaggaactacaaccgccggttcagcaggtgatttcccattgccgggttaattat

cgtcaattggttgccgcggacaaacccggcctggtgcttgatattgccgcactttcggaa

aacgatctggcattttattgccttgatgtcacccgagctggacataatggcgtacttgct

gccttgttattacgcgcattgtttaacggattattacaggaacagcttgcacaccaaaat

caacggttgccagagttgggcgcgttattgaagcaggtaaaccatttactccgtcaggcc

aatctgccggggcagtttccgctattagttggctattatcatcgcgaactgaaaaatctc

attctggtttctgcgggtctgaatgcgacgttaaataccggcgaacaccaggtgcaaatc

agtaatggtgttccgttaggcactttaggtaacgcttatttgaatcaattgagccagcga

tgcgatgcctggcaatgccaaatatggggaaccggtggtcggctgcgcttgatgttgtct

gcagaatgaatgagcgaagcacttaaaattctgaacaacatccgtactcttcgtgcgcag

gcaagagaatgtacacttgaaacgctggaagaaatgctggaaaaattagaagttgttgtt

aacgaacgtcgcgaagaagaaagcgcggctgctgctgaagttgaagagcgcactcgtaaa

ctgcagcaatatcgcgaaatgctgatcgctgacggtattgacccgaacgagctgctgaat

agccttgccgccgttaaatctggcaccaaagctaaacgtgctcagcgtccggcaaaatat

agctacgttgacgaaaacggcgaaactaaaacctggactggccagggccgtactccagct

gtaatcaaaaaagcgatggatgagcaaggtaaatccctcgacgatttcctgatcaagcaa

taaatgttaaaatttattctacgtcgctgtctggaagcgattccgacgctatttattctt

attactatttcgttctttatgatgcgcctcgcgccgggaagcccttttaccggcgaacgt

actttaccgccagaagtgatggccaatatcgaagcgaaatatcatcttaatgatccaatc

atgacacagtatttcagctacctgaaacaactggcgcacggtgatttcggtccatcgttt

aaatataaagattattcggtcaatgacctggtggcatccagttttcccgtttctgccaaa

ctgggagccgcagcatttttccttgcggtaatactgggtgttagtgctggcgttattgcc

gcattaaaacaaaacaccaaatgggactataccgtgatggggctggcaatgaccggggtt

gttatccccagttttgtggttgcgccattattagtcatgatatttgcgatcattttgcat

tggctgccgggcggtggctggaatggtggggcgcttaaattcatgatattgccgatggtg

gcgttgtcactcgcttatatcgccagtattgcgcgtattacccgtggctctatgattgaa

gtattacactccaactttattcgtactgcccgggcgaaagggttacctatgcggcggatc

attttacgccacgcattaaaacctgctctgttacccgtgctctcctatatgggccctgca

tttgtcggcattattaccggttctatggtcatcgaaaccatttatggtttgccggggatt

gggcaattgttcgttaatggtgcattgaaccgtgactattccttagtgttaagcctgacc

atcctggttggtgctttaaccattttgtttaatgccattgtcgatgtgctatatgcggtt

atcgacccgaaaatccgttactgaatgatgttaagtaagaaaaacagcgagacgctggaa

aatttcagtgaaaagctggaggtcgaagggcgcagcttgtggcaggacgcacgtcgacgt

tttatgcataaccgtgcggcggttgccagtctgatagtgctggtgctgatcacgttattt

gtaatcctggcaccgatgctttcgcagtttgcctatgacgatactgactgggcgatgatg

tccagcgccccggatatggagtccggtcactactttggtactgactcatccggtcgcgac

ctgcttgtgcgcgttgcgattggcgggcgtatctcactcatggtcggtgttgctgcggca

ctggtggcagtggtcgtggggacactttacggttcgctttccggttatctgggcggtaaa

gtggattcggtaatgatgcgtctgctggaaatcctcaactctttcccattcatgttcttc

gtcattttgctggtgacctttttcggccaaaacatcctgctgattttcgtggcgattggc

atggtttcctggctggatatggctcgtattgtgcgtgggcaaaccctgagtctgaagcgc

aaagagtttattgaggcggcacaagttggcggtgtatcgacgccgggcattgttattcgc

cacattgtgccgaacgtactcggtgtggtggtggtctacgcatcgctactggtgcccagc

atgatcctctttgaatctttccttagcttcctggggttgggtacgcaagagccgttaagc

agctggggggcattgctgagtgatggcgcgaactcgatggaagtctctccatggttactg

ttgttcccagcgggattcctcgtggtgacgctgttttgtttcaactttatcggcgatggc

ttgcgtgatgccctcgacccgaaagatcgttaaatgtctacaacacataacgtccctcag

ggcgatcttgttttacgtactttagccatgcccgccgataccaatgccaatggtgacatc

tttggtggttggttaatgtcacaaatggatattggcggcgctattctggcaaaagaaatt

gcccacggtcgcgtagtgactgtgcgggttgaaggaatgactttcttacggccggttgcg

gtcggcgatgtggtgtgctgctatgcacgctgtgtccagaaagggacgacatcggtcagc

attaatattgaagtgtgggtgaaaaaagtagcgtctgaaccaatcgggcaacgctataaa

gcgacagaagcattatttaagtatgtcgcggttgatcctgaaggaaaacctcgcgcctta

cctgttgagtaaatgaagcagtttcttgattttttaccgctggttgtctttttcgcgttt

tacaagatttatgacatctatgcggctactgcggcgctgatcgtcgccacggcgattgtg

cttatatatagctgggttcgctttcgtaaggttgagaagatggccctgatcacttttgtt

ctggtggtcgtcttcggtggcttgacgctgttcttccacaatgatgagtttattaaatgg

aaggttacagtcatttatgccctgtttgcgggtgccctgttagtcagccaatgggtgatg

aaaaagccgctaattcagcggatgctgggtaaagaactcacgctgccgcaaccggtatgg

tcgaagctgaatctggcctgggctgttttctttatcctttgcggtctggcaaacatctac

atcgcattctggctgccgcaaaatatttgggtcaactttaaagtctttggcctgaccgcc

cttaccttaatcttcacattgttaagcggtatctatatctaccgccacatgccgcaggaa

gataaatcctaaatgtctatcacggcgcagtccgtataccgtgacaccggaaatttcttc

cgtaatcaatttatgaccattctgttggtatcgttgctatgtgcgtttatcacagtggtg

ttagggcatgttttctcacccagtgatgcacagcttgcgcagctcaatgacggcgtgccc

gttagcggcagtagtgggttgttcgacctggttcagaatatgtcaccggaacagcaacaa

attttgctgcaggcttcagcggcgtccactttttcaggattaatcggtaacgccattctc

gccggaggcgtaatattaattatccagctggtgtctgcgggtcagagagtcagtgcgctg

cgagctattggtgccagtgcgccgatattgccaaagttatttattctgatttttctgact

acccttttagtacagattggcatcatgctggtggtcgttccgggaattatcatggccatt

ttactggccctggcaccggtgatgttggttcaggacaaaatgggcatttttgcctcgatg

cgtagcagtatgcggctgacttgggcgaatatgcgtctggtggcacccgcagtactgagc

tggttgctggcaaaaacactgttgctgctttttgcctcttcttttgccgcattaaccccg

gaaattggtgccgtactggcgaacaccttgagcaacctgatttcagccgtattgctcatc

tatctgttccgtctgtatatgttgattcgccaataagtgaaatatttactcattttctta

ctggtgttagcgatcttcgtgatttcggtcacgttgggtgcgcagaacgatcaacaggtg

acgtttaattatctgttagcgcaaggggagtaccgtatttccacattgctggcggtattg

tttgctgcggggtttgctatcggttggttgatttgtggcctgttctggctgcgagttcgt

gtttccctggcgcgcgctgaacgtaaaataaagcgactggaaaaccagctttcacccgcg

actgacgtggctgtagtgcagcactcgtcagcggcgaaggaataaatgaattcccgacaa

caaactattctacagatggtcattgaccagggtcaggttagcgtaaccgatctggcaaaa

gccactggagtttctgaagttaccattcgccaggatctcaacaccctcgaaaaactgagt

tacctccgccgtgcacatggctttgcagtttcgcttgatagtgatgacgtcgaaacccgt

atgatgagcaattatacgctgaagcgtgaactcgccgagtttgccgcgtcactggttcaa

ccgggcgaaaccatctttatcgaaaatggcagcagcaatgccctgctcgctcggactctg

ggcgagcagaagaaaaatgtcactatcatcacggtcagcagctacatcgcgcatttgctg

aaagacgcgccttgtgaagttattttgctcggtggcgtgtaccagaaaaaaagcgaaagt

atggttggccctttgacacgccagtgcatccaacaggtgcatttcagcaaagcatttatt

ggtattgatggctggcaacctgaaactggatttaccggtcgcgatatgatgcgtaccgat

gtggtcaatgccgtgctggaaaaagagtgcgaagcgatagtcctgactgacagctcgaaa

tttggtgctgtacattcatactccatcggtcccgttgagcgattcaatcgcgtgattacc

gattcgaaaatacgcgccagcgatctgatgcatcttgagcacagtaaactcaccgttcac

gtcgttgacatttaaatgggtattttttctcgctttgccgacatcgtgaatgccaacatc

aacgctctgttagagaaagcggaagatccacagaaactggtgcgtctgatgatccaggag

atggaagatacattggttgaagtacgttctacttcggcgcgtgcgctggcagaaaagaaa

cagctgactcgccgtattgaacaagcgtcggcgcgtgaagttgaatggcaggaaaaagcc

gaactggcgctgctgaaagagagagaggatttggcgcgtgcggcgttaattgaaaaacag

aaactgaccgatctgattaagtccctggaacatgaagtgacgctggtggacgatacgctg

gcacgcatgaagaaagagatcggtgagctggaaaacaaattgagcgaaacacgcgctcgc

cagcaggcattgatgttacgccatcaggcggcaaactcgtcgcgcgatgtgcgtcgtcag

ctggacagtggcaaactggatgaagcaatggctcgtttcgaatctttcgaacgtcgaatt

gaccagatggaagcggaagcagaaagccacagcttcggtaaacaaaaatcgctggacgat

cagtttgccgaactgaaagccgatgatgcaataagcgagcaactggcacaattaaaagcc

aaaatgaagcaagacaatcaataaatgtcacaaaccgttcatttccagggcaacccggtt

acagtcgccaattccatcccacaggcgggtagcaaagcgcagacttttactctcgtggca

aaagatctgtctgacgtcaccctcggtcagtttgcgggtaaacgcaaagtgctgaacatt

ttcccgagtattgataccggtgtttgcgccgcatcagtacgtaagtttaaccaactggca

accgagatcgacaacaccgttgtgctgtgtatttctgccgatctgccgttcgcccagtct

cgtttctgcggcgcagaaggtctgaacaacgttatcaccctctccactttccgtaacgct

gaattcctgcaagcctacggtgtggcaattgctgatggcccactgaaaggtctggcagcg

cgtgccgttgtggttattgatgaaaatgacaatgtgattttcagccagctggtggatgaa

atcaccaccgagccggattacgaagcagctctggctgtactgaaagcataagtggaagcg

attaagggatcggacgttaatgtcccggatgcagtatttgcctggatgctggatggtaga

ggcggcgttaaaccgctggaaaatacagatgtgattgatgaagcgcatccctgctggctc

caccttaattatgtacaccatgatagcgcccaatggctggcgacaacaccgctgcttccc

aataacgtacgtgatgcgctggcaggtgagagcacacggccccgagtcagccgtctcggt

gaaggcacgctgattacattgcgctgtataaacggcagcaccgatgaacgccccgatcaa

ctggtcgccatgcgtgtatatatggacgggcggttaattgtttcgacccgacaacgcaaa

gtgttggcgctggacgatgtggtgagcgatctggaagagggcacgggtccgaccgattgc

ggagggtggctggtggatgtgtgcgatgcgttgaccgatcattccagcgaatttatcgag

cagctgcacgataaaattatcgaccttgaagataatctccttgatcagcaaattccgccg

cgtggattcctggctctgctgcgcaaacaattaattgtgatgcgtcgctatatggcaccg

caacgtgatgtctatgctcgtcttgccagtgaacgtttgccgtggatgagcgatgaccaa

cgccgtcggatgcaggatattgccgatcgccttgggcgcggccttgacgaaatcgacgcc

tgtatagcacggactggtgtgatggcggatgaaatcgctcaggtgatgcaggaaaattta

gctcgtcgtacctatacaatgtcattgatggcaatggtctttttacccagtacctttctg

acagggttatttggcgtcaaccttggtgggatccctggcggcgggtggcaattcggattt

tcaattttttgtattctgttagttgttcttattggtggtgttgctttatggttgcatcgt

agtaaatggttgtaaatgaaactcgccgtttatagcacaaaacagtacgacaagaagtac

ctgcaacaggtgaacgagtcctttggctttgagctggaattttttgactttctgctgacg

gaaaaaaccgctaaaactgccaatggctgcgaagcggtatgtattttcgtaaacgatgac

ggcagccgcccggtgctggaagagctgaaaaagcacggcgttaaatatatcgccctgcgc

tgtgccggtttcaataacgtcgaccttgacgcggcaaaagaactggggctgaaagtagtc

cgtgttccagcctatgatccagaggccgttgctgaacacgccatcggtatgatgatgacg

ctgaaccgccgtattcaccgcgcgtatcagcgtacccgtgacgctaacttctctctggaa

ggtctgaccggctttactatgtatggcaaaacggcaggcgttatcggtaccggtaaaatc

ggtgtggcgatgctgcgcattctgaaaggttttggtatgcgtctgctggcgttcgatccg

tatccaagtgcagcggcgctggaactcggtgtggagtatgtcgatctgccaaccctgttc

tctgaatcagacgttatctctctgcactgcccgctgacaccggaaaactaccatctgttg

aacgaagccgccttcgatcagatgaaaaatggcgtgatgatcgtcaataccagtcgcggt

gcattgattgattctcaggcagcaattgaagcgctgaaaaatcagaaaattggttcgttg

ggtatggacgtgtatgagaacgaacgcgatctgttctttgaagataaatccaacgacgtg

atccaggatgacgtattccgtcgcctgtctgcctgccacaacgtgctgtttaccgggcac

caggcattcctgacagcagaagctctgaccagtatttctcagactacgctgcaaaactta

agcaatctggaaaaaggtgaaacctgcccgaacgaactggtttaaatgtctggaggatta

gttacagctgcatacattgttgccgcgatcctgtttatcttcagtctggccgggctttcg

aaacatgaaacgtctcgccagggtaacaacttcggtatcgccgggatggcgattgcgcta

atcgccaccatttttggaccggatacgggtaacgttggctggatcttgctggcgatggtc

attggtggggcaattggtatccgtctggcgaagaaagttgaaatgaccgaaatgccagaa

ctggtggcgatcctgcatagcttcgtgggtctggcggcagtgctggttggctttaacagc

tatctgcatcatgacgcgggaatggcaccgattctggtcaatattcacctgacggaagtg

ttcctcggtatcttcatcggggcggtaacgttcacgggttcggtggtggcgttcggcaaa

ctgtgtggcaagatttcgtctaaaccgttgatgctgccaaaccgtcataaaatgaacctg

gcggctctggtcgtttccttcctgctgctgattgtatttgttcgcacagacagcgtcggc

ctgcaagtgctggcattgctgataatgaccgccattgcgctggtattcggctggcattta

gtcgcctccatcggtggtgctgatatgccagtggtggtgtcaatgctgaactcgtactcc

ggctgggcggctgcggctgcgggctttatgctcagcaacgacctgctgattgtgaccggt

gcgctggtcggttcttcgggcgctatcctttcttacattatgtgtaaggcgatgaaccgt

tcctttatcagcgttattgcgggtggtttcggcaccgacggctcttctactggcgatgat

caggaagtgggtgagcaccgcgaaatcaccgcagaagagacagcggaactgctgaaaaac

tcccattcagtgatcattactccggggtacggcatggcagtcgcgcaggcgcaatatcct

gtcgctgaaattaccgagaaactgcgcgctcgtggtatcaacgtgcgtttcggtatccac

ccggttgcggggcgtttgcctggacatatgaacgtattgctggctgaagcaaaagtaccg

tatgacatcgtgctggaaatggacgagatcaacgatgactttgctgataccgataccgta

ctggtgattggtgctaacgatacggttaacccggcggcgcaggatgatccgaagagtcct

attgctggtatgcctgtgctggaagtgtggaaagcgcagaacgtgattgtctttaaacgt

tcgatgaacactggctatgctggtgtgcaaaacccgttgttcttcaaggaaaacacccac

atgctgtttggtgacgccaaagccagcgtggatgcaatcctgaaagctctgtaaatgggt

aaaacccagcccttgccaatattaattactggcggaggtcgtcgcatcggcctcgccctc

gcatggcatttcattaatcaaaagcaaccggtgattgtcagctatcggacacactatcca

gccattgatggactgattaatgcaggtgcgcagtgtattcaggctgatttttcgaccaac

gacggtgtgatggcgtttgccgatgaagtactaaaaagcacccatggtctgcgtgctatt

ttgcataacgccagtgcgtggatggcggaaaaaccgggtgcgccactggccgacgtactg

gcttgcatgatgcagatccacgttaataccccatacctgctcaaccatgcgctggaaaga

ttactgcgtgggcacggacacgccgccagcgatatcattcactttaccgattatgtggtg

gagcgcggtagcgacaaacatattgcgtatgctgcaagcaaagcggcactggataatatg

acccgctcgtttgcccgcaagctggcaccggaagtgaaagtgaattctattgcgccatcg

ctgatcctgtttaatgaacatgatgatgccgaatatcgacaacaggcgctgaataaatca

ctgatgaaaaccgcgcctggcgagaaagaagtgatcgacctggtcgattacttacttacc

agctgctttgtcaccggacgcagtttcccacttgatggcggtcgtcatctgcgttaaatg

gctaccgccaaaaaaataaccattcatgatgttgcgctggctgcgggcgtgtcggtaagt

accgtttcgctggtgcttagtggcaaagggcgaatctctaccgccacaggagaacgcgtt

aacgccgccattgaagagctgggatttgtgcgcaatcgccaggcgtcggcgctgcgcggc

gggcaaagcggcgtcattggtttgatcgtccgtgatttatctgcgccgttttacgccgaa

ttgacggccggattgacggaagctctggaagcgcagggacggatggtttttttgcttcac

ggcggtaaagacggcgagcagctggcacagcggttttcactgttactgaatcagggtgtc

gatggtgtggtaattgccggggctgcaggaagcagcgatgacctgcgacggatggcagaa

gaaaaagctatcccggtgattttcgcttcccgtgccagttatcttgatgatgttgatacg

gttcgcccggacaacatgcaggctgcacagttgttgacggagcatctcattcgcaatggg

catcagcggatcgcctggctgggagggcaaagttcctcattaacccgtgcagaacgggtg

gggggctattgtgcaactctactaaaatttggccttccgtttcacagcgattgggtgttg

gagtgcacttccagccagaagcaagccgcggaagctatcacggcgcttttacgtcataac

ccgaccatcagtgccgtggtttgctataacgaaactattgcgatgggggcatggtttggt

ttgctgaaagcagggcggcaaagcggggaaagcggagtcgatcgttactttgagcaacag

gtttcgctggcggcatttaccgatgcgacaccaaccacacttgatgatatacccgttacc

tgggccagcacgcctgcgcgggaacttggtaccacacttgcggatcgcatgatgcaaaaa

atcacccatgaagagacgcattcacgcaatcttattattcccgcccggctcattgcggcg

aaataaatgattgataccaccctgccattaactgatatccatcgccaccttgatggcaac

attcgtccccagaccattcttgaacttggccgccagtataatatctcgcttcctgcacaa

tccctggaaacattgattccccacgttcaggtcattgccaacgaacccgatctggtgagc

tttctgaccaaacttgactggggcgttaaagttctcgcctctcttgatgcctgccgccgc

gtggcatttgaaaacattgaagatgcagcccgtcacggcctgcactatgtcgagctgcgt

ttttcaccaggctacatggcaatggcacataagctgcctgtagcgggtgttgtcgaagcg

gtgatcgatggcgtacgtgaaggttgccgcacctttggtgtgcaggcgaagcttatcggc

attatgagccgaaccttcggcgaagccgcctgtcagcaagagctggaggcctttttagcc

caccgtgaccagattaccgcacttgatttagccggtgatgaacttggtttcccgggaagt

ctgttcctttctcacttcaaccttgcgcgtgatgcgggctggcatattaccgtccatgca

ggcgaagccgctgggccggaaagcatctggcaggcgattcgtgaactgggtgcggagcgt

attggacatggcgtaaaagccattgaagatcgggtgctgatggattttctcgccgagcaa

caaattggtattgaatcctgtctgacctccaatattcagaccagcaccgtagcagagctg

gctgcacatccgctgaaaacgttccttgagcatggcattcgtgccagcattaacactgac

gatcccggcgtacagggagtggatatcattcacgaatataccgttgccgcgccagctgct

gggttatcccgcgagcaaatccgccaggcacagattaatggtcttgaaatggctttcctc

agcgcagaggaaaaacgcgcactgcgagaaaaagtcgctgcgaagtaaatgactgactac

ctgttactgtttgtcggaactgtactggtcaataactttgtactggtcaagtttctcggt

ctctgtccgtttatgggggtttccaaaaagctggaaaccgcgatgggcatggggctggca

acaacgtttgtgatgacgctggcgtctatttgcgcctggcttatcgatacgtggattttg

atcccacttaatctgatttacctgcgcaccctggcatttattctggtgattgctgtggtc

gtgcagttcaccgagatggtggtgcgcaaaaccagcccggtgctttaccgtttgctgggg

atttttttgccgcttatcaccaccaactgtgccgtgctcggcgtggcgttgctgaatatc

aatctcgggcacaatttcttgcagtcggcgctgtacggtttttccgccgctgtcggtttt

tcgctggtgatggtgctcttcgcagccatccgcgaacgccttgctgtggctgatgtcccg

gctccttttcgcggtaatgccattgcgttaattaccgcaggtcttatgtctctggccttt

atgggctttagtggtttggtgaagttgtaaatgaatgctatctggattgccgttgccgcc

gtgagcctgctgggcctggcgtttggcgccattctgggttatgcctcccgccgttttgcg

gtggaagacgatccggtcgttgaaaaaattgacgaaatcttaccgcagagccagtgtggt

cagtgcggttatcccggctgtcgcccctacgcggaagccatcagctgtaacggtgaaaaa

atcaaccgttgcgcccctggtggcgaagctgtgatgctaaaaattgccgagttgcttaat

gtcgaaccgcagccgctggatggcgaagcgcaagagctaacgcctgcgcggatggtggcg

gttattgatgaaaataactgtattggctgcactaaatgtattcaggcgtgtccggtagac

gccatcgttggcgctacccgagccatgcatacggtaatgagtgaactctgtacgggctgc

aatttatgtgttgatccgtgcccgacgcactgcatctcgttgcaaccggtcgcagaaaca

cctgactcctggaaatgggatctgaacaccattcccgtgcgtatcattcccgtggaacac

catgcttaaatggtattcagaatagctagctccccttatacccataaccagcgccagaca

tcgcgcattatgctgttggtgttgctcgcagccgtgccaggaatcgcagcgcaactgtgg

ttttttggttggggtactctcgttcagatcctgttggcgtcggtcagtgctctgttagcc

gaagctctcgtactcaaactacgcaaacagtcggtagccgccacgttgaaagataactca

gcattgctgacaggcttattgctggcggtaagtattccccccctcgcgccatggtggatg

gtcgtgctgggtacggtgtttgcggtgattatcgctaaacagttatatggcggtctggga

caaaacccgtttaatccggcaatgattggttatgtggtcttactgatctccttcccggtg

cagatgaccagctggttaccgccacatgaaattgcggtcaacctccctggttttatcgac

gccatacaggttattttcagcgggcataccgccagtggtggtgatatgaacacactacgc

ttaggtattgatggcattagtcaggcgacaccgctggatacatttaaaacctctgtccgt

gccggtcattcggttgaacagattatgcaatatccgatctacagcggtattctggcgggc

gctggttggcaatgggtaaatctcgcctggctggctggcggcgtgtggttgctatggcag

aaagcgattcgctggcatattcccctcagcttcttagtaacgctgacgttatgcgcaacg

ttgggctggttgttctcaccagaaacactggcagcaccgcaaattcatctgctgtctgga

gcgaccatgctcggcgcattctttattttgactgacccggttaccgcttctacgaccaat

cgtggtcgtcttattttcggcgcgcttgcgggcttattagtctggttgatccgcagtttc

ggcggctatcctgacggcgtggcttttgccgtcctgctggcgaacatcacggttcctctg

atcgattactacacgcgtccgcgcgtctacggccatcgcaaagggtaaatgctgaaaact

atccgaaaacacggcattacgttggcgctatttgcagcgggttcaacagggttaactgcg

gccatcaaccagatgaccaaaacgacgattgctgaacaggccagtctgcaacaaaaggct

ttatttgatcaggtgctgccagccgaacgctataacaatgcgctggcacagagttgctat

ctggtaactgcgccagagttaggtaaaggtgagcatcgggtttacatcgccaaacaggat

gacaaaccggtagccgccgttctggaagcaaccgcgccagatggctattccggtgcgatt

cagctgctggtgggagccgattttaacggcacggtacttggcacgcgcgtgacagagcac

cacgaaacgccagggcttggcgataaaatcgaactgcgcctttctgactggatcacccat

tttgcgggtaaaaaaatcagtggtgcggatgatgcgcactgggcggtgaagaaagatggt

ggtgatttcgaccagttcaccggcgcaacgattactccccgcgcggtggttaatgcggta

aaacgcgccggattgtacgctcagacgttaccggcacaactttctcaacttcctgcctgt

ggagaataagtgagcgaaattaaagacgttattgttcaggggttgtggaaaaacaactct

gcgctggtccagttgctcggcctttgtcctctgttggcggtcacgtccactgccactaac

gctctgggtttaggacttgcgactacgctggtactgacgctgaccaacctgaccatttcg

acgctgcgtcactggacgccagccgagatccgcattcccatttacgtgatgatcatcgcc

tcggtggtcagcgctgtacagatgctgatcaacgcctacgcctttggcctgtatcaatca

ttagggatttttattccgctgattgtcactaactgtatcgttgtgggccgcgctgaagcc

ttcgccgccaaaaaaggtccggcgctttcggcactggacggcttttcaattggtatgggc

gcaacctgcgccatgttcgtgctgggttcactacgcgaaattatcggcaatggcacattg

tttgacggtgcagatgcgctgttaggtagctgggcaaaagtattacgcgtggagattttc

cacaccgactcccctttcctgctggcgatgctgccaccaggtgcatttattggcctggga

ctgatgctggcaggaaaatacctgattgatgaaagaatgaaaaagcgccgtactgaagca

gctgccgaacgtgcattgccaaacggtgaaacagggaatgtctgaatgaacaaagcaaaa

cgcctggagatcctcactcgcctgcgtgagaacaatcctcatcccaccaccgagcttaat

ttcagttcgccttttgaattgctgattgccgtactgctttccgctcaggcgaccgatgtc

agtgttaataaggcgacggcgaaactctacccggtggcgaatacgcctgcagcgatgctt

gaactgggcgttgaaggggtgaaaacctatatcaaaacgattgggctttataacagcaaa

gcagaaaatatcatcaaaacctgccgtatcttactggagcagcataatggcgaggttccg

gaagatcgtgctgcgcttgaagccctgcccggcgtaggtcgtaaaacagccaacgtcgta

ttaaacactgcattcggctggccgactattgctgtcgacacgcacattttccgcgtttgt

aatcgtactcaatttgcgccggggaaaaacgtcgaacaggtagaagaaaagctactgaaa

gtggttccggcagagtttaaagtcgactgccaccattggttgatcctgcacgggcgttat

acctgcattgcccgcaagccccgctgtggctcttgtattattgaagatctttgtgaatac

aaagagaaagttgacatctgaatgtctgataacgacgaattgcagcaaatcgcgcatctg

cgccgtgaatacaccaaaggcgggttacgccgccgcgatcttcctgccgaaccattaacc

ctttttgaacgctggctctctcaggcttgtgaagccaaactggcggaccctaccgcgatg

gtggtcgctaccgtggatgaacatggtcagccttatcagcgcatcgttttactcaaacat

tacgacgaaaaaggcatggtgttttacaccaacctcggcagccgtaaagcacatcaaatc

gaaaataatccgcgcgttagcctgctgttcccgtggcatacccttgagcgccaggtgatg

gtgatcggtaaagcggaacgactttcgactctcgaagtgatgaaatattttcatagccgc

ccgcgtgatagccagattggtgcatgggtttcgaagcagtccagtcgcatttctgcccgc

ggtatccttgaaagtaaattcctggagctgaagcagaagtttcaacagggcgaagtgcca

ttgccgagcttttggggcggttttcgcgtcagccttgaacagattgagttctggcagggg

ggtgagcatcgcctgcatgaccgctttttgtaccagcgtgaaaatgatgcgtggaagatt

gatcgtcttgcaccctgaatgattaaacgcgtattggttgtttcaatggtaggtctgtct

cttgtcggttgtgttaataacgacaccctgtcaggggatgtttataccgcttctgaagcg

aaacaagtacagaatgtcagctatggcaccatcgttaacgtacgtccggtacagattcag

ggcggtgatgattccaacgtgatcggtgcaattggcggtgctgttcttggtggtttcctg

ggaaatactgttggtggcggaaccgggcgttctctggctactgcagcaggcgctgttgca

ggtggcgtagccggtcagggcgtacagagtgcaatgaacaaaacgcagggtgtcgagctg

gaaattcgtaaagacgatggtaataccatcatggtggtacagaaacaaggcaactctcgt

ttctctccgggccaacgtgtcgtactggccagcaatggcagtcaggtgaccgtttctccg

cgctaaatgcgtcttcttcataccatgctgcgcgttggcgatttgcaacgctccatcgat

ttttataccaaagttctgggcatgaaactgctgcgtaccagcgaaaacccggaatacaaa

tactcactggcgtttgttggctacggcccggaaaccgaagaagcggtgattgaactgacc

tacaactggggcgtggataaatacgaactcggcactgcttatggtcacatcgcgcttagc

gtagataatgccgctgaagcgtgcgaaaaaatccgtcaaaacgggggtaacgtgacccgt

gaagcgggtccggtaaaaggcggtactacggttatcgcgtttgtggaagatccggacggt

tacaaaattgagttaatcgaagagaaagacgccggtcgcggtctgggcaactaaatgtcc

gataacgctcaacttaccggtctgtgcgaccgttttcgtggtttttatcctgttgtgatc

gatgttgaaacagctggatttaacgccaaaaccgatgcgctgcttgagattgccgccatc

accctgaaaatggatgaacaaggctggctgatgccggacaccacattacatttccacgtc

gaaccatttgtcggcgcaaatttgcagccagaggccctcgccttcaacggcattgacccg

aacgatcccgatcgcggcgcggtcagcgaatacgaggcgctgcacgaaatttttaaagtt

gtacgtaaaggtattaaagcgagcggctgtaaccgcgccattatggtggcacacaacgcc

aatttcgatcacagctttatgatggccgccgctgaacgcgcctcactgaaacgtaacccg

ttccaccctttcgccacttttgacactgctgcactggccgggctggcactcggacaaacc

gtattgtcaaaggcttgccagaccgctggcatggacttcgacagcacccaggcgcactcc

gcgctgtacgacaccgaacgcactgcggtgctgttttgtgaaatcgtcaaccgctggaaa

cgtctgggaggctggccgctacctgccgccgaagaggtgtaaatgagcaccactatcgaa

aaaatccaacgccagattgctgaaaacccgatcctgctgtacatgaaaggttcaccgaaa

ctgccgagctgcggtttctctgcccaggcagtccaggcgcttgccgcatgtggcgaacgt

tttgcctatgttgatattctgcagaatccggacattcgtgcggaactgccgaaatatgct

aactggccgaccttcccgcaactgtgggttgacggcgagctggtcggcggttgtgatatc

gtgatcgaaatgtatcagcgtggcgaactgcagcagctgatcaaagaaactgccgctaaa

tacaagtctgaagagccggacgcggaataaatgtcattcgaattacctgcactaccatat

gctaaagatgctctggcaccgcatatttctgcggaaactatcgagtatcactacggcaag

catcatcagacttatgtcactaacctgaacaacctgattaaaggtaccgcgtttgaaggt

aaatcactggaagagattattcgcagctctgaaggtggcgtattcaacaacgcagctcag

gtctggaaccatactttctactggaactgcctggcaccgaacgccggtggcgaaccgact

ggaaaagtcgctgaagctatcgccgcatcttttggcagctttgccgatttcaaagctcag

tttactgatgcagcgatcaaaaactttggttctggctggacctggctggtgaaaaacagc

gatggcaaactggctatcgtttcaacctctaacgcgggtactccactgaccaccgatgcg

actccgctgctgaccgttgatgtctgggaacacgcatattacatcgattatcgcaatgca

cgtcctggttatctggagcacttctgggcgctggtgaactgggaattcgtagcgaaaaat

ctcgctgcataaatgcaacctgggaaaagatttttagtctggctggcgggtttgagcata

ctcggttttctggcaaccgatatgtatctgcctgctttcgccgccatacaggccgacctg

caaacgcctgcgtctgctgtcagtgccagccttagtctgttccttgccggttttgccgca

gcccagcttctgtgggggccgctctccgaccgttatggtcgtaaaccggtattattaatc

ggcctgacaatttttgcgttaggtagtctggggatgctgtgggtagaaaacgccgctatg

ctgctggtattgcgttttgtacaggctgtgggtgtctgcgccgcggcggttatctggcaa

gcgttagtaacggattattatccttcacagaaagttaaccgaatttttgcgaccatcatg

ccgctggtgggtctgtctccggccctggctcctctgttaggaagctggctgctggtccat

ttttcctggcaggcgattttcgccaccctgtttgccattaccgttgtgctgattctgcct

attttctggctcaaacccacgacgaaggcccgtaacaatagtcaggatggtctgaccttt

accgacctgctacgttctaaaacctatcgcggcaacgtgctgatatacgcagcctgttca

gccagtttttttgcatggctgaccggttcaccgttcatccttagtgaaatgggctacagc

ccggcagttattggtttaagttatgtcccgcaaactatcgcgtttctgattggtggttat

ggctgtcgcgccgcgctgcagaaatggcaaggcaagcagttattaccgtggttgctggtg

ctgtttgctgtcagcgtcattgcgacctgggctgcgggcttcattagccatgtgtcgctg

gtcgaaatcctgatcccattctgtgtgatggcgattgctaatggcgcgatctacccgatt

gttgtcgcccaggcgctgcgtcccttcccacacgcaactggtcgcgccgcagcgttgcag

aacactcttcaactgggtctgtgcttcctcgcaagtctggtagtttcctggctgatcagt

atcagcacgccattgctcaccaccaccagcgtgatgttatcaacagtagtgctggtcgcg

ctgggttacatgatgcaacgttgtaaagaagttggctgccagaatcatggcaatgccgaa

gtcgctcatagcgaatcacactgaatggctttattgccggataaagaaaaattgctgcgt

aattttttacgctgcgccaactgggaagagaaatatctctacattattgagctgggccag

cgtctgccagaattacgcgacgaagacagaagcccacaaaatagcattcagggttgccag

agtcaggtgtggattgtcatgcgccagaatgcgcagggaattattgaattgcaaggcgac

agcgatgcggcgattgtaaaagggcttattgcggtcgtatttattctctacgatcagatg

acgccgcaggatattgtcaatttcgatgtgcgtccgtggtttgaaaaaatggcgctcacc

caacatctcaccccatctcgttcacaaggtctggaagcgatgattcgcgcaattcgcgcc

aaagccgctgcacttagctaaatgatatggaaacgaaaaatcaccctggaagcactgaat

gccatgggtgaaggaaacatggtggggttactggatattcgctttgaacatattggtgat

gacacccttgaagcgacaatgccagtagactcgcggacaaagcagcctttcgggttgcta

catggaggtgcatccgtggtactggccgaaagtatcggttccgttgccggttatttatgt

accgaaggtgagcaaaaagtggttggtctggaaatcaatgctaaccacgtccgctcggca

cgagaagggcgggtacgcggcgtatgcaaaccgttgcatctcggttcgcgtcaccaggtc

tggcagattgaaatcttcgatgagaaagggcgtttgtgctgttcgtcacgattgacgacc

gccattttgtgaatggataatgctgttgatcgccacgttttttatatttctgatggtacg

gcaataactgcggaggtattaggacacgcagtaatgtcacaatttcccgtcactatcagc

agcatcacgctgccgtttgtcgaaaatgagagccgtgcacgggcagtgaaggatcagatt

gacgcaatttatcaccagacaggcgtgcgcccgctggtcttctactccatcgtgttgccg

gagattcgcgccatcatcttgcaaagtgaaggcttttgccaggatatcgttcaggcgctg

gttgccccgctacaacaagagatgaaactggatccaacgccgattgctcatcgtacccat

ggccttaaccctaataatctcaataaatatgatgcgcgcattgcggcgattgattacacc

ctcgcccacgatgacggcatttcgttgcgcaatctggatcaggctcaggtgatcctgctc

ggtgtttctcgctgtggtaaaacccccaccagtctgtatctggcaatgcaatttggtatc

cgcgcggcaaactacccctttattgccgacgatatggataatctggtgctacccgcgtcg

ctcaaaccgcttcagcataaattgttcggcctgactatcgacccggaacgtctggcggcg

attcgcgaggaacgtcgggagaacagtcgctatgcctcgcttcgtcagtgcaggatggaa

gtcgcggaagtggaagccttgtaccgtaaaaatcagatcccgtggattaacagtaccaat

tattcggtagaagagattgccaccaagatcctcgatatcatgggccttagtcgccgaatg

tactagatgcgtttctgccttattttgatcacagcactgtttctggccgggtgtagccac

cataaagcaccgccgccaaatgccagactttctgattcgattaccgttattgccggtttg

aacgaccagctacaaagctggcatggcacgccgtatcgttatggtggcatgacgcggcgc

ggtgtggactgttcgggatttgtggttgtgacgatgcgcgatcgtttcgatttgcagctg

ccccgagaaaccaaagaacaagcctctatcggcacgcaaattgataaagacgagttgctg

cctggtgacctggtctttttcaaaacgggttccggacaaaatggtttgcatgtaggtatt

tacgataccaacaaccaatttatccacgcctctaccagcaagggagtgatgcgttcctca

cttgataatgtctattggcagaaaaatttctggcaggcgagacgaatctagatggccagt

ggcgatcttgtccgttatgtcataaccgtcatgttgcatgaggatacattgactgaaatt

aacgagttgaataattacctgactcgcgacggttttttgctcaccatgacggatgatgag

ggaaatatccatgagctggggactaacacttttggacttatcagtacccaaagtgaagaa

gaaattagagaactggtttcggggcttacccaaagtgcaaccggcaaagatcctgaaatc

accatcacgacctgggaggaatggaatagcaacagaaaataaatgtggcaggcaatcagt

cgtcttttgagcgagcagttaggtgaaggcgaaatcgaactgcgtaatgaactgcctggc

ggagaagtccatgccgcatggcatttgcgctatgcaggacatgactttttcgtcaaatgt

gatgaaagggaactgcttcccggttttaccgccgaagccgaccaactggagttactgtcg

cgtagtaaaaccgtcaccgtgcctaaggtttgggcagtaggcgctgaccgtgactacagt

tttctggtgatggattatctcccacctcgtccgctggatgcgcatagcgcatttattctt

ggtcagcaaattgcgcgtttacatcaatggagtgaccaaccacaatttggcctcgatttc

gataacgcgctctccacaactccacagcccaacacctggcaacgtcgctggtcaacgttt

tttgctgaacaacggattggctggcagttggaactggcagcagagaaagggatcgctttc

ggcaatatcgacgccatcgtcgagcatattcagcagcgtctcgcctcacatcaaccgcag

ccttctctgttgcacggcgatttatggtccggcaactgtgcgctgggtccggatggcccg

tacattttcgacccggcctgctactggggtgaccgagagtgcgacctggcgatgttaccg

ctgcatactgaacagccgccacaaatctatgacggctatcagtcagtatccccgctacct

gccgatttccttgaacgtcaaccggtttaccaactctacacgctgttaaatcgtgcaagg

ttatttggcggtcagcatttggttattgctcagcagtcattggatagattattagcagca

tgaatgacgtatcaacaagctggacgcattgctgttttgaaaaggattttgggttgggtg

atttttatacctgcattgatctctacattgatttctttgctgaaatttatgaatacccgg

caggaaaaccaggaaggcattaatgcggtcatgctcgactttactcatgtcatgatcgat

atgatgcaggcgaatacgccttttttgaatctattctggtataactccccgacgcccaat

tttaatggtggcgtgaacgttatgttctgggtgattttcatcctgatttttgtcggactg

gcgttgcaggattccggtgcccggatgagtcgccaggcgcgcttcttgcgggaaggtgtt

gaagatcaactcattctggaaaaagccaaaggggaagaggggctaacgcgtgaacaaatt

gagtcccgtattgttgttccgcaccatactattttcctgcagtttttctcactgtatatc

ctgccggttatttgtattgctgcaggttatgtgttcttttctctgcttgggtttatttaa

atgtcaaccccgcgtcagattcttgctgcaatttttgatatggatggattacttatcgac

tcagaacctttatgggatcgagccgaactggatgtgatggcaagcctgggggtggatatc

tcccgtcgtaacgagctgccggataccttaggtttacgcatcgatatggtggtcgatctt

tggtacgcccggcaaccgtggaatgggccaagccgtcaggaagtagtagaacgggttatt

gcccgtgccatttcactggttgaagaaacacgtccattattaccaggcgtgcgcgaagcc

gttgcgttatgcaaagaacaaggtttattggtgggactggcctccgcgtcaccactacat

atgctggaaaaagtgttgaccatgtttgacttacgcgacagtttcgatgccctcgcctcg

gctgaaaaactgccttacagcaagccgcatccgcaagtatatctcgactgcgcagcaaaa

ctgggcgttgacccactgacctgcgtggcgctggaagactcggtaaatggcatgatcgct

tctaaagcagcccgcatgcgttccatcgtcgttcctgcgccagaagcgcaaaatgatcca

cgttttgtattagcaaacgtcaaactctcatcgctgacagaactcaccgcaaaagacctt

ctcggttaaatgaacaagaatatggcaggaattctgagtgcagcggcggtattaaccatg

ctggcgggttgtacggcttatgatcgtaccaaagaccagtttgtacagcctgtggtgaaa

gacgtcaaaaaaggcatgagccgggcgcaggttgcacaaattgcgggtaaaccttcgtct

gaagtgagcatgatccatgctcgtggtacttgccagacctacatcctgggtcaacgtgat

ggtaaagcagaaacctactttgtcgcgttagatgataccggacatgtcatcaactccggt

tatcagacctgtgctgaatacgacactgatccacaggctgcgaagtaagtgaccaaactc

aaacttctggcacttggagtgcttatcgcaacgtctgcaggcgtagcgcacgctgaaggt

aaattttccctgggcgcaggcgtaggtgtcgttgaacacccatataaagattacgatacc

gatgtttacccagtaccggtaatcaactatgaaggcgataacttctggttccgtggctta

ggtggtggttactacctgtggaatgacgcaacggataaactttcaattaccgcttactgg

tcgccgctttacttcaaagctaaagacagtggcgatcaccaaatgcgtcacctggatgac

cgtaagagcaccatgatggctggtctgtcttatgctcactttacccagtacggttacctg

cgtaccaccctggctggcgataccctggataacagcaacggcatcgtctgggatatggcc

tggttgtatcgttacaccaacggtggcctgaccgtgactccgggtattggtgtgcagtgg

aacagcgaaaaccagaacgaatactattatggcgtatcgcgcaaagagtccgctcgcagc

ggtctgcgtggctataacccgaacgacagctggagcccttacctggagctgagcgccagc

tacaacttcctcggcgactggagtgtttacggtaccgcgcgctacacccgtctgtctgat

gaagttactgacagcccgatggtggataaatcctggactggcctgatttctaccgggatc

acctacaaattctgagtggaataccgtagcctgacgcttgatgattttttatcgcgcttt

caacttttgcgcccacaaattaaccgggaaaccctaaatcatcgtcaggctgccgtgtta

atccccatcgtccgtcgaccgcaaccggggttgttgctgactcagcgttcaattcatctg

cgtaaacacgctggacaagtggcattccctggaggtgcagtcgatgacacggacgcatca

gttatcgccgccgcgctgcgcgaagctgaagaagaggttgctataccgccttccgccgtt

gaagttatcggcgtgctgccgcccgtcgatagcgtaactggctatcaggtaaccccagtg

gtcggcattatcccgcccgatctgccgtatcgcgccagtgaagatgaagtctcggcggtg

tttgaaatgccgctcgcccaggcattacatctgggtcgttatcaccctttagatatctac

cgccgtggcgattcacatcgggtatggctgtcctggtacgaacagtattttgtatgggga

atgaccgcaggcataattcgtgagctggcgctgcaaattggtgtgaaaccctgagtgacc

attgctattgttataggcacacatggttgggctgcagagcagttgcttaaaacggcagaa

atgctgttaggcgagcaggaaaacgtcggctggatcgatttcgttccaggtgaaaatgcc

gaaacgctgattgaaaagtacaacgctcagttggcaaaactcgacaccactaaaggcgtg

ctgtttctcgttgatacatggggaggcagcccgttcaatgctgccagccgcattgtcgtc

gacaaagagcattatgaagtcattgcaggtgttaacattccaatgctcgtggaaacgtta

atggcccgtgatgatgacccaagctttgatgaactggttgcgctggcagtagaaacaggc

cgtgaaggcgtgaaagcactgaaagccaaaccggttgaaaaagccgcgccagcacccgct

gccgcagcaccaaaagcggctccaactccggccaaaccaatgggaccaaacgactacata

gttattggccttgcgcgtatcgacgaccgtctgattcacggtcaggtcgccacccgctgg

actaaagaaaccaatgtctcccgtattattgttgttagtgatgaagtggctgcggatacc

gttcgtaagacactgctcacccaggttgcacctccgggcgtaacagcacacgtagttgat

gttgccaaaatgattcgcgtctacaacaacccgaaatatgctggcgaacgt--gtaatgc

tgttatttaccaacccaacagatgtagagcgtctcgttgaaggcggcgtgaaaatcacct

ctgttaacgtcggtggtatggcattccgtcagggtaaaacccaggtgaataacgcggttt

cggttgatgaaaaagatatcgaggcgttcaaaaaactgaatgcacgcggtattgagctgg

aagtccgtaaggtttccaccgatccgaaactgaaaatgatggatctgatcagcaaaatcg

ataagtaaatgtcgttttcctgtccactttgccatcagcctctttcgcgtgaaaaaaaca

gctatatctgtccccaacgacatcagtttgatatggcgaaagaagggtatgtcaatctgc

tgcccgttcagcataaacggtcccgtgatccgggcgacagcgcggaaatgatgcaagcac

gccgcgcattcttagatgccggacattatcagccgctgcgtgatgcaattgtcgcccaac

tgcgggaacggcttgatgaaaaggccacggcggtgctggatattggctgtggtgaagggt

attacacacacgcatttgccgatgcgttgcccgaaatcaccacgtttggtctggatgttt

cgaaggtagcgataaaagcggcggcgaaacgctatccgcaggtcactctttgtgtcgctt

ccagccaccgtttgccgttttccgataccagtatggacgccataatacgtatttacgcgc

cgtgtaaagcagaagaattagcacgagtagtgaagcccggcggctgggtcattactgcca

cgccgggaccgcgacatttgatggagctgaaggggctgatttacaatgaagtacatcttc

atgcacctcatgcagaacaactggaaggttttacattacagcagagtgatgagttgtgtt

atccgatgcgtcttcgcggtgatgaagccgtcgcattattgcagatgacgccgtttgcct

ggcgtgcgaagccagaagtctggcaaacactggcagcaaaagaagtgttcgactgccaga

cggactttaatattcacctctggcagcgttcttattaaatgaacgaagttgtaaattcag

gcgtgatgaacattgcgtctttggttgtatcggtggtggttcttcttatcgggctcatct

tgtggttttttattaatcgtgccagttctcggactaacgaacagattgaactgcttgagg

cgttgctggatcagcaaaaacgtcaaaatgcactgttacgtcgtttgtgcgaagcaaacg

aaccagagaaagcagataaaaagaccattgagagtcaaaaatcggttgaagacgaagata

ttattcgcctggtcgccgaacgataaatgatgaaaaaaagtattctggcgtttctgttac

tcaccagttctgcagcggcgctggctgcaccgcaggtgattaccgtcagccgttttgaag

tgggtaaagacaaatgggcgtttaatcgcgaagaggtgatgctgacttgccgaccgggta

atgctttgtatgtcatcaacccaagtaccctcgtgcagtatcctttaaacgatatcgcac

aaaaggaagttgccagtgggaagactaaaacccaacccatttcggtgattcagattgatg

atcctaacaatcccggcgaaaaaatgagtctggcaccgtttatagaacgagctgaaaaac

tctgttaaatgaaaaactggaaaacaagtgcagaatcaatcctcaccaccggcccggttg

taccggttatcgtggtaaaaaaactggaacacgcggtgccgatggcaaaagcgttggttg

ctggtggggtgcgcgttctggaagtgactctgcgtaccgagtgtgcagttgacgctatcc

gtgctatcgccaaagaagtgcctgaagcgattgtgggtgccggtacggtgctgaatccac

agcagctggcagaagtcactgaagcgggtgcacagttcgcaattagcccgggtctgaccg

agccgctgctgaaagctgctaccgaagggactattcctctgattccggggatcagcactg

tttccgaactgatgctgggtatggactacggtttgaaagagttcaaattcttcccggctg

aagctaacggcggcgtgaaagccctgcaggcgatcgcgggtccgttctcccaggtccgtt

tctgcccgacgggtggtatttctccggctaactaccgtgactacctggcgctgaaaagcg

tgctgtgcatcggtggttcctggctggttccggcagatgcgctggaagcgggcgattacg

accgcattactaagctggcgcgtgaagctgtagaaggcgctaagctgtaaatgacaagtc

tggtttccctggaaaatgtctcggtttcttttggccaacgccgcgtcctctctgatgtgt

cgctggaacttaaacctggaaaaattttgactttacttgggccaaatggcgcaggtaagt

cgacactggtacgggtagtgctcgggctggtaacacccgatgaaggggttatcaagcgca

acggaaaactgcgcatcggctatgtaccgcagaagctgtatctcgacaccacgttgccac

tgaccgtaaaccgttttttacgcttacgccctggtacacataaagaagatattttgcctg

cactgaaacgtgtccaggccgggcacctgattaacgcaccgatgcaaaagctctctggtg

gcgaaacgcagcgtgtactgttagcgcgagcattgttaaatcgcccgcaattattagtgc

tggatgaacccactcaaggcgtggatgtaaatggccaggtggcgttatatgaccttattg

accaactgcgtcgtgaactggattgtggcgttttaatggtttctcacgatctgcatctgg

tgatggcaaaaaccgatgaagtgctgtgcctgaatcaccacatttgttgttccggcacac

cggaggttgtttccctgcatccggagtttatttcaatgtttggtcctcgtggtgctgaac

aactgggtatctatcgccatcatcataatcatcgtcacgatttacagggacgaattgttt

tgcgtcggggaaatgatcgctcatgaatgcaatttaatatccctacgttgcttacactgt

tccgtgtcatccttatcccattctttgtattggtcttttatctgcctgtcacctggtcgc

cgtttgccgccgcgctcattttctgcgtcgcggcggtgactgactggttcgatggttttc

tggcacgccgctggaaccagagtacccggtttggtgctttccttgaccctgtagcagata

aagttctcgtggctatcgccatggtgctggtaaccgagcattaccacagctggtgggtga

ccttaccggcggcaacgatgatcgcccgtgaaattattatttctgcgctacgcgaatgga

tggcggagttgggtaaacgcagtagcgtggctgtctcctggattgggaaagtgaaaacca

ctgcccagatggtggcgttggcatggctgctgtggcgtccgaacatttgggttgagtacg

ccggtattgcacttttctttgtggctgcggtactgactctgtggtcaatgttgcaatatt

tgagcgctgcgcgtgcagatttgcttgatcagtgaatgcgtctgtgtgaccgagatattg

aagcctggcttgatgaaggccgtttgtcgatcaacccacgtccgccagtggagcgtatta

acggcgcgacggtggatgtacgcctgggcaataaatttcgtaccttccgtggtcacacgg

cagcgtttatcgatctgagcggtcccaaagatgaagtgagcgccgcgcttgaccgcgtga

tgagcgatgagatcgttctcgacgagggcgaggcgttctatcttcacccaggagagctgg

cgctggcggtgacgctggagtcggtgacgctgccagccgatctggtgggctggctggacg

ggcgttcctcactggcgcgtctggggctgatggtgcacgtcaccgcgcaccgcatcgatc

cgggctggtctggttgcattgtgctggagttctacaactccggtaagctgccgctggcgc

tgcgtccgggcatgttaattggtgcgctgagctttgagccgctttccggcccggcggcgc

gaccttacaaccgccgtgaagatgcgaaatatcgcaaccagcagggcgcggtagccagcc

gaatcgataaagactaaatgaagatgttgcgcgatccgctgttctggctcattgctctgt

ttgtggcgctgattttctggctgccttacagccagccgctgtttgctgccttgttcccac

aactgccacgacccgtttatcagcaagaaagttttgcagctctggcactggctcatttct

ggctggtgggaatttcgagtttgtttgcggtgatcattggcactggtgccggaattgctg

tcactcgcccgtggggcgcggaatttcgcccactggtggaaactattgccgccgttggac

agacttttccgcccgtcgcagttctggcgatcgccgttccggtgatcggctttggtctgc

aaccagcgattatcgccttgatcctttacggtgtgctgcccgtcctgcaggcgacacttg

ccgggctgggagcgattgatgccagcgtgacagaagttgcgaaaggtatgggaatgagtc

gtggtcagcgactgcgtaaggtcgagctaccgctggcggctccggtgattctggcgggcg

tgcgaacttcggtgattatcaacattggtacggcgacgatcgcctcaacggtaggggcca

gcacgctgggtacgcccatcatcatcgggcttagcggatttaataccgcgtatgtgatcc

agggggcgttactggtggcactggcggcgatcatcgcagaccgcctgtttgaaaggctgg

tgcaggcgcttagccagcacgcaaaataaatgttaaagcgcgtgttcctcagcctgttag

tcctgatcggcttgctgctgttgactgtgctcggcctcgatcgctggatgagctggaaaa

ccgcgccttatatctacgacgaattgcaggatctcccctaccgccaggtcggtgtggtgc

tcggaacagcaaaatattatcgtactggcgtaattaatcagtattatcgctaccgcattc

aaggagcgattaatgcctataacagcggtaaggtaaattatctattactgagcggcgata

acgcattgcaaagttataatgagccgatgaccatgcgcaaagatttaatcgctgctggtg

tcgacccatcagatattgttctcgattacgcaggctttcgtacgctggattccatcgtgc

gtacacgcaaagttttcgatactaatgatttcattattatcacccaacgtttccactgtg

agcgagcattatttattgcgctgcatatggggattcaggctcagtgttatgccgtaccgt

caccgaaagatatgctgtcagtacgtattcgtgaatttgccgcccgtttcggtgcgctgg

ctgacctttatatttttaaacgtgaaccgcgttttttagggccgctggtccctattccgg

ctatgcaccaggtaccggaagatgcgcaggggtatcccgccgtcacacccgaacagttac

ttgaattacaaaagaaacaaggaaagtagatgaataagaaggtgttaaccctgtctgctg

tgatggccagcatgttattcggtgccgctgcacacgctgctgaaactcgcattggtgtaa

caatctataagtacgacgataactttatgtctgtagtgcgcaaggctattgagcaagatg

cgaaagccgcgccagatgttcagctgctgatgaatgattctcagaatgaccagtccaagc

agaacgatcagatcgacgtattgctggcgaaaggggtgaaggcactggcaatcaacctgg

ttgacccggcagctgcgggtacggtgattgagaaagcgcgtgggcaaaacgtgccggtgg

ttttcttcaacaaagaaccgtctcgtaaggcgctggatagctacgacaaagcctactacg

ttggcactgactccaaagagtccggcattattcagggcgatttgattgctaaacactggg

cggcgaatcagggttgggatctgaacaaagacggtcagattcagttcgtactgctgaaag

gtgaaccgggccatccggatgcagaagcacgtaccacttacgtgattaaagaattgaacg

ataaaggcatcaaaactgaacagttacagttagataccgctatgtgggataccgctcagg

cgaaagataagatggacgcctggctgtctggcccgaacgccaacaaaatcgaagtggtta

tcgccaacaacgatgcgatggcaatgggcgcggtagaagcactgaaagcacacaacaagt

ccagcattccggtgtttggcgtcgatgctctgccagaagcgctggcgctggtgaaatccg

gtgcactggcgggcaccgtactgaacgatgctaacaaccaggcgaaagcgacctttgatc

tggcgaaaaacttggccgatggtaaaggtgcggctgatggcaccaactggaaaatcgaca

acaaagtggtccgcgtaccttatgttggcgtagataaagacaacctggctgaatttagca

agaaataaatgccatcactcagtaaagaagcggccctggttcatgaagcgttagttgcgc

gaggactggaaacaccgctgcgcccgcccgtgcatgaaatggataacgaaacgcgcaaaa

gccttattgctggtcatatgaccgaaatcatgcagctgctgaatctcgacctggctgatg

acagtttgatggaaacgccgcatcgcatcgctaaaatgtatgtcgatgaaattttctccg

gtctggattacgccaacttcccgaaaatcaccctcattgaaaacaaaatgaaggtcgatg

aaatggtcaccgtgcgcgatatcactctgaccagtacctgtgaacaccattttgttacca

tcgatggcaaagcgacggtggcttatatcccgaaagattcggtgatcggtctgtcaaaaa

ttaaccgtatcgtgcaattctttgcccagcgtccgcaggtacaggaacgtctgacgcagc

aaattctcattgcgctacaaacgctgctgggcaccaataacgtggctgtctctatcgacg

cggtgcattattgcgtgaaggcgcgtggcatccgcgatgcaaccagtgccacgacaacga

cctctcttggtggattgttcaaatccagtcagaatacgcgccacgagtttctgcgcgctg

tgcgtcatcacaactgaatgagcagacgtgttgctactatcacccttaatccggcttatg

accttgttggtttctgcccggaaattgaacgcggcgaagtgaacctggtgaaaaccaccg

gtctgcatgcggcgggtaaaggcatcaacgtggccaaagtattaaaagacctgggaattg

atgtcaccgttggcggcttcctgggtaaagacaatcaggatggttttcagcaactgttca

gcgagctgggcattgccaaccgtttccaggttgtacaggggcgcacccgaattaacgtta

agctgacggaaaaagacggcgaagtgaccgacttcaacttctcgggttttgaagtcactc

ccgccgactgggaacgctttgtgactgattctctgagctggctcggtcagttcgatatgg

tctgtgtcagcggaagcttaccgtcaggcgtcagcccggaagcgttcaccgactggatga

ctcgcctgcgtagtcagtgcccttgcattatctttgatagtagccgtgaagcgttagtag

caggtttgaaagcggcaccgtggctggtgaaacctaaccgccgcgagctggaaatctggg

caggccgtaaactgcctgaaatgaaagatgtgattgaagctgcgcatgcgctgcgtgaac

aaggtattgcgcatgtcgttatttcactgggtgccgaaggcgcgctttgggttaatgcct

ccggcgaatggatcgccaaaccaccgtcagtcgatgtcgtaagcaccgttggcgcagggg

attctatggttggtggcctgatttatggcttgctaatgcgtgaatccagtgaacacacac

tgcgtctggcgactgctgttgcagccctggcggtaagtcaaagcaatgtgggtattaccg

atcgtccgcagttggccgcaatgatggcgcgcgtcgacttacaaccttttaactgaatgt

tccagttatccgtacaggacatccatccgggcgaaaaggccggagacaaagaagaggcga

ttcgccaggtcgctgcggcgctggtgcaggccggtaatgtagcagaaggctacgtcaatg

gcatgctggcgcgcgaacagcaaacctcaacgttcctcggcaatggtattgctattccac

acggcactaccgacacccgcgatcaggtgctgaaaaccggcgttcaggtatttcagttcc

cggaaggcgtcacctggggtgacggtcaggtagcgtacgtagcgatcggtattgctgcca

gctcggatgagcatctgggcctgctacgccagctgacccacgtactgagcgatgattccg

ttgctgaacaactgaagtcagcaacaacagcagaagaacttcgcgcattgctgatgggcg

aaaagcagagtgagcagttgaagctcgacaacgaaatgctgacgctggatatcgtcgcca

gcgatctgctgactcttcaggcgctgaacgctgcgcgtctgaaagaggcgggggcagttg

acgcctctttcgtcaccaaagccatcaatgaacaaccgctgaatctcggacagggtatct

ggctgagcgatagcgccgaaggcaatctgcgtagcgcgattgcggtaagccgtgcggcaa

atgcttttgatgtggacggcgaaacggcagccatgctggtgagtgtggcgatgaatgacg

atcagcccattgcggttcttaagcgtctcgctgatttgttgctcgacaataaagctgacc

gcttgctgaaagcggatgcggcaacgttgctggcgctgctgaccagcgatgatgcgccga

ccgacgacgtgttaagcgcggagtttgtggtgcgcaatgaacacggcttgcatgctcgtc

caggtaccatgctggtcaataccattaaacaatttaacagtgatattaccgtgacaaatc

ttgatggcaccggcaaaccggcaaacggacgtagtctgatgaaagttgtggcacttggcg

ttaagaaaggtcatcgcctacgctttaccgcccagggtgcagatgctgaacaggcgctga

aagcaatcggcgacgctatcgctgctggtcttggggagggcgcataaatggtcaaatctc

aaccgattttgagatatatcttgcgcgggatccccgcgattgcagtagcggttctgcttt

ctgcatgtagtgcaaataacaccgcaaagaatatgcatcctgagacacgtgcagtgggta

gtgaaacatcatcactgcaagcttctcaggatgaatttgaaaacctggttcgtaatgtcg

acgtaaaatcgcgaattatggatcagtatgctgactggaaaggcgtacgctatcgtctgg

gcggcagcactaaaaaaggtatcgattgttctggtttcgtacagcgtacattccgtgagc

aatttggcttagaacttccgcgttcaacttacgaacagcaggaaatgggtaaatctgttt

ctcgcagtaatttgcgtacgggtgatttagttctgttccgtgccggttcaacaggacgcc

atgtcggtatttatatcggcaacaatcagtttgtccatgcttccaccagcagtggtgtta

ttatttccagcatgaatgaaccgtactggaagaagcgttacaacgaagcacgccgggttc

tcagccgcagctaaatgaatattcgccgtaaaaaccgcttgtggattgcctgtgccgtgt

tggcagggctggcgctgactatcggtctggtgctatatgcgctgcgctcgaatatcgatc

tcttttatacgccgggggaaattctctacggcaagcgtgaaactcagcaaatgccggaag

tcggtcagcgtctgcgcgttggcgggatggtgatgccgggtagtgtgcagcgcgatccca

attcgctgaaagtgaccttcaccatttacgacgctgaaggctcagtggatgtctcttacg

aaggcattttgccggatctgttccgtgaagggcagggcgttgtggtgcagggcgagctgg

aaaaaggcaatcatatcctcgcgaaagaagtgctggcgaaacacgacgaaaactacacgc

cgccagaagttgagaaagcgatggaagctaaccaccgtcgcccggcgagtgtttataagg

acccagcatcatgaatgggaaattctgaccgtaagcctggtctgattaagcgcctgtgga

aatggtggcgtacccccagccgtctggcgctggggacgctgctgttgatcggttttgtcg

gcggcatcgtcttctggggcggctttaacactgggatggaaaaagccaataccgaagagt

tctgcattagctgccacgaaatgcgtaacacggtgtatcaggaatacatggattccgtgc

actacaacaaccgtagcggcgtccgtgcgacctgtccggattgtcacgttccgcacgagt

ttgtgccgaagatgatacgcaagctcaaagcaagtaaagagctgtatggtaaaatttttg

gcgttattgacacgccgcagaaatttgaagctcatcgtctgacgatggcacagaatgagt

ggcggcgcatgaaggacaataactcgcaggagtgccgtaactgtcacaacttcgagtata

tggatacaaccgcccagaaatcggttgccgcgaagatgcatgaccaggcggtgaaagatg

ggcaaacctgtattgattgccataaagggatagcgcacaagctgcccgatatgcgtgaag

tcgagccaggtttttaaatgtcccggtcagcgaaacctcaaaatggtcgccgccgctttc

tgcgcgatgttgttcgcacagcaggcgggctggctgccgtgggtgtggcgctggggttac

aacagcaaaccgcacgcgcatctggcgtgcggttgcgcccgcccggagccataaacgaga

acgcctttgccagtgcctgtgtgcgttgtggtcagtgtgttcaggcttgcccttacgaca

ccttaaaactggcgacgctggcctctggtctgtcggcgggcacgccatattttgtcgcac

gggatattccttgcgaaatgtgtgaggacattccgtgcgccaaagtgtgcccgagcggtg

cgctggatcgtgagattgaatcgatcgacgacgcgcggatggggctggcggtactggtgg

accaggaaaactgtctcaactttcaggggctgcgctgcgatgtttgttatcgcgaatgcc

cgaaaattgatgaggccatcaccctggagctggagcgcaacacgcgtaccggtaagcacg

cccgctttctgccgacggttcacagcgacgcctgtactggttgcggtaagtgcgaaaaag

tgtgcgtgctggaacaaccggcaatcaaggtgttaccgctgtcactggcgaaaggggagt

taggtcaccattaccgcttcggctggctggaggggaacaatggcaaatcgtaaatggcta

gcgttaccctgcgcatcactggcacacaactgctgtgccaggatgaacacccttcccttc

tggcggcgctggaatcccacaatgtggcggttgagtaccagtgccgcgaaggttactgcg

gctcctgtcgcacccgactggtcgcaggccaggtggactggattgccgaaccgttagctt

ttattcagccgggggaaattttgccctgttgttgccgggcaaaaggcgatattgaaatcg

agttgtgaatgaccaaagtcggcttacgcattgatgtcgatacctttcgtggcacccgtg

aaggcgtgccgcgtctgctggaaatcttgagtaagcataatattcaggccagcatttttt

tcagcgtcggcccggacaatatgggccgccatctctggcgactggtgaagccacagtttt

tgtggaagatgctgcgctcaaacgcgacatcgctttatggctgggatattttactggcag

gtacggcctggccaggtaaagagattggtcatgccaatgccgatatcattcgtgaagcgg

ctaaacatcacgaagtcggcctgcacgcctgggatcaccatgcctggcaagcccatagcg

gtaactgggatcggcaaacaatgatcgacgatattgcactcggccttcgcaccctggaag

agattatcggtcaaccggtaacctgttctgccgctgcgggctggcgtgccgaccagcagg

tgatcgaagcaaaagaagcgttccatttgcgctacaacagcgattgtcgtggggccatgc

cgttccgtccactgctcgaatcaggaaaccctggcactgcgcaaattccggtgaccttac

ccacctgggatgaagtgattggtcgggatgtgaaagcagaagattttaacggttggttac

tcaaccgcatcctgcgagataaaggcacgccggtttataccattcatgcagaagttgaag

gctgcgcttatcagcataattttgtggatctcctcaaacgcgcagctcaggaaggcgtga

cattttgccctttaagcgaactgttatcagagacgttgccgctcggacaagttgttcgcg

gaaatattgccggacgtgaaggctggctgggttgccaacaaattgcgggtagtcgctgaa

tgatttatcctgatgaagcaatgctttacgcaccggttgaatggcacgactgctccgaag

gtttcgaggacattcgttatgaaaaatccaccgacggtatcgcaaaaatcaccattaatc

gtccgcaggtgcgcaatgccttccgtcctctgacggtaaaagagatgatccaggcactgg

cagatgcgcgttatgacgacaatatcggcgtgatcattctgactggcgcaggcgataaag

cgttctgctccggtggtgaccagaaagtgcgtggtgattacggcggctataaagatgatt

ccggcgtacatcacctgaacgtgctggacttccagcgtcagatccgtacctgtccgaaac

cggttgtcgcgatggtggctggctactccatcggcggtggtcacgttctgcacatgatgt

gcgacctgactatcgcggcagataatgccatctttggtcagactggcccgaaagtcggtt

ccttcgacggcggctggggcgcttcctacatggctcgtatcgtcgggcagaaaaaagcgc

gtgaaatctggttcctgtgccgtcagtacgacgcaaaacaggcgctggatatgggccttg

tcaacaccgtggtaccgctggcggatctggaaaaagaaaccgtccgttggtgccgtgaaa

tgctgcaaaacagcccgatggcgctgcgttgcctgaaagctgcgctgaacgccgactgtg

acgggcaggcggggctgcaggagctggcgggcaacgccaccatgctgttctacatgacgg

aagaaggtcaggaaggtcgcaacgccttcaaccagaaacgtcagcctgacttcagcaaat

tcaaacggaatccgtaaatgatcctgcacgcgcaggcaaaacacggcaaaccaggtttac

cctggctggtgtttttgcacggtttttccggcgattgccacgaatggcaagaagtaggcg

aggcgtttgccgactactcacggttgtatgttgatctcccaggtcacggtggttcggcgg

cgattagagtcgatggatttgatgatgtcactgacttactgcgtaaaaccctggttagtt

acaacatccttaacttctggttggcgggatactcacttggtggacgggtggcgatgatgg

cggcttgccaggggctggcggggctttgtggggttattgtcgaaggtgggcatccggggc

tgcaaaatgctgaacaacgtgcggaacgtcagcgttccgatcgccaatgggcgcagcgtt

ttcgctcagaaccgttaacggcggtgtttaccgactggtatcaacagcctgtttttgcct

cgctcaatgacgatcaacgccgggagctggtggcgctgcgcagcaacaataatggcgcaa

cccttgccgccatgctggaggcgacttctctcgccgttcagcctgatttacgtgctaacc

ttagcgcccgcacatttgcgttttattatttatgtggtgaacgtgacagcaaattccgcg

ccctggcggcggaactggctgccgactgccatgtcattcctcgcgccggacataacgcgc

atcgggaaaatcccgctggcgtaatcgcaagtctggcgcagatcttgcgtttctgaatgt

ctaatcagtttggtgatacacgtatcgatgacgacctgacgctgcttagtgaaacactgg

aagaggtgctccgctcctctggcgatcccgccgatcagaaatatgttgagctgaaagcgc

gtgcagaaaaagcgctggatgatgtgaaaaaacgggttagccaggcttcagacagttatt

actatcgggcgaagcaggctgtttatcgtgctgatgactacgtccacgaaaaaccctggc

agggaattggtgtgggtgcggccgttgggctggtactaggactgttgctggcacgccgtt

aaatgatccccttacaacatggactgatcctcgcggcaatcttattcgttcttggcttaa

ccggtctggttatccgtcgcaatctgctgtttatgctgattggtctggaaatcatgatta

acgcctccgcgctggcctttgtggtcgccggaagctactggggtcagaccgacggtcagg

tgatgtacattctcgccatcagcctcgcggcggcagaagcgagtatcggccttgcgctgc

tgctgcaacttcaccgtcgtcgccagaacctgaacatcgattcagtaagtgagatgcgcg

gatgaatgaccttaaaagaattgttagtaggtttcggcacccaggttcgtagtatctgga

tgatcggcctgcacgcgttcgccaaacgcgaaacgcgaatgtacccggaagagccggtct

atctgccgccccgttatcgtggtcgtatcgttctgacccgcgacccggacggcgaagagc

gttgcgtagcctgtaacctctgcgcggtagcctgcccggtcggctgtatctcgctgcaaa

aagcagaaaccaaagacggtcgctggtatccggaatttttccgcatcaacttctcacgct

gcattttctgtggtttgtgcgaagaagcctgtccgaccacggcgattcagttaaccccgg

atttcgaaatgggggaatacaagcgccaggatctggtttacgagaaagaggatctgctga

tctccggtccgggcaaatacccggagtataacttctaccggatggcaggtatggcaatcg

acggcaaagataagggcgaagcagagaacgaagccaagcctatcgacgtcaagagcctgt

taccgtaaatgcacgagaatcaacaaccacaaaccgaggcttttgagctgagtgcggcag

agcgtgaagcgattgagcacgagatgcaccactacgaagacccgcgtgcggcgtccattg

aagcgctgaaaatcgttcagaagcagcgtggctgggtgccggatggtgcgatccacgcga

tcgccgatgtgctgggtattccggcaagcgacgtcgaaggtgtggcaacgttctacagtc

agatcttccgccagccggttggtcgccatgtgatccgttattgcgacagcgtggtctgtc

atatcaacggttatcagggtattcaggcggcgcttgagaaaaagctgaacatcaaaccag

ggcaaacgacatttgatggccgctttacgctgctgccaacttgctgcctggggaactgtg

ataaagggccaaacatgatgatcgatgaggacactcacgcgcatctgaccccggaagcga

tccctgaactgctggagcggtataaatgaatggattatacgctcacccgcatagatccca

acggtgagaacgaccgttaccccctgcaaaagcaggagatcgtaaccgaccctctggagc

aagaagttaacaaaaacgtgtttatgggcaagctcaatgacatggttaactggggtcgta

aaaactcaatttggccgtataacttcggtctttcctgctgttacgttgagatggtgactt

cgtttaccgcggtgcatgacgtggcgcgttttggcgcagaagtattgcgtgcttcgccgc

gtcaggctgacctgatggtggttgcaggaacctgctttaccaaaatggcaccggttattc

agcgtctgtatgaccagatgctggaaccaaaatgggttatctcaatgggtgcctgtgcca

actctggtggtatgtacgatatttattccgttgtgcagggcgtcgataaattcatcccgg

ttgatgtgtatatcccgggctgcccgccgcgtcctgaagcgtacatgcaggcactgatgc

tgttgcaggaatctatcggcaaagaacgtcgtccgctctcctgggtggttggcgatcagg

gcgtttatcgcgccaatatgcaatcagagcgcgaacgcaagcgcggtgaacgcattgccg

taaccaacctgcgtacacctgacgagatttaaatgagtatgtcaacatccactgaagtca

tcgctcatcactgggcattcgctatctttcttatcgttgccattggcctgtgttgcctga

tgctggtaggcggttggtttttaggcggtcgcgcacgcgcgaggtcgaaaaacgtgccgt

ttgaatccggtatcgactcggtcggctccgcccgcttacgcctgtccgccaagttttatc

tggtggccatgttcttcgttatcttcgacgttgaagcgctgtatctgttcgcatggtcaa

cctctatccgcgaaagcggctgggtaggctttgtggaagctgcaatttttatttttgtgt

tactggcaggtctggtttatctggtgcgtattggcgcgctggactggacgcccgcgcgtt

cacgccgcgagcgtatgaacccggaaacgaacagtatcgctaatcgtcaacgctaaatgt

cccccattgaaaaatccagcaaattagagaatgtctgttatgacatccgtggtccggtgc

tgaaagaagcaaaacgcctggaagaagaaggtaacaaggtactgaaactgaacatcggca

acccagccccgttcggttttgacgcgccagatgaaatcctcgttgacgtgatacgcaacc

tgcctacagctcaagggtattgcgattccaaaggtctttactccgcgcgtaaagccatca

tgcagcactaccaggctcgtggcatgcgtgatgttaccgtggaagatatttacatcggca

atggtgtatcggagcttatcgttcaggcaatgcaggcattgctgaacagcggggacgaaa

tgttggttcctgcaccagattacccactatggaccgcggcggtttcgctttccagcggta

aagcggtgcattatctttgcgatgaatcctctgactggttcccggacctcgatgatattc

gcgctaaaattacgcctcgtacgcgtgggatcgttattatcaacccaaataacccaaccg

gcgcggtatattccaaagagcttttaatggagattgtggagattgcacgtcagcataatc

tcattatcttcgccgatgaaatttatgacaaaattctctacgacgacgctgagcatcact

caattgcgccgctggcacctgacctgctgaccattacctttaatggactgtcgaaaacgt

accgcgttgcaggcttccgtcaggggtggatggtgttgaacgggccgaaaaaacacgcca

aaggctacatcgaaggtctggaaatgctggcttcaatgcgcctgtgtgctaacgttcctg

cgcaacacgccattcagaccgcgctgggtggttatcagagcatcagtgaatttattaccc

ctggcggtcgtctttatgagcagcgtaaccgcgcgtgggaattgatcaacgatattccgg

gcgtttcctgcgtgaaacctcgtggtgcgctgtacatgttcccgaaaatcgacgccaaac

gctttaacattcacgacgatcagaaaatggtgctggatttcctgttgcaggaaaaagttc

tgttggtgcaagggacggcattcaactggccgtggccggatcacttccgcattgtcacgc

taccgcgtgtcgatgatatcgagctgtctttgagcaagttcgcgcgtttcctttctggtt

atcatcagctgtaaatgtcaacgccggataatcgttctgttaatttttttagcttgtttc

gccggggacagcattactcaaagacgtggccgctggaaaaacgccttgccccggtctttg

tcgaaaatcgcgttatcaagatgacgcgttatgcgatccgttttatgccgccgatcgccg

tatttactctctgctggcagattgccctgggcggtctgcttgggccggcagttgccactg

-cgctgttcgccttaagtttacccatgcagggattgtggtggctgggcaagcgttctgtc

acgccattaccccctgctatcctcaactggttttatgaagttcgcggtaaattgcaggag

tctggacaggtgttggcacccgttgaaggcaagcctgattaccaggcattagctgacacg

cttaagcgcgccttcaaacaactggataaaactttccttgatgatttgtaaatgtcgagt

aagttagtactggttctgaactgcggtagttcttcactgaaatttgccatcatcgatgca

gtaaatggtgaagagtacctttctggtttagccgaatgtttccacctgcccgaagcacgt

atcaaatggaaaatggacggcaataaacaggaagcggctttaggtgcaggcgccgctcac

agcgaagcgctcaactttatcgttaatactattctggcacaaaaaccagaactgtctgcg

cagctgactgctatcggtcaccgtatcgtacacggcggcgaaaagtataccagctccgta

gtgatcgatgagtctgttattcagggtatcaaagatgcagcttcttttgcaccgctgcac

aacccggctcacctgatcggtatcgaagaagctctgaaatctttcccacagctgaaagac

aaaaacgttgctgtatttgacaccgcgttccaccagactatgccggaagagtcttacctc

tacgccctgccgtacaacctgtacaaagagcacggcatccgtcgttacggcgcgcacggc

accagccacttctatgtaacccaggaagcggcaaaaatgctgaacaaaccggtagaagaa

ctgaacatcatcacctgccacctgggcaacggtggttccgtttctgctatccgcaacggt

aaatgcgttgacacctctatgggcctgaccccgctggaaggtctggtcatgggtacccgt

tctggtgatatcgatccggcgatcatcttccacctgcacgacaccctgggcatgagcgtt

gacgcaatcaacaaactgctgaccaaagagtctggcctgctgggtctgaccgaagtgacc

agcgactgccgctatgttgaagacaactacgcgacgaaagaagacgcgaagcgcgcaatg

gacgtttactgccaccgcctggcgaaatacatcggtgcctacactgcgctgatggatggt

cgtctggacgctgttgtattcaccggtggtatcggtgaaaatgccgcgatggttcgtgaa

ctgtctctgggcaaactgggcgtgctgggctttgaagttgatcatgaacgcaacctggct

gcacgtttcggcaaatctggtttcatcaacaaagaaggtacccgtcctgcggtggttatc

ccaaccaacgaagaactggttatcgcgcaagacgcgagccgcctgactgcctgaatggaa

cagcgtcgtttggcaagtactgaatgggtggatattgtcaatgaagagaacgaagtcatt

gcacaagccagccgggaacaaatgcgggcacagtgtctgcgtcatcgtgcaacttacatc

gtcgtgcatgatggcatgggcaaaattctggtccagcgtcgtaccgagacaaaagacttt

ttacccggcatgttagatgcgaccgcaggcggtgtagtccaggccgatgagcaactgctg

gaatccgcgcgtcgcgaagcggaagaagagttgggcattgccggtgtcccctttgccgag

cacgggcagttctatttcgaagataaaaattgccgtgtctggggcgcattgttcagctgc

gtctctcacggtcccttcgccctacaggaagatgaagtcagtgaagtttgctggctgacg

ccggaagaaatcaccgcacgctgcgatgagttcactccagactcgctgaaagcgctagcg

ttgtggatgaagcgcaatgccaaaaatgaagccgtagagactgaaacggcagaatgaatg

atcgatctctatttcgccccgacacccaatggccacaaaattacgctgtttctcgaagaa

gcagggctggattatcgcttgataaaggtagacctggggaaaggcggtcagtttcgcccg

gaatttttgcgcatttcgcctaacaacaaaattccggcaattgttgatcattctccagcc

gatggcggcgaaccgctaagcctctttgaatctggtgccattttgttgtatctggctgag

aaaacggggctctttttgagtcatgaaacgcgtgaacgcgccgccacattacagtggtta

ttctggcaggtaggcggactggggccgatgcttgggcaaaatcatcattttaatcacgca

gccccccaaaccattccttacgctattgaacgttatcaggttgaaactcagcgtctgtac

catgtactgaacaagcggctggaaaactcgccctggctgggaggcgagaactacagcatt

gcggatattgcctgctggccgtgggttaatgcctggactcgccagcgaattgacctcgca

atgtatccggcagtcaagaactggcatgagcggatccgttcgcgccctgccaccgggcag

gcactgctaaaagcacaactcggtgatgagcgttcggatagttaaatggtctggattgat

tacgccataatcgcggtgattgctttttcctctctggttagcctgatccgcggctttgtt

cgtgaagcgttatcgctggtgacatggggttgtgctttctttgttgccagtcattactac

acttacctgtcagtctggtttacgggctttgaagacgaactggttcgaaatgggattgcc

atcgcggtactgtttatcgctaccctgatcgttggtgctatcgtgaacttcgtgataggc

cagttggtggagaaaacggggttgtcaggcaccgatcgggtgctgggcgtctgtttcggt

gcgttgcgcggtgtgttgattgttgccgccattctcttctttctcgactcctttaccggg

gtgtcgaaaagcgaagactggagcaaatcacagctgatcccgcagttcagttttatcatc

agatggttttttgattatctgcaaagctcgtcaagtttcttgcccagagcgtaaatgatc

gcggagtttgaatcacgcattctggcattaatcgacggtatggttgaccatgccagtgat

gatgagttgtttgccagtgggtatttgcgtggccacctgacattagccatcgcagaactg

gaaagtggtgatgaccactccgctcaggcggtgcatacgaccgttagccagagtctggaa

aaagccattggtgccggtgaattgtcgccgcgtgaccaggcgctggtgaccgatatgtgg

gaaaacctgtttcagcaggcgtcacagcagtaaatgaacaaaaccgcgattgcgctgctg

gccctgcttgccagtagcgtcagcttggcagcgacgccgtggcaaaaaataacccaacct

gtgccgggtagcgcacaatcgataggcagtttttctaatggctgtatcgtcggcgctgac

acgctgccgatacagtccgaacattatcaggtcatgcgtaccgatcagcgtcgctatttc

ggtcacccggacctggtgatgtttatccagcgtctgagtagccaggtgagcaatctgggc

atgggtacggtgctgattggcgatatggggatgcccgctggtgggcgtttcaacggcggt

catgccagccaccagaccggactggatgtcgatatctttctgcaactgccgaaaactcgc

tggacctccgcgcagctcttgcgcccgcaagcactggacttagtatcccgcgacggtaaa

cacgttgtctccacgctgtggaagccagaaattttcagcttgatcaaactcgccgcccag

gacaaagacgtcacgcgcatttttgttaatccggcgattaaacaacaactttgccttgat

gcgggcaccgatcgcgactggttgcgcaaagtgcgaccctggttccagcatcgtgcgcat

atgcatgtacgattacgttgtcctgccgatagtctggagtgtgaagatcaacctttaccg

ccaccaggcgatggttgcggggcagaactgcaaagctggtttgaacctccaaaaccggga

acaacaaagcctgagaagaagacaccgcctccgttgccgccttcctgccaggcgctactg

gatgagcacgtgatctaaatgcaacttgaaaagatgattaccgaaggctcgaacgccgcc

tcggctgaaattgaccgcgtatcaacgttggaaatgtgccggattatcaacgatgaagat

aaaaccgtaccgcttgccgttgagcgcgtactgccggatatcgccgcggcgatcgatgtt

atccacgcccaggtcagcggcggcggtcgtctgatttatctcggtgcgggaacatccggt

cgtctggggattctggatgccagcgaatgtccgcccacctacggcgtgaaaccgggtttg

gtggttggtttgattgctggaggcgaatatgccattcagcacgcggtggaaggcgcggaa

gatagccgggaaggcggcattaacgatctgaaaaatattaatttaacggcacaggatgtg

gtggtcggtattgccgccagcggtcgtacgccgtatgtgattgccggactggaatatgca

cgccagctcggctgccgtacagtgggaatttcctgtaatccggggagcgccgtttccacc

accgctgagtttgctattacgccggtggttggggccgaagtggtcaccggttcttcgcgg

atgaaagcaggcacggcgcagaaactggtgctcaatatgctttccaccgggctgatgatt

aaatccggcaaagtgttcggcaacctgatggtcgatgtggtcgccaccaacgaaaaactg

catgtgcgccaggtcaatatcgttaaaaacgccaccggatgtagcgcagagcaagcggaa

gcggcgttaattgcctgcgagcgcaactgtaaaacggccattgtgatggtgctgaaaaat

ctcgatgccgctgaagctaaaaaacgcctgaatcaacacggcggatttattcgcaaggct

ctggaaaaggaataaatgagcacttttaaaccactaaaaacactcacttcgcgccgccag

gtgctgaaagccggtttggctgccctgacgttgtcaggaatgtcgcaagccatcgccaaa

gacgaacctttaaaaaccagcaacggacacagcaagccgaaagctaaaaaatctggcggc

aaacgtgtcgttgttctcgatccaggccacggcgggattgataccggagcgatcgggcgc

aacggttcgaaagaaaaacatgtggtgctggcgattgctaaaaacgtccgttccattttg

cgtaatcatgggattgatgcgcgtttaacgcgttctggcgatacgtttatcccactttac

gatcgcgttgaaatcgcccataaacatggcgcagatctgtttatgtcaattcatgccgat

ggctttaccaacccgaaagctgccggtgcttcggtatttgccctctctaaccgtggggca

agtagcgcaatggcgaaatacctgtctgaacgcgaaaaccgcgccgatgaagttgccggt

aaaaaggcgactgacaaggatcacctattgcagcaagtgctgtttgatctggtgcaaacc

gatactattaaaaacagcctgacgctcggctcgcatattctgaaaaagattaagccggtg

cataaactgcacagccgcaacaccgaacaagccgcgtttgtggtgttgaaatcgccgtcg

gttccttcggtgctggtggaaacctcgtttatcaccaacccggaagaagagcgactgtta

ggctcggcggcgtttcgtcagaaaatcgccacagcgattgctgaaggcgtgatcagttat

ttccactggttcgacaaccagaaagcacattcgaaaaagcgataaatggttacactttac

ggtatcaaaaattgtgacaccattaaaaaggctcgccgttggctggaagccaataacatc

gactatcgttttcatgattaccgcgtcgatgggctggacagcgaattattgaacggtttt

atcaacgaattaggctgggaagcgttactcaacacccgtggtacaacctggcgtaaactg

gacgaaaccacccgcaataaaatcaccgatgcggcctctgcggcggcattaatgactgaa

atgcctgcaattatcaaacgtccattgctctgcgcgcccggtaagcctatgctgctgggt

ttcagtgattccagttatcagcaatttttccatgaggtgtagatgaatccactgaaagcc

ggtgatatcgcaccgaaatttagcttgccggatcaagacggagaacaagttaatttgacc

gacttccagggacagcgtgttctggtttatttctacccgaaagccatgacccccggctgt

accgtacaggcctgcggcttacgcgataacatggatgagttgaaaaaagcgggcgttgat

gtgctgggtatcagcaccgataaacccgaaaaactctcccgttttgcggaaaaagagctg

cttaactttacgctcctgtctgatgaggaccacctggtgtgcgagcaattcggcgtctgg

ggggaaaagtccttcatgggcaaaacctacgatggtattcatcgcatcagcttcctgatt

gacgctgatggcaaaatcgaacatgtctttgacgatttcaaaaccagcaatcaccacgac

gttgtgctgaactggctgaaagaacacgcctgaatgctcgaaatgttgatgcaatggtat

cgccgccgttttagcgacccggaagcgattgccttgctggttattttagttgccggattt

ggcattatctttttctttagtggcctgcttgctccgttgctggtggctattgtgctggcc

tatttgctggaatggccaaccgtgcgcctgcaatctattggctgctcccgccgctgggcg

acgtcgattgtattggtggttttcgtcggtatattgctactgatggcgttcgtggtactg

cctatcgcctggcaacagggcatctacttaatccgcgatatgccggggatgctcaataag

ctttctgactttgccgccacgttgccgcgccgctatccggcgttaatggatgcgggcatt

attgatgcaatggccgaaaatatgcgcagtcggatgctgaccatgggcgattcggtggtg

aaaatttccctcgcctcgctggtcggtttgctgaccatagccgtctatctggtgctggtg

ccattgatggtcttcttcctgctgaaagacaaagagcagatgctgaacgccgttcgtcgg

gtgctgccgcgcaaccgtggactggcaggacaggtgtggaaggagatgaatcaacaaatc

accaactatatccgcggcaaagtgctggagatgatcgtggtggggatcgccacctggctg

gggttcttgctctttgggctgaactattcgctgctgctggcggtgctggtcggcttctcg

gtacttattccgtacattggcgcatttgtggtgaccattccggtggttggcgtggcgcta

ttccagtttggtgcaggcactgaattctggagctgtttcgcggtgtatctgattattcag

gcgcttgacggcaatctgttagtaccggtgttgttctccgaagcggttaacctgcatccg

ctggtgattattttatcggtggtgatcttcggtggtttgtggggattctggggcgtattc

ttcgccattccattggcgacgctgatcaaagccgtgatccacgcctggcccgatgggcaa

atagcgcaagaataaatgaatattgtggtgcttatttccggcaacggaagtaatttacag

gcaattattgacgcctgtaaaaccaacaaaattaaaggcaccgtacgggcagttttcagc

aataaggccgacgcgttcggccttgaacgcgcccgccaggcgggtattgcaacgcatacg

ctcatcgccagcgcgtttgacagtcgtgaagcctatgaccgggagttgattcatgaaatc

gacatgtacgcacccgatgtggtcgtgctggctggttttatgcgcattctcagcccggcg

tttgtctcccactatgccgggcgtttgctgaacattcacccttctctgctgccgaaatat

cccggattacacacccatcgtcaggcgctggaaaatggcgatgaagagcacggtacatcg

gtgcatttcgtcaccgatgaactggacggtggcccggttattttacaggcgaaagtcccg

gtatttgctggtgatacggaagatgacgtcaccgcccgcgtgcaaacccaggaacacgcc

atttatccactggtgattagctggtttgccgatggtcgtctgaaaatgcacgaaaacgcc

gcgtggctggatggtcaacgtctgccgccgcagggctacgctgccgacgagtaagtggaa

atttacgagaacgaaaacgaccaggtagaagcggttaaacgcttttttgctgaaaatggc

aaagcactggctgttggggtgattttgggcgttggcgcactgattggctggcgctactgg

aacagccatcaggttgattctgcacgctccgcttctcttgcctatcaaaatgcggttacc

gcagtgagcgaaggcaaaccggatagcatcccggcggcggaaaaatttgctgctgaaaat

aaaaatacttatggtgcgctggcttctttggaacttgcgcagcaatttgttgacaaaaat

gaactagagaaagctgccgcccagttacaacaggggctggcagacacgagcgatgaaaat

ctcaaagccgtgataaatctgcgtcttgctcgcgttcaggtacagctcaagcaggctgat

gccgcgctgaaaacccttgataccatcaaaggtgaagggtgggctgccattgttgccgac

ctgcgtggtgaagcattgctgagcaaaggtgataagcaaggtgcgcgtagtgcatgggaa

gcaggcgtgaaaagcgatgttactccggcactgagcgaaatgatgcagatgaaaattaat

aatttgtccatctgaatggctattgaacgtactttttccattatcaaaccgaacgcggta

gcaaaaaacgtcattggtaatatctttgcgcgctttgaagctgcagggttcaaaattgtc

ggcaccaaaatgctgcacctgaccgttgaacaggctcgtggcttttatgctgaacacgat

ggaaaaccgttctttgatggtctggttgaattcatgacctctggcccgatcgtggtttcc

gtgctggaaggtgaaaacgccgttcagcgtcaccgcgatctgctgggcgcgaccaatccg

gcaaacgcactggccggtactctgcgcgctgattacgctgacagcctgaccgaaaatggt

acccacggttctgactccgtcgaatctgccgctcgcgaaatcgcttatttcttcggcgaa

ggcgaagtgtgcccgcgcacccgttaaatggcttacagcgaaaaagttatcgaccattac

gagaatccgcgtaacgtgggttcctttgacaacaacgacgagaacgtcggcagcggcatg

gtgggtgcaccggcctgtggcgacgtgatgaagttgcagattaaagtcaacgatgaaggt

atcattgaagacgcgcgttttaaaacttacggctgcggttccgctatcgcttccagctcc

ctggtcaccgaatgggtgaaagggaagtctctcgacgaagcgcaggcgatcaaaaacacc

gatattgctgaagaacttgaactgccgccggtgaaaattcactgttctattctggcagaa

gacgcgatcaaagccgccattgcggactataaaagcaaacgtgaagcaaaataaatgaga

ctgacatctaaagggcgctatgccgtgaccgcaatgcttgatgttgcgctcaactctgaa

gcgggcccggtaccgttggctgatatttccgaacgtcagggaatttccctttcttatctg

gaacaactgttttcccgtctgcgtaaaaatggtctggtttccagcgtacgtggaccaggc

ggtggttatctgttaggcaaagatgccagcagcatcgccgttggcgaagtgattagcgcc

gttgacgaatctgtagatgccacccgttgtcagggtaaaggcggctgtcagggcggcgat

aaatgcctgacccacgcgctgtggcgtgatttgagcgaccgtctcaccggttttctcaac

aacattactttaggcgaactggttaataaccaggaagtgctggatgtgtctggtcgtcag

catactcacgacgcgccacgcacccgcacacaagacgcgatcgacgttaagttacgcgct

taaatgcatccgatgctgaacatcgccgtgcgcgcagcgcgcaaggcgggtaatttaatt

gccaaaaactatgaaaccccggacgctgtagaagcgagccagaaaggcagtaacgatttc

gtgaccaacgtagataaagctgccgaagcggtgattatcgacacgattcgtaaatcttac

ccacagcacaccatcatcaccgaagaaagcggtgaacttgaaggtactgatcaggatgtt

caatgggttatcgatccactggatggcactaccaactttatcaaacgtctgccgcacttc

gcggtatctatcgccgttcgtatcaaaggccgcaccgaagttgctgtggtatacgatcct

atgcgtaacgaactgttcaccgccactcgcggtcagggcgcacagctgaacggctaccgt

ctgcgcggcagcaccgctcgcgatctcgacggtactattctggcgaccggcttcccgttc

aaagcaaaacagtacgccactacctacatcaacatcgtcggcaaactgttcaacgaatgt

gcagacttccgtcgtaccggttctgcggcgctggatctggcttacgtcgctgcgggtcgt

gttgacggtttctttgaaatcggtctgcgcccgtgggacttcgctgcaggcgagctgctg

gttcgtgaagcgggcggcatcgtcagcgacttcaccggtggtcataactatatgctgacc

ggtaacatcgttgctggtaacccgcgtgttgttaaagccatgctggcgaacatgcgtgac

gagttaagcgacgctctgaagcgttaaatgcttgacgctcaaaccatcgctacagtaaaa

gccaccatccctttactggtggaaacggggccaaagttaaccgcccatttctacgaccgt

atgtttactcataacccagaactcaaagaaatttttaacatgagtaaccagcgtaatggc

gatcaacgtgaagccctgtttaacgctattgccgcctacgccagtaatattgaaaacctg

cctgcgctgctgccagcggtagaaaaaatcgcgcagaagcacaccagtttccagatcaaa

ccggaacagtacaacatcgtcggtgaacacctgttggcaacgctggacgaaatgttcagc

ccggggcaggaagtgctggacgcgtggggtaaagcctatggtgtactggctaatgtattt

atcaatcgcgaggcggaaatctataacgaaaacgccagcaaagccggtggttgggaaggt

actcgcgatttccgcattgtggctaaaacaccgcgcagcgcgcttatcaccagcttcgaa

ctggagccggtcgacggtggcgcagtggcagaataccgtccggggcaatatctcggcgtc

tggctgaagccggaaggtttcccgcatcaggaaattcgtcagtactctttgactcgcaaa

ccggatggcaaaggctatcgtattgcggtgaaacgcgaagagggtgggcaggtatccaac

tggttgcacaatcacgccaatgttggcgatgtcgtgaaactggtcgctccggcaggtgat

ttctttatggctgtcgcagatgacacaccagtgacgttaatctctgccggtgttggtcaa

acgccaatgctggcaatgctcgacacgctggcaaaagcaggccacacagcacaagtgaac

tggttccatgcggcagaaaatggcgatgttcacgcctttgccgatgaagttaaggaactg

gggcagtcactgccgcgctttaccgcgcacacctggtatcgtcagccgagcgaagccgat

cgcgctaaaggtcagtttgatagcgaaggtctgatggatttgagcaaactggaaggtgcg

ttcagcgatccgacaatgcagttctatctctgcggcccggttggcttcatgcagtttgcc

gcgaaacagttagtggatctgggcgtgaagcaggaaaacattcattacgaatgctttggc

ccgcataaggtgctgt--atggaaggctggcagcgcgcatttgtcctgcatagtcgcccg

tggagcgaaaccagcctgatgctggacgtcttcacggaggaatcggggcgcgtgcgtctg

gttgccaaaggcgcacgctctaaacgctctaccctgaaaggtgcattacagcctttcacc

cctctcttgctacgttttggcgggcgtggcgaagtcaaaacgctgcgcagtgctgaagcc

gtctcgctggcgctgccattaagcggtatcacgctttacagcggtctgtacatcaacgaa

cttctctcccgcgtactggaatacgagacgcgcttctctgaactttttttcgattacttg

cactgcattcagtctcttgcagggggcactggtacgccagaacccgcgctgcgccgcttt

gaactggcactgctcgggcatctgggttatggcgtcaattttacccattgtgcgggtagc

ggcgagccggtagatgacaccatgacgtatcgttatcgcgaagaaaaagggtttatcgca

agcgtcgttatcgataataaaacgttcaccggaaggcagttaaaagcgttaaacgcacgg

gaatttcctgacgcagacacactgcgcgccgcgaaacgctttacccgcatggcgcttaag

ccgtatcttggcggtaaacctttaaagagcagggaactgttccggcaatttatgcctaag

cgaacggtgaaaacacattatgaatgaatgaaccccatcgtaattaatcggcttcaacgg

aagctgggctacacttttaatcatcaggaactgttgcagcaggcattaactcatcgtagt

gccagcagcaaacataacgagcgtttagaatttttaggcgactctattctgagctacgtt

atcgccaatgcgctttatcaccgtttccctcgtgtggatgaaggcgatatgagccggatg

cgcgccacgctggtccgtggcaatacgctggcggaactggcgcgcgaatttgagttaggc

gagtgcttacgtttagggccaggtgaacttaaaagcggtggatttcgtcgtgagtcaatt

ctcgccgacaccgtcgaagcattaattggtggcgtattcctcgacagtgatattcaaacc

gtcgagaaattaatcctcaactggtatcaaactcgtctggacgaaattagcccaggcgat

aaacaaaaagatccgaaaacgcgcttgcaagaatatttgcagggtcgccatctgccgctg

ccgacttatctggttgtccaggtacgtggcgaagcgcacgatcaggaatttactatccac

tgccaggtcagcggcctgagtgaaccggtggttggcacaggttcaagccgtcgtaaggct

gagcaggctgccgccgaacaggcgttgaaaaaactggagctggaatgaatgaagcaactt

tggtttgccatgtcattagtgacaggtagcctgttattctctgctaacgcctcggccact

cccgcgtccggggcgttattacagcagatgaacctggccagtcagtcactgaattacgag

ctgtcattcatcagcatcaataaacagggtgttgagtctctgcgttatcgacatgcacgc

ctcgataaccgtcctcttgcacaattgttgcaaatggatggcccgcgccgggaagtggta

cagcgcggcaatgaaatcagctattttgaaccgggacttgaaccgttcacgcttaatggc

gattacattgttgattctctgccatcgctcatctataccgatttcaaacgcctttctcct

tactacgactttatctccgtcgggcgcacgcgtattgctgatcgtctttgcgaagtcatt

cgcgtggttgcccgagatggtacacgctacagctacatcgtgtggatggacaccgaatcg

aaattaccgatgcgggttgatcttcttgatcgcgatggtgaaacgctggaacaatttcgc

gtgattgcttttaacgtcaatcaggatatcagcagcagtatgcagacgctggcgaaggca

aatttgccgccgttgctttctgttcctgtaggtgaaaaagctaaattcagctggacgcca

acctggttgccacagggttttagcgaagtttccagtagtcgacgtccgctaccgacgatg

gacaacatgcctatcgaatcacgtctctattccgacggattattcagcttctcggtaaac

gttaaccgcgctacgccatcgagcaccgatcagatgttgcgcaccggacgcagaaccgtc

agtacaagcgtccgtgataacgccgaaatcaccattgtcggtgaactgccgccgcaaacg

gcgaaacgcattgccgagaatattaagttcggggcagcgcaatgaatgaataccgtttgt

acccattgtcaggccatcaatcgcattcccgacgatcggatcgaagatgcggcaaaatgc

ggacgctgcggtcacgacttgtttgacggagaggtgattaatgcgaccggtgaaacgctc

gacaaattgctgaaagatgatctaccagtggtgatcgacttctgggcaccgtggtgcggc

ccctgccgtaatttcgcaccaatttttgaagatgtcgcgcaagagcgtagcggtaaagtg

cgctttgtgaaagtgaataccgaagctgaacgtgaattaagcagccgctttggtattcgc

agtattccgacgatcatgattttcaaaaacggtcaggttgtcgacatgcttaatggcgca

gtgccgaaagcgccgttcgatagctggctgaacgaatctctttaaatggcacaacgagta

cagctcactgcaacggtgtccgaaaaccaactcggtcaacgcttagatcaggctttggcc

gaaatgttcccggattattcacgttcgcgaataaaagaatggatcctcgaccagcgcgtg

ctggtaaacggcaaagtttgtgataagccgaaagaaaaagtattgggtggcgagcaggtt

gccatcaacgctgagattgaagaagaagcgcgttttgaaccgcaggatatcccgctggat

atcgtctatgaagatgaagatatcatcgtcattaataaaccgcgcgatctggtggtacat

cctggcgcgggtaacccggatggcacggtactgaatgcgttacttcattactatccgccc

attgctgatgtaccgcgtgcaggcatcgtccatcgtctggataaagacaccactggcctg

atggttgtggcaaaaaccgttccggctcagacgcgtttagtcgaatctttgcaaaggcgt

gaaattactcgtgagtatgaagcggtggcaattggtcatatgaccgctggcggtacggtg

gatgagccaatcagtcgccacccgaccaaacgtactcacatggcggtgcatccgatgggc

aaaccagcggtgactcactatcgcatcatggaacacttccgtgtgcacacgcgtctgcgg

ttgcgtctggaaactggacgtacgcaccagatccgcgtgcatatggcccatatcactcat

ccgctggtgggcgatccggtttatggtggccgtccgcgtccgccaaaaggtgcttcggaa

gcgtttatctccacgctgcgtaagtttgaccgccaggcgcttcatgcaaccatgctgcgt

ctttatcacccgatctccggcatcgaaatggaatggcatgcgcctattccacaagatatg

gtggagctgattgaggcgatgcgtgccgatttcgaagaacataaggatgaagtggactgg

ttatgaatgacgcgcatgaaatatctggtggcagccgccacactaagcctgtttttggcg

ggttgctcggggtcaaaggaagaagtacctgataatccgccaaatgaaatttacgcgact

gcacaacaaaagctgcaggacggtaactggagacaggcaataacgcaactggaagcgtta

gataatcgctatccgtttggtccgtattcgcagcaggtgcagctggatctcatctacgcc

tactataaaaacgccgatttgccgttagcgcaggctgccatcgatcgttttattcgcctt

aacccgacccatccgaatatcgattatgtcatgtacatgcgtggcctgaccaatatggcg

ctggatgacagtgcgctgcaagggttctttggcgttgaccgtagcgatcgcgatcctcaa

catgcacgagctgcgtttagtgacttttccaaactggtgcgcggctatccgaacagtcag

tacaccaccgatgccaccaaacgtctggtattcctgaaagatcgtctggcgaaatatgaa

tactccgtagccgagtactatacagaacgtggcgcatgggttgccgtcgttaaccgcgta

gaaggcatgttgcgcgactacccggatacccaggctacgcgtgatgcgctgccgctgatg

gaaaatgcataccgtcagatgcagatgaatgcgcaagctgaaaaagtagcgaaaatcatc

gccgcaaacagcagcaatacataaatgaataatcatttcaagtgtattggcattgtggga

cacccacggcaccccactgcactgacaacacatgaaatgctctaccgctggctgtgcaca

aaaggttacgaggttatcgtcgagcagcaaatcgctcacgaactgcaactgaagaatgtg

aaaaccggcacactcgccgagattgggcaactggcagatctcgcagtagttgttggtggc

gacggtaatatgctcggcgcagcgcgcacgctcgcccgctacgatattaaagttattgga

atcaaccgtggcaacctgggtttcctgactgaccttgatcccgataacgcccagcaacag

ttagccgatgtgctggaaggtcactacatcagcgaaaaacgttttttgctggaagcgcaa

gtctgccagcaagattgccagaaacgcatcagcacggcgattaacgaagtggtacttcac

cctggcaaagtggcgcatatgattgagtttgaagtgtatatcgacgagatctttgcgttt

tcgcagcgatctgatggcctgattatttcgacgccaacaggctccaccgcctattccctc

tcagcaggcggtccaatactgacgccttctctggatgcgattaccctggtgcccatgttc

ccgcatacgttgtcagcacgaccactggtcataaacagcagcagcacgatccgtctgcgt

ttttcgcatcgccgtaacgacctggaaatcagttgcgacagccagatagcactgccgatt

caggaaggtgaagatgtcctgattcgtcgctgtgattaccatctgaatctgattcatccg

aaagattacagttatttcaacacattaagtaccaagctcggctggtcaaaaaaattattc

taaatgacgaagaaaaaagcacataaacctggttcagcgaccatcgcgcttaacaagcgc

gcccgtcacgaatactttatcgaagaagagttcgaagcgggacttgccctgcaaggctgg

gaagttaaatccctgcgcgcaggaaaagccaatatcagcgacagctacgtccttctgcgt

gacggagaggcatttctgtttggcgctaacatcacgccaatggccgtagcctccacgcat

gtggtgtgcgatcctacccgtacccgcaagttacttctcaaccagcgcgaactggactca

ttgtacggtcgcgtcaatcgagaaggctataccgtagtggcgctctccctgtactggaaa

aatgcctggtgcaaagtgaaaatcggcgtcgccaaaggtaagaaacagcacgataaacgt

tcagatatcaaagagcgcgaatggcaggtggataaagcgcgtatcatgaaaaacgcccac

cgttaaatgggtctgttcaattttgtgaaagatgccggagaaaaactctgggacgcggtt

acaggtcagcacgataaagacgatcaggcgaagaaggtgcaggagcatctgaacaaaacc

ggtatacctgatgccgataaagtgaatattcaaattgccgacggcaaagcgacggtcact

ggtgacggtcttagtcaggaggcgaaagagaaaatccttgttgcggtggggaatatttcc

ggcattgccagtgttgatgatcaggtgaaaacggcgacaccagccactgccagccagttt

tataccgttaagtctggcgacactctgagtgccatttccaaacaggtctacggtaacgct

aatctgtacaataaaatcttcgaagcgaataaaccgatgctaaaaagcccggataaaatt

tatccggggcaagtgttgcgtattccggaagagtagatgactgaactcgcgcaattacag

gctagtgccgaacaggcagcggccttattgaaagcaatgagccaccctaaacggttgctg

attctgtgcatgcttagcggttcccccggcaccagcgcgggagagctgacgcgcattacc

ggactgagtgcctctgcgacatcacagcatctcgcccgtatgcgggacgaagggcttatc

gacagccaacgggatgcccaacgcattctatattccattaaaaatgaggcggtaaatgcc

attatcgccaccctgaaaaatgtctattgtccgtaaatgtccgtaatgttacaaagttta

aataacattcgcaccctccgtgcgatggctcgcgaattctccattgacgttcttgaagaa

atgctcgaaaaattcagggttgtcactaaagaaagacgtgaagaagaagaacagcagcag

cgtgaactggcagagcgccaggaaaaaattagcacctggctggagctgatgaaagctgac

ggaattaacccggaagagttattgggtaatagctctgctgctgcaccacgcgctggtaaa

aaacgccagccgcgtccggcgaaatataaattcaccgatgttaacggtgaaactaaaacc

tggaccggtcagggccgtacaccgaagccaatcgctcaggcgctggcagaaggtaaatct

ctcgacgatttcctgatctaaatgttctcaccgcagtcacgcttgcgtcatgcagttgca

gatacgttcgcgatggttgtttactgttctgtcgtgaacatgtgtattgaagttttcctc

tccggaatgagcttcgaacagtctttttattccagattggtagcgattccggtgaacatc

ttaattgcatggccatacggtatgtaccgtgatctgtttatgcgcgcggcacgcaaagtt

agcccgtcgggctggataaaaaatctggcggatatcctggcttatgtgacgttccagtca

ccggtgtatgtggcgatcttgttagtggtgggcgcagactggcatcagattatggcggcg

gtcagttcaaacatcgttgtttcgatgttgatgggggcggtttatggctacttcctcgat

tattgccgccgactgtttaaagtcagccgttaccagcaggtaaaagcctgaatggaaagc

cctactacacagcctgctcctggttcggcgaccttcatggaaggatgcaaagacagttta

ccgattgttattagttatattccggtggcctttgcgttcggtctgaatgcgacccgtctg

ggattctctcctctcgaaagcgtttttttctcctgcatcatttatgcaggcgcgagccag

ttcgtcattaccgcgatgctggcagcagggagtagtttgtgggttgctgcactgaccgtc

atggcaatggatgttcgccatgttttgtatggcccgtcactgcgtagccgtattattcag

cgtctgcaaaaatcgaaaaccgccctatgggcgtttggcctgacggatgaggtttttgcc

gccgccaccgcaaaactggtacgcaataatcgccgctggagcgagaactggatgatcggc

attgccttcagttcatggtcatcgtgggtctttggtacggtaataggggcattctccggc

agcggcttgctgcaaggttatcccgccgttgaagcagcattaggttttatgcttccggca

ctctttatgagtttcctgctcgcctctttccagcgcaaacaatctctttgcgttaccgca

gcgttagttggtgcccttgcaggcgtaacgctattttctattcccgtcgccattctggca

ggcattgtctgtggctgcctcactgcgttaatccaggcattctggcaaggagcgcccgat

gagctatgaatgagctatgaggttctgctgcttgggttactggttggcgcggcgaattat

tgcttccgctatttgccgctgcgcctgcgtgtgggtaatgcccgcccaaccaaacgtggc

gcggtaggtattttgctcgacaccattggcatcgcctcgatatgcgctctgctggttgtc

tctaccgcaccagaagtgatgcacgatacacgccgtttcgtgcccacgctggtcggcttc

gcggtactgggtgccagtttctataaaacacgcagcattatcatcccaacactgcttagt

gcgctggcctatgggctcgcctggaaagtgatggcgattatataaatgccgttgttagat

agcttcacagtcgatcatacccggatggaagcgcctgcagttcgggtggcgaaaacaatg

aacaccccgcatggcgacgcaatcaccgtgttcgatctgcgcttctgcgtgccgaacaaa

gaagtgatgccagaaagagggatccataccctggagcacctgtttgctggttttatgcgt

aaccatcttaacggtaatggtgtagagattatcgatatctcgccaatgggctgccgcacc

ggtttttatatgagtctgattggtacgccagatgagcagcgtgttgctgatgcctggaaa

gcggcaatggaagacgtgctgaaagtgcaggatcagaatcagattccggagctgaacgtc

taccagtgtggcacttaccagatgcactcgttgcaggaagcgcaggatattgcgcgtagc

attctggaacgtgacgtgcgcatcaacagcaacgaagaactggcgctgccgaaagagaag

ttgcaggaactgcacatttagatgacagaatcaacgtcccgtcgcccggcatatgctcgc

ctgttggatcgtgcggtacgcattctggcggtgcgcgatcacagtgagcaagaactgcga

cgtaaactcgcggcaccgattatgggcaaaaatggcccagaagagattgatgctacggca

gaagattacgagcgcgttattgcctggtgccatgaacatggctatctcgatgacagccga

tttgttgcgcgctttatcgccagccgtagccgcaaaggttatggacctgcgcgtattcgc

caggaactgaatcagaaaggtatttcccgcgaagcgacagaaaaagcgatgcgtgaatgt

gacatcgactggtgcgcactggcgcgcgatcaggcgacgcgaaaatatggtgaacctttg

ccaactgtcttttcagaaaaagttaagatccagcgttttctgctctatcgtggctatctg

atggaagatatccaggatatttggcgaaattttgccgactgaatgtgcataggcgttccc

ggccagatccgcaccattgacggcaaccaggcgaaagtcgacgtctgcggcattcagcgc

gatgtcgatttaacgttagtcggcagctgcgatgaaaacggtcagccgcgcgtgggccag

tgggtactggtacacgttggctttgccatgagcgtaattaatgaagccgaagcacgcgac

actctcgacgccttacaaaacatgtttgatgttgagccggatgtcggcgcgctgttgtat

ggcgaggaaaaataaatgcgaattggacacggttttgacgtacacgcctttggcggtgaa

ggcccaattatcattggtggcgtacgcattccttacgaaaaaggattgctggcgcattct

gatggcgacgtggcgctccatgcgttgaccgatgcattgcttggcgcggcggcgctgggg

gatatcggcaaactgttcccggataccgatccggcatttaaaggtgccgatagccgcgag

ctgctacgcgaagcctggcgtcgtattcaggcgaagggttatacccttggcaacgtcgat

gtcactatcatcgctcaggcaccgaagatgttgccgcacattccacaaatgcgcgtgttt

attgccgaagatctcggctgccatatggatgatgttaacgtgaaagccactactacggaa

aaacttggatttaccggacgtggggaagggattgcctgtgaagcggtggcgctactcatt

aaggcaacaaaatgaatgggtaaactaacgctgctgttgctggctattctggtctggcta

cagtattcgctgtggttcggtaagaacggtatacatgactatacccgcgtcaatgatgat

gtggcggcacagcaagctacaaacgcgaaacttaaagcgcgaaacgatcaactttttgcc

gaaattgacgatctcaatggcggccaggaggcgctcgaagagcgtgcgcgtaatgaactc

agcatgaccaggccgggcgaaactttttatcgtctggtgcctgacgcgtcgaagcgcgca

cagtctgcggggcaaaacaatcgataaatgcgtaatagccataacattacactaacaaat

aacgacagccttactgaggatgaagaaaccacatggtcactgcctggtgccgtggtcggt

tttatctcttggttatttgcgctggcgatgccgatgttgatttatggctctaacacgctg

ttcttctttatctacacctggcctttctttcttgcgctgatgcccgtcgcggtagtggtg

gggattgcgctgcattcattgatggacggaaagctacgctacagtattgttttcaccctg

atgacagtaggcattatgtttggcgcactgtttatgtggctactgggctaaatggcgctg

catgacgaaaacgtcgtctggcatagccatccggtcactgtgcaacaacgcgagctacac

cacggtcatcgtggtgtagtgctgtggtttaccggcctctccgggtccggtaaatcaacg

gtcgccggggcgctggaggaggcgttacataaactcggcgtcagtacgtatctgctggat

ggcgacaatgttcgccacggattatgcagcgatctcggttttagcgatgccgatcgtaaa

gagaatatccgtcgcgtcggtgaagtggcgaatttgatggttgaagccggactggtggtg

ctgaccgcatttatctcgccacaccgcgccgaacgccagatggttcgtgaacgcgtagga

gaagggcgctttatcgaagtgtttgtcgatacgccgctggcgatttgcgaagcccgcgat

ccaaaaggcttatataagaaagcgcgtgccggtgaactgcgcaactttacgggaatagat

tccgtttacgaagcgcctgaatcggcagaaattcatctcaatggtgaacaattagtaaca

aatttggtacagcaattattagatctgttgagacagaacgatattatcagatcctgaatg

gaaacgactcaaaccagcacgattgcgtcgaaagactctcgtagtgcctggcgcaagaca

gacaccatgtggatgctgggcctttacggcacggcaatcggcgcgggcgtgctgttcctg

ccaatcaacgccggtgttggcggtatgatcccgctgatcatcatggctatccttgcgttc

ccgatgacgttttttgctcaccgcggcctgactcgcttcgtactgtctggtaaaaacccg

ggcgaagacatcaccgaggttgtagaagaacactttggtattggcgcaggtaaactgatt

accctgctctacttcttcgctatctacccgatcctgctggtttatagcgtggcaatcacc

aatacagttgaaagcttcatgtctcaccagctgggtatgacgccaccgccgcgtgcgatt

ctgtcgctgatcctgatcgtgggtatgatgaccatcgttcgcttcggtgagcagatgatc

gttaaagcaatgagtattctggtattcccgtttgttggcgtactgatgctgctggctctg

tacctgatcccgcagtggaacggcgcagcactggaaacgctgtctctggacactgcatct

gcaaccggaaacggtctgtggatgaccctgtggctggcaattccggtaatggtgttctcg

ttcaaccactctccgatcatctcttctttcgccgttgcgaagcgtgaagagtacggcgat

atggcagaacagaaatgctcgaagatcctggcattcgcacacatcatgatggtgctgacc

gtaatgttcttcgtcttcagctgcgtactgagcctgactccggcagacctggctgcggct

aaagagcagaacatctcgattctgtcttacctggctaaccactttaacgcaccggttatc

gcgtggatggctccgattatcgcgattatcgctatcaccaaatccttcctcggccactac

ctgggcgcacgtgaaggcttcaacggtatggtgattaaatctctgcgtggtaaaggtaag

tctatcgaaatcaacaagctgaaccgtatcactgcgctgttcatgctggtaacgacctgg

attgttgccaccctgaacccgagcatcctgggtatgattgaaaccctgggcggcccaatc

atcgcgatgatcctgttcctgatgccgatgtacgcaattcagaaagtaccggcaatgcgt

aagtacagcggtcacatcagcaacgtattcgttgtcgtgatgggtctgattgcaatctcc

gcaatcttctactctctgttcagctaaatggaacgaaataaacttgctcgtcagattatt

gacacttgcctggaaatgacccgcctgggactgaaccaggggacagcggggaacgtcagt

gtacgttatcaggatgggatgctgattacgcctacaggcattccatatgaaaaactgacg

gagtcgcatattgtctttattgatggcaacggtaaacatgaggaaggaaagctcccctca

agcgagtggcgtttccatatggcggcctatcaaagcagaccggatgccaatgcggttgtt

cacaatcatgccgttcattgcacagcagtttccattcttaaccgaccgatccccgctatt

cactacatgattgcggcggctggcggtaattctattccttgcgcgccttatgcgaccttt

ggaacacgcgaactttctgaacatgttgcgctggctctcaaaaatcgtaaggcaactttg

ttacaacatcatgggcttatcgcttgtgaggtgaatctggaaaaagcgttatggctggcg

catgaagttgaagtgctggcgcaactttacctgacgaccctggcgattacggacccggtg

ccagtgctgagcgatgaagagattgccgtagtgctggagaaattcaaaacctatgggtta

cggattgaagagtaaatgctgaaaacaatttcgccgttaatttctcccgaactattgaaa

gtgctggcagagatgggacatggagatgaaattattttttccgatgctcactttcccgcc

cattcgatgggaccgcaggtgatccgcgctgatggcctgttggtgagcgacttgctccag

gcgattatcccgttatttgagctggacagttatgcaccgccgctggtgatgatggcagcg

gtagaaggtgacactctcgatcctgaagtagaacgacgttaccgtaatgcgctttcacta

caagccccctgtcct----------------------------gacatcatccgcatcaa

tcgttttgcgttttatgaacgggcgcaaaaagcctttgcgatcgttatcacaggcgaacg

agcgaagtacgggaatattcttttaaaaaaaggggtaacaccgtaaatgtcccaacctcg

cccactgctctctcctcccgaaactgaagaacagttgttagcgcaagcacagcaactttc

tggttatacattgggagaactggcggcacttgccgggctggttacgccagagaatttaaa

acgcgataagggctggattggcgtgttactggagatctggctaggtgccagcgcagggag

taaacctgagcaagattttgctgctctgggcgtggaacttaaaactatccctgtggatag

tcttggtcgtccgctggaaacaacattcgtttgtgttgccccgttaacgggcaatagcgg

agtgacctgggaaaccagccacgtgcgccacaaactcaaacgcgtactgtggataccggt

tgaaggcgagcgcagcatcccgctggcgcagcgtcgcgttggatcaccgttgctgtggag

cccgaatgaagaggaagaccggcagctacgcgaagactgggaagaattaatggatatgat

tgttctcggtcaggttgagcggattaccgctcgtcacggggagtatttacagatacgacc

gaaagcagcgaatgcgaaagcgcttaccgaagccattggtgcccggggcgaacggattct

gacgctgccgcgcggcttttatttgaagaaaaatttcaccagtgcgctgctggcccgtca

ttttctgatccagtagatgttatttgcatggataaccgatcctaacgcctggcttgcgct

cggtacgctgacgctgctggagatcgttcttgggatcgacaatattattttcctttctct

ggtggtggcaaagctccccacagcacaacgtgctcatgcgcgccgtctggggttggcggg

agccatggttatgcgtctggcgctgctggcatcaatcgcctgggttacgcgcctgacgaa

tccgctttttacaatattcagtcaggaaatttccgcccgtgatttgattctgcttctggg

tggcttgttccttatctggaaagccagcaaggaaatccacgaatccattgaaggtgaaga

agaagggctgaaaacacgcgtttcatcattcctcggcgctatcgtgcagattatgctgct

ggatattatcttcagcctcgactcggtgattaccgctgtgggtctgtcagatcacctgtt

tattatgatggccgccgtggtaattgccgtaggcgtgatgatgttcgccgcgcgctcgat

tggtgattttgtcgagcgccatccttcggtaaaaatgctggcgctctctttcctgattct

ggtgggctttaccctgattctggaaagtttcgacatccacgtaccgaaaggttacatcta

cttcgcgatgttcttctctattgcggttgaaagcctcaacctgattcgcaacaaaaagaa

tccgctctgaatgaatatgggtcttttttacggttccagcacctgttacaccgaaatggc

ggcagaaaaaatccgcgatattatcggcccagaactggtgaccttacataacctcaagga

cgactccccgaaattaatggagcagtacgatgtgctcattctgggtatcccgacctggga

ttttggtgaaatccaggaagactgggaagccgtctgggatcagctcgacgacctgaacct

cgaaggtaaaattgttgcgctgtatgggcttggcgatcaactgggatacggcgagtggtt

cctcgatgcgctcggtatgctgcatgacaaactctcgaccaaaggcgtaaagttcgtcgg

ctactggccaacggaaggatatgaatttaccagcccgaaaccggtgattgctgacgggca

actgttcgtgggtctggcgctggatgaaactaaccagtatgaccttagcgacgagcgtat

tcagagctggtgcgagcaaatcctcaacgaaatggcagagcattacgcctgaatgaacga

tattgcgcataacctggcacaggtccgggacaaaatctcagcggctgcaacgcgttgcgg

ccgttctccagaagaaattacgctgcttgcagtcagtaaaacaaaacctgcgagcgccat

cgcagaagccattgatgccgggcagcgtcaatttggtgaaaactacgttcaggaaggggt

agataaaattcgccactttcaggaactgggcgtaacaggattagaatggcattttattgg

cccgttgcagtctaataaaagccgcctggtggcagagcatttcgactggtgtcataccat

cgaccgtttgcgcatcgctacccgcctcaacgatcagcgcccggcagaacttccccctct

taacgtgctgattcaaattaatattagtgatgaaaacagtaagtccgggattcaactggc

agaactggacgagctggcagctgcggtcgctgaactaccgcgtttacgtctgcgcgggct

gatggcaatccctgcgcctgagtcagaatatgtaaggcagtttgaagttgcacgccaaat

ggctgtagcatttgccggactgaaaacgcgctacccgcatatcgacacgctctctctggg

aatgtcggacgatatggaagccgccattgcggcaggtagcacgatggttcgtatcggcac

tgcaatttttggtgcgcgtgattactctaaaaaataaatgaatacgttgactttcctgct

ttcaacggtcattgagctgtataccatggtgctgttattacgcatctggatgcagtgggc

tcattgtgatttttacaaccccttctcacagtttgtagtgaaggtaacacagccaattat

cgggccactgcgccgcgttattccggcaatgggacccattgacagcgcctcgctgctggt

tgcctatattctcagttttatcaaagccattgtgctgtttaaagtggtgaccttcctgcc

aatcatctggattgccggtttactgattctgctgaaaaccatcggcctgctgattttctg

ggtcctgctggtgatggcgattatgagctgggtaagccagggtcgtagcccgattgaata

cgtgctgattcagctggccgatccgctgctgcgcccgattcgccgcctgctaccagcaat

gggtgggattgatttctcgccgatgatcctcgttctgctgctgtatgtcatcaatatggg

tgtcgcagaagtattacaggcaaccggaaacatgctgctgccggggctgtggatggcgtt

atgaatgcaaaaagttgtcctcgcaaccggcaatgccggtaaagtgcgtgagctggcgtc

gctgcttagcgacttcggtcttgatatcgtggcccaaacagacctcggcgtagattccgc

tgaagaaaccggcctgacctttatcgaaaacgcgattctgaaagcgcgccatgcggcaaa

agtgaccggtttaccggcaattgccgacgactccggtctggcggtagatgtacttggcgg

tgcgccggggatttactccgcgcgttattccggtgaagacgcgaccgatcaaaagaatct

gcaaaaactgctggaaacactgaaagacgtaccggacgaccaacgtcaggcgcgtttcca

ctgcgtgctggtatatctgcgtcacgcggaagatccgactccgctggtgtgccacggtag

ttggccgggcatgattactcgtgaaccagcgggtactggcggctttggttatgatccaat

cttcttcgtaccttccgaagggaaaaccgctgccgaactgacccgcgaagaaaagagcgc

catttcccaccgtggtcaggcattgaaactgctgctggacgctttacgtaatggttaaat

gaaaaacgacgtcatttcaccggaatttgatgaaaacggccgcccactgcgccgtatccg

tagttttgtgcgccgccaggggcgactgaccaaaggccaggaacatgcgctggaaaacta

ctggccggtgatgggcgttgagttcagcgaagatatgctggatttccccgcgctttttgg

ccgtgaagcgccggtgacgcttgagattggttttggcatgggggcgtcgctggtggcaat

ggctaaagatcgccccgagcaggacttcttcggcattgaagtgcattcaccgggcgttgg

tgcgtgcctggcttctgcgcatgaagagggcttaagcaacctgcgcgtgatgtgtcacga

tgcggttgaagtgctgcataaaatgattcctgacaattcattgcgcatggtgcagctctt

tttccctgacccgtggcacaaagcgcgccataataaacgccgtatcgttcaggtgccgtt

tgccgaactggtaaaaagcaaactgcagctggggggcgtattccatatggcgaccgactg

ggaaccttatgcggaacatatgcttgaagtgatgtcttctattgacggttataaaaacct

gtcagagagcaatgattacgtaccgcgtccggcatcacgtccggtgacgaaatttgaaca

acgtggtcatcgtcttggtcacggagtatgggacttaatgttcgagagggtgaaataagt

gaacagacgtaattttattaaagcagcctcctgcggggcattgctgacgggcgcgttgcc

gtctgtcagtcatgcggctgctgaaaaccgcccgccaattccgggatcgctggggatgtt

gtacgactcgaccttgtgcgtaggctgccaggcttgcgtcaccaagtgtcaggatatcaa

cttccctgaacgtaacccgcgaggggaacagacctggtcgaacaacgacaaactatcgcc

gtataccaataacatcattcaggtgtggaccagcggcactggcgtcaacaaagaccagga

ggagaacggctacgcgtacattaagaaacagtgtatgcactgcgtcgatccgaactgtgt

ctctgtgtgcccggtttctgcactgaaaaaagatccgaaaaccggcattgtccattacga

caaagacgtgtgcaccggctgccgttactgcatggtcgcctgtccgtacaacgtgccgaa

gtacgactacaacaacccgtttggtgcgctgcataagtgcgagctgtgcaaccagaaagg

tgtggaacgtctcgataaaggcggtctgcctggctgcgtagaagtgtgcccggcgggcgc

ggtgatttttggtacgcgtgaagagctgatggcggaggcgaaaaaacgtctggcgctgaa

gcctggcagcgaataccactatccgcgtcagacgctgaaatctggcgacacttacctgca

tacggtgccgaaatattatccgcatctgtacggcgagaaagagggcggcggtactcaggt

tctggtactgacgggtgtgccttatgaaaatctcgacctgccgaaactggacgatctttc

taccggtgcgcgttccgaaaatattcaacacaccctgtataaaggcatgatgctaccact

ggctgtgctggcgggcttaaccgtgctggttcgtcgcaacaccaaaaacgaccatcacga

cggaggagacgatcatgagtcatgaatgaataaccattttggtaaaggcttaatggcggg

attaaaagcaacgcatgccgacagtgcggttaatgtgacaaaattctgtgccgattataa

acgcggttttgtattaggctactcacaccggatgtacgaaaagaccggagatcgccagct

tagcgcctgggaagcgggtattctgacgcgccgctatggactggataaagagatggtaat

ggatttctttcgtgagaataattcctgttctacgttgcgcttttttatggccggttatcg

cctcgaaaattgaatggcaatgcatcttaacgaaaacctcgatgataacggcgaaatgca

tgatatcaacgtgacgccgtttatcgacgtgatgttggttctgctgattatctttatggt

ggcggcaccgttagcgacggtagatgtgaaggttaacttgcctgcttctaccagcacgcc

gcagccgcgcccggaaaaaccggtttatctgtcggtgaaggcagacaactcgatgtttat

cggtaacgatccggtcaccgatgaaacaatgattacggcgttgaatgcgttaaccgaagg

caagaaagacaccaccatcttcttccgagcggataaaaccgtcgattacgagacgttgat

gaaggtaatggatacgctgcatcaggcgggttacctgaagataggtctggtcggcgaaga

aaccgccaaagcgaagtaaatggctgttattcaagatatcatcgctgcgctctggcaaca

cgactttgccgcgctggcggatcctcatattgttagcgttgtttactttgtcatgtttgc

cacgctgtttttagaaaacggcctgctgcccgcctcatttttgccaggcgacagcttgtt

gatattggctggcgcattgattgcccagggggttatggattttctgcctacgattgcgat

tctgaccgccgcagcaagtctgggctgctggctaagttatattcaggggcgctggttagg

gaataccaaaacggtgaaaggctggctggcacagcttcctgctaaatatcatcagcgcgc

cacctgcatgtttgaccgccacggtctgctggcgctgctggctggacgttttcttgcatt

tgtccgtacgctgctgccaaccatggcgggaatttccggtctgccaaaccgccgtttcca

gtttttcaactggttaagcggattgctgtgggtcagcgtggtaaccagtttcggctatgc

cttaagtatgattccgttcgttaaacgccatgaagatcaggtaatgacgttcctgatgat

cctgccaattgccttgttaaccgctggcttgttaggcacgctgtttgtggtgattaaaaa

aaaatactgtaacgcctgaatgaacaactttaatctgcacaccccaacccgcattctgtt

tggtaaaggcgcaatcgctggtttacgcgaacaaattcctcacgatgctcgcgtattgat

tacctacggtggcggcagcgtgaaaaaaaccggcgttctcgatcaagttctggatgccct

gaaaggcatggacgtgctggaatttggcggtattgagccaaacccggcttatgaaacgct

gatgaacgccgtgaaactggttcgcgaacagaaagtgactttcctgctggcggttggcgg

cggttctgtactggacggcaccaaatttatcgccgcagcggctaactatccggaaaatat

cgatccgtggcacattctgcaaacgggcggtaaagagattaaaagcgccatcccgatggg

ctgtgtgctgacgctgccagcaaccggttcagaatccaacgccggtgcggtgatctcccg

taaaaccacaggcgataagcaggcgttccattcagcccatgttcagccggtatttgccgt

gctcgatccggtttatacctacaccctgccgccgcgtcaggtggctaacggcgtagtgga

cgcctttgtacacaccgtggaacagtatgttaccaaaccggttgatgccaaaattcagga

ccgtttcgcagaaggcattttgctgacgctgatcgaagatggtccgaaagccctgaaaga

gccagaaaactacgatgtgcgcgccaacgtcatgtgggcggcgacgcaggcgctgaacgg

tttgattggcgctggcgtaccgcaggactgggcaacgcatatgctgggccacgaactgac

tgcgatgcacggtctggatcacgcgcaaacactggctatcgtcctgcctgcactgtggaa

tgaaaaacgcgataccaagcgcgctaagctgctgcaatatgccgaacgcgtctggaacat

cactgaaggttccgacgatgagcgtattgacgccgcgattgccgcaacccgcaatttctt

tgagcaattaggcgtgccgacccacctctccgactacggtctggacggcagctccatccc

ggctttgctgaaaaaactggaagagcacggcatgacccaactgggcgaaaatcatgacat

tacgctggatgtcagccgccgtatatacgaagccgcccgctaaatgaaaacgattttcac

cgtgggagctgttgttctggcaacctgcttgctcagtggctgcgtcaatgagcaaaaggt

caatcaactggcgagcaatgtacaaacattaaatgccaaaatcgcccggcttgagcagga

tatgaaagcactacgcccacaaatctatgctgccaaatccgaagctaacagagccaatac

gcatcttgatgcacaggactattttgattgcctgcgctgcttgcgtatgtacgcagaatg

aatgtcactcagtcggcgtcagttcattcaggcatcggggattgcactttgtgcaggcgc

tgttcccctgaaggccagcgcagccgggcaacagcaaccgctacccgttccgccgctgct

tgaatctcgccgtgggcaaccgctgtttatgactgtacaacgtgcgcactggtcatttac

gccagggacacgcgcgtcggtctggggaatcaatggtcgttacctggggccgactatccg

cgtctggaagggcgacgatgttaagcttatttacagcaaccgcctgacagaaaatgtctc

aatgacggtggccgggctacaggtaccaggcccgctgatgggcggtccggcacggatgat

gtcgccaaacgctgactgggcacccgtactgcctattcgccagaacgcagctactctgtg

gtatcacgccaatactcccaaccgcacggctcagcaggtctataacggccttgccggaat

gtggctggtggaagatgaagtcagcaagtcgctgcctatccccaaccattatggtgtgga

tgattttccggtcattatccaggataaacggctggataactttggtacgccagaatacaa

cgaaccgggaagcggcggctttgttggtgatacgctgctggttaacggtgtacaaagccc

gtacgttgaagtctcgcgtggctgggtgcgcttgcgactgctgaacgcgtcgaactctcg

tcgctatcaactacagatgagcgatggtcgcccgttacatgtgatttctggcgatcaggg

attcctgcctgctcctgtatcggtgaagcaactttcgttggcaccgggcgagcgccgcga

gattctggtggatatgagcaacggtgatgaagtgtcgatcacctgtggcgaagcggcgag

cattgttgatcgtattcgtggcttctttgagccatccagcattctggtttctaccctggt

gctaacgctgcgcccaaccggccttctgccgctggtcacagacagtcttccgatgcgcct

gctgccaactgaaatcatggccggttcgccgattcgcagtcgcgatatcagtctgggtga

tgacccgggtattaatggacaactgtgggacgtcaaccgtattgatgtcaccgcgcagca

aggaacgtgggaacgctggacggtacgcgcggacgagccgcaagcgttccatattgaagg

cgtgatgttccagatccgtaacgtgaatggtgcgatgccgttcccggaagacagaggctg

gaaagataccgtttgggttgacggacaagtggagctgcttgtttatttcggtcagccttc

ctgggcgcacttcccgttctacttcaacagtcagacgctggaaatggcggaccgtggctc

gattgggcaactgttagtcaatccggtaccgtaaatgctatatatctttcgtcttattat

taccgtgatttacagcatcttagtctgtgtattcggctccatttactgccttttcagccc

gcgtaacccgaaacatgtggccacctttgggcatatgtttggtcgtcttgcgccgctgtt

cggcctgaaagttgagtgccgcaaacctgcagatgctgaaagctacggcaatgctatcta

tatcgctaaccaccagaacaactatgacatggtgacagcatcgaacattgtgcaaccgcc

gacggtgacggtaggtaaaaagagcttgctgtggatccccttcttcgggcagttgtactg

gttaaccggcaacttattgatcgacagaaacaatcgcactaaagctcacggcactattgc

ggaagtagtgaatcatttcaaaaaacgccgtatttccatttggatgttcccggaaggaac

ccgcagccgtggtcgcggcctgctaccgttcaagactggagcatttcacgcggcaattgc

agcaggcgtcccgattattcccgtgtgcgtctctacaacttcgaataagattaatcttaa

tcgactgcacaacggtctggtgattgtcgaaatgctgccgccaattgacgtcagtcagta

tggcaaagatcaggttcgtgagctggctgcccattgtcgttcgataatggaacaaaaaat

cgccgagctcgataaagaagtcgcagaacgcgaagccgccggaaaagtttaaatgtctac

gcttctttatttacacggtttcaacagctcgccgcgctctgcaaaagcgagcttgttaaa

aaactggctggcggaacatcaccctgacgttgagatgatcattccgcagttgccgccgta

tccttccgacgcggcagagctgctggaatccattgtcctggaacatggcggcgattcgct

gggtattgtcggttcgtcactggggggatattacgccacctggttgtcacaatgttttat

gctgcccgcagtggtggtaaacccggcggtgcgcccgtttgaactgctgacggactatct

cggtcagaacgagaacccctacaccgggcagcaatatgtgctagagtcacgccatattta

cgatcttaaagtcatgcagattgacccgctggaagcgccggatttgatctggctgctgca

acagacgggagatgaagtgctggattaccgccaggcggtggcgtactacgcttcctgccg

ccagactgtcatagaaggcggcaaccacgcattcacgggcttcgaagattatttcaaccc

gatcgtcgattttcttggtctgcaccatctctgaatgcttaagccagacaacctgcccgt

tacatttggcaaaaacgatgtagaaattattgcacgagaaacactttatcgcggcttttt

ttcattagatctttatagatttcgtcatcgtctattcaacgggcaaatgagtcatgaggt

acggcgggaaatttttgagcgcggtcacgccgcagtcttgctaccctttgacccagtgcg

tgatgaagttgtgctgattgagcagattcgaattgccgcatacgacaccagtgaaacccc

ctggctactggagatggttgccgggatgattgaagagggtgaaagtgtggaagatgttgc

ccgtcgcgaagcgattgaagaggcgggactgatagtcaaacggaccaaaccggtgttaag

tttcctggcaagcccggggggcaccagtgagcgttcgtcaattatggtgggcgaagtgga

cgccacgaccgcaagcggtattcatggtctggctgatgaaaacgaagatattcgcgttca

tgtggtaagccgggaacaggcataccagtgggtagaagaggggaaaatcgacaacgcagc

gtcggtcatcgctttgcaatggctgcagctgcatcatcaagcgttaaaaaatgagtgggc

ataaatgaatcagacgctactttcctcttttggtacgcctttcgaacgtgttgaaaatgc

actggctgcgctgcgtgaaggacgcggtgtaatggtgcttgatgatgaagaccgtgaaaa

cgaaggtgatatgatcttcccggcagaaaccatgactgttgagcagatggcgctgaccat

tcgccacggtagcggtattgtttgcctgtgcattactgaagatcgccgtaaacaactcga

tctgccaatgatggtagaaaataacaccagcgcctatggcaccggttttaccgtgaccat

tgaagcggctgaaggtgtgactaccggtgtttctgccgctgaccgtattacgaccgttcg

cgcagcgattgccgatggcgcaaaaccttctgatctgaatcgcccgggccacgttttccc

acttcgcgcgcaggcaggtggagtactgacgcgtggcggtcatactgaagcgactattga

tctgatgacgctggcaggctttaaaccggctggtgtgctgtgtgagctgactaatgacga

tggcacgatggcgcgtgcaccagagtgtattgagtttgccaataaacacaatatggcgct

cgtgactattgaagacctggtggcataccgtcaggcacatgagcgtaaagccagctgaat

gagtgcaatcgcgcctggaatgatcctcatcgcgtacctctgcggctccatttccagtgc

cattctggtttgccgcttgtgtgggctgcccgatccgcgaaccagcggctccggcaatcc

cggcgcaaccaatgtgttacgtatcggtggcaagggagcagccgtagcagtactgatttt

cgacgttctgaaaggaatgttgcctgtctggggcgcgtatgaattaggtgtcagcccctt

ctggctaggcttaattgccatcgccgcctgtcttggacacatctggcccgttttcttcgg

ttttaaaggaggaaaaggcgttgctaccgcttttggagcaatcgcaccaattggctggga

tctcaccggagtaatggcgggaacctggttactgaccgtgctaatgagcggatactcgtc

gctgggagcgattgtcagtgcactgattgctccgttttatgtctggtggtttaagccaca

attcaccttcccggtttcgatgctctcttgcctgatcctgctgcgtcatcatgacaacat

ccagcgtctgtggcgtcgtcaggagacaaaaatctggacgaaattcaaaagaaagcgcga

aaaggatcccgagtgaatggcaacactaaaagacatcgcaatcgaagctggcgtatccct

ggcgacagtatccagggtcttaaatgacgatccgacattgaatgtgaaagaagagacgaa

gcatcgcattctcgagatcgccgaaaagctggagtacaagaccagtagcgcccgtaaact

acagacaggcgcagtcaaccaacaccatattctggctatctacagctaccagcaggagct

ggagatcaacgatccttactatctggcgatccgccacggcattgaaactcagtgcgaaaa

gctggcgatcgaactgaccaactgttatgaacacaacggattaccagacattaaaaacgt

caccggtattttaattgtcggcaaacccacgcccgccctgcgcgccgccgcctgtgcgtt

gaccgacaatatctgttttatcgactttcacgaacccagcagcggttacgatgcggtgga

tatcgatctggcacgcatcagtaaagaaatcatcgacttctatatcaaccagggcgttaa

tcgcattggttttattggcggtgaagatgagcctggcaaggcggatattcgtgaggtcgc

ctttgcggaatatggccgactgaaacaagtggtacgcgaagaggatatctggcgcggcgg

tttttccagttcgtcgggttatgaactagcaaaacagatgctggcgcgggaagactatcc

gaaggcactgtttgttgcttccgattccattgctatcggcgtactgcgggcaattcatga

acgtggcctgaacatcccacaggatatttcgcttatcagcgttaacgatatccccaccgc

gcgatttacctttccgccgctctccaccgtgcgcatccattccgaaatgatgggaagtca

gggcgtaaacctggtgtatgaaaaagcccgcgatggtcgcgcgctgccgctgttagtctt

cgttcccagcaaattaaaactgcgcggcacgacccgttaaatggaacttttgacccaatt

gctgcaagccctgtgggcgcaggattttgaaaccctggccaatccatcgatgattggcat

gttgtattttgtcttgtttgtaattttgttccttgaaaacggcttgcttccggcggcctt

tttaccgggcgacagtttactggtattggtcggcgtgttgattgcgaaaggggcgatggg

ctatccgcaaacgattctgctgctgaccgttgccgccagcctcggctgctgggtcagcta

tattcaggggcgatggctgggcaatacccgcaccgtacaaaactggctatctcatttacc

cgcgcattatcatcaacgcgcacaccatctttttcataaacacggtttatcggcgctgtt

aattggtcgctttattgcgtttgtcagaacactgctgccgacgattgccgggttatcagg

gctgaataacgcgcgctttcagtttttcaactggatgagcggtctgctgtgggtattgat

cctgacaactctgggttacatgctcggcaaaacgccggtatttttaaagtacgaggacca

gctgatgtcatgcctgatgctgctcccggtggtgctgctggtttttggcctggcaggttc

tctggtcgtgttatggaaaaagaaatatggaaatcgggggtaaatgtcgaaagaacacac

tacggaacatctgcgtgctgagttgaaatccctttccgatacgctggaagaggtgcttag

ctcatctggcgagaagtcgaaagaagagttgagtaagattcgtagcaaagcggagcaggc

actgaaacagagccgttatcgcctgggtgaaaccggtgatgccatcgccaaacaaacccg

tgtcgcggcggcgcgtgccaatgagtatgtgcgtgaaaatccatggacgggcgtgggcat

tggcgctgcaatcggtgtagtgctcggcgttctgctgtcgcgtcgttaaatgattactac

ccgaactgccaggcagtgtggacaagcagactacggatggttgcaggcccggtatacttt

ttcctttggacactacttcgacccgaaattgttaggctatgcctccctgcgtgtgcttaa

ccaggaagtgctggccccaggtgccgcctttcagccgcgaacctatcccaaagtcgatat

tttaaatgtgattctggatggagaagcagagtatcgcgacagcgaaggcaatcatgttca

ggccagcgccggtgaggcgttgctgctctctacccagccgggtgtcagctacagcgaaca

caatctcagcaaagacaaaccgttaacacgaatgcagctttggctggacgcctgcccgca

gcgagagaatccgctgattcaaaaactggcgcttaatatgggcaagcagcaattaatcgc

ctcgccagagggtgcgatgggaagcctgcaattacgccagcaagtgtggctgcaccatat

cgtgctcgacaaaggcgaaagtgcgaatttccagttgcatgggccacgcgcgtatttgca

atcgattcacgggaaatttcatgcgcttacgcatcatgaagagaaagcggcgctgacctg

cggtgatggggcgtttattcgtgacgaggctaacattacgctggttgccgattccccact

gcgcgctttgctgatagatttgcctgtctagatgaagccttttttgcgctggtgtttcgt

tgcgacagcacttacgcttgcaggatgcagtaatacttcctggcgtaaaagtgaagtcct

cgcggtaccattgcaaccgactttacagcaggaagtgattctggcacgtatggaacaaat

ccttgccagtcgggctttaaccgatgacgaacgcgcacagcttttatatgagcgcggagt

gttgtatgatagtctcggtatgagggcattagcgcgtaacgatttttcgcaagcgctggc

aatccgaccggatatgcctgaagtattcaattacttaggcatatatttaacgcaggcagg

caattttgatgctgcctatgaagcgtttgattctgtacttgagcttgatccaacttacaa

ctacgcgcacttgaatcgcgggatcgcattatattacggcggtcgtgacaagttagcgca

agatgatctgctggcgttttatcaagacgatcccaatgatcctttccgtagtctgtggct

ttatctcgccgagcagaagctcgatgagaagcaggctaaagaagtgttgaaacagcactt

cgaaaaatcggataaggaacagtggggatggaacattgtcgagttctacctgggcaacat

tagcgaacaaacgttaatggaaaggctcaaggcggacgcaacggataacacctcgctcgc

tgagcatctcagtgaaaccaacttctatttaggtaagtactacctaagtctgggggattt

ggacagcgccacggcactgttcaaactggcggttgccaacaacgttcataactttgttga

gcaccgatacgcattgttggaattatcgctcctgggccaggaccaagatgacctggcaga

atcggaccagcaatagatgagtcgtcctcgtcgtcgcggtcgcgacattaacggcgtttt

gttgctggataaacctcagggtatgtccagcaacgatgcgctgcaaaaagtgaaacggat

ttataacgccaaccgtgccgggcataccggtgcgctggacccgctggcgaccggcatgtt

gccgatttgcctcggggaagcgacgaagttttcccagtatctgctggactccgacaaacg

ctatcgggtcattgcgcgtcttggacagcgtaccgatacttctgacgccgacggacaaat

cgttgaagagcgtccggtaacctttagtgcagaacaactggcggcggcactggatacttt

ccgtggcgatatcgaacagatcccatcgatgtattcagcactgaaataccagggcaaaaa

actgtacgaatatgcgcgtcagggcattgaagttccgcgtgaagcgcgtccgattaccgt

ttatgaattgctgtttattcgccatgaaggcaatgagctggagctggaaattcactgctc

aaaaggcacttatatccgcactatcattgacgacctgggtgagaaactcggctgtggcgc

gcatgttatttacctgcgccgtctggcggtaagtaaatatccgattgaacggatggtgac

cctggagcacctgcgtgaacttgttgagcaagctgaacagcaggatattccggctgcgga

gttacttgatccattactgatgccaatggacagtccagcttcggactatccggtggtgaa

tcttccgttaacgtcttctgtttacttcaaaaatggtaacccggttcgtacatctggtgc

gccgctggaaggactggttcgcgtcacggaaggtgagaacggcaaatttatcggtatggg

cgaaattgacgatgaaggccgcgttgcgcctcgtcgcctggtggttgaatacccggcgta

aatgacaggtaagaagcgttctgccagctccagccgctggcttcaggaacactttagcga

taaatatgttcaacaggcacagaaaaaggggttacgttcccgtgcctggtttaaacttga

tgaaatacagcaaagtgacaaactctttaaaccgggaatgacggttgtcgaccttggtgc

tgctccgggtggttggtcacaatatgtggtcacccaaattggcggcaaaggccgcatcat

cgcttgcgatcttttacctatggatcctatcgttggtgtggactttcttcagggcgattt

tcgtgatgaactggtgatgaaagcactgctggagcgcgttggcgacagcaaagtccaggt

tgtcatgtccgatatggcaccaaacatgagcggaacaccggcggtggatatcccccgtgc

catgtatctggtggaactggcgctagaaatgtgtcgtgatgtattagcgccaggtggcag

ttttgtagtgaaggtgttccagggcgaaggtttcgatgagtatctaagggaaattcgctc

cctgtttacgaaggtcaaagttcgtaagccggactcttctcgtgcacgttcgcgggaagt

gtatattgtagcgaccgggcgtaaaccctaaatgaagcagcaggcaggcattggcattct

tttggcgctcaccacagcaatttgctggggggcgttgccaatcgcaatgaagcaggtgct

ggaggtgatggaacctccgacaatcgtgttttaccgtttcttgatggcgagtattggcct

gggggccattcttgcggtgaagaagaggttgccgccattacgcgtgtttcgtaagccacg

ctggttgattttgttggcagtggcgaccgccgggctgtttgggaacttcatcctgttcag

ctcatccttgcaatacctgagtccgaccgcttcgcaggtgattgggcaactctcgccagt

tggcatgatggttgccagcgtatttatcctgaaagagaaaatgcgcagcactcaggttgt

aggggcattgatgctcctgagcggcctggtgatgttttttaacaccagtctggtcgagat

atttacaaagctaaccgattacacctggggagttatctttggggtcggtgctgcgacggt

ttgggtgagttatggcgtggcgcaaaaggttttattgcgtcggctggcctcaccgcagat

cctgtttttactgtatactttatgtacaattgcgctcttccctctggcaaagcctggagt

gatagcgcagcttagccactggcagctcgcatgtttaattttttgcggactgaatacctt

ggtaggatatggcgccctggcggaagcgatggctcgctggcaggcagcgcaggtgagcgc

gatcatcacgctcaccccactgtttacgctgtttttttcagatcttttatcactggcctg

gcccgatttcttcgccagaccgatgttaaaccttttaggttatctcggtgcgtttgtcgt

ggttgcgggcgcgatgtattccgccattggtcatcgtatttggggcggattacgtaagca

tacaccggtggtatcgcaaccccgcgcaggcgaatgaatgaatttagaaaaaatcaatga

gttaaccgcgcaagatatggcgggtgttaatgcggcaatccttgagcagcttaattccga

cgtccaactgatcaatcagttaggctattacatcgtcagcggcggcggtaaacgtattcg

tccgatgattgctgtactggctgcacgagctgttggctatgagggaaatgcgcatgtcac

cattgctgccctgatcgagtttatccacacggcgactctgctacacgacgacgttgtgga

tgaatcagatatgcgcaggggtaaagctaccgccaacgccgcatttggcaatgccgccag

cgtgctggtaggcgattttatttatacccgcgctttccagatgatgaccagcctcggttc

gctcaaagtgctggaagtcatgtcagaagccgtaaacgtcatcgcagaaggtgaagttct

gcaactgatgaatgttaacgatccggacatcactgaagaaaactacatgcgcgttatcta

tagcaaaaccgcgcgtctgtttgaggctgccgcgcagtgttccgggattctggctggctg

tacgccggaggaggagaaaggcctgcaggattatgggcgctatctcggcactgctttcca

gttgatcgacgatttactcgattacaatgccgatggcgaacagttaggtaaaaacgtcgg

cgacgatctgaacgaaggtaaaccgacgctgccgctgctgcatgcgatgcatcatggcac

accagaacaggcacagatgatccgtaccgccatcgaacagggtaacggtcgccatcttct

ggaaccggttctggaagcaatgaacgcttgtggatctcttgaatggacgcgtcagcgcgc

cgaggaagaagcagacaaagccatcgcagcgttacaggtgctcccggacaccccttggcg

agaagcactcatcggcctcgcgcacatcgctgttcaacgcgatcgttaaatggaaagtaa

tttcattgactggcatcccgctgacatcattgcgggtttgcgcaagaagggaacgtcaat

ggcggcggaatctcgcagaaatggtttgagttcctcaacgctggcgaatgcattatcgcg

cccatggccgaaaggagaaatgattattgcgaaagccctgggaactgacccctgggttat

ctggccatcacgctaccatgatccgcagacccatgagtttatcgacagaacgcagttgat

gcgtagctacactaaaccgaaaaaatgaatggataaatttcgtgttcaggggccaacgaa

gctccagggcgaagtcacaatttccggcgctaaaaatgctgctctgcctatcctttttgc

cgctctactggcggaagaaccggtagagatccagaacgtcccgaaactgaaagacgtcga

tacatcaatgaagctgctaagccagctgggtgcgaaagtagaacgtaatggttctgtgca

tattgatgcccgcgacgttaatgtattctgcgcaccttacgatctggttaaaaccatgcg

tgcttctatctgggcactggggccgctggtagcgcgctttggtcaggggcaagtttcact

acctggcggttgtacgatcggtgcgcgtccggttgatctacacatttctggcctcgaaca

attaggcgcgaccatcaaactggaagaaggttacgttaaagcttccgtcgatggtcgtct

gaaaggcgcacatatcgtgatggataaagtcagcgttggcgcaacggtgaccatcatgtg

tgctgcaactctggcggaaggcaccacgattattgaaaacgcagcgcgtgaaccggaaat

cgtcgataccgcgaacttcctgattacgctgggtgcgaaaattagcggtcagggcaccga

tcgtatcgtcatcgaaggtgtggaacgtttaggcggcggtgtctatcgcgtgctaccgga

tcgtatcgaaaccggtactttcctggtggcggcagcgatttctcgcggcaaaattatctg

ccgtaacgcgcagccagatactctcgacgccgtgctggcgaaactgcgtgacgctggagc

ggacatcgaagtcggcgaagactggattagcctggatatgcatggcaaacgtccgaaggc

tgttaacgtacgtaccgcgccgcatccggcattcccgaccgatatgcaggcccagttcac

gctgttgaacctggtggcagaagggaccgggttcatcaccgaaacggtctttgaaaaccg

ttttatgcatgtgccagagctgagccgtatgggcgcacacgccgaaatcgaaagcaatac

cgttatttgtcacggtgttgaaaaactttctggcgcacaggttatggcaaccgatctgcg

tgcatcagcaagcctggtgctggctggctgtattgcggaagggacgacggtggttgatcg

tatttatcacatcgatcgtggctacgaacgcattgaagacaaactgcgcgctttaggtgc

aaatattgagcgtgtgaaaggcgagtaaatgctgttaaatgcgctggcgtcgctcggaca

taaagggattaaaaccctgagaacgttcgggcgggccgggttaatgttattcaatgcgct

ggtcggcaaaccggaatttcgcaaacatgcgccgctgctggtgcgtcagctctataatgt

cggcgtcctgtcgatgctgattattgtagtttctggcgtgttcatcggaatggtgttggg

gctgcaaggttatctggttctgaccacttatagtgcggaaaccagtctgggtatgctggt

ggcgttatcgctactgcgtgaactggggccggtggttgccgcgctgttgtttgccgggcg

tgctggttcggcgttaaccgcagaaatcggcctgatgcgcgctacagagcaactctccag

tatggagatgatggcggtggatccgctgcgtcgggtcatttctccccgtttctgggctgg

ggttatttcattaccactgttgacggttatcttcgtcgccgtggggatctggggcggatc

gctggtcggcgtcagttggaaaggcattgatagcgggttcttctggtcggcaatgcaaaa

tgccgtcgactggcgtatggatctggtcaactgtctgattaagagcgtggtgttcgccat

cacggtgacgtggatttcgttgtttaacggctacgacgccatcccgacgtctgccgggat

tagccgggcaaccactcgcaccgttgtccactcgtctctggctgttctggggctggattt

tgtgctgaccgcattgatgtttgggaattgaatggagcagtctgtggcgaatttagtcga

tatgcgcgatgtcagttttacgcgtggcaatcgctgcatcttcgataatatttccctgac

cgtgccgcgagggaagatcacggcgatcatggggccatcgggcatcggtaaaacgacgct

actccgtctgattggcgggcaaatcgcaccagatcatggtgagatccttttcgatggtga

gaatattccggcgatgtcacgttcgcgcctgtatacagtgcgcaaacggatgagcatgtt

atttcagtccggggcgttgttcactgatatgaacgtatttgacaacgtcgcctatccact

gcgcgaacatacccaacttcccgcgccattgttgcatagtacggtgatgatgaagctgga

ggccgtggggctgcgtggagcggctaaacttatgccttctgaactttccggtgggatggc

gcggcgtgcagcgctggcacgtgcgattgcgctggagccggatctcatcatgtttgatga

accttttgttgggcaagatcccattaccatgggcgtactggtgaagctgatttctgagct

gaacagcgcgctgggcgtgacttgtgtggtggtttctcacgatgtgccggaagtgttaag

tattgcggatcacgcctggatcctggcggacaaaaaaattgtcgctcatggcagtgccca

ggcgttgcaggcgaatcctgatccgcgcgtacgtcagtttctggacgggatagctgacgg

gcctgttccgttccgctatcctgccggcgattatcacgctgatcttttaccagggagtta

aatgagtaaagccagacgttgggttatcattgtgctatcactggcggttctggtgatgat

cggcattaatatggccgaaaaagacgataccgcccaggtggtcgtcaacaacaatgatcc

cacctataaaagcgagcatacggacacgctcgtctataacccagaaggggcactaagcta

tcgattgattgctcaacacgttgaatattattccgatcaggccgtttcgtggtttacgca

gccggtacttaccacgtttgataaggataaaatcccgacatggtccgtaaaagcagataa

agccaagctgaccaatgaccggatgctctatttatatggacacgttgaagtcaacgcact

cgtgccggactctcaacttcgcagaatcacgacggataacgcgcagatcaatctggtgac

gcaggatgttacctctgaagacctcgtcacgttatacggaacaacatttaactccagcgg

tctgaaaatgcgcggcaacttacgcagcaagaacgccgagctgattgaaaaggttagaac

atcctatgaaattcaaaacaaacaaactcagccttaaatggcaacattaactgcaaagaa

ccttgcaaaagcctataaaggccgtcgcgtggtagaagacgtcagcctgaccgtcaactc

cggggaaattgtcggtctgctggggccaaacggtgccggtaagaccaccactttctacat

ggttgttggcattgtgccgcgcgatgcgggcaacatcattattgatgatgacgatatcag

tctgctgcctctgcatgcacgcgcgcgccgcggtatcggctatctgccacaggaagcctc

cattttccgtcgcctcagcgtttacgataacctgatggcggtactgcaaattcgtgacga

cttgtctgctgaacaacgtgaagaccgtgcgaacgagctgatggaagagtttcacattga

gcacctgcgtgacagcatggggcagtcactctccgggggtgaacgtcgccgcgtagaaat

tgcccgcgcactggctgcgaatccgaaatttattctgctcgacgaaccgtttgccggggt

tgacccgatctcggttatcgacattaaacgcatcattgagcacctgcgcgacagcggcct

gggcgtgctgatcactgaccacaacgtgcgtgaaacactggcggtttgtgaacgcgctta

tatcgtcagtcaggggcatttgatcgcccacggcacgcctacagaaatcttacaagacga

acacgttaagcgtgtataccttggggaagacttcagactctgaatggtactgatgatcgt

cagcggacgttcaggttcaggtaaatctgtcgccctgcgtgcgctggaagatatgggttt

ttactgcgtggataaccttcccgtagtgttgttacccgatctggctcgaaccctggccga

tcgcgagatttctgccgccgtcagcattgatgttcgtaatatgccggagtcaccagaaat

attcgaacaggcgatgagtaacctgcctgacgctttctcaccgcaactcctgttcctgga

tgccgaccgtaataccttaattcgtcgttacagtgacacgcgccgactgcatccgctttc

cagcaaaaacctgtcgctggaaagtgctatcgacaaagaaagcgatctgctggagcctct

gcgttcgcgagcggatctgattgtcgatacctcagaaatgtccgttcacgagctggcaga

aatgctgcgtacccgtctactgggtaaacgtgaacgcgaactgaccatggtctttgagtc

tttcggcttcaaacacggtatccctatcgatgcagattacgtctttgacgtgcgcttctt

gccgaacccgcactgggatccgaaactgcgtccaatgacaggtcttgataagcctgtcgc

cgcgttcctcgaccgccacacagaagtacacaattttatctaccagacgcgaagctatct

tgagctatggttacctatgctggaaaccaacaaccgtagctacctgacggtcgccattgg

ttgtaccggcgggaagcaccgttcggtgtatattgcagagcaactggctgactacttccg

ctcgcgcggtaaaaacgtccagtcacgccatcgtacgctggaaaaacgtaaaccatgaat

gaccgtcaagcaaactgttgaaatcacaaacaagctgggcatgcatgcccggcctgcaat

gaagctgtttgaattaatgcagggttttgacgctgaagtgctgttacgtaatgacgaagg

taccgaggctgaagccaacagcgttattgcgctgctgatgttggattctgccaaaggacg

gcagattgaagttgaagcgaccggtccacaggaagaggaagcactggccgccgttatcgc

cctctttaactctggttttgatgaagattaaatggctgtcgctgccaacaaacgttcggt

aatgacgctgttttccggtcctactgacatctatagccatcaggtccgcattgtgctggc

tgagaaaggtgtaagtttcgagatcgaacacgtggaaaaggacaatccgcctcaggatct

gattgacctcaacccgaatcagagcgttccgaccctggtggatcgtgagctgaccctgtg

ggaatctcgcatcattatggaatatctggatgagcgtttcccgcatccgccactgatgcc

tgtttacccggtagctcgcggtgaaagccgtctgtacatgcatcgcatcgaaaaagactg

gtacacgctgatgaacaccatcatcaacggttcagcttctgaagcagatgccgcacgtaa

gcaactgcgcgaagaactgctggcgattgcgccggtcttcggtcagaagccgtacttcct

gagcgatgagttcagcctggtcgattgctaccttgctccgctgctgtggcgtctgccgca

actgggcatcgagttcagcggcccgggtgcgaaagagctgaaaggctatatgacccgcgt

ctttgagcgtgactctttccttgcttctttaactgaagcagaacgtgaaatgcgtctggg

ccggagttaaatgcgaagctcggctaagcaagaagaactagttaaagcatttaaagcatt

acttaaagaagagaaatttagctcccagggcgaaatcgtcgccgcgttgcaggagcaagg

ctttgacaatattaatcagtctaaagtctcgcggatgttgaccaagtttggtgctgtacg

tacacgcaatgccaaaatggagatggtttactgcctgccagctgaactgggtgtaccaac

cacctccagtccattgaagaatctggtgctggatatcgactacaacgatgcagttgtcgt

gattcataccagccctggcgcggcgcagttaattgctcgcctgctggactcactgggcaa

agcagaaggtattctgggcaccatcgctggcgatgacaccatctttaccacccctgctaa

cggtttcaccgtcaaagacctgtacgaagcgattttagagctgttcgaccaggagcttta

aatgaatatttatacctttgattttgatgagattgagagtcaggaggatttttatcgcga

cttcagccaagcctttggtctggcgaaagataaggtacgcgatctcgactcactatggga

tgtgttaatgaacgatgtcctgccgctaccacttgagattgaatttgttcatctgggaga

gaaaacgcgtcgccgttttggcgcgttaatattgctgtttgatgaggcagaggaagagct

ggaagggcaattgcgttttaatgttcgtcattagatgacttctctgtatttagcttccgg

ttctccgcgtcgtcaggagttacttgcgcaacttggcgtgacctttgaacgtattgttac

gggcattgaggagcagcgtcagccgcaggagagcgcgcagcagtatgttgtgcgtctggc

gcgcgagaaagcacgggcaggtgtcgcgcaaacggcgaaggatctcccggtgctgggtgc

ggatactatcgttatcctgaacggagaagtgctggagaaaccgcgtgacgcagagcatgc

ggcgcagatgttgcgcaaattatcgagtcagacccatcaggtgatgacggcagtggcgtt

ggccgacagccagcacattctcgattgcctggtgatcaccgatgtgactttcagaacgtt

aacagacgaagacatcgcgggctatgtcgccagcggtgaaccgttagataaagcaggtgc

atacggtattcaggggctgggtggctgttttgtcaggaagataaatggcagctatcacgc

cgtagtcggtttaccgctggttgaaacgtatgaattattaagtaattttaacgcactgcg

tgagaaaagggataaacatgacggctgagtggcgagctatcgtagccagggacgctgggt

aatctggctctctttcctcattgcgctgttgctgcaaatcatgccctggccggataacct

gattgttttccggccaaactgggtgttactcatcttgttgtattggatcctggccttgcc

ccatcgcgtaaatgtgggcacaggttttgtgatgggtgccatactggatctgatcagcgg

ctcgacgcttggcgtacgcgtattggcgatgagcatcattgcttacctggtggcgctgaa

ataccagcttttccgcaacctcgcattatggcagcaggcgctggtcgtcatgttgctttc

gctggtggtggatattattgttttctgggcagagtttttagtgattaacgtctctttcag

accggaagtgttctggagtagtgtagtcaatggggtgctctggccgtggattttcttgct

gatgcgtaaagtccgtcagcagtttgcagtgcaataaatgcaggcgttacttttagaaca

gcaggacggcaaaactctcgcatcagtacagactctggacgaaagtcgcctgccggaggg

cgatgtcacggtcgatgttcactggtcgagcctgaactataaagatgcgctggcgattac

cggtaagggaaaaatcatccgtaattttccgatgattcctgggatcgattttgccggaac

tgtacgcaccagcgaagatccgcgttttcatgccggtcaggaggtgttactcactggctg

gggcgttggtgaaaaccactggggtgggctggcggagcaggcgcgagtgaaaggtgactg

gctggttgccatgccgcaagggctggacgcgcgtaaagcaatgattatcggtactgccgg

ttttaccgccatgctgtgtgtgatggcgctggaagatgccggtgttcgcccgcaggacgg

ggagattgtcgtgacgggtgccagtggtggcgtcggcagtaccgccgtggcgctgctgca

taagttgggttatcaggtcgttgccgtttccggtcgcgaaagtacgcatgaatatctgaa

aagtttaggtgccagccgtattctccctcgtgatgagtttgccgaatcccgtcctctgga

aaaacaagtctgggctggggcaattgacaccgttggcgacaaagtgctggcaaaagtgct

ggcgcaaatgaattacggcggctgcgtggcggcctgtggtctggcgggtggttttactct

gccaaccacggtcatgccatttattctgcgtaatgtccgtttgcaaggggtggattcagt

aatgacgccaccagaacgccgcgcacaagcctggcagcgactggtcgccgatttaccgga

atcattctatacccaggcggcaaaagagatatctctggcagaggcaccgaagtttgccga

ggccatcattaataaccagatccagggtcgcacgctggtgaaggttaactaaatggatat

tcgtaagattaaaaaactgatcgagctggttgaagaatcaggcatctccgaactggaaat

ttctgaaggcgaagagtcagtacgcattagccgtgcagctcctgccgcaagtttccctgt

gatgcaacaagcttacgctgcaccaatgatgcagcagccagctcaatctaacgcagccgc

tccggcgaccgttccttccatggaagcgccagcagcagcggaaatcagtggtcacatcgt

acgttccccgatggttggtactttctaccgcaccccaagcccggacgcaaaagcgttcat

cgaagtgggtcagaaagtcaacgtgggcgataccctgtgcatcgttgaagccatgaaaat

gatgaaccagatcgaagcggacaaatccggtaccgtgaaagcaattctggtcgaaagtgg

acaaccggtagaatttgacgagccgctggtcgtcatcgagtaaatgccttggatccaact

gaaactgaacaccaccggcgcgaacgcggaagatcttagcgatgcgctgatggaagcggg

tgccgtttctatcacttttcaggatacccacgatacaccagtatttgagccgctgccggg

cgaaacgcgcctgtggggcgacaccgatgtgattggtctgttcgacgctgaaaccgatat

gaacgacgtggtggcgattctggaaaaccatccgctgctcggcgcaggcttcgcgcataa

aatcgaacaactagaagataaagactgggagcgcgaatggatggataatttccacccgat

gcgctttggtgaacgactgtggatctgccctagctggcgtgatgtgccggacgaaaacgc

cgtcaacgtgatgttagatccagggctggcgtttggtacgggtacccatccaaccacctc

tctgtgcctgcaatggctcgacagcctcgatttaaccggtaaaacagtcatcgactttgg

ctgtggttccggcattctggcgatcgcggcgctgaaactgggtgcagcaaaagccattgg

tattgatatcgatccgcaggcgattcaggccagccgcgataacgccgaacgtaatggcgt

ttctgaccgtctggaactctacttaccgaaagatcagccagaagaaatgaaagccgacgt

ggtggtcgctaacatccttgcaggcccattacgtgaactggcaccgttaatcagcgtcct

gccggtttcaggcggtttgctgggcctttccggtattctggcaagccaggcagagagcgt

ttgtgaagcttatgccgacagcttcgcactggacccggtcgtggaaaaagaagagtggtg

ccgtattaccggtcgtaagaattaaatgtcagttttgcaagtgttacatattccggacga

gcggcttcgcaaagttgctaaaccggtagaagaagtgaatgcagaaattcagcgtatcgt

cgatgatatgttcgagacgatgtacgcagaagaaggtattggcctggcggcaacccaggt

tgatatccatcaacgtatcattgttattgatgtttcggaaaaccgtgacgaacggctggt

gttaatcaatccggagcttttagaaaaaagcggcgaaacaggcattgaagaaggttgcct

gtcgatccctgaacaacgtgctttagtgccacgcgcagagaaagttaaaattcgcgccct

ggaccgtgacggtaaaccatttgaattggaagcagacggtctgttagccatctgtattca

gcatgagatggatcacctggtcggcaaactgtttatggattatctgtcaccgctgaaaca

acaacgtattcgtcagaaagttgaaaaactggatcgtctgaaagcccgggcttaaatgaa

aattatcattctgggtgccggccaggttggcggcacactggcggaaaacctggttggcga

gaacaacgatatcactgttgtcgataccaacggtgagcgtctgcgaaccttgcaggataa

atttgacctgcgagtcgtgcaggggcatggctctcatccacgcgtattgcgggaggcagg

tgccgacgacgccgatatgctggttgctgtaaccagttcagatgaaaccaatatggttgc

ctgccaggtagcctactcgcttttcaacacccctaatcgcatcgctcgtatccgctcacc

agactacgtgcgcgatgccgataagctatttcattcagatgctgtaccgattgatcatct

gatcgcaccagagcagttggttatcgataatatttaccgactgattgagtatcccggcgc

attgcaggtggtgaacttcgctgagggtaaagtcagcctggctgtggttaaagcctatta

tggcggcccgctgattggtaatgcactttcgaccatgcgcgaacatatgccacatatcga

tactcgtgtggcagcaattttccgccacgatcgccccattcgtccgcaaggttcgaccat

tgttgaagctggtgatgaagtgttctttattgccgcttcacagcatatccgcgcggtgat

gagtgaattacagcgactggaaaaaccgtataagcggatcatgctggttggtggcggtaa

tatcggtgcagggctggcgcgtcgtctggaaaaagattacagcgttaaactcatcgaacg

taatcagcagcgcgctgccgaactggcggaaaagttacagaatacgatcgtcttttttgg

tgatgcgtcggatcaagaactgctggccgaagaacatatcgatcaagttgatctgtttat

tgccgtcaccaacgatgacgaggccaatatcatgtccgccatgcttgcaaaacgtatggg

tgcgaaaaaggtgatggtattgatccagcgtcgcgcttatgtggatctggttcaggggag

cgtgatcgatattgcgatttcaccacaacaagcaactatttctgcgttgcttagccatgt

gcgaaaagcagatattgttggtgtttcctcattgcgccgcggcgtagcagaagctattga

agccgttgctcacggtgatgaaagcacctcacgcgttgtcggcagagtcattgacgaaat

caagctaccgccaggaacgattattggagcggtggtacgtggaaacgacgtgatgattgc

caatgacaatctgcgcattgagcaaggcgatcacgtaattatgttcctcacagataaaaa

atttattaccgacgtcgaaagactcttccagccaagccctttcttcttgtaaatgcaggg

ttctgtgacagagtttctaaaaccgcgcctggttgatatcgagcaagtgagttcgacgca

cgccaaggtgacccttgagcctttagagcgtggctttggccatactctgggtaacgcact

gcgccgtattctgctctcatcgatgccgggttgcgcggtgaccgaggttgagattgatgg

tgtactacatgagtacagcaccaaagaaggcgttcaggaagatatcctggaaatcctgct

caacctgaaagggctggcggtgagagttcagggcaaagatgaagttattcttaccttgaa

taaatctggcattggccctgtgactgcagccgatatcacccacgacggtgatgtcgaaat

cgtcaagccgcagcacgtgatctgccacctgaccgatgagaacgcgtctattagcatgcg

tatcaaagttcagcgcggtcgtggttatgtgccggcttctacccgaattcattcggaaga

agatgagcgcccaatcggccgtctgctggtcgacgcatgctacagccctgtggagcgtat

tgcctacaatgttgaagcagcgcgtgtagaacagcgtaccgacctggacaagctggtcat

cgaaatggaaaccaacggcacaatcgatcctgaagaggcgattcgtcgtgcggcaaccat

tctggctgaacaactggaagctttcgttgacttacgtgatgtacgtcagcctgaagtgaa

agaagagaaaccagagttcgatccgatcctgctgcgccctgttgacgatctggaattgac

tgtccgctctgctaactgccttaaagcagaagctatccactatatcggtgatctggtaca

gcgtaccgaggttgagctccttaaaacgcctaaccttggtaaaaaatctcttactgagat

taaagacgtgctggcttcccgtggactgtctctgggcatgcgcctggaaaactggccacc

ggcaagcatcgctgacgagtaaatggctaaacaaccgggattagattttcaaagtgccaa

aggtggcttaggcgagctgaaacgcagactgctgtttgttatcggtgcgctgattgtgtt

tcgtattggctcttttattccgatccctggtattgatgccgctgtacttgccaaactgct

tgagcaacagcgaggcaccatcattgagatgtttaacatgttctctggtggtgctctcag

ccgtgcttctatctttgctctggggatcatgccgtatatttcggcgtcgatcattatcca

gctgctgacggtggttcacccaacgttggcagaaattaagaaagaaggggagtctggtcg

tcgtaagatcagccagtacacccgctacggtactctggtgctggcaatattccagtcgat

cggtattgctaccggtctgccgaatatgcctggtatgcaaggcctggtgattaacccggg

ctttgcattctacttcaccgctgttgtaagtctggtcacaggaacgatgttcctgatgtg

gttgggcgaacagatcactgaacgaggtatcggcaacggtatttcaatcattatcttcgc

cggtattgtcgcgggactcccgccagccattgcccatactatcgagcaagcgcgtcaagg

cgacctgcacttcctcgtgttgctgttggttgcagtattagtatttgcagtgacgttctt

tgttgtatttgttgagcgtggtcaacgccgcattgtggtaaactacgcgaaacgtcagca

aggtcgtcgtgtctatgctgcacagagcacacatttaccgctgaaagtgaatatggcggg

ggtaatcccggcaatcttcgcttccagtattattctgttcccggcgaccatcgcgtcatg

gttcgggggcggtactggttggaactggctgacaacaatttcgctgtatttgcagcctgg

gcaaccgctttatgtgttactctatgcgtctgcaatcatcttcttctgtttcttctacac

ggcgttggttttcaacccgcgtgaaacagcagataacctgaagaagtccggtgcatttgt

accaggaattcgtccgggagagcaaacggcgaagtatatcgataaagtaatgacccgcct

gaccctggttggtgcgctgtatattacctttatctgcctgatcccggagttcatgcgtga

tgcaatgaaagtaccgttctacttcggtgggacctcactgcttatcgttgttgtcgtgat

tatggactttatggctcaagtgcaaactctgatgatgtccagtcagtatgagtctgcatt

gaagaaggcgaacctgaaaggctacggccgataaatgcgtttaaatactctgtctccggc

cgaaggctccaaaaaggcgggtaaacgcctgggtcgtggtatcggttctggcctcggtaa

aaccggtggtcgtggtcacaaaggtcagaagtctcgttctggcggtggcgtacgtcgcgg

tttcgagggtggtcagatgcctctgtaccgtcgtctgccgaaattcggcttcacttctcg

taaagcagcgattacagccgaagttcgtctgtctgacctggctaaagtagaaggcggtgt

tgtagacctgaacacgctgaaagcggctaacattatcggtatccagatcgagttcgcgaa

agtgatcctggctggcgaagtaacgactccggtaactgttcgtggcctgcgtgttactaa

aggcgctcgtgctgctatcgaagctgctggcggtaaaatcgaggaataaatggctcacat

cgaaaaacaagctggcgaactgcaggaaaagctgatcgcggtaaaccgcgtatctaaaac

cgttaaaggtggtcgtattttctccttcacagctctgactgtagttggcgatggtaacgg

tcgcgttggttttggttacggtaaagcgcgtgaagttccagcagcgatccagaaagcgat

ggaaaaagcccgtcgcaatatgattaacgtcgcgctgaataacggcactctgcaacaccc

tgttaaaggtgttcacacgggttctcgcgtattcatgcagccggcttccgaaggtaccgg

tatcatcgccggtggtgcaatgcgcgccgttctggaagtcgctggggttcataacgttct

ggctaaagcatatggttccaccaacccgatcaacgtggttcgtgcaactattgatggcct

ggaaaatatgaattctccagaaatggtcgctgccaagcgtggtaaatccgttgaagaaat

tctggggaaataaatgtctcgtgttgctaaagcaccggtcgttgttcctgccggcgttga

cgtaaaaatcaacggtcaggttattacgatcaaaggtaaaaacggcgagctgactcgtac

tctcaacgatgctgttgaagttaaacatgcagataataccctgaccttcggtccgcgtga

tggttacgcagacggttgggcacaggctggtaccgcgcgtgccctgctgaactcaatggt

tatcggtgttaccgaaggcttcactaagaagctgcagctggttggtgtaggttaccgtgc

agcggttaaaggcaatgtgattaacctgtctctgggtttctctcatcctgttgaccatca

gctgcctgcgggtatcactgctgaatgtccgactcagactgaaatcgtgctgaaaggcgc

tgataagcaggtgatcggccaggttgcagcggatctgcgcgcctaccgtcgtcctgagcc

ttataaaggcaagggtgttcgttacgccgacgaagtcgtgcgtaccaaagaggctaagaa

gaagtaaatggcagcgaaaatccgtcgtgatgacgaagttatcgtgttaaccggtaaaga

taaaggtaaacgcggtaaagttaagaatgtcctgtcttccggcaaggtcattgttgaagg

tatcaacctggttaagaaacatcagaagccggttccggccctgaaccaaccgggtggcat

cgttgaaaaagaagccgctattcaggtttccaacgtagcaatcttcaatgcggcaaccgg

caaggctgaccgtgtaggctttagattcgaagacggcaaaaaagtccgtttcttcaagtc

taacagcgaaactatcaagtaaatgattggtttagtcggtaaaaaagtgggtatgacccg

tatcttcacagaagacggcgtttctatcccagtaaccgtaatcgaagttgaagcaaaccg

cgttactcaggttaaagacctggctaacgatggctaccgtgctattcaggtgaccaccgg

tgctaaaaaagctaaccgtgtgaccaagcctgaagctggccacttcgctaaagctggcgt

agaagctggccgtggtctgtgggaattccgcctggctgaaggcgaagagttcactgtagg

tcagagcattagcgttgaactgtttgctgacgttaaaaaagttgacgtaactggcacctc

taaaggtaaaggtttcgcaggtaccgttaagcgctggaacttccgtacccaggacgctac

tcacggtaactccttgtctcaccgcgttccgggttctatcggtcagaaccagactccggg

caaagtgttcaaaggcaagaaaatggcaggtcagatgggtaacgaacgtgtaaccgttca

gagccttgacgtagtacgcgttgacgctgagcgcaacctgctgctggttaaaggtgctgt

cccgggtgcaaccggtagcgacctgatcgttaaaccagctgtgaaggcgtaaatgattgt

tttctcctcgttacaaattcgtcgcggcgtgcgcgtcctgctggataatgccaccgccac

catcaaccccgggcagaaagtcggcctggtgggtaaaaacggctgtggtaaatctaccct

gctggcattgctgaaaaatgaaatcagcgccgacggcggcagctacacctttccgggaag

ctggcaactggcgtgggtgaatcaggaaacgccggcgttaccgcaagcggcgctggaata

tgtcattgacggcgaccgtgaatatcgtcaactggaagcgcagctgcacgacgccaacga

acgtaacgacgggcacgccattgcgaccattcatggcaagctggatgctattgacgcatg

gagtattcgctcccgtgccgccagcctgctgcacggcctcggtttcagcaatgaacaact

ggagcgcccggtaagtgatttctctggtggctggcgtatgcgtcttaaccttgcccaggc

gctgatttgccgttcagacttgctgctgctcgacgaaccgactaaccacctcgatctcga

tgccgttatctggctggaaaaatggctgaagagctatcagggcacgctgatcctgatctc

tcacgaccgcgacttcctcgatccgatcgtcgataaaattattcatatcgaacaacaaag

catgttcgagtacaccggcaactacagttcgtttgaagtacagcgcgccacccgtctggc

gcagcaacaagcgatgtatgaaagccagcaggaacgcgtggcacatctgcaaagttatat

cgaccgtttccgtgccaaagccaccaaagcgaagcaggcccagagccgcattaagatgct

cgagcgtatggagctgattgccccggcgcacgtcgacaatccgttccgctttagcttccg

cgcgccggaaagtctgccaaatccgttactgaagatggaaaaggtcagcgcaggctatgg

cgatcgcattattctcgactcgattaaactgaatctggtccccggttcgcgcattggtct

gttaggccgcaacggcgcgggtaaatcgacattaatcaaactgttagccggtgaacttgc

gccagtcagtggtgaaataggcctggcaaaagggatcaagctcggctacttcgcccagca

tcaacttgaatacctgcgcgccgacgaatcgcctattcaacatctggcacgtttagcgcc

gcaggagctggagcaaaaactgcgtgactacctcggcggctttggtttccagggcgataa

agtaaccgaagaaacgcgccgcttctcaggtggggaaaaagcccgcctggtgctggcatt

aattgtctggcagcggccgaatctgctgctgctcgacgaaccgactaaccaccttgacct

tgatatgcgtcaggcactcaccgaagcattaatcgagtttgaaggcgcgctggttgtcgt

ttcgcacgaccgtcatttgctgcgttccaccactgacgatctctacctggttcacgatcg

taaagtcgaaccgttcgacggcgatctggaagattatcaacagtggttgagcgacgtaca

aaagcaggaaaaccagaccgacgaagcgccaaaagagaacgcgaacagcgcccaggcacg

taaagatcagaagcgtcgggaagcggagctgcgtgcgcaaacccagccactgcgtaaaga

gattgcccgtctggaaaaagagatggagaagctgaacgcgcaactggcgcaggcggaaga

gaaactcggcgatagcgaactgtatgaccagagccgtaaagcggagttgaccgcctgcct

gcaacagcaagccagcgccaaatccggcctggaagagtgcgaaatggcatggctggaagc

ccaggagcagcttgagcagatgttgctggaaggccaaagcaactgaatggtgcttggcaa

accgcaaacagacccgactctcgaatggttcttgtctcattgccacattcataagtaccc

atccaagagcacgcttattcaccagggtgaaaaagcggaaacgctgtactacatcgttaa

aggctctgtggcagtgctgatcaaagacgaagagggtaaagaaatgatcctctcctatct

gaatcagggtgattttattggcgaactgggcctgtttgaagagggccaggaacgtagcgc

atgggtacgtgcgaaaaccgcctgtgaagtggctgaaatttcgtacaaaaaatttcgcca

attgattcaggtaaacccggacattctgatgcgtctgtctgcacagatggcgcgtcgtct

gcaagtcacttcagagaaagtgggcaacctggcgttcctcgacgtgacgggccgcattgc

acagactctgctcaatctggcaaaacaaccagacgctatgactcacccggacggtatgca

aatcaaaattacccgtcaggaaatcggtcagattgtcggctgttctcgtgaaaccgtggg

acgcattctgaagatgcttgaagatcagaacctgatctccgcacacggtaaaaccatcgt

cgtttacggcactcgttaaatgaaacagtatttgattgccccctcaattctgtcggctga

ttttgcccgcctgggtgaagataccgcaaaagccctggcagctggcgctgatgtcgtgca

ttttgacgtcatggataaccactatgttcccaatctgacgattgggccaatggtgctgaa

atccttgcgtaactatggcattaccgcccctatcgacgtacatctgatggtgaaacccgt

cgatcgcattgtgcctgatttcgctgccgctggtgccagcatcattacctttcatccaga

agcctccgagcatgttgaccgcacgctgcaactgattaaagaaaatggctgtaaagcggg

tctggtatttaacccggcgacacctctgagctatctggattacgtgatggataagctgga

tgtgatcctgctgatgtccgtcaatcctggtttcggcggtcagtctttcattcctcaaac

actggataaactgcgcgaagtacgtcgtcgtatcgacgagtctggctttgacattcgtct

ggaagtggacggaggcgtgaaggtgaacaacattggcgaaatcgctgcggcgggcgcgga

tatgttcgttgccggttcggcaatcttcgaccagccagactacaaaaaagtcattgatga

aatgcgcagtgaactggcaaaggtaagtcatgagtaaatgaagaaaaatcgcgctttttt

gaagtgggcagggggcaagtatcccctgcttgatgatattaaacggcatttgcccaaggg

cgaatgtctggttgagccttttgtaggtgccgggtcggtgtttctcaacaccgacttttc

tcgttatatccttgccgatatcaatagcgacctgatcagtctctataacattgtgaagat

gcgtactgatgagtacgtacaggccgcacgcgagctgtttgttcccgaaacaaattgcgc

cgaggtttactatcagttccgcgaagagttcaacaaaagccaggatccgttccgtcgggc

ggtactgtttttatatttgaaccgctacggttacaacggcctgtgtcgttacaatctgcg

cggtgagtttaacgtgccgttcggccgctacaaaaaaccctattttccggaagcagagtt

gtatcacttcgctgaaaaagcgcagaatgcctttttctattgtgagtcttacgccgatag

catggcgcgcgcagatgatgcatctgtcgtctattgcgatccgccttatgcaccgctgtc

tgcgaccgccaactttacggcgtatcacacaaacagttttacgcttgaacaacaagcgca

tctggcggagatcgccgaaggtctggttgatcgccatattcctgtgctgatctccaatca

cgatacgatgttaacgcgtgagtggtatcagcgcgcaaaattgcatgtcgtcaaagttcg

acgcagtataagcagcaacggcggcacacgtaaaaaggtggacgaactgctggctttgta

caaaccaggagtcgtttcacccgcgaaaaaataaatggagaggattgtcgttactctcgg

ggaacgtagttacccaattaccatcgcatctggtttgtttaatgaaccagcttcattctt

accgctgaaatcgggcgagcaggtcatgttggtcaccaacgaaaccctggctcctctgta

tctcgataaggtccgcggcgtacttgaacaggcgggtgttaacgtcgatagcgttatcct

ccctgacggcgagcagtataaaagcctggctgtactcgataccgtctttacggcgttgtt

acaaaagccgcatggtcgcgatactacgctggtggcgcttggcggcggcgtagtgggcga

tctgaccggcttcgcggcggcgagttatcagcgcggtgttcgtttcattcaagtcccgac

gacgttactgtcgcaggtcgattcctccgttggcggcaaaactgcggtcaaccatcccct

cggtaaaaacatgattggcgcgttctaccagcctgcttcagtggtggtggatctcgactg

tctgaaaacgcttcccccgcgtgagttagcgtcggggctggcagaagtcatcaaatacgg

cattattcttgacggtgcgttttttaactggctggaagagaatctggatgcgttgttgcg

tctggacggtccggcaatggcgtactgtattcgccgttgttgtgaactgaaggcagaagt

tgtcgccgccgacgagcgcgaaaccgggttacgtgctttactgaatctgggacacacctt

tggtcatgccattgaagctgaaatggggtatggcaattggttacatggtgacgcggtcgc

tgcgggtatggtgatggcggcgcggacgtcggaacgtctcgggcagtttagttctgccga

aacgcagcgtattataaccctgctcacgcgggctgggttaccggtcaatgggccgcgcga

aatgtccgcgcaggcgtatttaccgcatatgctgcgtgacaagaaagtccttgcgggaga

gatgcgcttaattcttccgttggcaattggtaagagtgaagttcgcagcggcgtttcgca

cgagcttgttcttaacgccattgccgattgtcaatcagcgtaaatgagcaaatcattaca

aaaacccaccattctgaatgttgaaactgtagcccgttcccgactgtttaccgtcgagag

cgtggatctggagttcagcaatggcgtgcggcgtgtttatgaacgaatgcgtccaaccaa

ccgggaagcagtgatgattgtgccgattgtggacgatcacctgatcctgatccgcgaata

tgcagtgggaactgaatcctacgaattaggtttttcgaaaggattaattgatccgggtga

aagcgtctacgaagccgctaaccgcgagctaaaagaagaggttggatttggtgcgaacga

tctgacttttttgaagaagctcagcatggcaccgtcttacttttccagcaaaatgaatat

cgtggtagcgcaagatctctacccggaatcgctggaaggcgatgagccagagccgctacc

acaggtgcgctggccgctggcgcatatgatggatttgctggaagaccctgacttcaacga

agcgcgtaatgtcagcgcgctgttcctcgtgcgcgaatggttgaaagggcaggggcgagt

gtaaatgaaagaaaaacctgctgttgaggttcgactggataaatggctatgggctgcccg

tttttataaaacccgcgcgctggcccgtgaaatgattgaaggcggtaaggtgcattacaa

cgggcagcgcagcaagccgagcaaaatcgtcgagctgaatgccacgctcactctgcgcca

gggaaatgacgaacgcacggtgattgtaaaggcgattactgaacagcgtcgccccgccag

cgaggcagccttgctgtatgaagagactgcggaaagtgtagagaaacgcgaaaaaatggc

gctggcacgtaaacttaatgccttaaccatgccgcacccggaccgacgcccggacaaaaa

agagcgccgcgacctgttacgatttacacacggcgacagtgaataaatgcaagagaacta

caagattctggtggtcgatgacgacatgcgcctgcgtgcgctgctggagcgttatctcac

cgaacaaggcttccaggttcgaagcgtcgctaatgcagaacagatggatcgcctgctgac

tcgtgaatctttccatcttatggtactggatttgatgttacctggtgaagatggcttgtc

gatttgccgacgtcttcgtagtcagagcaacccgatgccgatcattatggtgacggcgaa

aggggaagaagtggaccgtatcgttggcctggagattggcgctgacgattacattccaaa

accgtttaacccgcgtgaactgctggcccgtatccgtgcggtgctgcgtcgtcaggcgaa

cgagctaccaggcgcaccgtcacaggaagaggcggtaattgctttcggtaagttcaaact

taacctcggtacgcgcgaaatgttccgcgaagacgagccgatgccgctcaccagcggtga

gtttgcggtactgaaggcactggtcagccatccgcgtgagccgctctcccgcgataagct

gatgaaccttgcccgtggtcgtgaatattccgcaatggaacgctccatcgacgtgcagat

ttcgcgtctgcgccgcatggtagaagaagatccagcgcatccgcgttacattcagaccgt

ttggggcctgggctacgtctttgtaccggacggctctaaagcatgaatgcaggttttaca

tgtatgttcagagatgttcccgctgcttaaaaccggcggtctggctgatgttattggggc

attacccgcagcacaaatcgcagacggcgttgacgctcgcgtactgttgcctgcatttcc

cgatattcgccgtggcgtgaccgatgcgcaggtagtatcccgtcgtgataccttcgccgg

acatatcacgctgttgttcggtcattacaacggggttggcatatacctgattgacgcgcc

gcatctctatgatcgtccgggaagcccgtatcacgatactaacttatttgcctataccga

caacgtattgcgttttgcgctgctggggtgggttggggcagaaatggccagcgggcttga

cccattctggcgtcctgatgtggtgcatgcgcacgactggcatgcaggccttgcgcctgc

atatctggcggcgcgcgggcgtccggcgaagtcggtgtttactgtgcacaacctggccta

tcaaggcatgttttatgcacatcacatgaatgacatccaattgccatggtcattctttaa

tattcatgggctggaattcaacggacaaatctctttcctgaaggccggtctgtactatgc

cgatcacattacggcggtcagtccaacctacgctcgcgagatcaccgaaccgcagtttgc

ctacggtatggaaggtctgttgcaacagcgtcaccgtgaagggcgtctttccggcgtact

gaacggcgtggacgagaaaatctggagtccagagacggacttactgttggcctcgcgtta

cacccgcgatacgttggaagataaagcggaaaataagcgccagttacaaatcgcaatggg

gcttaaggttgacgataaagtgccgctttttgcagtggtgagccgtctgaccagccagaa

aggtctcgacctggtgctggaagccttaccgggccttctggagcagggcgggcagctggc

gctactcggcgcgggcgatccggtgctgcaggaaggtttccttgcggcggcagcggaata

ccccggccaggtgggcgttcagattggctatcacgaagcattttcgcatcgcattatggg

cggcgcggacgtcattctggtgcccagccgttttgaaccgtgcggcttaacgcaacttta

tggattgaagtacggtacgctgccgttagtgcggcgcaccggtgggcttgctgatacggt

ttctgactgttctcttgagaaccttgcagatggcgtcgccagtgggtttgtctttgaaga

tagtaatgcctggtcgctgttacgggctattcgacgtgcttttgtactgtggtcccgtcc

ttcactgtggcggtttgtgcaacgtcaggctatggcaatggattttagctggcaggtcgc

ggcgaagtcgtaccgtgagctttactatcgcttgaaatagatgacacaactcgccattgg

caagcccgctcccctcggcgcgcattacgacggtcagggcgtcaacttcacacttttctc

cgctcatgccgagcgggtagagctgtgtgtctttgacgccaatggccaggaacatcgcta

tgacttgccagggcacagtggcgacatttggcacggttatctgccggatgcgcgcccggg

tttgcgttatggttatcgcgttcatggcccctggcaacccgccgaggggcatcgctttaa

cccggcgaagttgttgattgatccttgcgcgcggcaaattgacggggaatttaaagataa

cccgctgctgcacgccggtcataatgaacctgactatcgcgacaacgccgccattgcgcc

gaaatgcgtagtggtggttgatcactatgactgggaagatgatgccccgccgcgcacgcc

gtggggcagcaccatcatttatgaagcccatgttaaaggattaacgtacctgcacccgga

gatcccggtcgagatccgtggcacttataaagccctcgggcatccggtgatgatcaacta

tttgaaacaattgggcattaccgcgctggaactgctgccagtggcgcagtttgccagtga

accacgtctgcaacgcatggggctaagtaactactggggttacaacccggtggcgatgtt

tgcgctgcatccggcgtatgcctgctcgccagaaacggcgctggatgagtttcgcgatgc

aatcaaagcactgcataaagcgggtatcgaagtcattcttgatatcgtactcaaccatag

tgcggaactggacctcgacggcccgttattctcgctgcgtgggatcgacaaccgtagcta

ttattggataagagaagacggcgattatcacaactggaccggttgtggcaacacgctcaa

tttgagtcacccggcggtggtggattatgccagcgcttgcctgcgttattgggtagaaac

ctgccacgtcgatggtttccgctttgatctggcggcagtcatgggccgtacgccagagtt

ccgtcaggatgcgccgttgtttaccgcaatccagaactgcccggtgctctcgcaggtgaa

gttaattgctgaaccgtgggatatcgctcctggtggttatcaggtgggaaatttcccgcc

gctgtttgccgagtggaacgatcatttccgcgatgctgcccgtcgtttctggctgcatta

tgatttgcctctgggggcgtttgccgggcgttttgctgcctccagcgatgtttttaaacg

taatggtcgtctgccgagtgccgcgattaatctcgtcaccgcacatgacggttttacgct

tcgcgactgcgtttgcttcaaccataaacacaatgaagcaaacggagaagaaaatcgcga

cgggaccaacaacaattacagtaacaatcatggtaaagaagggttaggcggtactcttga

tctggttgaacggcggcgcgacagcattcatgccctgttaacaacgttgttgctctccca

gggcacgccgatgttactggccggtgacgaacatggtcacagccagcatggcaataacaa

tgcctactgtcaggataaccaattaacctggttggactggtcgcaggcaagcagtggttt

aaccgcatttaccgccgcgttaatccatctgcgcaagcgtattcccgctttggtggagaa

tcgctggtgggaagaaggcgacggcaatgtccgttggctaaatcgatatgctcaaccttt

aagcacggatgagtggcaaaacgggccgaaacagctgcaaattctgctctcggatcgctt

tttgatcgcaattaacgccacgcttgaggtaacagagattgttttacctgctggggagtg

gcacgccattcccccattcgctggagaggataacccagtgattacggctgtctggcaggg

acctgcacacggattgtgtgtgttccagagatgaatgaaacgacttctgattcttacggc

actcctgccgtttgtcggctttgcacagcccattaatactctgaacaaccctaaccagcc

ggggtatcagatccccagtcagcagcggatgcaaacccagatgcagactcagcaaatcca

gcaaaaagggatgctgaatcagcaactgaaaacgcaaactcagttgcaacaacagcattt

agaaaaccagataaacaataattctcagcgggtgttgcagtcacagccgggggagcgaaa

tcccgcccggcagcaaatgctgcccaacaccaatggcgggatgttaaacagcaaccgtaa

tccggatagttcgttgaatcagcagcatatgttgccggagaggagaaacggcgacatgct

gaatcagcccggcacgccgcagcctgatataccgttgaaaactattgggccgtaaatgaa

gctgaccatcattcgattagaaaactttagcgaccaggaccggattgacctgcaaaagat

ctggccggagtattccccttcctctttacaggttgacgataaccaccgcatctacgccgc

gcgttttaacgagcgcctgctcgctgccgtacgggtaaccttaagcggcaccgagggagc

actggattccctgcgcgtgcgggaagtcactcgacgtcgcggtgtggggcaatatctgct

ggaagaggttttgcgtaacaatcctggcgtttcatgctggtggatggcggatgcaggcgt

ggaagatcgcggtgtgatgacggcgtttatgcaggcgctggggtttacggcacagcaggg

cggctgggagaagcgttaaatgaaaaaaccgaatcattccggcagcggccaaatccgcat

tattggcgggcagtggcgaggccgtaaactaccggttcctgatagcccaggtctgcgccc

caccaccgaccgcgtacgcgaaacgttgtttaactggctggctccggtcattgttgacgc

ccaatgtctggattgcttcgccgggagcggcgcgctggggctggaagcgttatcgcgcta

tgctgcgggggccacgttgattgagatggatcgcgcggtttctcagcagttaattaagaa

tctggcgacactaaaagcaggcaatgcacgcgtggtgaacagcaacgcgatgtcattcct

ggcgcaaaaaggcacaccgcataatatcgtgtttgtcgatccaccgttccgccgtggctt

gttagaagagacgataaatttactggaagataacggctggctggctgacgaatccctgat

ttatgtcgaaagcgaagtcgaaaacggtctgcccaccgttccggcaaactggtcattgca

tcgggaaaaagtggcgggtcaggtggcttatcggctgtatcaacgcgaagcacaaggaga

aagtgatgctgattaaatgtatcggatagttctggggaaagtttcgaccttaagcgcagc

tccactgccaccgggtttacgcgagcaagcaccgcaaggtccacgacgcgaacgctggct

ggcggggcgtgcattgctttcgcacacgctttccccgctaccggagatcatctatggcga

acaaggcaaacctgcatttgcgccggaaacgccgctatggttcaacttaagccatagcgg

tgacgatatcgctctgctgttgagtgatgaaggcgaagtcggctgcgatatcgaagtgat

tcgcccgcgcgccaactggcgctggctggcgaacgccgtattcagcctcggggaacacgc

tgagatggacgccgtgcatcctgatcagcaactggaaatgttctggcgcatctggacgcg

caaagaagccatcgttaaacagcgtggtggcagcgcctggcaaatcgtcagcgtagacag

cacctatcactcctcgctgtcagtcagccattgtcagctagaaaatttaagcctggcgat

ctgcacccccaccccctttacgctcaccgccgacagtgtgcaatggatcgattcagttaa

ctgaatgataagcaccgtcgcattattttgggctttatgtgtcgtttgcattgttaacat

ggcgcgctatttctcatcactacgcgcgttgttagtggtactgcgtaactgcgatccatt

gctctatcaatatgttgatggagggggcttttttacctcacatggccaacccaacaaaca

ggtgcgtctcgtttggtatatctatgcccaacgttatcgcgatcatcacgatgatgagtt

tattcgccgctgtgagcgggtgcgtcggcagtttattctgactagcgcattgtgtggtct

ggtggtggtcagcctgattgcattgatgatttggcattaaatggcttataaacacattct

catcgcggtcgatctctccccggaaagcaaagttctggtagagaaagcagtctctatggc

tcgcccctacaatgcgaaagtttctctgatccacgtagatgtaaactactctgacctata

caccgggcttattgatgtgaatctgggtgatatgcagaaacgcatctctgaagagacaca

tcatgcgctgaccgagctttccactaatgcaggctacccaatcactgaaaccctgagcgg

cagcggcgacctgggccaggttctggtcgatgcaatcaagaaatacgatatggatctggt

ggtttgtggtcaccaccaggacttctggagcaaactgatgtcttccgcacgtcagctgat

caacaccgttcacgttgatatgctgattgttccgctgcgcgacgaagaagaataaatgtt

tcttataattaccagggatacgatgttcttcaccgcgatgaaaaacattctgagtaaagg

taatgtcgttcatatacagaacgaagaagagatcgacgtaatgttgcatcagaatgcctt

cgtcattattgatacattaatgaataatgtatttcattctaattttctcactcaaattga

acgattaaaacctgtccatgtcattattttctccccctttaatattaaacgctgcctggg

gaaagtgccggtgacctttgttccgcggactatcactatcattgattttgtcgcactcat

caatggcagttactgctctgtgcctgaagcgaatgtgtcactttcgcgcaagcaacatca

ggtattgagctgcattgcgaatcaaatgacaacggaagatattctggagaaactgaaaat

atcgctaaaaacgttctactgccataaacacaatatcatgatgatcctcaatcttaagcg

gatcaatgagctggtacgccatcagcatattgattatctggtgtgaatgaaaaaagtatt

aggcgttattcttggtggtctgcttcttctgccagttgtgagcaatgcagcggatgcgca

aaaagcagctgataacaaaaaaccggtcaactcctggacctgtgaagatttcctggctgt

ggacgaatccttccagccaactgcagttggttttgctgaagcgctgaacaacaaagataa

accagaagatgcggttttagatgttcagggtattgcaaccgtaaccccagctatcgttca

ggcttgtactcaggataaacaagccaactttaaagataaagttaaaggcgaatgggacaa

aattaagaaagatatgtaaatgcaatcactacatgggaattgtctaattgcgtacgcaag

acataaatatattctcaccatggttaatggtgaatatcgctattttaatggcggtgactt

ggtttttgcggatgcaagccaaattcgagtagataagtgtgttgaaaattttgtatttgt

gtcaagggacacactttcattatttctcccgatgctcaaggaggaggcattaaatcttca

tgcacataaaaaagtttcttcattactcgttcatcactgtagcagagatattcctgtttt

tcaggaagttgcgcaactatcgcagaataagaatcttcgctatgcagaaatgctacgtaa

aagagcattaatctttgcgttgttatctgtttttcttgaggatgagcactttataccgct

gcttctgaacgttttacaaccgaacatgcgaacacgagtttgtacggttatcaataataa

tatcgcccatgagtggacactagcccgaatcgccagcgagctgttgatgagtccaagtct

gttaaagaaaaaattgcgcgaagaagagacatcatattcacagttgcttactgagtgtag

aatgcaacgtgctttgcaacttattgttatacatggtttttcaattaagcgagttgcagt

atcctgtggatatcacagcgtgtcgtatttcatttacgtctttcgaaattattatgggat

gacgcccacagagtatcaggagcgatcggcgcagagattgtcgaaccgtgactcggcggc

aagtattgttgcgcaagggaatttttacggcactgactgttctgcggaaggaataagatt

atagatgaaaacctctctgtttaaaagcctttactttcaggtcctgacagcgatagccat

tggtattctccttggccatttctatcctgaaataggcgagcaaatgaaaccgcttggcga

cggcttcgttaagctcattaagatgatcatcgctcctgtcatcttttgtaccgtcgtaac

gggcattgcgggcatggaaagcatgaaggcggtcggtcgtaccggcgcagtcgcactgct

ttactttgaaattgtcagtaccatcgcgctgattattggtcttatcatcgttaacgtcgt

gcagcctggtgccggaatgaacgtcgatccggcaacgcttgatgcgaaagcggtagcggt

ttacgccgatcaggcgaaagaccagggcattgtcgccttcattatggatgtcatcccggc

gagcgtcattggcgcatttgccagcggtaacattctgcaggtgctgctgtttgccgtact

gtttggttttgcgctccaccgtctgggcagcaaaggccaactgatttttaacgtcatcga

aagtttctcgcaggtcatcttcggcatcatcaatatgatcatgcgtctggcacctattgg

tgcgttcggggcaatggcgtttaccatcggtaaatacggcgtcggcacactggtgcaact

ggggcagctgattatctgtttctacattacctgtatcctgtttgtggtgctggtattggg

ttcaatcgctaaagcgactggtttcagtatcttcaaatttatccgctacatccgtgaaga

actgctgattgtactggggacttcatcttccgagtcggcgctgccgcgtatgctcgacaa

gatggagaaactcggctgccgtaaatcggtggtggggctggtcatcccgacaggctactc

gtttaaccttgatggcacatcgatatacctgacaatggcggcggtgtttatcgcccaggc

cactaacagccagatggatatcgtccaccaaatcacgctgttaatcgtgttgctgctttc

ttctaaaggggcggcaggggtaacgggtagtggctttatcgtgctggcggcgacgctctc

tgcggtgggccatttgccggtagcgggtctggcgctgatcctcggtatcgaccgctttat

gtcagaagctcgtgcgctgactaacctggtcggtaacggcgtagcgaccattgtcgttgc

taagtgggtgaaagaactggaccacaaaaaactggacgatgtgctgaataatcgtgcgcc

ggatggcaaaacgcacgaattatcctcttaaatggcacgcgtaactgttcaggacgctgt

agagaaaattggtaaccgttttgacctggtactggtcgccgcgcgtcgcgctcgtcagat

gcaggtaggcggaaaggatccgctggtaccggaagaaaacgataaaaccactgtaatcgc

gctgcgcgaaatcgaagaaggtctgatcaacaaccagatcctcgacgttcgcgaacgcca

ggaacagcaagagcaggaagccgctgaattacaagccgttaccgctattgctgaaggtcg

tcgttaaatgatcaccgttgcccttatagacgatcacctcatcgtccgctccggctttgc

gcagttgctggggctggaacctgatttgcaagtagttgccgagtttggttcggggcgcga

ggcgctggcggggctgccggggcgcggtgtgcaggtgtgtatttgcgatatctccatgcc

cgatatctccggtctggagctgctaagccagctgccgaaaggtatggcgacaattatgct

ctccgttcatgacagtccggcgctggttgagcaggcgcttaacgcgggggcgcgtggctt

tctctccaagcgttgtagccctgacgaactgattgctgcggtgcatacggttgccacagg

cggctgttatctgacgccggatattgccattaaactggcatccggtcgccaggacccact

aaccaaacgtgaacggcaggtggcggaaaaactggcgcaaggaatggcggtgaaagagat

tgccgccgaactgggcttgtcaccgaaaacggtacacgtccatcgcgccaatctgatgga

aaaactgggcgtcagtaacgacgttgaactggcgcgccgtatgtttgatggctggtgaat

gccggatagccgcaaagccagacgcattgccgacccagggctgcagccggagcgtacatc

actggcgtggtttcgtaccatgctgggctacggcgcgttgatggcgttggctatcaaaca

caactggcaccaggcgggcatgttattctggatttcgattggcatcctcgccatcgtggc

gctgatcctctggcactacactcgtaatcgcaatttaatggatgtcacgaatagcgattt

ttcccaatttcacgtagtccgtgacaaatttttgatctccctcgcggtgttatctctcgc

aatactgtttgctgtaacgcatatacatcaacttatcgtatttattgagagagtcgcatg

aatgttgaataatgctatgagcgtagtgatccttgccgcaggtaaaggcacgcgcatgta

ttccgatcttccgaaagtgctgcatacccttgccgggaaagcgatggttcagcatgtcat

tgatgctgcgaatgaattaggcgcagcgcacgttcacctggtgtacggtcacggcggcga

tctgctaaaacaggcgctgaaagacgacaacctgaactgggtgcttcaggcagaacagct

gggtactggtcatgcaatgcagcaggctgcacctttctttgccgatgatgaagacatttt

aatgctctacggcgacgtgccgctgatctctgtcgaaacactccagcgtctgcgtgatgc

taaaccgcagggtggcattggtctgctgacggtgaaactggatgatccgaccggttatgg

acgtatcacccgtgaaaacggcaaagttaccggcattgttgagcacaaagatgccaccga

cgagcagcgtcagattcaggagatcaacaccggcattctgatcgctaacggcgcagatat

gaaacgctggctggcgaagcttaccaacaataatgctcagggcgaatactacatcaccga

cattattgcgctggcgtatcaggaagggcgtgaaatcgtcgccgttcatccgcaacgttt

aagcgaagtagaaggcgtgaataaccgcctgcaactctcccgtctggagcgcgtttacca

gtccgaacaggctgaaaaactgctgttagcaggcgttatgctgcgcgatccggcgcgttt

tgatctgcgcggtacgcttactcacgggcgcgatgttgaaattgatactaacgttatcat

cgagggcaacgtgactctcggtcatcgcgtgaaaatcggcaccggttgcgtgattaaaaa

cagcgtgattggcgatgattgcgaaattagcccatataccgtcgtggaagacgcgaatct

ggcggcggcctgtaccattggcccgtttgcccgtttgcgtcctggtgctgagttgctcga

aggtgcacacgtcggtaactttgttgagatgaaaaaagcacgtctgggtaaaggctcgaa

agctggtcatctgacttaccttggcgatgcggaaattggcgataacgttaacatcggcgc

gggaaccattacctgcaactacgatggtgcgaataaatttaagaccattatcggcgacga

tgtgtttgtcggttccgacactcagctggtggccccggtaacagtaggcaaaggtgcaac

cattgctgcgggtacaactgtgacgcgtaatgtcggcgaaaatgcattagctatcagccg

tgtgccgcagactcagaaagaaggctggcgtcgtccggtaaagaaaaagtgaatggctac

tggaaagattgtccaggtaatcggcgccgtagttgacgtcgaattccctcaggatgccgt

accgcgcgtgtacgatgctcttgaggtgcaaaatggtaatgagcgtctggtgctggaagt

tcagcagcagctcggcggcggtatcgtgcgtaccatcgcaatgggttcctccgacggtct

gcgtcgcggtctggatgtaaaagacctcgaacacccgatcgaagtcccggtaggtaaagc

gactctgggccgtatcatgaacgtactgggtgaaccggtcgacatgaaaggcgagatcgg

tgaagaagagcgttgggcgattcaccgcgcagcaccttcctacgaagagctgtcaaactc

tcaggaactgctggaaaccggtatcaaagttatcgacctgatgtgtccgttcgctaaggg

cggtaaagttggtctgttcggtggtgcgggtgtaggtaaaaccgtaaacatgatggagct

cattcgtaacatcgcgatcgagcactccggttactctgtgtttgcgggcgtaggtgaacg

tactcgtgagggtaacgacttctaccacgaaatgaccgactccaacgttatcgacaaagt

atccctggtgtatggccagatgaacgagccgccgggaaaccgtctgcgcgttgctctgac

cggtctgaccatggctgagaaattccgtgacgaaggtcgtgacgttctgctgttcgttga

caacatctatcgttacaccctagccggtacggaagtatccgcactgctgggccgtatgcc

ttcagcggtaggttatcagccgaccctggcggaagagatgggcgttctgcaggaacgtat

cacctccaccaaaactggttctatcacctccgtacaggcggtatacgtacctgcggatga

cttgactgacccgtctccggcaaccacctttgcgcaccttgacgcaaccgtggtactgag

ccgtcagatcgcgtctctgggtatctacccggccgttgacccgctggactccaccagccg

tcagctggacccgctggtggttggtcaggaacactacgacaccgcgcgtggcgttcagtc

catcctgcaacgttatcaggaactgaaagacatcatcgccatcctgggtatggatgaact

gtctgaagaagacaaactggtggtagcgcgtgctcgtaagatccagcgcttcctgtccca

gccgttcttcgtggcagaagtattcaccggttctccgggtaaatacgtctccctgaaaga

caccatccgtggctttaaaggcatcatggaaggcgaatacgatcacctgccggagcaggc

gttctacatggtcggttccatcgaagaagctgtggaaaaagccaaaaaactttaaatggc

cggcgcaaaagagatacgtagtaagatcgcaagcgtccagaacacgcagaagatcactaa

agcgatggagatggtcgccgcttccaaaatgcgtaaatcgcaggatcgcatggcggccag

ccgtccttatgcagaaaccatgcgcaaagtgattggtcaccttgcacacggtaatctgga

atacaagcacccttacctggaagaccgcgacgttaaacgcgtgggctacctggtggtgtc

gaccgaccgtggtttgtgcggtggtttgaacattaacctgttcaaaaaactgctggcgga

aatgaagacctggaccgacaaaggcgttcaatgcgacctcgcaatgatcggctcgaaagg

cgtgtcgttcttcaactccgtgggcggcaatgttgttgcccaggtcaccggcatggggga

taacccttccctgtccgaactgatcggtccggtaaaagtgatgttgcaggcctacgacga

aggccgtctggacaagctttacattgtcagcaacaaatttattaacaccatgtctcaggt

tccgaccatcagccagctgctgccgttaccggcatcagatgatgatgatctgaaacataa

atcctgggattacctgtacgaacccgatccgaaggcgttgctggataccctgctgcgtcg

ttatgtcgaatctcaggtttatcagggcgtggttgaaaacctggccagcgagcaggccgc

ccgtatggtggcgatgaaagccgcgaccgacaatggcggcagcctgattaaagagctgca

gttggtatacaacaaagctcgtcaggccagcattactcaggaactcaccgagatcgtctc

gggggccgccgcggtttaaatggcagatatcactcttatcagcggcagcaccctcggcgg

tgccgaatatgtagcagaacacctggctgaaaagctggaagaggcgggttttaccaccga

aacgctgcacggcccgctgttggaagatttatctgcctcggggatctggctggttatcag

ctccacccacggtgccggagatattccggacaacctttctcctttctatgaagcattgca

ggaacagaagcccgatctttctgcggtccgctttggcgcaatcggtattggtagtcgtga

atatgacaccttttgtggggctattgataaactcgaggctgaactcaaaaattccggtgc

aaaacagacgggcgaaacactgaagatcaacattcttgatcacgacattccggaagatcc

ggcagaagaatggctgggatcgtggattaatttactcaaataaatggaaaattatctaat

cgacaatctggaccgtggcatcctggaagcattaatgggcaatgcgcgcaccgcttacgc

cgaactggcgaaacaatttggcgtcagtccggggacgattcacgttcgagtagagaaaat

gaagcaggcggggatcattaccggggcgcgtattgatgtcagcccgaagcagctcggtta

tgacgtaggctgctttatcggcattatattaaagagcgccaaagactatccttccgcgct

ggcaaagctggaaagcctcgatgaagtgaccgaggcgtactacaccaccggccactacag

catctttataaaagtgatgtgccgttcgatcgatgctctccagcatgtacttatcaacaa

gatccaaacaattgatgaaattcagtccaccgagacactgatcgtcctgcagaacccgat

catgcgtaccatcaagccctgaatggcggaaagctttacgacgactaatcgatatttcga

caataaacattatccacgtggattctctcgtcatggtgatttcaccatcaaagaggcaca

actgcttgagcgtcatggttatgccttcaatgagttggatcttggcaaacgcgagccggt

taccgaggaagagaaactcttcgtagcagtatgccgtggcgaacgtgagccagtgacaga

agcagaacgcgtgtggtccaagtatatgacgcgtatcaagcgtccaaaacgttttcacac

cctttccggcggtaaaccgcaggttgaaggtgctgaagactacaccgattctgacgatta

aatggcaaaaacagcagcagcactgcatatccttgtaaaagaagagaaactggctctgga

tcttctcgagcagattaagaacggtgccgatttcggcaagctggcgaagaaacactccat

ttgcccatcaggcaaacgcggcggtgatttaggtgaattccgccagggtcagatggttcc

ggcgttcgataaagttgtgttctcttgtccggtactggagccgactggcccgctgcatac

ccagttcggttaccacatcattaaggtgctttaccgtaactaagtgaatttactgacagt

gagtactgatctcatcagtatttttttattcacgacactgtttctgttttttgcccgtaa

ggtggcaaaaaaagtcggtttagtggataaaccaaacttccgcaaacgtcaccagggatt

gatacctctcgttggggggatttcggtttacgcagggatttgcttcacgttcggaattgt

cgattactatattccgcatgcatctctctatctcgcttgtgccggtgtgcttgttttcat

tggcgcgctggatgaccgttttgatatcagcgtaaaaatccgtgccaccatacaggccgc

tgttggcattgttatgatggtgttcggcaaactttatctcagtagcctgggttatatctt

tggctcctgggagatggtgctcggaccgtttggttacttcctgacgctatttgccgtctg

ggcggccattaatgcgttcaacatggttgatggcattgatggcttgctgggcgggttgtc

ctgcgtctcgtttgcagcaatcggtatgattttgtggttcgacgggcaaaccagcctcgc

aatctggtgctttgcgatgatcgccgccatccagccatacatcatgcttaaccttggtat

cctgggtcgccgctacaaagtctttatgggtgatgcgggcagtacgctgattggttttac

cgtgatctggatcctgctcgaaacgacccagggcaaaacccatcccatcagcccggttac

cgctttgtggataatcgccattccgctaatggatatggtggcgattatgtaccgtcgcct

gcgtaaaggcatgagcccattctctcctgaccgtcagcatattcaccatttgatcatgcg

tgccgggtttacttcccgccaggcgtttgtgctgattacccttgccgcagcactgctcgc

ttccattggcgtgctggcagaatattctcattttgtcccggagtgggtcatgctggtgct

ctttttgctagcattcttcctctatggatattgcattaagcgtgcctggaaagttgctcg

ctttattaagcgcgtaaaacgcagactgcgtagaaatcgtggtggcagccccaatttaac

caaataaatgattccatttaacgcaccgccggtggtgggaaccgaactcgactatatgca

gtcggcaatgggtagcggcaaactgtgtggcgatggcggttttacccgtcgctgccagca

gtggctggagcaacgttttggcagcgccaaagtgttactgacgccgtcctgcaccgcttc

gctggagatggcggcgctgctgctcgatatccagcctggcgatgaagtgatcatgccgag

ctacacctttgtctccaccgccaatgcctttgtgctgcgtggcgcaaaaatcgtttttgt

ggatgttcgcccggacaccatgaacatcgacgaaacgctgattgaagcggcgatcaccga

caaaacgcgcgttatcgtgccggtccattacgcgggtgtggcctgcgaaatggacaccat

tatggcgttggcaaaaaagcataatttgtttgtggtagaagatgccgctcagggcgtgat

gtccacttacaaagggcgtgcactgggaaccattggtcatattggctgctttagcttcca

tgaaaccaaaaactacacggcgggtggtgaaggcggcgcgacgctgattaacgataaagc

gttaatcgaacgagccgagatcatccgtgaaaagggcactaaccgcagccagttcttccg

tggtcaggtcgataaatatacctggcgcgatattggctccagctatttgatgtccgatct

gcaagctgcgtacctgtgggcgcaactggaagcagcggatcgtatcaaccagcaacgtct

ggcgctgtggcaaaactactacgatgcgttagcacctctggcgaaagccgggcgtatcga

gctgccgtcgattcccgatggctgcgtgcagaacgcgcatatgttctacattaaactgcg

ggatattgatgaccggagcgcgttgattaactttctgaaagaagcggaaattatggcggt

gttccattacattccgctgcacggttgccctgcgggggaacgctttggtgagttccacgg

tgaagatcgctacaccaccaaagagagcgagcgcctgctgcgcctgccgctgttctacaa

cctgtcgcccgtcaatcagcgtacggtaattgcgactttgttgaactacttctcctgaat

gagtatcctggtcacccgcccgtctcccgctggagaagagttagtgagccgtctgcgcac

actggggcaggtggcctggcattttccactgatagagttttccccaggtcggcaattacc

acaacttgccgacctgctatcagcacttggtgaaggcgatctgttgtttgccctttcgca

acacgccgttgcttatgcccaatcgcaactgcatcagcaagctcttaagtggccattact

tgctggttatttcgctatcggccgcaccactgcgctggcgctgcataccgtgagtggaca

ccaggttcgttatccgcaggatcgggaaattagtgaagtcttgctacaattacctgaatt

acaaaatattgctggcaaacgtgcgctgatattacgtggcaacggcggtcgtgagctgat

tggagaaactctgacagcacgcggtgctgaagtcgctttttgtgaatgttatcaacggtg

cgccattcattatgacggtgcagaagaggcgatgcgttggcaatctcgcgaggtgactac

cgtcgttgttaccagcggtgaaatgttgcatcaactctggtcattgatcccacaatggta

tcgtgagcactggttactacgctgtcggcttttggtcgtcagtgagcgtttggcgaaact

cgcccgggaactgggctggaatgacattaaggtcgccgataacgctgacaacgatgcgct

attacgggcattacaataaatgaacgacagtgaatttcatcgcctggctgatcaactgtg

gctgaccattgaagagcgtctggacgactgggatggcgacagcgatatcgactgcgaaat

caacggcggcgtgctgaccattacctttgagaatggcagcaaaatcattatcaaccgcca

ggaaccgctgcaccaggtatggctggcaaccaaacaaggcggctaccattttgatctgaa

aggcgatgagtggatttgcgatcgcagcggcgaaaccttctgggatttgctggaacaggc

ggcgacgcagcaggcgggtgaaacagtcagtttccgctaaatggtggataagtcacaaga

aacgacgcactttggttttcagaccgtcgcgaaggaacaaaaagcggatatggtcgccca

cgttttccattccgtggcatcaaaatacgatgtcatgaatgatttgatgtcatttggtat

tcatcgtttgtggaaacgattcacgattgattgcagcggcgtacgccgtgggcagaccgt

gctggatctggctggtggcaccggcgacctgacagcgaaattctcccgcctggtcggaga

aactggcaaagtggtccttgctgatatcaatgaatccatgctcaaaatgggccgcgagaa

gctgcgtaatatcggtgtgattggcaacgttgagtatgttcaggcgaacgctgaggcgct

gccgttcccggataacacctttgattgcatcaccatttcgtttggtctgcgtaacgtcac

cgacaaagataaagcactgcgttcaatgtatcgcgtgctgaaacccggcggccgcctgct

ggtgcttgagttctcgaagccaattatcgagccgctgagcaaagcctatgatgcatactc

cttccatgtgctgccgcgtattggctcactggtcgcgaacgacgccgacagctaccgtta

tctggcagaatccatccgtatgcatcccgatcaggataccctgaaagccatgatgcagga

tgccggattcgaaagtgtcgactactacaatctgacggcaggggttgtggcgctgcatcg

tggttataagttctgaatgacgccaggtgaagtacggcgcctatatttcatcattcgcac

ttttttaagctacggacttgatgaactgatccccaaaatgcgtatcaccctgccgctacg

gctatggcgatactcattattctggatgccaaatcggcataaagacaaacctttaggtga

gcgactacgactggccctgcaagaactggggccggtatggatcaagttcgggcaaatgtt

atcaacccgccgcgatctttttccgccgcatattgccgatcagctggcgttattgcagga

caaagtcgctccgtttgatggcaagctggcgaagcagcagattgaagctgcaatgggcgg

cttgccggtagaagcgtggtttgacgattttgaaatcaagccactggcttctgcttctat

cgcccaggttcataccgcgcgattgaaatcgaatggtaaagaggtggtgattaaagtcat

ccgcccggatattttgccggtcattaaagcagacctgaaacttatctaccgtctggctcg

ctgggtgccgcgtttgctgccggatggtcgccgtctacgcccaaccgaagtggtgcgcga

gtacgaaaagaccttgattgatgaactgaatttgctgcgggaatctgccaacgctattca

gcttcggcgcaattttgaagacagcccgatgctctacatcccggaagtttaccctgacta

ttgtagtgaagggatgatggtgatggagcgcatttacggcattccggtgtctgatgttgc

ggcgctggagaaaaacggcactaacatgaaattgctggcggaacgcggcgtgcaggtgtt

cttcactcaggtctttcgcgacagctttttccatgccgatatgcaccctggcaacatctt

cgtaagctatgaacacccggaaaacccgaaatatatcggcattgattgcgggattgttgg

ctcgctaaacaaagaagataaacgctatctggcagaaaactttatcgccttctttaatcg

cgactatcgcaaagtagcagagctacacgtcgattctggctgggtgccaccagataccaa

cgttgaagagttcgaatttgctattcgtacggtctgtgaacctatctttgagaaaccgct

ggccgaaatttcgtttggacatgtactgttaaatctgtttaatacggcgcgtcgcttcaa

tatggaagtgcagccgcaactggtgttactccagaaaaccctgctctacgtcgaaggggt

aggacgccagctttatccgcaactcgatttatggaaaacggcgaagcctttcctggagtc

gtggattaaagatcaggtcggtattcctgcgctggtgagagcatttaaagaaaaagcgcc

gttctgggtcgaaaaaatgccagaactgcctgaattggtttacgacagtttgcgccaggg

caagtatttacagcacagtgttgataagattgcccgcgagcttcagtcaaatcatgtacg

tcagggacaatcgcgttattttctcggaattggcgctacgttagtattaagtggcacatt

cttgttggtcagccgacctgagtgggggctgatgcccggctggttaatggcaggtggtct

gatcgcctggtttgtcggttggcgcaaaacacgctgagtgaaaacattaattcttttctc

aacaagggacggacaaacgcgcgagattgcctcctacctggcttcggaactgaaagaact

ggggatccaggcggatgtcgccaatgtgcaccgcattgaagaaccacagtgggaaaacta

cgaccgtgtggtcattggtgcttctattcgctatggtcactaccactctgcgttccagga

atttgtcaaaaagcatgcgacgcggctgaattcgatgccgagcgccttttactccgtaaa

cctggtggcgcgtaaaccggagaagcgcactccacagaccaacagctacgcgcggaagtt

tctgatgaactcgcaatggcgtcccgatcgctgcgcggtcattgccggggcgctgcgtta

cccacgttatcgctggtacgaccgttttatgatcaagctgattatgaagatgtcaggcgg

tgaaacggatacgcgcaaagaagttgtctataccgattgggagcaggtggcgaatttcgc

ccgagaaatcgcccatttaaccgacaaaccgacgctgaaataaatgattgcattaattca

acgcgtaacccgtgccagcgtcaccgtggagggagaagtgacgggcgaaattggcgcggg

acttttggtgttattgggtgtcgaaaaggatgacgacgaacagaaagcaaaccgtctgtg

cgagcgtgtgctcggctaccgcatctttagcgatgccgaaggcaagatgaatctcaacgt

gcaacaggcgggcggcagtgtgctggtggtttcccagtttaccctcgccgcagataccga

acgggggatgcgcccaagtttctccaaaggtgcatcaccggatcgcgcagaggcgttata

tgactatttcgtcgaacgctgccgtcagcaagagatgaacacgcaaacaggacgcttcgc

tgcggatatgcaggtatcgctggtcaatgatggccccgtgacattctggttgcaggtatg

aatgagtattcgcataatcccgcaagatgagctgggttcgagcgagaaacgtacggcgga

tatgattccgccgttattgttccctcggctcaagaatttatacaaccgccgcgccgagcg

tctgcgcgagctggcagaaaataatccgctgggtgattacctgcgctttgctgcgcttat

cgcccacgcccaggaagtggtgctgtacgaccatccgctggagatggatctgactgcacg

cattaaagaagccagcgcacaaggcaagcctccgctggatattcacgttctgccgcgtga

taagcactggcaaaagctgctgatggcgctaattgctgagctgaaacctgaaatgagcgg

cccggcgctggcagtgattgagaatctggagaaggcatcgactcaggagctggaagatat

ggccagcgcactgtttgcctctgatttctcatccgtcagtagcgataaagcgccgtttat

ctgggctgcactgtcgctctactgggcgcagatggccaatctgatccccggcaaagcccg

cgctgaatacggcgaacaacgtcaatattgcccggtatgtggctctatgccggtgtccag

catggtgcaaattggcaccactcagggtctgcgttacctgcactgcaacctgtgtgaaac

cgaatggcacgtagtgcgcgtaaaatgcagcaactgtgagcagagcggcaaactgcatta

ctggtcgctggatgacgaacaggccgcgattaaagccgaaagctgcgatgactgcggcac

ttacctgaaaattctctatcaggaaaaagaaccgaaagttgaagccgtggcagatgacct

cgcctctctggtactggacgctcgaatggagcaagaaggctatgcccgcagttccatcaa

cccgttcctgtttccgggtgaaggggagtaaatgaagccagggtgtacgctgttttttct

cttatgttctgcattaaccgttacaacaacggcgcatgcgcaaacaccagatacggcaac

gaccgcgccttatctgctggctggagcccctactttcgatctctccatcagccagtttcg

agaagactttaacagccagaatcccagcctgccactgaacgaatttcgtgccatcgacag

cagtcccgacaaagccaatctcactcgtgctgccagtaaaattaacgagaacttgtatgc

ttctacagcgctggagcgcggtaccttaaaaatcaaaagcattcaaatgacctggctacc

catccaggggccagagcaaaaagccgcgaaagcgaaagctcaggaatatatggcagcggt

gatccgcacactcaccccattaatgaccaaaacacaaagccagaaaaaactgcagtcgct

actaacggcggggaaaaacaaacgttattacaccgagacagaaggtgcactgcgttatgt

tgtcgcggacaacggcgaaaaggggctgaccttcgctgttgaaccgattaagctggcgct

atctgaatcgcttgaaggtttgaataaatgaatggcttataaacacattggcgtggcaat

ttccgggaatgaagaagatgccttactggtgaataaagccctggagctcgccagacataa

tgacgctcacctgacgttaattcatattgatgatggcttaagcgagttgtacccgggtat

ctacttccctgcaacagaagatattcttcaattgttgaagaataagtcggataacaagct

gtataaactgacgaaaaatattcaatggccgaagacaaaactgcgtattgaacgcggaga

aatgccggaaacactgctggaaattatgcaaaaagagcagtgcgacctccttgtctgtgg

tcatcaccactcatttatcaaccgtttgatgccggcatatcgcgggatgatcaataagtt

gagtgcggatttgctcatcgtgccgtttatcgataagtaaatggctgattgggtaacagg

caaagtcactaaagtgcagaactggaccgacgccctgtttagtctcaccgttcacgcccc

tgtgcatccgtttaccgccgggcaatttaccaagcttggccttgaaatcgacggcgaacg

cgtccaacgcgcctactcctatgttaactcgcccgataatcccgatctggagttttacct

ggtcaccgtccccgatggcaaattaagcccacgattggcggcactgaaaccaggagatga

agtgcaggtggttagcgaagcagctggcttctttgttctggatgaagtgccagattgcga

aacgctatggatgctggcaaccggtacagcgattggcccttatttatcgattctacaact

aggcaaagatttagatcgcttcaaaaatctggtcctggtgcacgccgcacgttatgccgc

cgacttaagctatttgccactgatgcaagaactggaaaaacgctacgaaggaaaactgcg

cattcagacggtggtcagtcgggaaacggcagcggggtcgctcaccggacggataccggc

attaattgaaagtggtgaactggaaagcgcgattggcctgccgatgaataaagaaaccag

ccatgtgatgctgtgcggcaatccacagatggtgcgcgatacccaacagttgctgaaaga

gacccggcagatgacgaaacatttacgtcgccgaccgggccatatgacagcggagcatta

ctggtaaatgaaacaagcaaatcaagatagaggtacgctgctgctggcgttggttgctgg

cttatcgattaatggtactttcgcagcgctgtttagctccattgtgccattttctgtatt

cccgattatttccctggtgctgacggtttactgcctgcatcaacgttatcttaatcgcac

catgccggtaggcttgccgggtctggcagctgcctgttttattctcggcgtactgctgta

cagcacggtagttcgtgcggaatatccggatatcggctctaacttcttcccggcagtact

ctccgtcattatggtgttctggattggcgcgaagatgcgtaaccgtaagcaggaagttgc

tgagtaaatgagtgcgaataccgaagctcaaggaagcgggcgcggcctggaagcgatgaa

gtgggtcgttgtggtggcattgctcctggtggcgattgtcggcaactacctttatcgcga

cattatgctgccgctgcgtgcgctggccgtagtaattctgattgctgcagcgggtggtgt

cgcgctgttaacaacaaaaggtaaagctaccgttgcttttgcccgtgaagcgcgtaccga

agtccgtaaggtcatttggccgactcgccaggaaacattgcacaccacgctgattgtggc

tgcggttaccgcagtaatgtcactgatcctgtggggactggatggtattctggttcgcct

ggtatcctttatcactggcctgaggttctgaatgtctgaagctcctaaaaagcgctggta

cgtcgttcaggcgttttccggttttgaaggccgcgtagcaacgtcgctgcgtgagcatat

caaattacacaacatggaagatttgtttggtgaagtcatggtaccaaccgaagaagtggt

tgaaatccgtggcggtcagcgtcgcaaaagcgaacgtaaattcttccctggctacgtcct

cgttcagatggtgatgaacgacgcgagctggcacctggtgcgcagcgtaccgcgtgtgat

gggcttcatcggcggtacttccgatcgtcctgcgccaatcagcgataaagaagtcgatgc

gattatgaaccgcctgcagcaggttggtgataagccgcgtccgaaaacgctgtttgaacc

gggtgaaatggtccgtgttaatgatggtccgttcgctgacttcaacggtgttgttgaaga

agtggattacgagaaatctcgtctgaaagtgtctgtttctatcttcggtcgtgcgacccc

ggtagagctggacttcagccaggttgaaaaagcctaaatgcttaaccagctcgataacct

gacggagcgcgtcagaggaagtaacaaactggttgatcgctggctacatgtacgtaagca

tctgctcgtggcttactacaatctggttggcattaagcctggcaaagaatcgtacatgag

gctaaacgaaaaagcccttgatgatttttgtcagagcctggtcgattacttgtctgccgg

acatttcagtatttatgagcgcattcttcataagctggaaggcaacgggcaactcgcgcg

cgccgcgaagatttggccgcaactcgaagccaatacccaacagattatggattactacga

ttccagcctggaaaccgctatcgatcatgataattaccttgagtttcaacaggttttatc

tgacatcggcgaggcgctggaagcgcgctttgtgctggaagataagctgattctgctggt

gcttgacgctgcccgcgtcaaacatcctgcttgaatgttacaaaacccaattcatctgcg

tctggagcgcctggaaagctggcagcatgtcactttcatggcttgcttatgcgaacgcat

gtaccccaattacgccatgttctgccagcaaaccggttttggtgatgggcaaatttaccg

tcgtattctcgatctcatctgggaaacgctgaccgttaaagatgcaaaagtaaatttcga

cagccaactggagaaatttgaagaagcgattccttcagccgacgatttcgatctgtacgg

cgtttatccggcaatcgatgcctgtgtggcgttaagtgaactggtccattcgcgtttgag

tggtgaaacgctcgaacacgcggtggaagtgagtaagacctccatcacgaccgttgcgat

gctggaaatgactcaggctggtcgcgaaatgagcgatgaagagctcaaagaaaacccagc

tgtagagcaagaatgggatattcagtgggaaatattccgacttttagctgagtgcgaaga

acgcgatatcgagctgataaaaggccttagggcagacctgcgtgaggcgggtgagagcaa

tattggtataatttttcagcaataaatggccaataataccactggattcacccgaattat

caaagctgctggctattcctggaaaggtttacgcgctgcatggatcaacgaagcggcatt

ccgtcaggaaggcgtagcggtattgttggcggtggtcatcgcctgctggctggatgtgga

cgcgattacccgcgtgctgcttatcagctccgtgatgctggtgatgattgtggaaatcct

caatagcgccatcgaagcagtggttgaccgaattggctctgaataccatgagctttccgg

acgcgctaaagatatgggatccgccgcggtgctgattgcgattatcgtcgccgtgattac

ctggtgcatcctgttatggtcgcattttggataaatgaaagcgttaacggccaggcaaca

agaggtgtttgatctcatccgtgatcacatcagccagacaggtatgccgccgacgcgtgc

ggaaatcgcgcagcgtttggggttccgttccccaaacgcggctgaagaacatctgaaggc

gctggcacgcaaaggcgtaattgaaattgtttccggcgcatcacgcgggattcgtctgtt

gcaggaagaggaagaagggttgccactggtaggtcgtgtggctgccggtgaaccgcttct

ggcgcaacagcatattgaaggtcattatcaggtcgatccttccttgttcaagccgaatgc

tgatttcctgctgcgcgtcagcgggatgtcgatgaaagatatcggcattatggatggcga

cttgctggcagtgcataaaactcaggatgtacgtaacggtcaggtcgttgtcgcacgtat

tgatgacgaagttaccgttaagcgcctgaaaaaacagggcaataaagtcgaactgttgcc

agaaaatagcgagtttaaaccaattgtcgttgaccttcgtcagcagagcttcaccattga

agggctggcggttggcgttattcgcaacggcgactggctgtaaatgtggtatcaaaagac

gctcacgcttagcgccaaatctcgtgggtttcatctggtaacggatgaaattctgaatca

gctggctgatatgccgcgcgttaacatcggcttactgcatctgttgctgcaacatacctc

cgcctctctgacacttaatgagaactgcgatcccaccgtacgccacgacatggagcgttt

tttcctccgcaccgttcccgataatggaaactatgagcatgactatgagggagcagacga

tatgccttctcatatcaaatcctcaatgctgggaacatcgcttgtattgccggtgcataa

agggcgtattcagaccggcacctggcaaggcatttggctgggggaacatcgcatccacgg

cggatcgcgtcgcatcatcgcgacactacaaggggagtaaatgaccatttcggagttgct

acaatattgcatggcaaaaccaggcgcagaacagagcgtgcataacgactggaaagcgac

gcagatcaaagtggaagatgtactgtttgcgatggtgaaagaagtagaaaatcgcccagc

tgtttcgctgaaaaccagcccggagctggcggagctgctacgtcagcagcacagcgatgt

gcgtccaagccgccatctgaataaagcgcactggagcaccgtgtatctcgacggttcgct

gccagattcgcaaatctattatctggtggatgcgtcttatcagcaggcggtgaatttact

gccggaagaaaaacgtaaattgctggtgcaactctgaatgaggatttgcagcgaccaacc

ttgtattgttttattgactgaaaaagatgtctggataagggtgaatgggaaagaacctat

tagccttaaagctaaccatatggcgttattaaattgtgaaaataatattatcgacgtctc

ctctcttaacaacactttggttgctcatattagtcacgacatcatcaaagattacctccg

gtttctgaataaagatctctcgcaaataccagtatggcaacgtagcgctacgcccattct

caccctgccatgcctgacaccagacgtctttcgtgttgccgcgcaacacagcatgatgcc

cgcagaaactgagtcagaaaaggaacgaacacgcgcattattattcactgtgctatcccg

ttttctcgacagtaaaaaattcctttcattaatgatgtatatgttacgtaattgtgtaag

tgacagcgtttatcaaattattgaaagcgatattcacaaagactggaatcttagtatggt

agccagttgtttatgtcttagcccaagtctgttaaagaaaaagctgaaaagcgaaaacac

cagttatagccaaataatcaccacctgccgcatgcgttatgccgtaaatgaattaatgat

ggacggtaaaaatatctctcaggtatcacagtcctgcggctacaacagtacgtcgtactt

tatttctgtctttaaagacttctacggtatgacgccgctgcattatgttagtcagcacag

agaacgcactgtcgcctgaatgctagttgtagaactcatcatagttttgctggcgatctt

cttgggcgccagattggggggaataggtattggttttgcaggcggattgggggtgctggt

tcttgccgctattggcgttaaacccggtaacatcccgttcgatgtcatttccattatcat

ggcggttatcgccgctatttctgccatgcaggttgctggcggtctggactatctggttca

tcagacagaaaagctgctgcgccgtaacccgaaatacatcacgatcctcgcaccgatcgt

gacctatttcctgactatctttgctggtactggcaacatctctctggcgacactgccagt

tatcgctgaagttgcgaaggaacaaggcgttaaaccttgccgtccgctgtctactgcagt

ggtatccgcgcagattgcgatcaccgcatcgccaatctcagcggcagtggtttacatgtc

ttccgtgatggaaggtcatggcatcagctacctccatctgctatccgtggtcatcccgtc

caccctgctggcggttctggtgatgtccttcctggtcactatgctgttcaactccaaact

ctctgacgatccgatttatcgcaagcgtctggaagagggcctggttgaactgcgcggtga

aaagcagattgaaatcaaatccggtgcaaaaacgtccgtctggctgttcctgctgggcgt

agttggcgtggttatctatgcaatcatcaacagcccaagcatgggtctggttgaaaaacc

gctgatgaacaccaccaacgcaatcctgatcatcatgctcagcgttgcaactctgaccac

cgttatctgtaaagtcgataccgacaacatcctcaactccagcaccttcaaagcaggtat

gagcgcctgtatttgtatcctgggtgttgcgtggctgggcgatactttcgtttccaacaa

catcgactggatcaaagataccgctggtgaagtgattcagggtcatccgtggctgctggc

cgtcatcttcttctttgcttctgctctgctgtactctcaggctgcaaccgcaaaagcact

gatgccgatggctctggcactgaacgtttctccgctgaccgctgttgcttctttcgctgc

ggtgtctggtctgttcattctgccgacctacccgacgctggttgctgcggtacagatgga

tgacacgggtactacccgtatcggtaaattcgtcttcaaccatccgttcttcatcccggg

tactctgggtgttgccctggccgtttgcttcggcttcgtgctgggtagcttcatgctgta

aatgcgcttactccctctcgttgccgcagcgacagctgcatttctggtcgttgcctgcag

ttctcctacgccgccgcgtggcgtgaccgtagtgaataatttcgacgctaaacgctatct

cggtacctggtatgagattgcccgttttgatcaccgctttgaacgtggactggaaaaagt

caccgcaacatacagcctgagggatgatggcggcctgaatgtcattaataaaggctataa

ccctgacagaggaatgtggcagcagagtgaagggaaagcgtactttaccggcgcaccaac

tcgcgctgcgctgaaagtgtcattctttggtcctttctatggcggttataacgttattgc

actcgatcgggaataccaccatgcgctggtttgcggcccggaccgcgactacctgtggat

actctcccgcacgccaaccatttctgacgaagtgaaacaggagatgctggcagtcgcgac

ccgggaagggtttgatgtcagtaagtttatttgggtacagcagcccggtagttagatgac

gactaaacgtaaaccgtatgtacggccaatgacgtccacctggtggaaaaaattgccgtt

ttatcgcttttacatgctgcgcgaaggcacggcggttccggctgtgtggttcagcattga

actgattttcgggctgtttgccctgaaaaatggcccggaagcctgggcgggattcgtcga

ctttttacaaaacccggttattgtgatcattaacttgatcactctggcggcagctctgct

gcacaccaaaacctggtttgaactggcaccgaaagcggccaatatcattgtaaaagacga

aaaaatgggaccagagccaattatcaaaagtctctgggcggtaactgtagttgccaccat

cgtaatcctgtttgttgccctgtactggtaaatgatgaatcgagtaattccgctccctga

tgagcaggcaacattagacctgggcgagcgggtagcgaaagcctgcgatggcgcaaccgt

aatctatctgtatggcgatttaggcgcaggtaaaaccacctttagccggggctttttgca

ggctttgggtcatcagggtaatgtcaaaagccccacttatacgctggtcgaaccctatac

gctcgacaacttaatggtttatcactttgatttgtaccgccttgccgatcccgaggagct

ggagtttatggggatccgcgattattttgccaacgatgccatctgcctggtggagtggcc

acaacaaggtacaggtgttcttcctgacccggatgtcgaaatacacattgattatcaggc

acaaggccgtgaggcgcgcgtaagtgcggtttcctctgcgggtgaattgttgctggcgcg

tttagccggttaaatggctaaggggcaatctttacaagatccgttcctgaacgcactgcg

tcgggaacgtgttccagtttctatttatttggtgaatggtattaagctgcaagggcaaat

cgagtcttttgatcagttcgtgatcctgttgaaaaacacggtcagccagatggtttacaa

gcacgcgatttctactgttgtcccgtctcgcccggtttctcatcacagtaacaacgccgg

tggcggtaccagcagtaactaccatcatggtagcagcgcgcagaatacttccgcgcaaca

ggacagcgaagaaaccgaataaatggcgtggaatcagcccggtaataacggacaagaccg

cgacccgtggggaagcagcaaacctggcggcaactctgagggaaatggaaacaaaggcgg

tcgcgatcaagggccacctgatttagatgatatcttccgcaaactgagcaaaaagctcgg

tggtctgggcggcggtaaaggcaccggatctggcggtggcagttcatcgcaaggcccgcg

cccgcagcttggcggtcgtgtcgttaccatcgcagcggcagcgattgtcattatctgggc

ggccagtggtttctataccattaaagaagccgaacgcggcgtggtaacacgctttggtaa

attcagccatctggttgagccgggtctgaactggaaaccgacgtttatcgacgaagtcaa

accggtgaacgtggaagccgtgcgtgaactggccgcttctggtgtgatgctgacgtcgga

cgagaacgtagtgcgcgttgagatgaacgtgcagtaccgcgtcaccaatccggaaaaata

tctgtatagcgtgaccagcccggatgacagcctgcgtcaggctaccgacagcgccctgcg

tggagttatcggtaaatacaccatggaccgcattctgacggaaggtcgtaccgtgattcg

tagcgatactcagcgcgaactggaagagacgattcgtccgtatgacatgggcatcactct

gctggacgtcaacttccaggcagctcgtccgccggaagaagtaaaagcggcgtttgacga

tgcgattgccgcgcgtgaaaacgaacagcaatacattcgtgaagcagaagcgtataccaa

cgaagttcagccgcgtgcgaacggtcaggcgcaacgtatcctcgaagaggcgcgtgcgta

caaggcccagaccatcctggaagctcagggtgaagtggcgcgctttgctaaacttctgcc

ggaatataaagccgcgccggaaattactcgcgagcgtctgtatatcgagacgatggaaaa

agtgttgggtaacacccgcaaagtgctggttaacgacaaaggtggcaacctgatggttct

gccgttagaccagatgctgaaaggtggtaacgcccctgcggcgaagagcgataacggtgc

cagcaatctgctgcgtctgccgccagcctcttcctccacaaccagtggagcaagcaacac

gtcgtccaccagtcagggcgatattatggaccaacgccgcgccaacgcgcagcgtaacga

ctaccagcgtcagggggaataaatgcgtaagtcagttatcgcgattatcatcatcgtgct

ggtagtgctttacatgtctgtctttgtcgtcaaagaaggtgagcgcggtattacgctgcg

ttttggtaaggtactgcgtgacgatgacaacaaacctctggtttatgagccgggtctgca

tttcaagataccgttcattgaaacggtgaaaatgctcgacgcacgtattcagaccatgga

caaccaggccgaccgctttgtgaccaaagagaagaaagacctgatcgtcgactcttacat

caaatggcgcatcagcgatttcagccgttactacctggcaacgggtggtggcgacatttc

gcaagcggaagtgctgttgaaacgtaagttctctgaccgtctgcgttctgaaattggtcg

cctggacgtgaaagatatcgtcactgattcccgtggtcgtctgaccctcgaagtacgtga

cgcgctgaactccggttctgcgggtacagaagatgaagttactaccccggcggcagataa

cgccattgccgaagcggcagagcgcgtaacggctgagacgaagggcaaagttccggtcat

caacccgaacagtatggcggcgctgggtattgaagttgtcgatgtgcgtatcaagcagat

caacctgccgaccgaagtgtctgaagcgatctacaaccgtatgcgcgccgagcgtgaagc

ggtagcgcgtcgtcaccgttcacaaggtcaggaagaagcggaaaaactgcgcgcgactgc

cgactatgaagtgaccagaacgctggcagaagctgagcgtcagggccgcattatgcgtgg

tgaaggcgatgccgaagcagccaaactgtttgctgatgcattcagtaaagatccggactt

ctacgcattcatccgtagcctgcgtgcttatgagaacagcttctctggcaatcaggacgt

gatggtcatgagcccggatagcgatttcttccgctacatgaagacgccgacttccgcaac

gcgttaagtgcagttaacgagtttcactgattacggattacgtgcgctgatctacatggc

gtcattgccagaagggcggatgaccagtatttctgaagtgactgacgtctacggcgtctc

ccgtaatcatatggtcaaaataatcaatcaacttagtcgtgccggctacgtgactgctgt

tcgtggaaaaaatggcggcattcgcctgggtaaaccggcgagtgcgatacgtattggtga

tgtggtgcgcgagctggagcccttatcgctggtgaattgcagcagtgagttttgccacat

tacacctgcctgtcggttgaaacaggcgctttctaaggccgtgcaaagttttcttacgga

actggataactacacgcttgccgatttggttgaagagaatcaaccgctttataaattatt

gctggtggagtgaatgcgaatatttgtctacggcagtttacgccacaaacaaggcaacag

tcactggatgaccaatgcccagttactgggcgatttcagtatcgataactaccagttgta

tagcctgggccactatccaggcgcagttccggggaacggaacagtacacggtgaagttta

tcgtattgacaacgccacgctggccgaacttgatgccttgcgcaccaggggcggtgaata

cgcgcgccagttgattcagacgccgtacgggagtgcatggatgtacgtttatcaacgacc

cgtcgatggattaaagctaattgaaagcggcgactggttagacagggataagtaaatgag

cttactcaacgtccctgcgggtaaagatctgccggaagacatctacgttgttattgagat

cccggctaacgcagatccgatcaaatacgaaatcgacaaagagagcggcgcactgttcgt

tgaccgcttcatgtccaccgcgatgttctatccgtgcaactacggttacatcaaccacac

cctgtctctggacggtgacccagttgacgtactggtcccgactccgtacccgctgcagcc

gggttctgtgatccgttgccgtccggttggcgttctgaaaatgaccgacgaagccggtga

agatgcgaaactggttgcggttccgcacagcaagctgagcaaagaatacgatcacattaa

agacgttaacgatctaccagagctgctgaaagcgcagatcgctcacttcttcgagcacta

caaagacctcgaaaaaggcaagtgggtgaaagttgaaggttgggaaaacgcagaagccgc

taaagctgaaatcgttgcctccttcgagcgcgccaagaataaataaatggctaatccgct

atatcagaaacatatcatttccataaacgaccttagtcgcgatgaccttaatctggtgct

ggcgacagcggcgaaactgaaagcaaacccgcaaccagagctgttgaagcacaaagtcat

tgccagctgtttcttcgaagcctctacccgtacccgcctctctttcgaaacttccatgca

ccgcctgggtgccagcgtggtgggcttctccgacagcgccaatacatcactgggtaaaaa

gggcgaaacgctggccgataccatttcggttatcagcacttacgtcgatgcgatagtgat

gcgtcatccgcaggaaggtgcggcgcgcctggccaccgagttttccggcaatgtaccggt

actgaatgccggtgatggctccaaccaacatccgacgcaaaccttgctggacttattcac

cattcaggaaacccaggggcgtctggacaatctccacgtcgcaatggttggtgacctgaa

atatggccgcaccgttcactccctgactcaggcgttagcgaagttcgacggcaaccgttt

ttacttcatcgcgccggacgcgctggcaatgccgcaatacattctggatatgcttgatga

aaaagggatcgcatggagtctgcacagctctattgaagaagtgatggcggaagtagacat

cctgtacatgacccgcgtgcaaaaagagcgtctggacccgtccgagtacgccaacgtgaa

agcgcagtttgttcttcgcgccagcgatctccacaacgccaaagccaatatgaaagtact

gcatccgctgccgcgtgttgatgagattgcgacggatgttgataaaacgccacacgcctg

gtacttccagcaggcaggcaacgggattttcgctcgccaggcgttactggcactggttct

gaatcgcgatctggtactgtaaatggcaaacccggaacaactggaagaacagcgtgaaga

aacacgtttgattattgaagaattactggaagatggcagcgatccggacgcgctgtacac

catcgaacaccatctttccgcagacgatctggaaaccctggaaaaagcagcagttgaagc

gtttaaactcggttacgaagtgaccgatccagaagagctggaagttgaagatggtgatat

cgtgatttgctgcgacatcctcagcgagtgcgcgttgaatgccgatctgatcgatgtcca

ggttgaacaactgatgacgctggcggagaaatttgacgttgagtacgacggttggggcac

ttactttgaagatcccaacggcgaagacggcgacgatgaagattttgtcgatgaagacga

tgacggggttcgccactaaatgaaaaacgcgacgttctaccttctggacaatgacaccac

cgtcgatggcttaagcgccgttgagcaactggtgtgtgaaattgccgcagaacgttggcg

cagcggtaagcgcgtgctcatcgcctgtgaagatgaaaagcaggcttaccggctggatga

agccctgtgggcgcgtccggcagaaagctttgttccgcataatttagcgggagaaggacc

gcgcggcggtgcgccggtggagatcgcctggccgcaaaaacgcagcagcagcccgcgcga

tatattgattagtctgcgaacaagctttgcagattttgccaccgctttcacagaagtggt

agacttcgttccttatgaagattctctgaaacaactggcgcgcgaacgctataaagccta

ccgcgtggctggtttcaacctgaatacggcaacctggaaataaatgaaaaacgttggcga

cctgatgcaacgcctgcaaaaaatgatgcctgcccatatcaaacccgcattcaaaacggg

tgaagaactactggcctggcaaaaagaacaaggggcgatccgctctgccgctctcgaacg

tgaaaatcgggcgatgaaaatgcagcgcacctttaaccgctccggtattcgtccactgca

tcagaactgctcctttgagaactatcgcgttgagtgtgaagggcagatgaatgcgttaag

caaagcgcgccagtatgtcgaagagtttgacggcaacatcgccagctttatcttttctgg

taagccaggaaccggcaaaaaccatctggcggcggcaatctgcaacgagctgctgctacg

cggtaaatccgtattgatcatcaccgtggccgatattatgtcggcgatgaaagatacctt

caggaatagcggtaccagcgaagaacaactgcttaacgatctgagcaacgttgatctgct

ggtgatcgatgagatcggcgtgcagaccgaatcgaaatacgaaaaagtgatcatcaacca

gatcgtcgatcgccgctcttcttccaaacgcccaaccgggatgctgaccaatagtaatat

ggaagagatgaccaagttgctgggcgaacgcgtgatggaccgtatgcgcctgggtaacag

tttgtgggtgatcttcaactgggatagctaccgtagccgtgtaacaggtaaagagtatta

aatgaacacgatttcttccctcgaaacgactgatttaccagcggcttaccacattgaaca

acgcgcccacgcctttccgtggagtgaaaaaacctttgccagcaaccagggcgagcgtta

tctcaactttcagttaacgcaaaacggcaaaatggcggcgtttgcgattacgcaagtggt

gctggatgaagctacattgttcaatattgcggtcgatcctgactatcagcgtcagggatt

gggaagggcgctgctggaacatctgatcgacgaactggaaaaacgcggcgtggcgacact

atggctggaagtccgtgcctcaaatgctgccgccattgccctgtacgaaagtttaggctt

taacgaggcgacgattcgccgcaattactaccccaccacggacggtcgcgaagacgccat

catcatggcgttgccaatcagtatgtaaatggatcaggccggcattattcgcgacctttt

aatctggctggaaggtcatctggatcagcccctgtcgctcgacaatgtagcggcgaaagc

aggttattccaagtggcacttacagagaatgtttaaagatgtcactggccatgctattgg

cgcgtatattcgtgctcgtcgtttgtcgaaatcggcggtcgcactacgcctgactgcgcg

tccgattctggacatcgcgctgcaataccgcttcgactctcaacagacatttacccgcgc

attcaagaagcagtttacccagactcctgcactttaccgccgttctcctgaatggagcgc

ctttggtattcgcccgccgctgcgtctgggtgaattcactatgccagagcataaatttgt

caccctggaagatacgccgctgattggtgttacccagagctactcctgttcgctggagca

aatctctgatttccgccatgaaatgcgttatcagttctggcacgattttctcggcaacgc

gccgaccattccgccagtgctctacggcctgaatgaaacgcgtccgagtcaggataaaga

cgacgaacaagaggtattctataccaccgcgttagcccaggatcaggcagatggctatgt

actgacggggcatccggtgatgctgcagggcggcgaatatgtgatgtttacctatgaagg

tctgggaaccggcgtgcaggagtttatcctgacggtatacggaacgtgcatgccaatgct

caacctgacgcgccgtaaaggtcaggatattgagcgatactacccggcagaagacgccaa

agcgggagatcgcccaattaatctgcgctgtgaactgctgattccgatccgtcgttaaat

gaaatacaagcatttgatcctgtctttaagcctgataatgctggggccattggctcatgc

agaagagattggttcggtcgacaccgtatttaaaatgatcggcccggatcacaaaattgt

tgtggaagcctttgacgatcccgatgtgaaaaatgtcacctgttatgtgagccgggcgaa

aacaggtggtattaaagggggattgggtctggcggaagatacctccgatgcggctatttc

ttgtcagcaagtcgggccgattgaactgtcggatcgtattaaaaacggcaaagctcaggg

cgaggtagtattcaaaaaacgcacgtccctggtctttaagtcgttacaggtcgtgcgctt

ttatgacgccaaacgcaacgcgctcgcttatctggcttactccgacaaagttgtagaagg

ttcgccgaaaaacgcgattagcgcggttcctgtcatgccgtggcggcaataaatgcagac

cccgcacattcttatcgttgaagacgagttggtaacacgcaacacgttgaaaagtatttt

cgaagcggaaggctatgatgttttcgaagcgacagatggcgcggaaatgcatcagatcct

ctctgaatatgacatcaacctggtgatcatggatatcaatctgccgggtaagaacggtct

tctgttagcgcgtgaactgcgcgagcaggcgaatgttgcgttgatgttcctgactggccg

tgacaacgaagtcgataaaattctcggcctcgaaatcggtgcagatgactacatcaccaa

accgttcaacccgcgtgaactgacgattcgtgcacgcaacctgctgtcccgtaccatgaa

tctgggtactgtcagcgaagaacgtcgtagcgttgaaagctacaagttcaatggttggga

actggacatcaacagccgttcattgatcggccctgatggcgagcagtacaagctgccgcg

cagcgagttccgcgccatgcttcacttctgtgaaaacccaggcaaaattcagtcccgtgc

tgaactgctgaagaaaatgaccggccgtgagctgaaaccgcacgaccgtactgtagacgt

gacgatccgccgtattcgtaaacatttcgaatctacgccggatacgccggaaatcatcgc

caccattcacggtgaaggttatcgcttctgcggtgatctggaagattaa

>CP006262.fa.conservedCDS.fa

atggttaaagtttatgccccggcttccagtgccaatatgagcgtcgggtttgatgtgctc

ggggcggcggtgacacccgttgatggtgcattgctcggagatgtagtcacggttgaggcg

gcagagacattcagtctcaacaacctaggacgctttgccgataagctgccgtcagaacca

cgggaaaatatcgtttatcagtgctgggagcgtttttgccaggagcttgggaagcaaatt

ccagtggcgatgactctggaaaagaatatgccgatcggttcgggcttaggctccagcgcc

tgttcggtggtcgcggcgctgatggcgatgaatgaacactgtggcaagccgcttaatgac

actcgtttgctggctttgatgggcgagctggaaggacgaatctccggcagcatacattac

gacaacgtggcaccgtgttttcttggtggtatgcagttgatgatcgaagaaaacgacatc

atcagccagcaagtgccagggtttgatgagtggctgtgggtgctggcgtatccggggatt

aaagtctcgacggcagaagcccgggctattttaccggcgcagtatcgccgccaggattgc

attgcgcacgggcgacatctggcaggcttcattcacgcctgctattcccgtcagcctgag

cttgccgcgaagctgatgaaagatgttatcgcagaaccctaccgtgaacggttactgcct

ggcttccggcaggcgcggcaggcggtcgcggaaatcggcgcggtagcgagcggtatctcc

ggctccggcccgaccttgttcgcgctgtgtgacaagccggataccgcccagcgcgttgcc

gactggttgggtaagaactacctgcaaaatcaggaaggttttgttcatatttgccggctg

gatacggcgggcgcacgagtactggaaaactaaatgggcaacactaagttggctaatccg

gcaccgctgggcctgatgggcttcggcatgaccaccattctgcttaacctgcacaacgtg

ggttatttcgctctggacggtattattcttgccatgggcattttctacggcggcatcgcg

caaatttttgccggtctgctggagtacaaaaaaggcaacactttcggtttaaccgcattc

acctcttacggttctttctggctgacgctggttgcgattctgctgatgccgaaactgggt

ctgaccgatgcgccaaatgcacagttccttggtgtctacctgggtctgtggggcgtattt

acgctgtttatgttcttcggcacgctgaaaggcgcacgcgttctgcaattcgttttcttt

agcctgaccgtgctgtttgccctgctggcgatcggtaacattgccggtaacgccgcaatc

atccactttgccggctggattggtctgatctgcggtgccagcgcaatctatctggcgatg

ggtgaagtactgaacgagcagtttggtcgcaccgttctgccgattggtgaatcccactaa

atgggtaaaataattggtatcgacctgggtactaccaactcttgtgtagcgattatggat

ggcaccactcctcgcgtgctggagaacgccgaaggcgatcgcaccacgccttctattatt

gcctatacccaggatggtgaaactctggttggtcagccggctaaacgtcaggcagtgacg

aacccgcaaaacaccctgtttgcgattaaacgcctgattggccgccgcttccaggacgaa

gaagtacagcgtgatgtttccatcatgccgttcaaaattattgctgctgataacggcgac

gcatgggtcgaagttaaaggccagaaaatggcaccgccgcagatttctgctgaagtgctg

aaaaaaatgaagaaaaccgctgaagattacctgggtgaaccggtaactgaagctgttatc

accgtaccggcatactttaacgatgctcagcgtcaggcaaccaaagacgcaggccgtatc

gctggtctggaagtaaaacgtatcatcaacgaaccgaccgcagctgcgctggcttacggt

ctggacaaaggtactggcaaccgtactatcgcggtttatgacctgggtggtggtactttc

gatatttctattatcgaaatcgacgaagttgacggcgaaaaaaccttcgaagttctggca

accaacggtgatacccacctgggtggtgaagacttcgacagccgtctgatcaactatctg

gttgaagaattcaagaaagatcagggcattgacctgcgcaacgatccgctggcaatgcag

cgcctgaaagaagcggcagaaaaagcgaaaatcgaactgtcttccgctcagcagaccgac

gttaacctgccgtacatcactgcagatgcgaccggtccgaaacacatgaacatcaaagtg

actcgtgcgaaactggaaagcctggttgaagatctggtaaaccgttccattgagccgctg

aaagttgcactgcaggacgctggcctgtccgtatctgatatcgacgacgttatcctcgtt

ggtggtcagactcgcatgccaatggttcagaagaaagttgctgagttctttggtaaagag

ccgcgtaaagacgttaacccggacgaagctgtagcaatcggtgctgctgttcaggggggt

gttctgactggtgacgtaaaagacgtactgctgctggacgttaccccgctgtctctgggt

atcgaaaccatgggcggtgtgatgacgacgctgatcgcgaaaaacaccactatcccgacc

aagcacagccaggtgttctctaccgctgaagacaaccagtctgcggtaaccatccatgtg

ctgcagggtgaacgtaaacgtgcggctgataacaaatctctgggtcagttcaacttggat

ggtatcaacccggcaccgcgcggcatgccgcagatcgaagttaccttcgatatcgatgct

gacggtatcctgcacgtttccgcgaaagataaaaacagcggtaaagagcagaagatcacc

atcaaagcttcttctggtctgaacgaagatgaaatccagaaaatggtacgcgacgcagaa

gctaacgccgaagctgaccgtaagtttgaagagctggtacagactcgcaaccagggcgac

catctgctgcacagcacccgtaagcaggttgaagaagcaggcgacaaactgccggctgac

gacaaaactgctatcgagtctgcactgactgcactggaaactgctctgaaaggtgaagac

aaagccgctatcgaagcgaaaatgcaggagctggcacaggtttcccagaaactgatggaa

atcgcccagcagcaacatgcccagcagcagactgccggtgctgatgcttctgcaaacaac

gcgaaagatgacgatgttgtcgacgctgaatttgaagaagtcaaagacaaaaaataaatg

agtcaatcgatctgttcaacagggctacgctggctgtggctggtggtagtcgtgctgatt

atcgatctgggcagcaaatacctgatcctccagaactttgctctgggggatacggtcccg

ctgttcccgtcgcttaatctgcattatgcgcgtaactatggcgcggcgtttagtttcctt

gccgatagcggcggctggcagcgttggttctttgccggtattgcgattggtattagcgtg

atcctggcagtgatgatgtatcgctcgaaggccacgcagaagctaaacaatatcgcttat

gcgctgattattggcggcgcgctgggcaacctgttcgaccgcctgtggcacggcttcgtt

gtcgatatgatcgacttctacgtcggcgactggcacttcgccaccttcaaccttgccgat

actgccatttgtgccggtgcggcactgattgtgctggaaggttttttgccttctaaagcg

aaaaaacaataaatgtctgaatctgtacagagcaatagcgccgtcctggtgcacttcacg

ctaaaactcgacgatggcaccaccgccgagtctacccgcaacaacggtaaaccggcgctg

ttccgcctgggcgatgcttctctttctgaagggctggagcaacacctgctggggctgaaa

gtgggcgataaaaccaccttctcactggagcccgatgcggcgtttggcgtgccgtcaccg

gacctgattcagtacttctcccgccgtgaatttatggatgcaggcgagccagaaattggc

gcaatcatgctttttaccgcaatggatggcagtgagatgcctggtgtgatccgcgaaatt

aacggcgactccattaccgttgatttcaaccatccgctggccgggcagaccgttcatttt

gatattgaagtgctggaaatcgatccggcactggaggcgtaaatgatcctcataatttat

gcgcatccgtatccgcatcattcccatgcgaataaacggatgcttgaacaggcaaggacg

ctggaaggcgtcgaaattcgctctctttatcaactctatcctgacttcaatatcgatatt

gccgccgagcaggaggcgctgtctcgcgccgatctgatcgtctggcagcacccgatgcag

tggtacagcattcctccgcttcttaaactttggatcgataaagttttctcgcacggctgg

gcttacggtcacggcggcacggcgctgcatggcaaacatttgctgtgggcggtgacgacc

ggcggcggggaaagccattttgaaattggtgcgcatccgggctttgatgtgctgtcgcag

ccgctacaggcgacggcaatctactgcgggctgaactggctgcctccgtttgccatgcac

tgcacctttatttgtgacgacgaaaccctcgaagggcaggcgcgccactataagcaacgt

ctgctggaatggcaggaggcacatcatggatagatgaataatcgagtccaccagggccac

ttagcccgtaaacgcttcgggcaaaactttctcaacgatcagttcgtgatcgacagcatt

gtttctgccattaacccgcagaagggtcaggcgatggtcgaaatcggccccggtctggct

gcattgaccgaaccggtcggcgaacgtctggaccaactgacggttatcgaacttgaccgc

gatctggcggcacgtctgcaaacgcatccattcttaggcccgaaactgacgatttatcag

caggatgcgatgacctttaactttggcgaactggccgagaaaatgggtcagccgctgcgt

gttttcggcaacctgccttataacatctccacgccgttgatgttccatctgtttagctat

actgatgccattgccgacatgcactttatgttgcaaaaagaggtggtgaatcgtctggtt

gcaggaccgaacagcaaggcgtatggtcgattaagcgtcatggcgcaatactattgcaac

gtgatcccagtgctggaagtacctccgtcagcctttacaccaccacccaaagtggattcc

gccgtcgtgcgcctggttcctcatgcaacgatgcctcacccggttaaagatgttcgcgtg

ttgagccgcatcaccaccgaagcctttaaccagcgtcgtaaaaccattcgtaacagcctc

ggcaacctgtttagcgtcgaggtgttaacgggaatggggatcgacccggcgatgcgagcg

gaaaatatctctgtcgcgcaatattgccagatggcgaactatctggcggagaacgcgcct

ttgcaggagagttaagtgaaactggatgaaatcgctcggctggcgggagtgtcgcggacc

actgcaagctatgttattaacggcaaagcgaagcaataccgtgtgagcgacaaaaccgtt

gaaaaagtcatagctgtggtgcgtgagcacaattaccacccgaacgccgtggcagctggg

cttcgtgctggacgcacacgttctattggtcttgtgatccccgatctggagaacaccagc

tatacccgcatcgctaactatcttgaacgccaggcgcggcaacggggttatcaactgctg

attgcctgctcagaagatcagccagacaacgaaatgcgttgcattgagcaccttttacag

cgtcaggttgatgccattattgtttcgacgtcattgccacctgagcatcctttttatcaa

cgctgggctaacgacccgttcccgattgtcgcgctggaccgcgccctcgatcgtgaacac

tttaccagcgtggttggtgccgatcaggatgatgccgaaatgctggcggaagagttacgt

aagtttcccgccgagacggtgctttatcttggcgcgctgccggagctttctgtcagcttc

ctgcgtgaacaaggtttccgtactgcctggaaagatgatccgcgcgaagtgcatttcctg

tatgccaacagctatgagcgggaggcggctgcccagttattcgaaaaatggctggaaacg

catccgatgccgcaggcgctgttcacaacgtcgtttgcgttgttgcaaggagtaatggat

gtcacgctgcgtcgcgacggcaaactgccttctgacctggcaattgccacctttggcgat

aatgaactgctcgacttcttacagtgcccggtgctggcagtggctcaacgtcaccgcgat

gtcgcagagcgtgtgctggagattgtcctggcaagcctggacgaaccgcgtaagccaaaa

cctggtttaacgcgcattaaacgtaatctctatcgccgcggtgtgctcaaccgtagctaa

atgatcagcagagtgacagaagctctaagcaaagttaaaggatcgatgggaagccacgag

cgccatgcattgcctggtgttattggtgacgatcttttgcgatttgggaagctgccactc

tgcctgttcatttgcattattttgacggcggtgaccgtggtaaccacggcgcaccatacc

cgtttactgaccgctcagcgcgaacaactggtgctggagcgagatgctttagacattgaa

tggcgcaacctgatccttgaagagaatgcgctcggcgaccatagccgggtggaaaggatc

gccacggaaaagctgcaaatgcagcatgttgatccgtcacaagaaaatatcgtagtgcaa

aaataaatgagtggtcaaggaaagcgattaatggtgatggcaggcggaaccggtggacat

gtattcccgggactggcggttgcgcaccatctaatggctcagggttggcaagttcgctgg

ctggggactgccgaccgtatggaagcggacttagtgccaaaacatggcatcgaaattgat

ttcattcgtatctctggtctgcgtggaaaaggtataaaagcactgatagctgcgccgctg

cgtatcttcaatgcctggcgtcaggcgcgggcgattatgaaagcgtacaaacctgacgtg

gtgctcggtatgggcggctacgtatcaggtcctggtggtctggccgcgtggtcgttaggc

attccggttgtacttcatgaacaaaacggtattgcgggcttaaccaataaatggctggcg

aagattgccaccaaagtgatgcaggcgtttccaggtgctttccccaatgcggaagtggtg

ggtaacccggtgcgtaccgatgtgttggcgctaccgttgccgcagcaacgtttggctgga

cgtgaaggtccggttcgtgtgctggtagtgggtggttcccagggcgcacgcattcttaac

cagacaatgccgcaggttgctgcgaaactgggtgattcagtcactatctggcatcagagc

ggcaaaggttcgcaacaatccgttgaacaggcgtatgccgaagcggggcaaccgcagcat

aaagtgactgaatttattgatgatatggcggcggcgtatgcgtgggcggatgtcgtcgtt

tgccgctccggtgctttaacggtgagtgaaatcgccgcggcaggactaccggcgttgttt

gtgccgtttcaacataaagaccgccagcaatactggaatgcgctaccgctggaaaaagcg

ggcgcagccaaaattatcgagcagccacagcttagcgtggatgctgtcgccaacaccctg

gccgggtggtcgcgagaaaccttattaaccatggcagaacgcgcccgcgctgcatccatt

ccggatgccaccgagcgagtggcaaatgaagtgagccgggctgcccgggcgtaaatgtcg

catgctgctctgaacacgcgaaacagcgaagaagaggtttcttctcgccgcaataatgga

acgcgtctggcggggatccttttcctgctgaccgttttaacgacagtgttggtgagcggc

tgggtcgtgttgggctggatggaagatgcgcaacgcctgccgctctcaaagctggtgttg

accggtgaacgccattacacgcgtaatgacgatatccggcagtcgatcctggcattgggt

gagccgggtacctttatgacccaggatgtcaacatcatccagacgcaaatagaacaacgc

ctgccgtggattaagcaggtgagcgtcagaaagcagtggcctgatgaattgaagattcat

ctggttgaatatgtgccgattgcgcggtggaatgatcaacacatggtagacgcggaagga

aataccttcagcgtgccgccagatcgcaccagcaagcaggtgcttccaatgctgtatggc

ccggaaggcagcgccaatgaagtgttgcagggctatcgcgaaatggggcagatgctggca

aaggacagatttactctgaaggaagcggcgatgaccgcgcggcgttcctggcagttgacg

ctgaataacgatattaagctcaatcttggccggggcgatacgatgaaacgtttggctcgc

tttgtagaactttatccggttttacagcagcaggcgcaaaccgatggcaaacggattagc

tacgttgatttgcgttatgactctggagcggcagtaggctgggcgcccttgccgccagag

gaatctactcagcaacaaaatcaggcacaggcagaacaacaatgaatgatcaaacaaagg

acacttaaacgtatcgttcaggcgacgggtgtcggtttacataccggcaagaaagtcacc

ctgacgttacgccctgcgccggccaacaccggggtcatctatcgtcgcaccgacttgaat

ccaccggtagatttcccggccgatgccaaatctgtgcgtgataccatgctctgtacgtgt

ctggtcaacgagcatgatgtacggatttcaaccgtagagcacctcaatgctgctctcgcg

ggcctgggcatcgataatattgttatcgaagttaacgcgccggaaatcccgatcatggac

ggcagcgccgctccgtttgtatacctgctgcttgacgccggtatcgacgagttgaactgc

gccaagaaatttgttcgcatcaaagagactgttcgtgtcgaagatggcgataagtgggct

gaatttaagccgtacaatggtttttcgctggatttcaccatcgattttaaccatccggct

attgattccagcaaccagcgctatgcgatgaacttctccgctgatgcgtttatgcgccag

atcagccgtgcgcgtacgttcggtttcatgcgtgatatcgaatatctgcagtcccgtggt

ttgtgcctgggcggcagcttcgattgtgccatcgttgttgacgattatcgcgtactgaac

gaagacggcctgcgttttgaagacgaatttgtgcgtcacaaaatgcttgatgcgatcggt

gacttgttcatgtgtggtcacaatattattggtgcatttaccgcttataaatccggtcat

gcactgaataacaaactgctgcaggctgtcctggcgaaacaggaagcctgggaatatgtg

accttccaggacgacgcagaactgccgttggccttcaaagcgccttcagccgtactggca

taaatgcagacccaggtcctttttgaacatccactaaatgaaaaaatgcgtacatggctg

cgcattgagtttttgactcagcaactcaccgttaatttacccatcgttgaccacactggc

gcgctgcatttcttccgtaatgtcagtgaattactggatgttttcgagcgcggcgaagtc

cgcactgagctgttgaaagaacttgaccggcagcaacgtaaactccagacctggattggc

gtgcctggcgtggaccagagccgtattgaagcattaattcagcagttaaaagcggcgggg

agcgtattaatttccgcgccgcgtatcgggcaatttctgcgtgaagatcgtttgattgct

ctggtgcgtcagcgactgagcatcccaggcggctgttgcagctttgatttacctacattg

cacatttggctgcatctaccccaggcgcagcgcgacagccaggtagaaacctggattgcc

agcctgaacccgctcacccaggcgcttaccatggtgctggatttaattcgccagtcggcc

cccttccgtaaacaaaccagcctgaatggtttttatcaggataacggtggcgatgccgac

ttgctgcgcctgaatctgtcgctcgattcacagctttatccgcaaatttccggtcataag

agccgttttgccattcgttttatgccgctggacagtgaaaacggacaggtaccggaacgt

ctggatttcgaactggcctgttgctaaatgaggtatatagttgccttaacgggaggcatt

ggcagtggcaagagtaccgttgccaatgcgtttgctgatctcggaattaacgtcattgat

gccgatattattgcgcgtcaggtggttgaaccaggtgcacctgcgctacatgccattgct

gatcactttggcgctaacatgattgctgctgatggaacattgcagcgccgggccttgcgc

gagcggatcttcgccaacccggaagagaaaaactggcttaacgacctgctgcatccgctg

attcagcaagagacgcaacaccagatccagcaagcaacctccccctatgtactgtgggtt

gtgccattgctggtagaaaactcactgtataaaaaagcgaatcgagtgctggtggtggat

gtcagcccagaaacgcaacttaagcgcaccatgcagcgcgatgatgtaactcgcgagcat

gtcgaacaaatccttgctgctcaggcaacgcgcgaagcccgccttgccgtggcagatgac

gtcattgataataacggcgcaccggatgctatcgcatcggatgttgcccgcctgcacgca

cactatttgcagcttgcgtcgcagtttgtctcacaggaaaaaccgtaaatgttgttagaa

caggggtggctggttggcgcgcgccgcgttccctcaccacattacgattgccgcccggat

gacgaaacacccaccctgctggtggtgcacaatattagcctaccgccaggcgagtttggc

ggtccgtggatcgacgcattattcactggaactattgatccgcaggcacatcctttcttt

gctgagatcgcccatttgcgcgtctccgctcactgtttgattcgccgtgatggtgaaata

gtccagtatgttcctttcgataaacgtgcatggcatgcgggagtctctcagtatcagggg

cgcgaacgctgcaatgatttttctattgggattgagcttgaaggcaccgatacgctggcg

tataccgatgcgcagtatcaacagcttgcggcggttacgcgggcactgattgattgctat

ccggatatcgctaataacatgacgggccattgtgatattgcgccggatcggaaaaccgat

cccggtcctgcatttgattgggcacggtttcgtgcgctggtcagcaaggagacaacatga

atgacgctatttacaaccttactggtgttaattttcgagcgcctgtttaagttgggcgag

cactggcagcttgatcatcgtcttgaagcgttctttcggcgggtgaaacatttttctctc

gggcgcacgttaggcatgaccattattgcgatgggcgtgacttttttactgttacgcgca

ttgcagggagtattgttcaacgttcccacgctgctggtgtggctgctgattggtttgctg

tgtattggcgcaggtaaagttcgtcttcattatcatgcttatctgacagctgcttcacgt

aatgatagccatgcccgtgccacgatggctggcgaactcaccatgattcacggcgtcccg

gcaggctgcgacgaacgtgagtatttgcgtgagctgcaaaatgcattgctgtggattaac

tttcgtttttatcttgcaccgctgttctggctgattgtggggggaacctggggacccgtt

acgctgatggggtatgcgttcttgcgtgcatggcaatactggctggcacgatatcagacg

ccgcatcatcgtttacagtccggcattgatgccgtgcttcatgtactggattgggtgccg

gttcgtcttgcgggtgtggtatatgccttgatcggtcatggtgagaaagcgttaccggcc

tggtttgcttcgctgggtgatttccatacttcgcagtatcaggtgttaacgcgtctggcg

cagttctctctggcgcgtgaaccgcatgtcgataaggtggagacgccgaaggcagcggtt

tcaatggcgaagaaaacctcgttcgtggtcgtggtggtgattgcgctactgacgatttac

ggggcgttggtgtaaatggccgaaaaaaaacagtggcatgaaacgctacacgaccagttt

gggcagtactttgcggtagataacgttctgtatcatgaaaagaccgatcaccaggatctg

atcatttttgagaacgctgcatttggtcgcgtaatggcgctggatggcgtagtacaaacc

accgagcgcgacgagtttatctatcatgagatgatgacccatgttccgctactggcccac

ggtcacgcgaaacatgtgctgattatcggcggcggcgacggtgccatgctgcgtgaagta

acccgacataaaaacgttgagtcaatcacgatggtggaaatcgatgcgggtgtcgtgtcg

ttctgccgtcagtatctacccaaccataacgccggtagctacgacgatccgcgctttaag

ctggtgatcgacgatggcgtcaatttcgttaatcaaaccagccagacctttgatgtcatt

atctccgactgcaccgatcctatcggtcccggcgaaagccttttcacttcggcattttat

gaaggctgcaaacgttgcctgaatcctggcggtatcttcgtcgcacaaaacggcgtctgc

tttttacagcaggaagaagccatcgacagccatcgcaaactcagccattacttcagcgac

gttggcttttatcaggcggcgatcccgacctattacggcggtatcatgacttttgcatgg

gcgacagataacgacgccttacgccatctctcaaccgaaattattcaggcgcgttttctc

gcctctggcctgaaatgccgttattacaatccggcagtccatacggcagcttttgcctta

ccccagtatctgcaagacgcactggcttcacagccgtcctaaatgaaagacatagataca

ctcatcagcaacaatgcactatggtcaaaaatgctggtggaagaggatcccgggtttttt

gagaaactggcacaagcgcaaaaaccgcgctttctatggattggatgttccgacagtcgc

gttcctgcagaacgtttaaccggtcttgagccgggcgaactctttgttcaccgtaatgtt

gctaacctggtcattcacaccgacctgaactgcctttccgtggttcagtatgcagtggat

gtactcgaagttgaacacattattatctgtggccactacggttgcggcggcgtacaagcc

gcagttgaaaacccggaactggggcttatcaacaactggctgctgcatatccgcgatatc

tggttcaaacatagctcattgctcggcgaaatgccgcaagagcgccgtctggataccttg

tgtgaactgaacgtcatggaacaggtgtataacctgggccactccaccattatgcaatca

gcgtggaaacgcgggcagaaagttaccattcacggctgggcctatggcattcacgacggc

ttgctgcgtgatctggatgttactgccaccaaccgcgaaacccttgagcaacgttaccgt

catgggatttccaacctcaagctgaaacacgccaaccacaaataaatgatgcatctttac

tgggtggcgctaaaaagcatctgggcgaaagagatccatcgctttatgcgtatctgggtg

cagacgctggtgccgccagttatcaccatgaccctttactttattattttcggtaacctg

attggttcgcgtattggcgatatgcatggcttcagctacatgcagttcatcgtaccgggg

ctgatcatgatgtcggtgatcaccaacgcctacgccaacgtcgcgtcatcattttttggt

gccaaattccagcgtaatattgaagagctgctggtagcgccggttccgactcacgtcatt

attgccggatatgtcggcggtggcgtggcgcgtggtctgtttgttggcattctggtgacg

gcaatttcactgttttttgtgccgtttcaggtgcattcgtgggtattcgttgccttaacc

ctggtgctcacggcggtgttgttctcccttgcgggtttgctgaacggcgtgtttgccaaa

acgttcgatgacatcagcctggtgccaacctttgtgttaacgccactcacgtatttgggt

ggggtcttttactcactgactttgttgccgccgttctggcaagggctgtcgcacctgaac

ccaatcgtttatatgatcagtggtttccgctacggcttcctcggtatcaatgatgttccg

ctggtcactacctttggcgtactggtggtctttattgtggcgttttatttgatctgttgg

tcgctgatccaacgtggacgtggtttgcgtagctaaatgattcgcacgatgctgcagggc

aaactccaccgcgtgaaagtgactcatgcggacctgcactatgaaggttcttgcgccatt

gaccaggattttcttgacgcagccggtattctcgaaaacgaagccattgatatctggaat

gtcaccaacggcaagcgtttctccacttatgccatcgcggcagaacgcggttcgagaatt

atttctgttaacggtgcggcggcccactgcgccagtgtcggcgatattgtcatcatcgcc

agcttcgttaccatgccagatgaagaagctcgcacctggcgacccaacgtcgcctatttt

gaaggcgacaatgaaatgaaacgtaccgcgaaagcgattccggtacaggttgcttgaatg

gaattctctccccctctacagcgcgcgacgctaattcagcgttacaaacgttttttagcc

gatgtgatcacacccgatggtcgcgaattaacgctacactgcccgaatacgggtgcgatg

accggttgtgcaacgcctggcgataccgtctggtattcgacttcagacaacaccaaacgg

aaatacccacacacctgggaattaactcaaagccagagcggcgcatttatttgcgtcaac

acgctttgggctaacaggttgacgaaagaggctatccttaatgaatcaatttcagaactg

tcaggctatagctcgctgaaaagcgaagtaaaatacggcgcagaacgcagccgtattgac

tttatgttgcaggcggattcgcgtccagactgctatattgaagtgaaatcggttacgtta

gcggagaacgaacagggatattttcccgatgcggtcactgaacgaggtcagaaacacctt

cgggagttgatgagcgtagcggctgaaggccagcgtgcggttatctttttcgccgtgctg

cattcagccattacacggttttcacccgcgcgccacatcgatgagaaatacgcgcaacta

ttgtcagaagctcaacagaggggggtagaaattctggcttacaaagcggaaatttctgct

gaaggcatggctcttaaaaaatcactgccggttacattgtagatgctcgtctattggctg

gatatagtcggcacagcggtatttgccatctccggcgttttgttagccggaaaattgcgt

atggacccttttggtgttctggtactgggcgtggttaccgctgtaggcggcgggacaatt

cgcgacatggcgctggatcacggcccggtattttgggtgaaagatcccaccgatctggtc

gttgcaatggtaaccagcatgctgaccatcgtgctggtgcgccagccaagacgcttacca

aaatggatgttgccggtgctggacgccgttggtctggcggtgtttgtcggcattggcgtg

aataaagcctttaatgcggaagccggtccgttaatcgcggtttgtatgggcgtcattact

ggcgttggcggcgggatcattcgtgatgttctggcccgcgaaatccccatgattttacgt

acagaaatctacgcaactgcctgtattatcggcggtattgtccacgctacggcttattac

acattttccgtaccactggaaacagccagtatgatgggcatggtcgtgacgctattgatt

cggctggcggctattcgttggcatcttaagctaccgacgtttgcgctggatgagaatggg

cgttgaatgaaaatcggcatcattggtgcaatggaagaagaagttacgctgctgcgtgac

aaaatcgaaaaccgtcaaactatcagtctcggcggttgcgaaatctataccggccaactg

aatggaaccgaggttgcgcttctgaaatcgggcatcggtaaagtcgctgcggcgctgggt

gccactttgctgttggaacactgcaagccagatgtgattattaacaccggttctgccggt

ggcctggcaccaacgctgaaagtgggcgatatcgttgtctcggacgaagcacgttatcac

gacgcggatgtcacggcatttggttatgaatacggtcagttaccaggctgtccggcaggc

tttaaagctgacgataaactgatcgctgccgctgaggcctgcattgccgaactgaatctt

aacgctgtacgtggcctgattgttagcggcgacgctttcatcaacggttctgttggtctg

gcgaaaatccgccacaatttcccacaggccattgctgtagagatggaagcgacggcaatc

gcccatgtctgccacaatttcaacgtcccgtttgtcgtagtacgcgccatctccgacgtg

gccgatcaacagtctcatcttagcttcgatgagttcctggctgttgccgctaaacagtcc

agcctgatggttgagtcactggtgcagaaactggcacatggctaaatgtacgacaatctg

aaaagtctgggtattaccaatcctgaagaaattgatcgttacagcctccggcaggaagcc

aacaacgatattctgaaaatctatttccagaaagacaaaggcgagtttttcgccaagagc

gttaagtttaaatatccgcgtcagcgtaaaacggtcgtcgctgatggtgtgggtcagggt

tataaagaagtccaggaaatcagcccgaatctacggtatatcattgatgagcttgatcaa

atctgccagcgtgaccgcagcgaagttgatcttaagcgtaagatcctcgacgacttacgt

cacctggagtcagtcgtaaccaataagatcagcgagattgaagccgatctggaaaaacta

acgcgtaaataaatgcagcagttacagaacattattgaaaccgcttttgaacgccgtgcc

gagatcacgccagccaatgcagacaccgttacccgcgaagcggtaaatcaggtgatcgcc

ctgctggattccggcgcactgcgtgtagcggaaaaaattgacggtcagtgggtgacgcat

cagtggttgaaaaaagcggtactgctctctttccgtattaatgataatcaggtgatcgaa

ggggcagaaagccgctacttcgataaagtgccgatgaaattcgctgattacgacgaagca

cgtttccagaaagaaggcttccgtgttgtgccaccagcggcggtacgtcagggcgcgttc

attgcccgtaacaccgtgctgatgccgtcttacgtcaacatcggcgcatatgttgatgaa

ggcaccatggttgatacctgggcgaccgtcggttcctgtgcgcagattggtaaaaacgtc

cacctttccggtggcgtgggtatcggcggcgtgctggagccgctgcaggctaacccgacc

attattgaagataactgcttcatcggcgcgcgctctgaagtggttgaaggggtgattgtc

gaagaaggttccgtcatttccatgggcgtatacattggtcagagcacccgtatttacgac

cgtgaaaccggcgaaatccactacggtcgcgttccggcggggtctgtggttgtttcaggt

aatctgccgtcgaaagatggcaaatacagcctctactgtgcggttatcgttaagaaagtt

gacgcgaaaactcgcggcaaagtcggcattaacgaactgctgcgtaccatcgactaaatg

gctaccaatgcaaaacccgtctataaacgcattctgcttaagttgagtggcgaagctctg

cagggcactgaaggcttcggtattgatgcaagcatactggatcgcatggctcaggaaatc

aaagaactggttgaactgggtattcaggttggtgtggtgattggtgggggtaacctgttc

cgtggcgctggtctggcgaaagcgggtatgaaccgcgttgtgggcgaccacatggggatg

ctggcgaccgtaatgaacggcctggcaatgcgtgatgcactgcaccgcgcctatgtgaac

gctcgcctgatgtccgctattccattgaatggcgtgtgcgacagctacagctgggcagaa

gctatcagcctgttgcgcaacaaccgtgtggtgatcctctccgccggtacaggtaacccg

ttctttaccaccgactcagcagcttgcctgcgtggtatcgaaattgaagccgatgtggtg

ctgaaagcaaccaaagttgacggcgtgtttaccgctgatccggcgaaagatccaaccgca

accatgtacgagcaactgacttacagcgaagtgctggaaaaagagctgaaagtcatggac

ctggcggccttcacgctggctcgtgaccataaattaccgattcgtgttttcaacatgaac

aaaccgggtgcgctgcgccgtgtggtaatgggtgaaaaagaagggactttaatcacggaa

taagtgattgataaatccgcctttgtgcatccaaccgccattgtggaagagggcgcgtcg

attggcgcgaacgcacacattggtcctttttgtatcgttggaccccatgtcgaaattggt

gagggtaccgtactgaaatctcacgttgtcgtgaatggtcatactaaaattggccgcgat

aatgagatttatcagttcgcctccatcggcgaagttaaccaggatttgaaatatgctggc

gaaccgacccgtgtggaaatcggcgatcgtaaccgcattcgcgaaagcgtcaccattcat

cgtggcacagtccagggcggtggattgacgaaggtgggcagcgacaacttactgatgatc

aacgcgcacattgcgcacgattgtacggtaggtaaccgctgtattctcgccaacaacgca

acgctggcgggtcacgtatcggttgacgacttcgcgatcatcggcggcatgaccgcagtc

catcagttctgcatcattggtgcgcatgtgatggttggcggctgctccggtgtggcgcag

gacgtccctccttatgtcattgcgcagggtaaccacgccacgccgttcggtgtcaatatc

gaagggctgaagcgccgcggattcagccgtgaggcgattaccgctatccgcaatgcgtat

aagctgatttatcgtagcggtaaaacgctcgatgaagtgaaaccggaaattgctgaactg

gcggaaacatatccggaagtgaaagcctttaccgatttctttgcacgctcaacgcgcggt

ctgattcgttaaatgactgaacagcgtccattaacgattgccctggtcgccggagaaacc

tccggcgatatcctgggggccggtttaatccgtgctctgaaagaacgtgtgcccaacgcc

cgctttgttggtgttgccgggccacgaatgcaggctgaaggctgcgaagcctggtacgaa

atggaagaactggcggtgatgggcattgttgaagtgctcggtcgtctgcgtcgcttactg

catattcgtgccgatctgacaaagcgttttggtgaactaaagccagatgtttttgttggc

attgatgcgcctgacttcaatattaccctcgaaggtaaccttaaaaagcagggtatcaaa

accattcattatgtcagtccgtccgtctgggcgtggcgacagaaacgcgttttcaaaata

ggcagagccaccgatctggtgctcgcatttctgcctttcgaaaaagcgttttatgacaaa

tacaacgtaccgtgccgctttatcggtcataccatggctgatgccatgccattagatcca

gataaaaatgccgcccgtgatgtgctggggatcccgcacaatacccactgtctggcattg

ttgccgggaagtcgtggcgcggaagttgagatgcttagcgccgatttcctgaaaacggcc

cagcttttgcgccagacgtaccctgatctggagatcgtggtgccgctggtgaatgccaaa

cgccgcgagcagtttgaacgcatcaaagctgaagtcgcgccagacctttcagttcatttg

ctggatgggatgggccgtgaggcgatggtcgccagcgatgcggcactactggcatcgggg

acggcagccctggagtgtatgctggcgaaatgcccgatggtggtgggatatcgcatgaag

ccttttaccttctggttggcgaagcggctggtgaaaactgattatgtctcgctgccaaat

ctgctggcgggcagagagttagtcaaagagttattgcaggaagagtgtgagccgcaaaaa

ctggctgaggcgctgttaccgctgctggcgaacgggaaaaccagccacgcgatgcacgat

accttccgtgaactgcatcagcagatccgctgcaatgccgatgagcaggcggcacaagcc

gttctggagttagcacaatgaatgcgtgctttaccgatctgtttagtagcactcatgcta

agcggctgttccatgttaagcagatcccctgtcgaacccgttcaaagcactgcaccccag

ccgaaagcggagcctgcaaaaccgaaagcgccgcgcgccacgccggtccgaatttatacc

aatgcagaagaattagtcggcaaaccgttccgcgatctcggtgaagtcagtggcgactct

tgccaggcctctaatcaggactctccgccgagcattccaaccgcacgtaagcggatgcaa

atcaacgcctctaaaatgaaagccaatgctgtattactgcatagctgcgaagtcaccagc

ggtacgccaggctgctatcgtcaggctgtatgtatcggttctgcgcttaacattacggcg

aaatgaatgataaaactttcgaatatcaccaaagtgttccaccagggcacccgcaccatc

caggcgttgaacaacgtcagcctgcatgtgccagctgggcaaatttatggcgttatcggt

gcctcaggcgcgggtaagagtacgcttatacgttgtgtaaacctgctggagcgcccaacc

gagggtagtgtgctggtcgatggccaggaactgaccacgctgtcagaatccgagttgacc

aaagctcgccgccagattggtatgattttccagcattttaacctgctctcttcgcgtact

gtttttggcaacgtggctctgccgctggagctggacaacacaccgaaagacgagatcaaa

cgtcgcgtgacggaattgctgtcattagttggtcttggcgataagcatgatagctacccg

tcgaatctttccggtgggcagaaacaacgtgtggcgattgcccgtgcattagccagcaat

cccaaagtattgctgtgtgatgaagccaccagcgcgctggacccggcaacgacacgttct

attctcgaactgctgaaagacatcaaccgccgtctgggtttgacgattctgttgatcact

cacgaaatggacgttgtgaagcgcatttgtgattgcgtggcggtcatcagcaatggcgaa

ctgatcgagcaggacacggtaagtgaagtgttctcgcatccgaaaacgccgctggcgcag

aagtttattcagtcaaccctgcatctggatatcccggaagattaccaggaacgtctgcaa

gcggagccattcactgactgcgtcccgatgctgcgtctggagtttaccggtcaatcggtc

gatgccccactgctttctgaaaccgcgcgtcgtttcaacgtcaacaacaacattattagc

gcgcagatggattacgccggtggcgtgaagttcggcatcatgctgactgaaatgcacggc

acacaacaagatacgcaagccgccattgcctggctgcaggaacaccatgtaaaagtagag

gtactgggttatgtctgaatgaaagccacgtcggaagaactcgccatttttgtttcggtc

gtagaaagcggcagctttagccgggcagcggaacaattagggcaagcaaactcagcggta

agccgggcggtgaaaaagctggagatgaaacttggcgttagcctgcttaatcggaccacg

cgacaacttagcctgacggaagaaggcgagcgttatttccgtcgcgtacagtcaattttg

caggagatggcagcggcagaatcagaaattatggagacgcgtaatacaccgcgtggactg

ttacggatcgatgccgcaactccagtggtgctgcactttctgatgccgttaattaagcct

ttccgtgaacgctatccggaagtcactttgtcgctagtctcctccgaaacgattattaat

ttgatcgaaagaaaagtggatgtcgcgatacgcgctggtacgttaacggattccagctta

cgtgccaggccgttatttaacagttatcgaaaaattatcgcctcccccgattatatttcc

cgctacgggaagccagaaacgatcgacgatttaaagcaacatgtttgcctgggattcact

gaacccgcttccctcaatacctggccgatagcctgtagcgatggacaattacatgaggtg

aagtacggtttgtcatccaatagtggggaaacactgaaacagctttgcctgagtgggaac

gggattgcgtgtttgtccgactatatgatcgacagagaaatcgctcgcggagaattggtg

gagttaatggcagataaagtgttgccagtggaaatgccattcagtgcagtctattacagc

gaccgtgcggtaagtacgcgcatccgggcttttatcgatttccttagcgagcatgtaaaa

acagctcccggaggagctgtcagagaggcttaaatgggcaggataagctcgggaggaatg

atgtttaaggcaataacgacagtcgccgcactggtcatcgccaccagtgcaatggcgcag

gatgatttaaccattagcagccttgcaaagggcgaaaccaccaaagctgcatttaatcag

atggtgcaagggcataagctgcctgcctgggtgatgaaaggcggtacttatacccctgca

caaaccgtgacattgggagatgagacgtatcaggtgatgagcgcgtgcaaaccgcatgac

tgtggctcgcaacgtatcgctgtgatgtggtccgagaaatctaatcagatgacggggctg

ttctcgactattgatgagaaaacgtcgcaagagaaactcacctggttgaatgtgaacgat

gcgctttcgattgatggtaaaacggtgctgttcgcggcgttgaccggcagcctggaaaac

catccggatggctttaattttaaataaatgagcgaaaaatacatcgtcacctgggacatg

ttgcagatccatgcacgtaaactcgcaagccgactgatgccttctgaacaatggaaaggc

attattgccgtaagccgtggcggtctggtaccgggtgcgttactggcgcgtgaactgggt

attcgtcatgtcgataccgtttgtatttccagctacgatcacgacaaccagcgcgagctt

aaagtgctgaaacgcgcagaaggcgatggcgaaggcttcatcgttattgatgacctggtg

gataccggtggtactgcggttgcgattcgtgaaatgtatccaaaagcgcactttgtcacc

atctttgcaaaaccggctggtcgtccgctggttgatgattatgttgttgatatcccgcaa

gatacctggatcgaacagccgtgggatatgggcgtcgtattcgtcccgccaatctccggt

cgctaaatgacgttaccgagtggacacccgaaaagcagattgatcaaaaaatttaccgca

ctaggcccatatattcgtgaaggtaagtgcgaagataatcgattctttttcgattgtctg

gctgtatgcgtcaacgtgaaaccggcaccggaagtgcgtgagttctggggctggtggatg

gagcttgaagcacaggaatcccgttttacatacagttaccagtttggtctgttcgataaa

gcaggcgactggaagagtgttccggtaaaagacactgaagtggttgaacgactggagcac

accctgcgtgagttccacgagaagctgcgtgagctgctgacgacgctgaatctgaagctg

gaaccggcggatgattttcgtgatgaaccggtgaagttaacggcgtgaatggctgatttc

accctgtcaaaatcgctgtttagcggaaaatatcgcaatgcctcttcaacgcctggcaac

attgcctatgcgttgtttgtgctgttttgcttttgggctggggcgcaattgctgaacctg

ttagtgcatgcgcccggcgtctatgagcgtttaatgcaggtccaggaaacaggtcgccca

cgggtggaaattggtttaggtgtcggcaccattttcgggctgatcccgtttttagtaggc

tgcctcatttttgcagtggtggcgctatggctgcactggcgacatcgccgccagtaaatg

acacaacctctttttctgatcgggcctcggggctgtggtaaaacaacggtcggaatggcc

cttgccgattcgcttaaccgtcggtttgtcgataccgatcagtggttgcaatcacagctc

aatatgacggtcgcggagatcgtcgaaagggaagagtgggcgggatttcgcgccagagaa

acggcggcgctggaagcggtaactgcgccatccaccgttatcgctacaggcggcggcatt

attctgacggaatttaatcgtcacttcatgcaaaataacgggatcgtggtttatttgtgt

gcgccagtatcagtcctggttaaccgactgcaagctgcgccggaagaagatttacggcca

accttaacgggaaaaccgctgagcgaagaagttcaggaagtgctggaagaacgcgatgcg

ctatatcgcgaagttgcgcatattatcatcgacgcaacaaacgaacccagccaggtgatt

tctgaaattcgcagcgccctggcacagacgatcaattgttgaatgacccatcaattaaga

tcgcgcgatatcatcgctctgggctttatgacatttgcgttgttcgtcggcgcaggtaac

attatttttcctccaatggttggcttacaggcaggcgaacacgtctggactgcggcattc

ggcttcctcattactgccgttggtctgccggtattaacggtagtggcgctggcaaaagtt

ggcggcggtgttgacagcctcagcacgccaatcggtaaagtcgctggcgtactgctggca

acggtttgttacctggcggtggggccgcttttcgctacgccgcgtacagctaccgtttct

tttgaagtggggattgcgccgctgacgggtgattccgcgctgccgctgtttatctacagc

ctggtctatttcgctatcgttattctggtttctctctatccgggcaagctgctggatacc

gtgggcaacttccttgcgccgctgaaaattatcgcgctggtcatcctgtctgttgccgcg

attgtctggccggcgggttctatcagcacggcgactgaggcttatcaaaacgctgcgttt

tctaacggcttcgttaacggctatctgaccatggatacgctgggcgcaatggtgtttggt

atcgttattgttaacgcggcgcgttctcgtggcgttaccgaagcgcgtctgctgacccgt

tataccgtctgggctggcctgatggcgggtgttggtctgactctgctgtacctggcgctg

ttccgtctgggttcagacagcgcgtcgctggtcgatcagtctgcaaacggcgctgctatt

ctgcatgcttacgttcagcacacctttggcggcggcggtagcttcctgctggcggcgtta

atcttcatcgcctgcctggtaacggcagttggcctgacctgtgcttgtgcagaattcttt

gcccagtacgtaccgctctcttatcgtacgctggtgtttatcctcggcggcttctcgatg

gtggtttctaacctcggcttaagccagctgattcagatctccgtaccggtgctgaccgct

atttatccgccgtgtatcgcactggttgtattaagttttacacgctcatggtggcataat

tcgtcccgcgtgattgctccgccgatgtttatcagcctgctttttggtattctcgacggg

atcaaagcatctgcattcagcgatatcttaccgtcctgggcgcagcgtttaccgctggcc

gaacaaggtctggcgtggttaatgccaacagtggtgatggtggttctggccattatctgg

gatcgcgcggcaggtcgtcaggtgacctccagcgctcactaaatgaaatttgaactggac

accaccgacggtcgcgcacgccgtggccgcctggtctttgatcgtggcgtagtggaaacg

ccttgttttatgcctgttggcacctacggcaccgtaaaagggatgacgccggaagaagtt

gaagccactggcgcgcaaattatcctcggcaacaccttccacctgtggctgcgcccgggt

caggaaatcatgaaactgcacggcgatctgcacgattttatgcagtggaagggaccgatc

ctcaccgactccggcggcttccaggtcttcagccttggtgatattcgtaaaatcaccgaa

cagggcgttcacttccgtaacccgatcaacggcgacccgattttcctcgacccggaaaag

tcgatggagattcagtacgatcttggttcggatatcgtcatgatctttgatgagtgtacg

ccgtatcctgctgattgggattacgcaaaacgctccatggagatgtctctgcgttgggcg

aagcgtagccgtgagcgttttgacagtctcggaaacaaaaatgcgctgtttggtatcatt

cagggcagcgtttacgaagatttacgtgatatttctgttaaaggtctggtagatatcggt

tttgatggctacgctgtcggcggtctggctgtgggtgagccgaaagcagatatgcaccgc

attctggagcatgtatgcccgcaaattccggcagacaaaccgcgttacctgatgggcgtt

ggtaaaccagaagacctggttgaaggcgtacgtcgtggtatcgatatgtttgactgcgta

atgccaacccgcaacgcccgaaatggtcatttgttcgtgaccgatggcgtggtgaaaatc

cgcaatgcgaagtataagagcgatactggcccactcgatcctgagtgtgattgctacacc

tgtcgcaattattcacgcgcttacttgcatcatcttgaccgttgcaacgaaatattaggc

gcgcgactcaataccattcataaccttcgttactaccagcgtttgatggcgggtttacgc

aaggctattgaagagggtaaattagagagcttcgtaactgatttttaccagcgtcagggg

cgagaagtaccacctttgaacgttgattaaatgcattgcccattctgtttcgccgtggac

actaaggtaattgactctcgtctcgtgggcgagggttcatccgtacgccgccgtcggcag

tgtctggtgtgtaatgaacgtttcaccacctttgaagtggcggagctggttatgccgcgt

gttgtaaaaagcaacgacgtgcgtgaaccgtttaatgaagaaaaattacgtagcggaatg

ctgcgggcgctggaaaaacgtccggtgagttccgatgacgtcgaaatggcaatcaatcat

attaaatcgcagctgcgcgccaccggtgagcgcgaagtgccgagcaagatgattggcaat

ctggtgatggagcaattgaaaaagctcgataaagtcgcctatatccgttttgcctctgtc

taccgcagtttcgaagatatcaaagaatttggcgaagagatcgcgcgcctggaggactaa

atgtccagtcaatatttacgtatttttcaacagccgcgttcagccatattgctgatcctg

ggttttgcttccgggctaccgctcgccctgacatccggcaccttacaggcctggatgacg

gtcgagaatatcgatctcaaaaccattggtttcttctctctggtaggccaggcttacgtt

tttaaattcctctggtcaccgctgatggaccgctacacgcccccattttttgggcggcgg

cgcggttggctgctcgccacgcaaatcctgttattagtcgccattgcggcgatgggtttt

ctcgaaccaggcacccaactccgctggatggcggcgctggcggtggtgatcgctttttgc

tctgcctcccaggatattgtcttcgatgcgtggaaaaccgatgtgctcccggcagaagaa

cgtggtgcgggcgcggcaatcagcgtgctgggttaccgtttagggatgctggtttccggc

ggcctagccctgtggctggcagataaatggctgggctggcagggcatgtactggttgatg

gcggcgctgttgatcccctgtattatcgcgacgttgcttgcaccagaaccaaccgacacc

attcctgtgccaaaaacgctggaacaagcggttgttgcacctctgcgagatttctttggt

cgcaataatgcctggcttattttgcttcttatcgtgctgtataagctgggcgacgcattc

gccatgagcctgacaaccacgtttttgattcgcggcgtcgggtttgatgcgggtgaagta

ggcgtggttaacaaaacgcttggcttactagcgaccattgttggcgcattgtacggtggg

attttgatgcagcgcctgtcactgttccgggcactgctgattttcggcattttacaaggt

gcgtctaacgctggttactggctgctgtcgattactgataagcatctctacagcatgggc

gcagccgtctttttcgaaaacctctgtggcgggatgggcacatcagcctttgtcgcgctg

ttaatgacactatgtaataagtcattttccgctactcaatttgccctgctctcagcgctt

tctgctgtagggcgagtttatgtcggccccgtggcgggttggtttgttgaagcacacggc

tggtcgacattctatctattctccgtcgccgctgccgtaccagggcttattttgctgctg

gtttgccgccagacgcttgaatatacacgagtaaatgacaactttatctcccgtaccgca

tatccggcaggttatgcctttgccatgtggacactggcggcgggcgtcagcctgttggcc

gtgtggttactgctgttgacgatggacgcgctggatttgacgcacttctctttcctgcct

gctctgctggaagtcggggttttagtcgccctttctggcgtcgtgcttggtggtttgctg

gattatctggcgctacgaaaaacgcatctgacgtaaatgcaaacacaaatcaaagttcgt

ggatatcatctcgacgtttaccagcacgtaaacaacgctcgctaccttgaattcctcgaa

gaagcccgctgggatgggttggaaaatagcgacagttttcagtggatgacggcccataac

atcgctttcgtggtggtgaatatcaatattaactatcgtcgtccggcggtattaagcgac

ttgttaaccattacgagccagttacagcaattaaacggtaaaagcggcatcttaagccag

gtcattacactggagccggaagggcaggtggtagcggatgcgcttattacgtttgtttgt

attgatcttaaaacgcagaaagcattagctctggaaggggaattgcgcgaaaagctggag

cagatggttaagtaaatgcaacgaatcattttaatcatcattggctggctggcggtagtg

ctgggtacgctgggcgtggtattaccggtattaccgacgacgccgtttatcctgctggcg

gcctggtgctttgcccgttcttccccgcgctttcacgcctggttgctgtaccgctcatgg

tttggcagctatctacgtttctggcagaaacatcatgcgatgccgcgcggtgtcaaaccg

cgggcgattttgcttattttgctcacgtttgccatttctctgtggttcgtccagatgcca

tgggtgcgcatcatgttgctggtaattctcgcctgtttgcttttctatatgtggcgaatt

ccggtgattgatgaaaagcaagaaaagcactgaatgcaaaccagcccgctgttaacacag

cttatggaagcactgcgctgtctgccgggcgttggcccgaagtcggcgcagcgtatggcg

ttcacgctgcttcagcgcgatcgtagcggcgggatgcgtctggcgcaggcgctcacccgg

gcgatgtcggaaatcggccactgcgccgattgccgcaccttcaccgaacaggaagtctgt

aacatctgttcgaatccgcgtcgtcaggaaaacggtcaaatctgcgtggtggagagtccg

gcggacatttacgccattgagcagacggggcagttttcaggtcgttattttgtgttgatg

ggacatctgtcaccgctggacggcatcggtccggatgatatcggccttgatcgtctggaa

cagcgtctggcagaggaaaaaatcactgaagtgatcctcgccaccaaccctacggttgaa

ggtgaagctaccgctaactacattgccgagctttgcgcgcaatatgacgtggaagccagc

cgaatcgctcatggcgtaccggttggcggcgaactggaaatggtcgatggcaccacgctg

tcacactcccttgccgggcgtcataagattcgtttttaaatgcgtcagactaaaaccggt

atcctgctggcaaacctgggtacgcccgatgcccccacacctgaagcggtaaaacgctat

ctgaaacaatttttaagcgacagacgcgtggttgatacctcacggttgttatggtggcca

ttgctgcgcggcgtgattttgccgctgcgctcgccgcgtgtggcgaagctgtatgcctct

gtctggatggaaggtggctcgccgctgatggtttacagccgccagcaacagcaggcgctg

gcacaacgtttaccggagacgcccgtagcgctgggaatgagctacggctcgccatcactg

gaaagcgccgtagatgaactcctggcagagcatgtagatcatattgtggtgctgccgctt

tatccgcaattctcctgttctacggtcggtgcggtatgggatgaactggcacgcattctg

gcgcgcaaacgtagcattccggggatatcgtttatacgtgattacgccgataaccacgat

tacattaatgcactggcgaacagcgtacgcgcttcttttgccaaacatggcgaaccggat

ctgctgctgctctcttatcatggcattccccagcgttatgcagatgaaggcgatgattac

ccgcaacgttgccgcacaacgactcgtgaactggcttccgcattggggatggcaccggaa

aaagtgatgatgacctttcagtcgcgctttggtcgggaaccctggctgatgccttatacc

gacgaaacgctgaaaatgctcggagaaaaaggcgtaggtcatattcaggtgatgtgcccg

ggctttgctgcggattgtctggagacgctggaagagattgccgagcaaaaccgtgaggtc

ttcctcggtgccggcgggaaaaaatatgaatatattccggcgcttaatgccacgccggaa

catatcgaaatgatggctaatcttgttgccgcgtatcgctaaatgatctggaaacgccat

ttaacgctcgacgaactgaacgccaccagcgataacacaatggtggcgcatctgggaatt

gtgtatacccgtctgggcgatgatgtgctggaagccgaaatgccggttgatacccgtact

catcagccgtttggcctgctgcatggcggcgcgtcggcggcgctggctgaaacgctggga

tcgatggccggatttatgatgacccgtgacgggcagtgtgtggtgggcacggaacttaac

gccacccatcatcgcccggtgtctgaaggcaaggtacgcggcgtctgccagccgctgcat

cttgggcggcaaaatcagagctgggaaatcgtcgttttcgatgaacaggggcggcgttgc

tgcacttgtcggctgggtacggcagt----------atgtccttgattaacaccaaaatt

aaaccttttaaaaaccaggcattcaaaaacggcgaattcatcgaaatcaccgaaaaagat

accgaaggccgctggagcgtcttcttcttctacccggctgactttactttcgtatgcccg

accgaactgggtgacgttgctgaccactacgaagaactacagaaactgggcgtagacgta

tacgcagtatctaccgatactcacttcacccacaaagcatggcacagcagctctgaaacc

atcgctaaaatcaaatatgcgatgatcggcgacccgactggcgccctgacccgtaacttc

gacaacatgcgtgaagatgaaggtctggctgaccgtgcgaccttcgttgttgacccgcag

ggtatcatccaggcaatcgaagttaccgctgaaggcattggccgtgacgcgtcagacctg

ctgcgtaaaatcaaagcagcacagtacgtagcttctcacccaggtgaagtttgcccggct

aaatggaaagaaggtgaagcaactctggctccgtctctggacctggttggtaaaatctaa

gtgttacaacttcttttagcagtttttattggcggtggtacgggaagcgtggcgagatgg

ctgttaagtatgcgatttaacccgctgcatcaggcgattccgttggggacgctggctgca

aatctgattggggcattcatcataggaatgggattcgcctggttcagcaggatgacgaac

attgatccagtgtggaaagtattaatcaccaccggattttgtggcggtctaacaaccttc

tcaacattttcggcagaagtggtgtttttgttacaagagggccgctttggctgggcatta

ctgaacgttttcgtcaaccttctggggtcttttgccatgaccgcactggcattctggctg

ttttcggcctcaaccgcacactaaatgaacaaggttgctcaatattaccgtgaactggtt

gcgtcactgagcgaacgcctgcgcaatggcgaacgtgatatcgacgcactggtggaacag

gcgcgcgagcgcgtaataaaaacaggggagttaacgcgaaccgaggtcgatgagctgacg

cgagctgtcagacgtgacctggaagagttcgccatgagctatgaagagagcctgaaagaa

gaatctgacagcgtctttatgcgggtgattaaagaaagcttgtggcaggagctggcagac

atcaccgataaaacgcagcttgaatggcgcgaagttttccaggacctcaatcatcatggg

gtttatcacagcggagaagtggtcgggctgggaaatctggtctgcgagaaatgtcacttc

catctcccgatctacacaccggaagtgctgacgctatgcccgaaatgtggtcatgaccag

ttccagagacgcccgtttgagccgtaaatgagcgacgacaattcacacagtagtgacacg

ataagcaacaagaagggatttttctccctgttactcagccaacttttccacggtgaaccg

aaaaaccgtgacgaactgctggcgctgatccgtgattccgggcagaacgaccttatcgac

gaagatacgcgcgatatgctcgaaggggtgatggacatcgcagaccaacgcgtccgcgac

atcatgatcccccgctcccagatgattaccctgaaacgcaaccagacgctggacgaatgc

cttgatgtcatcatcgagtccgcccactcacgtttcccggtgattagcgaagacaaagat

cacattgaagggattctgatggcgaaagacttgctgccgtttatgcgcagcgatgctgaa

gccttcagcatggacaaagtgttacgtcaggcggttgtcgttcctgaaagtaagcgcgta

gaccggatgctgaaagagtttcgctctcagcgttaccacatggcgatcgttattgacgaa

ttcggtggggtttccggtctggtaaccattgaagacatcctggaactgattgttggtgag

attgaagacgaatatgacgaagaagatgatatcgacttccgtcagctgagtcgtcatacc

tggaccgtgcgcgcactggcttccattgaagacttcaacgaagcgttcggcacccacttt

agcgatgaagaagtcgacactatcggtggtctggtgatgcaggcatttgggcatcttccg

gcacgtggcgaaaccatcgacatcgacggttaccagttcaaagtggcgatggccgacagt

cggcgtattattcaggttcatgtcaaaatcccggatgactcaccccagccgaagctggat

gaataaatgagtcaggtgatcctcgatttacaactggcatgtgaagataattccgggtta

ccggaagagagccagtttcagacatggctgaatgcggtgatcccgcagtttcaggaagag

tcggaagtgacgattcgcgtggtcgataccgccgaaagccacagtctcaatctgacctat

cgcggtaaggataagccgaccaacgtgctctccttcccgtttgaagtgccgcccggcatg

gagatgtcgctactgggcgatctggttatctgccgtcaggtggttgagaaggaagcacag

gagcaaggcaaaccactggaggcgcactgggcgcatatggtggtgcacggcagtctgcat

ttgttaggttacgatcacatcgaagatgacgaagcagaagaaatggaagccctcgaaaca

gagattatgcttgctctgggctatgaggatccgtacattgccgagaaagaataaatgtat

gcattaacccagggccggatctttaccggccacgaatttcttgatgaccacgcggttgtt

atcgctgatggcctgattaaaagcgtctgtccggtagcggaactgccgccagagatcgaa

caacgttcactgaacggggccattctctcccccggttttatcgatgtgcagttaaacggc

tgcggcggcgtacagtttaacgacaccgctgaagcggtcagcgtggaaacgctggaaatc

atgcagaaagccaatgagaaatcaggctgtactaactatctgccgacgcttatcaccacc

agcgatgagctgatgaaacagggcgtgcgcgttatgcgcgagtacctggcaaaacatccg

aatcaggcgttaggtctgcatctggaaggtccgtggctgaatctggtaaaaaaaggcacc

cataatccgaattttgtgcgtaagcctgatgccgcgctggtcgatttcctgtgtgaaaac

gccgacgtcattaccaaagtgaccctggcaccggaaatggttcctgcggaagtcatcagc

aaactggcaaatgccgggattgtggtttctgccggtcactccaacgcgacgttgaaagaa

gcaaaagccggtttccgcgcggggattacctttgccacccatctgtacaacgcgatgccg

tatattaccggtcgtgaaccgggcctggcgggcgcgatcctcgacgaagctgacatttat

tgcggtattatcgctgatggcctgcatgttgattacgccaacattcgtaacgctaaacgc

cttaaaggcgacaaactgtgtctggttaccgacgccaccgcgccagcaggtgccaacatt

gaacagttcatttttgcgggtaaaacaatatactaccgtaacggactttgtgtggatgag

aacggtacgttaagcggttcatccttaaccatgattgaaggcgtgcgtaatctggtcgaa

cattgcggtatcgcactggatgaagtgctgcgtatggcgacgctctatccggcgcgtgcg

attggcgttgagaaacgtctcggcacactcgccgcaggtaaagtagccaacctgaccgca

ttcacacctgattttaaaatcaccaagaccatcgttaacggtaacgaggtcgtaactcaa

taaatgactgataacaataccgccctaaagaaagctggcctgaaagtaacgcttccacgt

ttaaaaatcctggaagttcttcaggagccggacaaccatcacgtcagtgcggaagattta

tacaaacgtctgatcgatatgggtgaagaaattggtctggctacggtatatcgcgtactg

aaccagtttgacgacgctggtatcgtcacccgccacaattttgaaggcggtaaatccgta

tttgaactgacacagcaacatcaccacgatcacctgatctgcctcgactgcggcaaggtt

atcgaatttagtgatgattcaatcgaagcgcgtcagcgtgaaattgccgcaaaacatggc

attcgcctgactaaccacagtctctatctttacggtcactgtgccgaaggcgattgccgc

gaagatgagcacgcgcacgaaggcaaataaatggtaagcaacgcctccgaattaggacgc

aatggcgtacatgatttcatcctcgttcgcgctaccgctatcgtcctgacgctctacatc

atttatatggtcggttttttcgccaccagtggcgagctgacatatgaagtctggatcggt

ttcttcgcctctgcgttcaccaaagtgttcaccctgctggcgctgttttctatcttgatc

catgcctggatcggcatgtggcaggtgttgaccgactacgttaaaccgctggccttgcgc

ctgatgctgcaactggtgattgtcgttgcactggtggtttacgtgatttatggattcgtt

gtggtgtggggtgtgtgaatgagactcgagttttcaatttatcgctataacccggatgtt

gatgatgctccgcgtatgcaggattacaccctggaagcggatgaaggtcgcgacatgatg

ctgctggatgcgcttatccagctgaaagagaaagatcccagcctgtcgttccgtcgctcc

tgtcgtgaaggtgtgtgcggttccgacggtctgaacatgaacggcaagaatggtctggcc

tgtattaccccgatttcggcactcaaccagccgggcaagaagattgtgattcgccctctg

ccaggtttaccggtgatccgcgatttggtggtagacatgggacaattctatgcgcaatat

gagaaaattaagccttacctgttgaataatgaacaaaatccgccagctcgcgagcattta

cagatgccagagcagcgcgaaaaactcgacgggctgtatgaatgtattctctgcgcatgt

tgttcaacctcttgtccgtctttctggtggaatcccgataagtttatcggcccggcaggc

ttgttagcggcatatcgtttcctgatcgatagccgtgataccgagactgacagccgcctc

gacggtttgagcgatgcattcagtgtattccgctgtcacagcatcatgaactgcgtcagt

gtatgtccgaaggggctgaacccgacgcgcgccatcggccatatcaagtcgatgttgttg

caacgtaatgcgtaaatgagtagcgtagatattctggtccctgacctgcctgaatccgta

gccgatgccaccgtcgcaacctggcataaaaaacccggcgacgcagtcgtacgtgatgaa

gtgctggtagaaatcgaaactgacaaagtggtactggaagtaccggcatcagcagacggc

attctggatgcggttctggaagatgaaggtacaacggtaacgtctcgtcagatccttggt

cgcctgcgtgaaggcaacagcgccggtaaagaaaccagcgccaaatctgaagagaaagcg

tccactccggcgcaacgccagcaggcgtctctggaagagcaaaacaacgatgcgttaacc

ccggcgatccgtcgcttgctggctgaacataacctcgacgccagcgccattaaaggcacc

ggcgtgggtggtcgtctgacccgtgaagatgtggaaaaacatctggcgaaatccccggcg

aaagagtctgcaccggcagcggctgctccggcggcgcaaccggctctggctgcacgtagt

gaaaaacgtgtcccgatgactcgcctgcgtaagcgtgtggcagagcgtctgctggaagcg

aaaaactccaccgccatgctgaccacgttcaacgaagtcaacatgaagccgattatggat

ctgcgtaagcagtacggtgaagcgtttgaaaaacgccacggcatccgtctgggctttatg

tccttctacgtgaaagcggtggttgaagccctgaaacgttacccggaagtgaatgcttct

atcgacggcgatgacgtggtttaccacaactatttcgacgtcagcatggcggtttctacg

ccgcgcggcctggtgacgccggttctgcgtgatgtcgataccctcggcatggcagacatc

gagaagaaaatcaaagagctggcagtcaaaggccgtgatggcaagctgacggttgaagat

ctgaccggtggtaacttcaccatcaccaacggtggtgtgttcggttccctgatgtctacg

ccgatcatcaacccgccgcagagcgcaattctgggtatgcacgctatcaaagatcgtccg

atggcggtgaatggtcaggttgagatcctgccgatgatgtacctggcgctgtcctacgat

caccgtctgatcgatggtcgcgaatccgtgggcttcctggtaacaatcaaagagttgctg

gaagatccgacgcgtctgctgctggacgtgtagatgagtaagattatcgcgaccttgtat

gcggtaatggacaagcgccccctgcgggcgctttccttcgtgatggcgcttctgttagca

ggatgtatgttttgggacccatcacgtttcgccgcgaagaccagtgatctggaaatctgg

catggtttattgctgatgtgggccgtctgtgctggtgtgattcacggcgtgggctttcgt

ccgcagaaggttctttggcaagggattttttgcccattgcttgccgatattgttctcatt

gtcgggctgattttcttcttcttttaagtgaatacaacgctgtttcgatggccggttcgc

gtctactatgaagataccgatgccggtggtgtggtgtaccacgccagttacgtcgctttt

tatgaaagagcacgcacagagatgctgcgtcatcatcacttcagtcaacaggcgctgatg

gctgaacgcgttgcctttgtggtacgtaaaatgacggtggaatattacgcacctgcgcgg

cttgacgatatgctcgaaatacagactgaaataacatcaatgcgtggcacctctttggtt

ttcacgcaacgtattgtcaacgccgagaatactttgctgaatgaagcagaggttctggtt

gtttgcgttgacccactcaaaatgaagcctcgtgcgcttcccaagtctattgtcgcggag

tttaagcagtgaatggccagagcgcgtggacgaggtcgtcgcgatctcaagtccgaaatc

aacattgtaccgttgctggacgtactgctggtgctgttgctgatctttatggcgacagcg

cccatcatcacccagagcgtggaggtcgatctgccagacgctactgaatcacaggcggtg

agcagtaacgataatccgccagtgattgttgaagtgtctggtattggtcagtacaccgtg

gtggttgagaaagatcgcctggagcgtttaccaccagagcaggtggtggcggaagtgtcc

agccgtttcaaggccaacccgaaaacggtctttctgatcggtggcgcaaaagatgtgcct

tacgatgaaataattaaagcactgaacttgttacatagtgcgggtgtgaaatcggttggt

ttaatgacgcagcctatctaaatgaaactcatcagtaatgatctgcgcgatggcgataag

ttgccacatcgtcatgtctttaacggcatgggttacgatggcgataatatttcaccgcat

ctggcgtgggatgatgttcctgcgggaacgaaaagttttgttgtcacctgctacgacccg

gatgcgccaaccggctccggctggtggcactgggtagttgttaatttacccgctgatacc

cgcgtattaccgcaagggtttggctctggtctggtagcaatgccagacggcgttttgcag

acgcgtaccgactttggtaaaaccgggtacgatggtgcagcgccgccgaaaggcgaaacc

catcgctacatttttaccgttcacgcgctggatgtagagcgtattgatgtcgatgaagga

gccagcggcgcgatggtcgggtttaacgttcatttccactctctggcaagcgcatcgatt

accgcgatgttcagttaaatgagtcaggtaagcactgaatttatcccgacccgtattgct

attcttacggtttctaatcggcgcggtgaagaagacgatacctccggtcactatttgcgc

gattcggcgcaagaagcgggccatcacgttgtcgataaagccattgtgaaagaaaaccgc

tacgctattcgcgctcaggtatctgcgtggatcgccagcgacgatgtacaagtggtgttg

attacggggggtactggcctgacggaaggtgatcaggctcccgaagcattgctgccgttg

ttcgaccgtgaagttgaaggttttggtgaagtgttccgtatgttgtcgtttgaagagatt

ggcacttccacgttgcaatctcgtgcggtagcgggcgtcgctaacaaaacgctgattttc

gccatgccgggttcgaccaaagcgtgccgtaccgcatgggaaaatatcatcgcgccgcag

ctggatgcccgtacgcgtccgtgtaatttccatccacatttgaagaaataaatgtcgcaa

ctgacccatatcaacgccgctggcgaagcgcacatggtggatgtctccgccaaagcggaa

accgtgcgtgaagcgcgcgccgaagcctttgtcaccatgcgcagcgagacgctggcgatg

attattgatggtcgccaccacaaaggcgacgtatttgccactgcgcgtattgccggtatt

caggcggcaaaacgcacgtgggatctgatcccgctgtgtcatccgctgatgctcagcaaa

gttgaagtcaatttacaggccgagccggagcacaatcgggtgcgtatagaaaccttatgc

cgcctgaccgggaaaaccggtgtcgaaatggaagcgttaaccgcggcctccgtggcggcg

ctgaccatttatgacatgtgcaaagcggtgcaaaaagatatggtgattggtccggtacgt

ttgctggcgaagagcggcggcaagtcgggtgactttaaggtggaagcggatgattaaatg

aagtctgtattaaaagtttcactggctgcactgaccctggcttttgcggtttcttctcat

gccgcggataaaaaattagttgtcgcgacggataccgccttcgttccgtttgaatttaaa

cagggcgataaatatgtgggctttgacgttgatctgtgggctgccatcgctaaagagctg

aagctggattacgaactgaagccgatggatttcagtgggatcattccggcactgcaaacc

aaaaacgtcgatctggcgctggcgggcattaccatcaccgacgagcgtaaaaaagcgatc

gatttctctgacggctactacaaaagcggcctgttagtgatggtgaaagctaacaataac

gatgtgaaaagcgtgaaagatctcgacgggaaagtggttgctgtgaagagcggtactggc

tccgttgattacgcgaaagcaaacatcaaaactaaagatctgcgtcagttcccgaacatc

gataacgcctatatggaactgggcaccaaccgcgcagacgccgttctgcacgatacgcca

aacattctgtacttcatcaaaaccgccggtaacggtcagttcaaagcggtaggtgactct

ctggaagcgcagcaatacggtatagcgttcccgaaaggtagcgacgagctgcgtgacaaa

gtcaacggcgcgttgaaaaccctgcgcgagaacggaacttacaacgaaatctacaaaaaa

tggttcggtactgaaccgaaataaatgagtcgtcgcgcaggtacgccaacagcaaaaaaa

gtgacgcagttagtgaacgtggaagagcacgttgaagggttccgccaggtcagagaggcg

catcggcgcgagcttattgatgattacgttgagctgatttctgacttgatcagggaagtg

ggggaagctcgccaggtagacatggctgctcgtttgggagtttcgcaaccgacggtggct

aaaatgcttaagcgactggcaactatggggctgattgaaatgatcccctggcgaggcgtg

tttttaacggcagaaggagagaagctggcgcaggaaagccgcgagcgacatcagatagtc

gaaaatttcttattggtgttgggcgtcagtccggaaatcgcccgtcgcgacgcggaaggc

atggagcaccatgttagtgaagagacgctggatgcctttcgtttgtttacccagaaacac

ggtgccaaatgaatgaatatgaaattgaaaacattattcgcagcggccttcgctgttgtc

ggcttttgcagtaccgcctctgcggtaacttatcctctgccaaccgacgggagtcgcctg

gttggtcagaatcaggtgatcaccattcctgaaggtaacactcagccgctggagtatttt

gccgcggagtaccagatggggctttccaatatgatggaagcgaacccgggtgtggatacc

ttcctgccgaaaggcggtactgtcctgaacattccgcagcagctgatcctgccggatacc

gttcatgaaggcatcgtcattaacagtgcagagatgcgtctgtattactatccgaaaggg

accaacaccgttatcgtgctgccgatcggcattggtcagttaggcaaagatacgcctatc

aactggaccaccaaagttgagcgtaagaaagcaggcccgacctggacgccgaccgccaaa

atgcacgcagagtaccgcgctgcgggcgaaccgcttccggctgtcgttccggcaggtccg

gataacccgatggggctgtatgcactctacatcggtcgcctgtatgctatccatggcacc

aacgccaacttcggtatcggcctgcgtgtaagtcatggttgtgtgcgtctgcgtaacgaa

gacatcaaattcctgttcgagaaagtaccggtcggtacccgcgtacagtttattgatgag

ccggtaaaagcgaccaccgagccagacggcagccgttatattgaagtccataatccgctg

tctaccaccgaagcccagtttgaaggtcaggaaattgtgccaattaccctgacgaagagc

gtgcagacagtgaccggtcagccagatgttgaccaggttgttcttgatgaagcgattaaa

aaccgctccgggatgccggttcgtctgaattaagtgaatataaacgtcgccgaattgtta

aatgggaattacattctgttattatttgtggtcctcgcgcttgggctatgtctcggaaag

ttacgacttggttcgatccaactgggtaattccattggcgttttagtcgtatcgctgtta

ttaggccaacaacatttcagcattaacaccgacgcgcttaatcttggctttatgctgttt

attttctgcgttggggttgaagccggaccgaactttttttccattttttttcgcgatgga

aaaaattacctaatgttagcactggtgatggttggcagtgcgctggtgatcgccttaggg

ttaggtaagctgtttggctgggatattggcctgacggccggtatgttagcaggctctatg

acgtcgacaccggttctggtcggtgctggcgatacactgcgtcattccggcatggaaagc

aggcagctctcactggcactggataatctgagcctcgggtatgccttaacctatttaatc

ggtctggtgagtttgattgttggtgcgcgttacttgccgaaattgcagcatcaggactta

cagaccagcgcccagcaaatcgcccgcgaacgtggcctggacactgatgccaaccgtaag

gtttatttaccggtgatccgcgcctatcgcgtcggcccggagctggtggcctggaccgac

ggcaaaaatctgcgtgaactgggtatttatcgacaaaccggctgctacattgaacgtatt

cgacgtaacgggattctggcaaatccagacggtgatgccgtgctacaaatgggcgatgaa

atagcgttggtaggctatcccgacgcccatgcccgactcgatcccagcttccgtaacggc

aaagaagttttcgatcgtgaccttctcgatatgcgtatcgtcactgaagaagtggtcgtt

aaaaaccataacgccgttggcaaacgtctcgcacaactgaagttgaccgaccacggttgc

ttccttaaccgcgtcattcgtagccagattgagatgccgatagatgacaacgtcgtgctt

aacaaaggtgacgttttacaagtcagcggcgatgcccgtcgcgtaaaaaccatcgccgat

cgcattggctttatctcgattcacagccaggtcactgacttgctggcattctgcgccttc

tttgttattgggctgatgatcgggatgatcacattccagttcagcacattcagtttcggc

atggggaacgctgccgggttgttattcgccggaattatgctgggctttatgcgtgctaac

cacccgaccttcggttacattccgcaaggtgcattaagcatggtgaaagagttcggcttg

atggtgtttatggcaggcgttggtctgagcgccggtagcggtattaataacggcctgggc

gcgattggcggtcaaatgttgattgccggattgattgtcagtctggtgcccgtggttatc

tgtttcttgttcggtgcttatgtattgcgaatgaaccgcgcactgttgttcggcgcaatg

atgggcgcacgcacctgcgcgccggcaatggagatcatcagtgatacagctcgcagtaac

atcccggcgctgggctatgcgggcacctacgcaatcgccaacgttctgctgacgctggca

gggacaatcatcgtcatggtatggccaggattaggataagtgaaacataaacaacgttgg

gcgggggcaatctgctgttttgtcctcttcattgtggtgtgcctttttctggcgacgcac

atgaaaggcgcttttcgggctgccgggcatcctgaaatcggcttgctgtttttcattctt

cctggagcagttgccagtttcttttcacagcgtagagaagtcctgaaacctctgtttggc

gcaatgctggcggcaccctgttcaatgcttattatgcggctgtttttttcaccgacccgc

tcattctggcaagagctggcatggttactaagcgcggtgttctggtgtgcgctgggggca

ctgtgtttcttatttatcagtagtttgtttaaaccacagcacagaaaaaatcagtaaatg

cgcgcgatcggtaaattgcctaagagcgtgttgatactggaatttatcggaatgatgttg

ctggcggtggcgctgctgtcggtaagcgactccctgtcgctgcctgagccattttctcgg

ccagaagtgcagattctgatgatttttctcggtgttttgctcatgcttcccgctgcggtg

gtggttattcttcaggtggcaaaacgtcttgccccacagctgatgaaccgtccaccgcaa

tattcacgttcagaaagagaaaaagataatgacgccaaccattgagtgaaaattgccata

ttgtcccgggatggaacgctctattcgtgtaagcggctgcgtgaagccgctatacagcgc

ggtcacctggttgaaattcttgatccgctttcttgctacatgaacataaatcctgcggcg

tcttctattcactacaaaggccgcaagttaccccattttgacgcagtgatcccgcgtatt

ggcaccgccattaccttttatgggacggcggcactgcgccagttcgagatgctggggagc

tatccgctcaatgagtcggtcgccattgcccgggcgcgtgacaaattgcgttccatgcaa

ctgctggcgcgtcagggcatcgacctgcctgtcacgggcattgcgcattcgccggatgat

accagcgatttaatcgacatggtcggtggtgcgccgctggtggtcaagttggttgaaggc

acgcagggaattggcgtcgtgctggcggagacgcgtcaggcggcggaaagcgtgattgac

gctttccgcggtctgaacgcgcatattctggtgcaggaatatatcaaagaggcgcaaggg

tgcgatatccgctgtctggttgttggcgatgaagtggtcgctgcgattgaacggcgggcg

aaagagggcgattttcgttccaatttgcatcgtggcggcgcggcaagtgtcgccagtatc

acaccacaggagcgtgaaatcgcgataaaagccgcgcgaacgatggcgctggacgttgct

ggtgtggatattctgcgtgctaatcgcgggccgttggtgatggaagtgaatgcgtcgccg

gggctggaaggaatagaaaaaaccaccggtatcgacatcgcgggtaaaatgatccgctgg

atcgaacgctacgctacgacagaatattgcctgaaaacgggtggttagatgtttgagtat

ttacccgaactgatgaaagggctgcacaccagcctgacgctaaccgttgcctcgctgatt

gtggcactgattctggcgttgatttttaccatcatcctgacgctgaaaacgccggtgctg

gtgtggctggtgcggggttatatcacgctgtttaccggtacaccgctgctggtgcagatc

ttcctgatttattacgggccgggtcagttcccgacgttgcaggagtatccggcactgtgg

catttgttgtcagaaccgtggttatgtgcgctgattgcgttgtcgctgaacagtgcggcg

tataccacgcagctgttttacggtgcaattcgtgcgatcccggaaggtcagtggcagtcc

tgtagcgccctgggaatgagcaaaaaagatacgctggcgatcctgctgccgtatgccttt

aaacgctcgctctcttcttattccaacgaagtggtgctggtgttcaaaagtacctctctg

gcttacaccattacgctgatggaagtgatgggatacagccagttgttgtacggacgcacc

tacgatgtaatggtgttcggcgcggcagggattatttatctggtcgtcaacggcctgctg

acgctgatgatgcgtctgatcgagcgcaaagcactggcatttgaacggcggaattaaatg

aatgaattttttcctttagcaagcgccgccgggatgaccgtcggccttgccgtttgtgca

ttgattgtcggactggcgctggcgatgttctttgcggtatgggagtcggcaaaatggcgt

cctgtcgcgtgggcaggttcagcgctggtaaccattctgcgtggcctgccagaaattctg

gtggtgctgtttatctattttggttcctcgcagctgctgctgacgctttcggatggcttc

actatcaatcttgggttcgtgcagatcccggtgcagatggacattgagaacttcgacgtg

agtccgttcctttgtggtgtcatcgctctgtcactgctgtatgccgcctatgcctcgcaa

acgctgcgcggcgcgttgaaagcggtgccgatgggtcagtgggaatccggccaggcgctg

gggctgtcgaaatcggctatctttttccgtctggtgatgccgcagatgtggcgtcatgcg

ctgcctggcctcggtaaccagtggctggtgctgctgaaagataccgcgctggtcagtttg

attagtgtgaatgatttaatgctgcaaaccaaaagcatcgctactcgtacccaggaacca

tttacctggtacattgtggcggcggcgatttacctggtgatcaccctgctcagtcagtac

attctcaaacgcattgacctgcgcgcgacacgttttgagcggaggcccagctaaatgcaa

ttttccacaactccaactctggaaggccagaccatcgttgaatattgcggtgtggtgact

ggcgaagcgattttaggcgcgaatattttccgtgatttctttgccggtatccgcgatatc

gttggcggacgctccggtgcctacgaaaaagagctacgtaaagcgcgggagatcgccttt

gaggagcttggctcccaggcgcgggcgctgggggccgatgccgtagtcggtattgatatc

gactacgaaacggtcgggcaaaacggcagtatgctgatggttagcgtcagcggtacggcg

gtgaaaacgcgtcgatgaatgacggcaattgccccggttattaccattgatggcccaagc

ggtgcagggaaaggcaccttgtgtaaggctatggcggaagcgttgcaatggcatctgctg

gattcgggtgcaatttatcgcgtactggcattggcggcattacatcaccatgttgatgtt

gcgtcggaagacgcgctggtgccgctggcatcccatctggatgtacgttttgtgtcaacc

aatggcaatctggaagtgatcctcgaaggggaagatgtcagcggcgaaattcgtactcag

gaagtggcgaatgcagcttcacaagtcgcggcattcccacgcgttcgtgaagcattattg

cgtcgccaacgcgcgtttcgcgaattaccaggcctgattgccgatggccgcgacatggga

acggtggtattccctgatgcgccagtgaaaattttccttgacgcctcctcggaagaacgt

gcgcatcgccgcatgctacagttgcaggagaagggctttagtgttaactttgagcgcctt

ttggccgagatcaaagaacgcgacgaccgcgatcgtaaccgagcggtagcgccactggtt

ccggcagccgatgctttagtgttggattccaccaccttaagcattgagcaagtgattgaa

aaagcgctacaatacgcgcgccagaaattggctctcgcataaatgactgaatcttttgct

caactctttgaagagtccttaaaagaaatcgaaacccgcccgggttctatcgttcgtggc

gttgttgttgctatcgacaaagacgtagtactggttgacgctggtctgaaatctgagtcc

gccatcccggctgagcagttcaaaaacgcccagggcgagctggaaatccaggtaggtgac

gaagttgacgttgctctggacgcagtagaagacggcttcggtgaaactctgctgtcccgt

gagaaagctaaacgtcacgaagcctggatcacgctggaaaaagcttacgaagatgctgaa

actgttaccggtgttatcaacggcaaagttaagggcggcttcactgttgagctgaacggt

attcgtgcgttcctgccaggttctctggtagacgttcgtccggtgcgtgacactctgcac

ctggaaggcaaagagcttgaattcaaagtaatcaagctggatcagaagcgcaacaacgtt

gttgtttctcgtcgtgccgttatcgaatccgaaaacagcgcagagcgcgatcagctgctg

gaaaacctgcaggaaggcatggaagttaaaggtatcgttaagaacctcactgactacggt

gcattcgttgatctgggcggcgttgacggcctgctgcacatcactgacatggcctggaaa

cgcgttaagcatccgagcgaaatcgtcaacgtgggcgacgaaatcactgttaaagtgctg

aagttcgaccgcgaacgtacccgtgtatccctgggcctgaaacagctgggcgaagatccg

tgggtagctatcgctaaacgttatccggaaggtaccaaactgactggtcgcgtgaccaac

ctgaccgactacggctgcttcgttgaaatcgaagaaggcgttgaaggcctggtacacgtt

tccgaaatggattggaccaacaaaaacatccacccgtccaaagttgttaacgttggcgat

gtagtggaagttatggttctggatatcgacgaagaacgtcgtcgtatctccctgggtctg

aaacagtgcaaagctaacccgtggcagcagttcgcggaaacccacaacaagggcgaccgt

gttgaaggtaaaatcaagtctatcactgacttcggtatcttcatcggcctggacggcggc

atcgacggcctggttcacctgtctgacatctcctggaacgttgcaggcgaagaagcagtt

cgtgaatacaaaaaaggcgacgaaatcgctgcagttgttctgcaggttgacgcagaacgt

gaacgtatctccctgggcgttaaacagctcgcagaagatccgttcaacaactgggttgct

ctgaacaagaaaggcgctatcgtaaccggtaaagtgactgcagttgacgctaaaggcgca

accgtagaactggctgatggcgttgaaggttacctgcgtgcttctgaagcatcccgtgac

cgcgttgaagacgctaccctggttctgagcgttggcgacgaagttgaagctaaattcacc

ggcgttgatcgtaaaaaccgcgcaatcagcctgtctgtacgtgcgaaagacgaagctgac

gagaaagatgcaatcgcaactgttaacaaacaggaagatgcaaacttctccaacaacgca

atggctgaagctttcaaagcagctaaaggcgagtaaatggaacagctgcgtgccgaatta

agccatttactgggcgaaaaactcagtcgtattgagtgcgtcaatgaaaaagcggatacg

gcgttgtgggctttgtatgacagccagggaaacccaatgccgttaatggcaagaagcttt

agtacgcccggaaaagcccgacaactggcatggaaaaccaccatgctggcaagaagtggg

actgtccgtatgccgactatttatggtgtgatgacgcatgaagaacaccccggccccgat

gtcctgctactggagcggatgcgtggtgtttcggtggaggcaccagcccgaacaccagaa

cgctgggaacaactcaaagaccaaatcgttgaagccttactggcctggcaccgtcaggac

agtcgcggttgcgtcggcgcggtcgacaatactcaggaaaatttctggccctcatggtac

cggcaacatgttgaagtgctatggaccacgctcaatcagttcaataacaccggtctgacg

atgcaggataagcggatcctgtttcgcactcgcgaatgtctcccggcattatttgaaggc

tttaacgacaatagtgtgttgattcacggtaacttctgtttacgcagcatgctgaaagat

tcgcgcagcgatcagttactggcgatggtcgggccgggactaatgctttgggcaccgcga

gaatacgaactgttccgactaatggataattctctggcggaagatttgctctggagttac

ctgcaacgcgcgccagtggcggagtcgttcatctggcggcgttggttgtatgtgttatgg

gatgaagttgcgcaactggttaataccggacgatttagtcggcgcaacttcgatctggca

tcaaaatcactcttgccgtggctcgcctgaatgaactatcgtattattccggtcaccgca

ttctcccagaactgttcattgatctggtgtgaacaaacccgcctggccgcactggtcgat

cctggcggcgatgcggaaaaaatcaaacaggaagttaatgacagcggcctgacactgatg

cagatcctgctgacgcatggtcatctggaccacgttggcgcagcggcggaactggcgcaa

cattacggcgtgccggtttttggcccggaaaaagaagatgagttctggctgcaaggcttg

cctgcgcaaagtcgtatgtttggtctggaagagtgtcagccgctgacgccagatcgttgg

ctgaaagaaggcgataccatcagcatagggaatgtgactttacaggtgttacattgccct

gggcatacgccgggtcatgtcgtgttttttgatgatcgggcaaagctgctgatttctggc

gatgttattttcaaaggcggagtagggcgcagtgacttcccgcgtggcgatcataatcaa

ctgatttcttcaatcaaagataaattgctgccactgggggatgacgtgacatttattccg

ggtcacggaccattatccacacttggttatgaacgcctgcataatcccttcctgcaagac

gaaatgcccgtctggtaaatgtttgagaacattaccgccgctcctgccgacccgattctg

ggcctggccgatctgtttcgtgccgatgaacgtcccggcaaaattaacctcgggattggt

gtctataaagatgagacgggcaaaaccccggtactgaccagcgtgaaaaaggctgaacag

tatctgctcgaaaatgaaaccaccaaaaattacctcggcattgacggcatccctgaattt

ggtcgctgcactcaggaactgctgtttggtaaaggtagcgccctgatcaatgacaaacgt

gctcgcacggcacagactccgggtggcactggcgcactacgcgtagctgccgatttcctg

gcaaaaaataccagcgttaagcgagtgtgggtgagcaacccaagctggccgaaccataag

agcgtctttaactctgcaggtctggaagttcgtgaatacgcttattatgatgcggaaaac

cacacccttgacttcgatgcactgattaacagcctgaacgaagctcaggctggcgacgta

gtgctgttccatggctgctgccacaacccaaccggtatcgaccctacgctggaacaatgg

cagacactggcacaactctccgttgagaaaggctggttaccgctgtttgacttcgcttac

cagggttttgcccgtggtctggaagaagatgctgaaggactgcgcgctttcgcggctatg

cataaagagctgattgttgccagttcctactctaaaaactttggcctgtacaacgagcgt

gttggcgcttgtactctggttgctgccgacagtgaaaccgttgatcgcgcattcagccaa

atgaaagcggcgattcgcgctaactactctaacccaccagcacacggcgcttctgttgtt

gccaccatcctgagcaacgatgcgttacgtgcgatttgggaacaagagctgactgatatg

cgccagcgtattcagcgtatgcgtcagttgttcgtcaatacgctgcaggaaaaaggcgca

aaccgcgacttcagctttatcatcaaacagaacggcatgttctccttcagtggcctgaca

aaagaacaagtgctgcgtctgcgcgaagagtttggcgtgtatgctgttgcttctggtcgc

gtaaacgtggccgggatgacaccagataacatggctccgctgtgcgaagcgattgtggca

gtgctgtaaatgcgtgtcatcaccctggcgggtagtcctcgctttccttctcgctccagc

tccttgctggaatatgcgcgggaaaaactaaatggcctggatgtagaggtttatcactgg

aatctgcaaaacttcgccgcggaagatctgctttatgctcgtttcgatagtccggcactc

aataccttcaccgaacagttgcaacaggctgacgggctgattgtcgccacgcctgtgtat

aaagccgcttattccggtgcgctgaaaaccctgctcgacctgctgccagaacgtgcgttg

caaggcaaagtggtgctaccgctggcgacgggcggtacggtggcccatctgctggcggtc

gattatgcccttaagccagttttaagtgcgctgaaagcccaggagatcctgcacggcgtg

tttgccgatgactcacaagtaattgattaccatcacaaaccccagttcacgccgaattta

caaacccgtcttgataccgcgctagaaactttctggcaggcattgcaccgccgcgatgtt

caggttcctgatcttcattctctgcgaggtaatgcccatgcgtaaatgcgtaccgttttg

aacattctgaactttgtgcttggcggatttgccaccactctgggctggttgttggcgact

ctggtcagtattgtgctgatttttaccttaccgctgacacgatcctgctgggagatcact

aaactgtctctggtgccttatggcaatgaagctattcatgtcgatgaactgaacccggct

ggcaaaaatgtgctgctgaatactggcggtacggtattgaatattttctggctgattttc

tttggctggtggttatgcctgatgcacattgcaacgggcatcgcacaatgtatttcaatc

attggcattcctgtcggcattgcgaactttaaaattgccgctattgcactatggccggtt

ggtcgtcgcgtggtatcggtagaaacagcgcaagctgcgcgtgaagccaatgcacgtcgt

cgttttgaataaatgaaaaccggcatcgtgaccaccttgattgcattatgtttgccggta

tccgtttttgccaccacgctgcggctttcaaccgatgtcgatcttctggtactcgacggc

aagaaggtttccagctctctgctgcgcggtgcggacagcattgaactggataatggaccg

catcagttagtgtttcgcgttgagaagacgattcatctttccaatagtgaagaacggctg

tatatctccccaccgctggtggtcagttttaatacccagctcatcaaccaggtcaatttt

cgcctgcctcgcctggagaatgagcgggaagctaaccattttgatgccgcgccgcgcctt

gaattgttggatggcgatgcgacgccgattccggtaaagctggatattctcgccattacc

tcaacggcaaaaacgattgattatgaggtcgaggttgaacgctataacaaatccgcgaaa

cgcgcttcactaccgcaatttgccacgatgatggcagatgacagtacgctgctttcgggt

gtttccgagctggatgctattccaccgcagtctcaggtgctcacagaacaacggctgaag

tattggtttaaactggctgacccacaaacgcgaaatactttcctgcaatgggcggaaaaa

caaccatcttcctgaatggatcgtattgttagttcttcacatgaccgtacatcactgctt

agcacccataaagtgctgcgtaatacctattttctgctgagcctgacgctggccttttcg

gcgattaccgcaactgccagtacggtgctgatgctgccatctccgggtctgattctgacg

ctggtgggtatgtatggtttgatgttcctgacctataaaacggcgaataagccgaccggg

attatctccgcattcgcctttaccggttttctgggttatatcctcggaccaattctgaac

acctatctgtctgccggaatgggtgacgtaatcgctatggcactgggcggaacggcgtta

gtgttcttctgctgctctgcatatgtgctgaccacccgcaaagatatgtcgttcctcggc

ggtatgctgatggcgggtattgtggtggtgctgattggtatggttgctaatatcttcctg

cagctgcctgctctgcatctggcgatcagcgcggtcttcattttgatctcctctggcgct

attttgtttgaaaccagcaacatcattcatggcggtgaaacgaactatattcgtgccacg

gttagcctgtatgtttctctgtacaacatcttcgtcagcctgctgagcattctgggcttc

gccagccgcgattaaatgccacatcacattgttattgttgaagatgagccggttacccag

gcgcgattacaatcctacttcactcaggaggggtataccgtttccgttacggcgagcggt

gccgggctgcgggaaattatgcagaatcagccggtggatttaattctgctggatatcaac

ttgcccgatgaaaatggcctgatgttaacccgcgccctgcgagaacgctcaacggtgggg

attattctggttaccggacgcagcgatcggattgaccgtattgttgggctggaaatgggc

gcagacgattacgtcaccaaaccgctggaactgcgcgaactggtagtacgggtaaaaaat

ctgctctggcgaatcgacctcgcgcgacaagttcaaccgctcactcaggacaactgctat

cgctttgcgggttattgcctgaatgtgtcgcgccatacgctggagcgggatggtgagccg

attaaactgacccgcgcagagtatgaaatgttggtggcatttgtgacgaatccgggagaa

attctcagccgtgaacgtctgctgcgtatgctctctgcgcgtcgggtggaaaaccctgac

ctgcgcaccgtcgatgtgttaattcgtcgtttacgccataaactcagcgcggatttactg

gtgacgcaacatggcgaaggttatttcttagccgctgatgtgtgctgaatggctaatgtt

acggtgacttttactattaccgaattttgcctgcataccggcatctctgaagaggaattg

aatgaaattgtcggtttgggggtggttgaaccgcgtgagattcaggaaacaacctgggta

tttgacgaccatgccgccatcgtggtgcaacgcgcggtgcgcctgcgtcatgaactggct

ctggactggccggggatcgcggtggcgctgacgttaatggatgatattgcgcacctgaag

caggaaaaccgcctgctgcgccagcggctttcccggtttgtagctcatccgtgaatggaa

ttaaaggattattacgccatcatgggcgtgaaaccgacggacgatctcaagacaatcaag

accgcctatcgtcgacttgcccgcaaataccatcctgatgtcagcaaagaaccggatgcc

gaagcccgcttcaaagaggtcgctgaagcctgggaagtgttaagtgatgaacaacgtcgc

gctgagtatgatcagatgtggcaacatcgcaacgatccgcaatttaaccgtcagttccat

catggcgacggacagagttttaacgccgaagattttgacgatatcttctcgtcaattttc

ggtcagcatgcccgccagagccgtcaacgccccgccacacgcggccacgatattgaaatc

gaagtggcggtattcctcgaagaaacgcttactgagcataagcgtaccatcagctataac

ctgccggtttataacgcctttggcatgatcgaacaggaaatcccgaaaacgctgaatgtg

aagatcccggcgggcgtcggcaatggtcaacgtatccgcctgaaaggccaggggacgccg

ggcgaaaacggcggtccaaatggcgacctgtggctggtgattcatattgcgccacatccg

ctgtttgatattgtcggccaggatctggaaattgtggtgccggttagcccgtgggaagcg

gcgctgggtgctaaagtcaccgttccaacactgaaagaaagcattttgctgactatcccg

cctggcagccaggccgggcaacgattgcgcgttaaaggcaaaggtctggtgagcaaaaaa

cagaccggcgatctgtatgcggtactgaaaatcgtgatgccgccgaaaccggatgaaaac

actgccgcgctgtggcagcaactggcagacgcccagtcgtcttttgatccacgtaaagat

tgggggaaagcataaatgaacgagttttctatcctctgtcgtgtgctgggttcgctctat

taccgccaaccacaagatcctttactggtgccgctgtttactctgattcgtgaggggaaa

ctggctgcgaactggccactggagcaggatgagttactgacgcgtttacagaaaagttgt

gatatgacgcaagtctctgccgattacaatgcgttgtttatcggcgatgaatgtgccgtg

ccgccatatcgtagcgcatgggttgaggacgcaacggaagcggaagtgcgcgcttttctt

tccgaacgagggatgccactggcggatacgccagccgatcacatcggcacattgctgctt

gcggcttcctggctggaagatcagtcaacggaagatgagagcgaagcactggaaacactg

ttcagtgagtatctgttaccctggtgtggcgcgttccttggcaaagtggaggcccatgca

accacgcctttctggcgcaccatggcaccgctaacccgtgatgccattagtgcaatgtgg

gacgagctggaagaggattctgaagagtaaatgaaaacgcgtattcatgttgtgcagggt

gatattaccaaactggccgttgatgtgattgtgaatgcggctaatccgtcattaatggga

ggcggcggcgtcgatggggccattcatcgcgcagcgggtccggccctgctggatgcctgt

ttaaaagtcaggcaacagcagggcgattgccctacggggcatgccgttattacgcttgca

ggcgatcttcccgctaaagccgtagtgcacaccgtcgggccagtctggcgtggtggtgaa

caaaacgaagaccagcttttgcaggatgcctatctcaatagcctacgactggtggcggca

aacagctatacgtcagtggcttttactgcaatcagtactggggtttatggttaccctcgt

gcggcagcggctgaaatcgcagtaaaaaccgtttcagaatttattacccgtcacgcttta

cccgaacaggtatactttgtctgttatgatgaagaaaacgcccacctctacgaaagactc

cttacccaacaaggagatgaatgaatgactgcaccatcccaggtattaaagatccgccgc

ccagacgactggcaccttcacctccgcgatggcgacatgttaaaaactgtcgtgccgtat

accagcgaaatttatggacgggctatcgtaatgcccaatctggctccgcccgtgaccacc

attgaggctgccgtggcgtatcgccaacgtattcttgacgccgtacctgccgggcacgat

ttcaccccattgatgacctgttatttaacagattcgctggatcctaatgagctggagcgc

ggatttaacgaaggcgtgttcaccgctgcaaaactttacccggcaaacgcaaccactaac

tccagccacggcgtgacgtcaattgacgcaatcatgccggtacttgagcgcatggaaaaa

atcggtatgccgctactggtgcatggtgaagtgacacatgcagatatcgacatttttgat

cgtgaagcgcgctttatagaaagcgtgatggaacctctgcgccagcgcctgactgcgctg

aaagtcgtttttgagcacatcaccaccaaagatgctgccgactatgtccgtgacggaaat

gaacggctggctgccaccatcactccgcagcatctgatgtttaaccgcaaccatatgctg

gttggaggcgtgcgtccgcacctgtattgtctacccatcctcaaacgcaatattcaccaa

caggcattgcgtgaactggtcgccagcggttttaatcgagtattcctcggtacggattct

gcgccacatgcacgtcatcgcaaagagagcagttgcggctgcgcgggctgcttcaacgcc

ccaaccgcgctgggaagttacgctaccgtctttgaagagatgaatgctctgcagcacttt

gaagcattctgttctgtaaatggcccgcagttctatgggttgccggtcaacgacacattc

atcgaactggtacgtgaagagcatcaggttgctgaaagcatcgcactgactgatgacacg

ctggtgccattcctcgccggggaaacggtacgctggtccgttaaacaataagtgaagcta

tacatttacgatcactgcccttactgcctcaaagcccgcatgattttcggcctgaaaaat

atccccgtcgaattacatgttctgctcaacgacgacgcagaaacacccacccggatggtc

ggtcaaaaacaggttcccattctgcaaaaagatgacagccgctatatgccagaaagcatg

gatatcgttcactatgtcgataaactcgacggcaaaccgttactgaccggcaaacgttcc

cctgcaattgaagagtggctgcgcaaggtcaatggctacgccaacaaactgctgttgccg

cgttttgccaaatcggcatttgatgagttttctactcccgccgcgcgcaaatatttcgtc

gacaagaaagaggccagcgcgggtaattttgccgacctgctggcccactctgacggtctg

attaagaatatcagcgatgatttacgtgcgctggacaaactgatcgtcaaaccgaacgcc

gtgaatggcgaactttcggaagatgatattcagctattcccgctactgcgtaatctgacg

ctggtagccggaattaactggccaagccgcgttgctgattaccgcgataatatggcgaaa

cagacacaaatcaatttgttatcatcaatggcgatttaaatgtttggctatcgcagtaac

gtgccaaaagtgcgcttaaccacagaccgactggtcgtgcgtctggtgcatgatcgtgat

gcctggcgtcttgcggattattacgcagagaatcgccatttcctcaagccctgggagcca

gtgcgcgacgaaagccactgctatccatcaggctggcaggccaggctggggatgattaac

gaatttcataaacaaggttcagctttctactttggcttattcgacccggacgaaaaagag

attattggcgttgccaatttttccaatgttgttcgcggctcttttcatgcctgctatctc

ggttattcgattgggcaaaaatggcagggcaaaggactcatgtttgaagccctgaccgca

gccattcgttatatgcagcgcacccaacatattcatcgcattatggctaattatatgccg

cacaataaacgtagcggtgatttactggcgcgactgggttttgaaaaagaaggctatgcg

aaagactatctgttgattgatggacaatggcgcgatcacgtactgacggcattaactacc

ccagactggacgcccggccgctaaatgtatacgaagattattggtactggcagctatctg

cccgaacaagtgcggacaaacgccgatttggaaaaaatggtggacacctctgacgagtgg

attgtcactcgtaccggtatccgcgaacgccacattgccgcgccaaacgaaaccgtttca

accatgggctttgaagcggcgacacgcgcaattgagatggcgggcattgagaaagaccag

attggcctgatcgttgtggcaacgacttctgctacgcacgctttcccgagcgcagcttgt

cagattcaaagcatgctgggcattaaaggttgcccggcatttgacgttgcagcagcctgc

gcaggtttcacctatgcattaagcgtagccgatcaatacgtgaaatctggggcggtgaag

tatgctctggtcgtcggttccgatgtactggcgcgcacctgcgatccaacagatcgtggg

actattattatttttggcgatggcgcgggcgctgcggtgctggctgcctctgaagagccg

ggaatcatctccacccatctgcatgccgacggtagctatggtgagttgctgacgctgcct

aatgctgaccgtgtgaatccagagaattcaattcatctgacgatggcgggcaacgaagtc

ttcaaggttgcggtaacggaactggcgcacatcgttgatgagacgctggcggcgaataat

cttgaccgttctcaactggactggctggttccgcatcaggctaacttgcgtattatcagt

gcaacggcgaaaaaactcggtatgtcgatggacaatgtcgtggtgacgctggatcgccac

ggtaatacctctgcggcctctgtcccgtgcgcgctggatgaagctgtacgcgacgggcgc

attaagccggggcagttggttctgcttgaagcctttggcggtggattcacctggggctcc

gcgctggttcgtttctagatgtttaagaatgcatttgctaacctgcaaaaggtcggtaaa

tcgctgatgctgccggtatccgtactgcctatcgcaggtattctgctgggcgtcggttcc

gcgaatttcagctggctgcccgccgttgtatcgcatgttatggcagaagcaggcggttcc

gtctttgcaaacatgccactgatttttgcgatcggtgtcgccctcggctttaccaataac

gatggcgtatccgcgctggccgcagttgttgcctatggcatcatggttaaaaccatggcc

gtggttgcgccactggtactgcatttacctgctgaagaaatcgcctctaaacacctggcg

gatactggcgtactcggagggattatctccggtgcgatcgcagcgtacatgtttaaccgt

ttctaccgtattaagctgcctgagtatcttggcttctttgccggtaaacgctttgtgccg

atcatttctggcctggctgccatctttactggcgttgtgctgtccttcatttggccgccg

attggttctgcaatccagaccttctctcagtgggctgcttaccagaacccggtagttgcg

tttggcatttacggtttcatcgaacgttgcctggtaccgtttggtctgcaccacatctgg

aacgtacctttccagatgcagattggtgaatacaccaacgcagcaggtcaggttttccac

ggcgacattccgcgttatatggcgggtgacccgactgcgggtaaactgtctggtggcttc

ctgttcaaaatgtacggtctgccagctgccgcaattgctatctggcactctgctaaacca

gaaaaccgcgcgaaagtgggcggtattatgatctccgcggcgctgacctcgttcctgacc

ggtatcaccgagccgatcgagttctccttcatgttcgttgcgccgatcctgtacatcatc

cacgcgattctggcaggcctggcattcccaatctgtattcttctggggatgcgtgacggt

acgtcgttctcgcacggtctgatcgacttcattgttctgtctggtaacagcagcaaactg

tggttgttcccgatcgtcggtatcggttatgcgattgtttactacaccatcttccgcgtg

ctgattaaagcactggatctgaaaacgccgggtcgtgaagatgcgactgaagatgcaaaa

gcaacaggtaccagcgaaatggcaccggctctggttgctgcatttggtggtaaagaaaac

attactaacctcgacgcatgtattacccgtctgcgcgtcagcgttgctgatgtgtctaaa

gtggatcaggctggcctgaagaaactgggcgcagcgggcgtagtggttgctggttctggt

gttcaggcgattttcggtactaaatccgataacctgaaaaccgagatggatgagtacatc

cgtaaccactaaatgagaaaaggatgctttgggctgatgtctctggcgttgttactgctg

gtgggctgtcgttcacatccggaaattccggtgaatgatgagcaatcgctggtgatggag

tcatctttattggctgcgggcatcagtgcagaaaagcccgtcctttcgacgtctgatatt

caaccttcagcatcctcaacgctttataacgaaaggcaagaacccgttaccgttcattat

cgtttttactggtatgacgccagagggctggagatgcatcctctggaaaggccacgcagc

gttaccattcccgcacattcggcggtaacgctgtacggcagcgccaattttctgggggcg

cacaaagtcagactttatctatatttgtaagtgccgtttcgcagcaataatcccatcacg

cgcgacgaattgctgtcgcgctttttcccgcagtttcatcccgtcacgacgtttaatagt

gggcttagtggcgggagttttctcattgaacatcagggccagcgttttgttgtgcgtcag

ccgcacgatcctgatgcgccgcagtccgcgttcttgcgccagtatcgggctttatcacaa

ctacccgcatgcattgcaccgaagccgcatttatatctccgtgactggatggtagtcgac

tatctgcccggcgcggtaaaaacgtatttgccggataccaacgaactggcaggcttgctg

tattatctacatcaacaaccacgttttggctggcgaataacgctgttgccgttactggaa

ctgtactggcagcaaagcgatccggcgcggcggacagtgggttggctgcgaatgttaaaa

tgtctgcgcaaagcgcgggaaccacggcctttacgcttaagtccattgcatatggatgtc

cacgccggaaatttagtgcatagcgcgtcagggttaaaactcatcgactgggagtatgca

ggagatggtgatatcgcgctggaactggcggcggtgtgggtggaaaatactgaacagcac

cggcaattggtcaatgactatgccactcgcgcgaagatttatccggcgcaattatggcgt

caggtcaggcgatggtttccctggctgctgatgctcaaagcagggtggtttgagtaccgc

tggcgacaaaccggcgatcaacaatttatcaggctggccgatgacacctggcggcagcta

ttaataaaacaataaatgaaaaaattactgcgtctttttttcccgctctcgctgcgggta

cgttttctgttggcaacggcagcggtagtactggtgctttcgcttgcctacggaatggtc

gcgctgatcggttatagcgtcagtttcgataaaactacgtttcggctgttacgtggcgag

agcaatctgttctatacccttgcgaagtgggaaaacaataagttgcatgtcgagttaccc

gaaaatatcgacaagcaaagccccaccatgacgctaatttatgatgagaacgggcagctt

ttatgggcgcaacgtgacgtgccctggctgatgaagatgatccagcctgactggctgaaa

tcgaatggttttcatgaaattgaagcggatgttaacgataccagcctcttgctgagtgga

gatcattcgatacagcaacagttgcaggaagtgcgggaagatgatgacgacgcggagatg

acccactcggtggcggtaaacgtctacccggcaacatcgcggatgccaaagttaaccatt

gtggtggtggataccattccggtggagctaaaaagttcctatatggtctggagctggttt

atctatgtgctctcagccaatctgctgttagtgatcccgctgctgtgggtcgccgcctgg

tggagtttacgccccatcgaagccctggcaaaagaagtccgcgaactggaagaacataac

cgcgaattgctcaatccagccacaacgcgagaactgaccagtctggtacgaaacctgaac

cgattgttaaaaagcgaacgcgaacgttacgacaaataccgtacaacgctcaccgacctg

acccatagtctgaaaacgccactggcggtgctgcaaagtacgctgcgttctctgcgtagt

gaaaagatgagcgtcagtgatgctgaaccggtaatgctggagcaaatcagccgcatttca

cagcaaattggctactacctgcatcgtgccagtatgcgcggcgggacattgctcagccgc

gagctgcatccggtcgccccactgctggacaatctcacctcggcgctgaacaaagtgtat

caacgcaaaggggtcaatatctctctcgatatttcgccagagatcagctttgtcggtgag

cagaacgattttgtcgaggtgatgggcaatgtgctggataatgcctgtaaatattgcctc

gagtttgtcgaaatttctgcaaggcaaaccgacgagcatctctatattgtggtcgaggat

gatggacccggtattccattaagcaagcgagaggtcattttcgaccgtggtcaacgggtt

gatactttacgccctgggcaaggtgtggggctggcggtagcccgcgaaatcaccgagcaa

tatgagggtaaaatcgtcgccggagagagcatgctgggcggtgcgcggatggaggtgatt

tttggtcgccagcattctgcgccgaaagatgaataaatgcgcgtactggttgttgaagac

aatgcgttgttacgtcaccaccttaaagttcagattcaggatgctggtcatcaggtcgat

gatgcagaagatgccaaagaagccgattattatctcaatgaacatttaccggatattgcg

attgtcgatctcggattgccagacgaggacggtctgtcactgattcgccgctggcgtagc

aatgatgtttcactgccgattctggtattaaccgcccgtgaaagctggcaggacaaagtc

gaagtattaagtgccggtgctgatgattacgtgactaaaccgtttcatattgaagaggtg

atggcgcgaatgcaggcattaatgcggcgtaatagcggcctggcttcacaggtcatttcg

ctccccccgtttcaggttgatctctctcgccgtgaattatctattaatgacgaagtgatc

aaactgaccgcgttcgaatacaccattatggaaacgttgatacgcaataatggcaaagtg

gtgagcaaagattcgttaatgctccaactctatccggatgcggagctgcgggaaagccat

accattgatgtactgatgggacgtctgcgcaaaaaaattcaggcacaatatccccaagaa

gtgattaccaccgttcgcggccagggctatctgttcgaattgcgctgaatggcattactc

gatttctttctctcgcggaagaaaaacacagccaacattgcaaaagaacggctgcagatt

attgttgctgaacgccgtcgcagcgatgcagaaccgcattatctgccgcagttgcgtaaa

gatattcttgaggtcatttgtaaatacgtacaaattgatcctgagatggtaaccgtacag

cttgagcaaaaagatggcgatatttctattcttgagctgaacgtgaccttaccggaagca

gaagagctgaaataaatggcacgcattattgttgttacttcgggcaaagggggtgttggt

aagacaacctccagcgcggccatcgccactggtttggcccagaagggaaagaaaactgtc

gtgatagattttgatatcggcctgcgtaatctcgacctgattatgggttgcgaacgccgg

gtcgtttacgatttcgtcaacgtcattcagggcgatgcaacgctaaatcaggcgttaatt

aaagataagcgtactgaaaatctctatattctgccggcatcgcaaacacgcgataaagat

gccctcacccgtgaaggggtcgctaaagttcttgatgatctgaaagcgatggattttgaa

tttatcgtttgtgactccccggcagggattgaaaccggtgcgttaatggcactctatttt

gcagacgaagccattattaccaccaacccggaagtctcttcagtacgcgactctgaccgt

attttaggcattctggcgtcaaaatcacgccgcgcagaaaatggcgaagagcctattaaa

gagcacctgctgttaacgcgctataacccaggccgcgtaagcagaggtgacatgctgagc

atggaagatgtgctggagatcctgcgcatcaaactcgtcggcgtgatcccagaggatcaa

tcagtattgcgcgcctctaaccagggtgaaccggtcattctcgacattaacgccgatgcg

ggtaaagcctacgcagataccgtagaacgtctgttgggagaagaacgtcctttccgcttc

attgaagaagagaagaaaggcttcctcaaacgcttgttcggaggataaatgtatcaacat

cacaactggcaaggtgcgctgctggattatccggtgagtaaagtagtctgtgttggcagt

aactatgccaaacatattaaagagatgggcagcgcagtgcccgaagagccagtgctgttt

attaaaccagaaacggcactgtgcgatctgcggcagccattggcgatcccatccgatttc

ggttcagttcatcatgaagtcgaactggcggtgttgattggcgcgacgctgcgtcaggct

acggaagagcatgtccgcaaagccattgccggttacggcgtggcgctcgatctgacgttg

cgtgatgttcagggaaaaatgaagaaagccgggcagccgtgggaaaaggctaaagcgttt

gataactcttgtccgctttccgggtttattcccgcggcggaattcaccggcgatccgcaa

aatacaacgctgggcctgagcgtaaacggcgaacaacgccagcaaggtacgactgcggac

atgatccataaaatcgttccgctgatcgcttatatgagcaagttttttaccctcaaggcc

ggtgacgttgtgctgacaggcacgcctgatggcgtcggcccgttgcaaagcggtgatgag

ctgacagtcactttcgatgggcattctttgacaactcgcgttttgtaaatgttgtttatc

aagcctgcggatctccgcgaaattttgacttttccgctatttagcgatcttgttcagtgt

ggctttccttcaccggccgcagattacgttgaacagcgcatcgatctgaatcaactgttg

atccagcatcccagcgcgacttacttcgtcaaagcaagtggtgattctatgattgatggt

ggaattagtgacggtgatttactgattgtcgatagcgctattaccgccagccatggtgat

attgtcatcgctgctgttgacggcgagtttacggtgaaaaaattgcaactacgcccgacg

gtacagcttattcccatgaacagcgcgtactcgcccattaccatcagtagtgaagatacg

ctggatgtctttggtgtggtgatccacgtcgttaaggcgatgcgctgaatgggattcaaa

tgcggtatcgtcggtttgcccaacgtcgggaaatctaccctgttcaacgcgctgaccaaa

gccggtattgaagcggccaactttccattctgcaccattgagccgaacacaggcgtcgta

ccaatgcctgaccctcgcctggatcaactggctgaaatcgtaaaaccgcagcgtacgctt

cccacgaccatggaatttgtcgatatcgccggtctggtaaaaggcgcatcgaaaggcgaa

ggtctgggtaaccagttcctgaccaacatccgtgaaaccgaagcgatcggtcacgttgtt

cgctgctttgaaaatgacaacatcattcacgtttccggcaaagttaacccggctgacgat

attgaagttatcaataccgaactggcgctggcagacctcgacacctgcgaacgtgcgatt

catcgcgtacagaagaaagccaaaggtggcgataaagacgcgaaagctgagctggcggtc

ctggaaaaatgcctgccccagctggaaaacgcaggtatgctgcgcgcgctggatttaagc

gctgaagagaaagcggctattcgttacctgagcttcctgacgctaaaaccaacaatgtac

atcgccaacgtcaacgaagacggttttgaaaacaacccatatcttgaccaggtgcgtgaa

atcgcggcgaaagaaggttctgttgtggttccggtttgtgctgctgttgaagcagacatt

gccgaactggacgacgaagaacgtgacgagtttatgcaggagcttgggcttgaagagccg

ggcctgaaccgtgtgatccgtgccggttataagctgctgaacctgcaaacttacttcacc

gctggggtgaaagaagtgcgtgcatggaccattccggttggagcaaccgcgccgcaggca

gcgggaaaaatccatactgattttgaaaaaggctttatccgtgcacaaaccatctcgttt

gaagatttcatcacttacaaaggtgaacaaggcgcgaaagaagcaggcaaaatgcgtgca

gaaggtaaagattacatcgttaaagatggcgatgtgatgaacttccttttcaacgtctaa

atgaaacgcaaaaacgcttcgttactcggtaacgtgctcatggggttgggtctggtggta

atggtggttggcgtggggtattcaatcctcaaccagttaccacagtttaatatgccccag

tatttcgcacatggtgcagtgctaagtattttcgtcggtgccattctctggctggcgggt

gcccgtgttggcgggcatgaacaggtgtgcgaccgttactggtgggttcgccactatgac

aaacgttgccgccgtagcgataatcgccgtcatagctaaatgcggacacagtggccctct

ccggcaaaacttaatctgtttttatacattaccggtcagcgtgcggatggttaccacacg

ctgcaaacgctgtttcagtttcttgattacggcgacaccatcagcattgagcttcgtgac

gatggggatattcgtctgttaacgcccgttgaaggcgtggaacatgaagataacctgatc

gttcgcgcagcgcggttattgatgaaaactgcggcagacagcgggcgtcttccgacggga

agcggtgcgaatatcagcattgacaagcgtttgccgatgggcggcggtctcggcggtggt

tcatccaatgccgcgacggtcctggtggcattaaatcatctctggcaatgcggactaagc

atggatgagctggcggaaatggggctgacgctgggcgcagatgttcctgtctttgttcgg

gggcatgccgcgtttgccgaaggcgttggtgaaatactaacgccggtggctccgccagag

aagtggtatctggtggcgcaccctggtgtaagtattccgactccggtgatttttaaagat

cctgaactcccgcgcaatacgccaaaaaggtcaatagaaacgttgctaaaatgtgaattc

agcaatgattgcgaggttatcgcaagaaaacgttttcgcgaggttgatgcggtgctttcc

tggctgttagaatacgccccgtcgcgcctgactgggacaggggcctgtgtctttgctgaa

tttgatacagagtctgaagcccgccaggtgctagagcaagccccggaatggctcaatggc

tttgtggcgaaaggcgctaatctttccccattgcacagagccatgctttaaatggaatat

caatactggttacgtgaagcaataaaccaacttcaggcgagcgaaagcccgcggcgtgat

gctgaaatcctgctggagcatgttaccggcagagggcgtacttttattctcgcctttggt

gagacgcagctgactgacgaacaatgtcagcaacttgatgcgctactgacacgtcgtcgc

gatggtgaacccattgctcatttaaccggggtgcgagaattctggtcgttgccgttattt

gtttcgccagcgaccttaattccgcgcccggatacggagtgtctggtggagcaggcactg

gcgcggttgcctgaacaaccttgccgtattctcgatctcgggacgggtaccggggcgatt

gcgcttgcgctggctagcgagcgcccggactgcgaaattatcgctgtagatcgtatgcct

gatgctgtctccctggcacaacgtaatgcccagcatctggcgatcaaaaatatccacatt

ctgcaaagcgactggtttagcgcgctagccgggcagcagtttgcgatgattgtcagcaat

ccgccgtatattgacgagcaggacccacatcttcaacaaggcgatgtccgctttgagccg

ctcactgcgctggttgcggcagacagtggaatggcagacatcgtgcatatcatcgaacag

tcgcgtaacgcgctggtatccggcggctttctgcttctggaacatggctggcagcagggc

gaagcggtgcgacaggcatttatcctcgcggggtatcatgacgtcgaaacctgccgtgac

tatggtgataacgagcgtgtaacgctcggccgctattatcaatgaatgacaagtttttct

acactgcttagtgttcatcttattagtatcgcgctttctgttgggctattaaccttacgt

ttctggctacgttatcagaagcatcctcaggcatttgcgcgctggacgcgcattgtgccg

ccggttgtcgatacggtgttattgttaagcggcattgcgttgatggctaaagcgcacatc

ctgccattttccgggcaggcacagtggctgactgaaaagctgtttggagttatcatttat

atcgttttgggttttattgcactcgattatcgtcgtatgcacagtcagcaggcgcgcatt

attgccttcccgctggcgttggtggtgctgtacatcatcattaaactcgccaccacaaaa

gtaccgttactggggtaaatgcaaaaaatcgtgatcgttgccaatggcgcaccttacggg

agcgaatccttgtttaacagcttgcggctggccattgcgttacgagagcaggagagcaat

ctggatctgcgtctgttcctgatgtctgatgcggtcacagccgggttgcgcgggcaaaaa

ccaggggaaggctacaacattcagcaaatgctggagatccttaccgctcagaatgtaccg

gtgaaattgtgcaaaacctgtaccgacgggcgcgggattagtacacttcctctgattgat

ggggtggaaatcggtactcttgtggaactggcgcaatggacgctgtcagccgataaagtg

ctcacattttaaatgcattcactccaacgtaaagttctgcgtactatttgtccggaccaa

aaaggtctgatcgcacgtattaccaatatttgctacaagcacgagttaaatatcgtacag

aacaatgaatttgttgatcaccgtaccgggcgcttttttatgcgcacggaactggaaggg

atttttaatgattccaccctgctggcggatctctatagcgcattgccagaaggctccgtg

cgtgagctgaatcctgccggtcgtcgccggatagtgattctggtcactaaagaagcgcat

tgccttggcgatttgttgatgaaagccaattatggcggcctggatgtcgaaatcgcggca

gtgattggtaaccacgatactttacgttctctggttgagcgttttgatataccgtttgag

ctggtaagccatgaagggttaagccgcaacgagcacgatcaaaagatggcggatgccatt

gatgcttatcaacctgactacgtggtgctggcgaagtatatgcgggtattaacaccggaa

tttgtgtcacgcttcccgaataagatcatcaatattcaccattccttcctgccagcgttt

atcggcgcacgtccttatcaccaggcctatgaacgtggcgtgaagattattggcgcaacc

gctcactatgtgaatgacaatctggacgaaggcccaatcatcatgcaggacgttattcat

gtcgatcatacctacacagctgaagatatgatgcgcgcaggtcgtgacgtcgagaaaaac

gtcttaagtcgcgcgctctacaaagtactggcgcagcgcgtctttgtttacggtaatcgg

acgattattctttaaatgacgcagccattggtcggaaaacagattctcattgttgaagat

gagcaggtatttcgctcgcttctggattcgtggttttcctcattgggagcgacaacggta

ctggcggctgatggggtggatgcccttgagttgctgggaggtttcactccagacctgatg

atatgtgatatcgcgatgccacgaatgaacgggcttaaactgctggagcatatacgtaac

agaggcgaccagaccccagttctggtgatatctgccactgaaaatatggcagatattgcc

aaagcgttacgtctgggcgttgaagatgttttgctgaaaccagttaaagatctgaatcgc

ttgcgcgagatggtttttgcctgtctctatcccagcatgtttaattcgcgcgttgaggaa

gaggaaaggctttttcgcgactgggatgcaatggttgaaaaccctgccgcagcggcgaaa

ttattacaggaactacaaccgccggttcagcaggtgatttcccattgccgggttaattat

cgtcaattggttgccgcggacaaacccggcctggtgcttgatattgccgcactttcggaa

aacgatctggcattttattgccttgatgtcacccgagctggacataatggcgtacttgct

gccttgttattacgcgcattgtttaacggattattacaggaacagcttgcacaccaaaat

caacggttgccagagttgggcgcgttattgaagcaggtaaaccatttactccgtcaggcc

aatctgccggggcagtttccgctattagttggctattatcatcgcgaactgaaaaatctc

attctggtttctgcgggtctgaatgcgacgttaaataccggcgaacaccaggtgcaaatc

agtaatggtgttccgttaggcactttaggtaacgcttatttgaatcaattgagccagcga

tgcgatgcctggcaatgccaaatatggggaaccggtggtcggctgcgcttgatgttgtct

gcagaatgaatgagcgaagcacttaaaattctgaacaacatccgtactcttcgtgcgcag

gcaagagaatgtacacttgaaacgctggaagaaatgctggaaaaattagaagttgttgtt

aacgaacgtcgcgaagaagaaagcgcggctgctgctgaagttgaagagcgcactcgtaaa

ctgcagcaatatcgcgaaatgctgatcgctgacggtattgacccgaacgagctgctgaat

agccttgccgccgttaaatctggcaccaaagctaaacgtgctcagcgtccggcaaaatat

agctacgttgacgaaaacggcgaaactaaaacctggactggccagggccgtactccagct

gtaatcaaaaaagcgatggatgagcaaggtaaatccctcgacgatttcctgatcaagcaa

taaatgttaaaatttattctacgtcgctgtctggaagcgattccgacgctatttattctt

attactatttcgttctttatgatgcgcctcgcgccgggaagcccttttaccggcgaacgt

actttaccgccagaagtgatggccaatatcgaagcgaaatatcatcttaatgatccaatc

atgacacagtatttcagctacctgaaacaactggcgcacggtgatttcggtccatcgttt

aaatataaagattattcggtcaatgacctggtggcatccagttttcccgtttctgccaaa

ctgggagccgcagcatttttccttgcggtaatactgggtgttagtgctggcgttattgcc

gcattaaaacaaaacaccaaatgggactataccgtgatggggctggcaatgaccggggtt

gttatccccagttttgtggttgcgccattattagtcatgatatttgcgatcattttgcat

tggctgccgggcggtggctggaatggtggggcgcttaaattcatgatattgccgatggtg

gcgttgtcactcgcttatatcgccagtattgcgcgtattacccgtggctctatgattgaa

gtattacactccaactttattcgtactgcccgggcgaaagggttacctatgcggcggatc

attttacgccacgcattaaaacctgctctgttacccgtgctctcctatatgggccctgca

tttgtcggcattattaccggttctatggtcatcgaaaccatttatggtttgccggggatt

gggcaattgttcgttaatggtgcattgaaccgtgactattccttagtgttaagcctgacc

atcctggttggtgctttaaccattttgtttaatgccattgtcgatgtgctatatgcggtt

atcgacccgaaaatccgttactgaatgatgttaagtaagaaaaacagcgagacgctggaa

aatttcagtgaaaagctggaggtcgaagggcgcagcttgtggcaggacgcacgtcgacgt

tttatgcataaccgtgcggcggttgccagtctgatagtgctggtgctgatcacgttattt

gtaatcctggcaccgatgctttcgcagtttgcctatgacgatactgactgggcgatgatg

tccagcgccccggatatggagtccggtcactactttggtactgactcatccggtcgcgac

ctgcttgtgcgcgttgcgattggcgggcgtatctcactcatggtcggtgttgctgcggca

ctggtggcagtggtcgtggggacactttacggttcgctttccggttatctgggcggtaaa

gtggattcggtaatgatgcgtctgctggaaatcctcaactctttcccattcatgttcttc

gtcattttgctggtgacctttttcggccaaaacatcctgctgattttcgtggcgattggc

atggtttcctggctggatatggctcgtattgtgcgtgggcaaaccctgagtctgaagcgc

aaagagtttattgaggcggcacaagttggcggtgtatcgacgccgggcattgttattcgc

cacattgtgccgaacgtactcggtgtggtggtggtctacgcatcgctactggtgcccagc

atgatcctctttgaatctttccttagcttcctggggttgggtacgcaagagccgttaagc

agctggggggcattgctgagtgatggcgcgaactcgatggaagtctctccatggttactg

ttgttcccagcgggattcctcgtggtgacgctgttttgtttcaactttatcggcgatggc

ttgcgtgatgccctcgacccgaaagatcgttaaatgtctacaacacataacgtccctcag

ggcgatcttgttttacgtactttagccatgcccgccgataccaatgccaatggtgacatc

tttggtggttggttaatgtcacaaatggatattggcggcgctattctggcaaaagaaatt

gcccacggtcgcgtagtgactgtgcgggttgaaggaatgactttcttacggccggttgcg

gtcggcgatgtggtgtgctgctatgcacgctgtgtccagaaagggacgacatcggtcagc

attaatattgaagtgtgggtgaaaaaagtagcgtctgaaccaatcgggcaacgctataaa

gcgacagaagcattatttaagtatgtcgcggttgatcctgaaggaaaacctcgcgcctta

cctgttgagtaaatgaagcagtttcttgattttttaccgctggttgtctttttcgcgttt

tacaagatttatgacatctatgcggctactgcggcgctgatcgtcgccacggcgattgtg

cttatatatagctgggttcgctttcgtaaggttgagaagatggccctgatcacttttgtt

ctggtggtcgtcttcggtggcttgacgctgttcttccacaatgatgagtttattaaatgg

aaggttacagtcatttatgccctgtttgcgggtgccctgttagtcagccaatgggtgatg

aaaaagccgctaattcagcggatgctgggtaaagaactcacgctgccgcaaccggtatgg

tcgaagctgaatctggcctgggctgttttctttatcctttgcggtctggcaaacatctac

atcgcattctggctgccgcaaaatatttgggtcaactttaaagtctttggcctgaccgcc

cttaccttaatcttcacattgttaagcggtatctatatctaccgccacatgccgcaggaa

gataaatcctaaatgtctatcacggcgcagtccgtataccgtgacaccggaaatttcttc

cgtaatcaatttatgaccattctgttggtatcgttgctatgtgcgtttatcacagtggtg

ttagggcatgttttctcacccagtgatgcacagcttgcgcagctcaatgacggcgtgccc

gttagcggcagtagtgggttgttcgacctggttcagaatatgtcaccggaacagcaacaa

attttgctgcaggcttcagcggcgtccactttttcaggattaatcggtaacgccattctc

gccggaggcgtaatattaattatccagctggtgtctgcgggtcagagagtcagtgcgctg

cgagctattggtgccagtgcgccgatattgccaaagttatttattctgatttttctgact

acccttttagtacagattggcatcatgctggtggtcgttccgggaattatcatggccatt

ttactggccctggcaccggtgatgttggttcaggacaaaatgggcatttttgcctcgatg

cgtagcagtatgcggctgacttgggcgaatatgcgtctggtggcacccgcagtactgagc

tggttgctggcaaaaacactgttgctgctttttgcctcttcttttgccgcattaaccccg

gaaattggtgccgtactggcgaacaccttgagcaacctgatttcagccgtattgctcatc

tatctgttccgtctgtatatgttgattcgccaataagtgaaatatttactcattttctta

ctggtgttagcgatcttcgtgatttcggtcacgttgggtgcgcagaacgatcaacaggtg

acgtttaattatctgttagcgcaaggggagtaccgtatttccacattgctggcggtattg

tttgctgcggggtttgctatcggttggttgatttgtggcctgttctggctgcgagttcgt

gtttccctggcgcgcgctgaacgtaaaataaagcgactggaaaaccagctttcacccgcg

actgacgtggctgtagtgcagcactcgtcagcggcgaaggaataaatgaattcccgacaa

caaactattctacagatggtcattgaccagggtcaggttagcgtaaccgatctggcaaaa

gccactggagtttctgaagttaccattcgccaggatctcaacaccctcgaaaaactgagt

tacctccgccgtgcacatggctttgcagtttcgcttgatagtgatgacgtcgaaacccgt

atgatgagcaattatacgctgaagcgtgaactcgccgagtttgccgcgtcactggttcaa

ccgggcgaaaccatctttatcgaaaatggcagcagcaatgccctgctcgctcggactctg

ggcgagcagaagaaaaatgtcactatcatcacggtcagcagctacatcgcgcatttgctg

aaagacgcgccttgtgaagttattttgctcggtggcgtgtaccagaaaaaaagcgaaagt

atggttggccctttgacacgccagtgcatccaacaggtgcatttcagcaaagcatttatt

ggtattgatggctggcaacctgaaactggatttaccggtcgcgatatgatgcgtaccgat

gtggtcaatgccgtgctggaaaaagagtgcgaagcgatagtcctgactgacagctcgaaa

tttggtgctgtacattcatactccatcggtcccgttgagcgattcaatcgcgtgattacc

gattcgaaaatacgcgccagcgatctgatgcatcttgagcacagtaaactcaccgttcac

gtcgttgacatttaaatgggtattttttctcgctttgccgacatcgtgaatgccaacatc

aacgctctgttagagaaagcggaagatccacagaaactggtgcgtctgatgatccaggag

atggaagatacattggttgaagtacgttctacttcggcgcgtgcgctggcagaaaagaaa

cagctgactcgccgtattgaacaagcgtcggcgcgtgaagttgaatggcaggaaaaagcc

gaactggcgctgctgaaagagagagaggatttggcgcgtgcggcgttaattgaaaaacag

aaactgaccgatctgattaagtccctggaacatgaagtgacgctggtggacgatacgctg

gcacgcatgaagaaagagatcggtgagctggaaaacaaattgagcgaaacacgcgctcgc

cagcaggcattgatgttacgccatcaggcggcaaactcgtcgcgcgatgtgcgtcgtcag

ctggacagtggcaaactggatgaagcaatggctcgtttcgaatctttcgaacgtcgaatt

gaccagatggaagcggaagcagaaagccacagcttcggtaaacaaaaatcgctggacgat

cagtttgccgaactgaaagccgatgatgcaataagcgagcaactggcacaattaaaagcc

aaaatgaagcaagacaatcaataaatgtcacaaaccgttcatttccagggcaacccggtt

acagtcgccaattccatcccacaggcgggtagcaaagcgcagacttttactctcgtggca

aaagatctgtctgacgtcaccctcggtcagtttgcgggtaaacgcaaagtgctgaacatt

ttcccgagtattgataccggtgtttgcgccgcatcagtacgtaagtttaaccaactggca

accgagatcgacaacaccgttgtgctgtgtatttctgccgatctgccgttcgcccagtct

cgtttctgcggcgcagaaggtctgaacaacgttatcaccctctccactttccgtaacgct

gaattcctgcaagcctacggtgtggcaattgctgatggcccactgaaaggtctggcagcg

cgtgccgttgtggttattgatgaaaatgacaatgtgattttcagccagctggtggatgaa

atcaccaccgagccggattacgaagcagctctggctgtactgaaagcataagtggaagcg

attaagggatcggacgttaatgtcccggatgcagtatttgcctggatgctggatggtaga

ggcggcgttaaaccgctggaaaatacagatgtgattgatgaagcgcatccctgctggctc

caccttaattatgtacaccatgatagcgcccaatggctggcgacaacaccgctgcttccc

aataacgtacgtgatgcgctggcaggtgagagcacacggccccgagtcagccgtctcggt

gaaggcacgctgattacattgcgctgtataaacggcagcaccgatgaacgccccgatcaa

ctggtcgccatgcgtgtatatatggacgggcggttaattgtttcgacccgacaacgcaaa

gtgttggcgctggacgatgtggtgagcgatctggaagagggcacgggtccgaccgattgc

ggagggtggctggtggatgtgtgcgatgcgttgaccgatcattccagcgaatttatcgag

cagctgcacgataaaattatcgaccttgaagataatctccttgatcagcaaattccgccg

cgtggattcctggctctgctgcgcaaacaattaattgtgatgcgtcgctatatggcaccg

caacgtgatgtctatgctcgtcttgccagtgaacgtttgccgtggatgagcgatgaccaa

cgccgtcggatgcaggatattgccgatcgccttgggcgcggccttgacgaaatcgacgcc

tgtatagcacggactggtgtgatggcggatgaaatcgctcaggtgatgcaggaaaattta

gctcgtcgtacctatacaatgtcattgatggcaatggtctttttacccagtacctttctg

acagggttatttggcgtcaaccttggtgggatccctggcggcgggtggcaattcggattt

tcaattttttgtattctgttagttgttcttattggtggtgttgctttatggttgcatcgt

agtaaatggttgtaaatgaaactcgccgtttatagcacaaaacagtacgacaagaagtac

ctgcaacaggtgaacgagtcctttggctttgagctggaattttttgactttctgctgacg

gaaaaaaccgctaaaactgccaatggctgcgaagcggtatgtattttcgtaaacgatgac

ggcagccgcccggtgctggaagagctgaaaaagcacggcgttaaatatatcgccctgcgc

tgtgccggtttcaataacgtcgaccttgacgcggcaaaagaactggggctgaaagtagtc

cgtgttccagcctatgatccagaggccgttgctgaacacgccatcggtatgatgatgacg

ctgaaccgccgtattcaccgcgcgtatcagcgtacccgtgacgctaacttctctctggaa

ggtctgaccggctttactatgtatggcaaaacggcaggcgttatcggtaccggtaaaatc

ggtgtggcgatgctgcgcattctgaaaggttttggtatgcgtctgctggcgttcgatccg

tatccaagtgcagcggcgctggaactcggtgtggagtatgtcgatctgccaaccctgttc

tctgaatcagacgttatctctctgcactgcccgctgacaccggaaaactaccatctgttg

aacgaagccgccttcgatcagatgaaaaatggcgtgatgatcgtcaataccagtcgcggt

gcattgattgattctcaggcagcaattgaagcgctgaaaaatcagaaaattggttcgttg

ggtatggacgtgtatgagaacgaacgcgatctgttctttgaagataaatccaacgacgtg

atccaggatgacgtattccgtcgcctgtctgcctgccacaacgtgctgtttaccgggcac

caggcattcctgacagcagaagctctgaccagtatttctcagactacgctgcaaaactta

agcaatctggaaaaaggtgaaacctgcccgaacgaactggtttaaatgtctggaggatta

gttacagctgcatacattgttgccgcgatcctgtttatcttcagtctggccgggctttcg

aaacatgaaacgtctcgccagggtaacaacttcggtatcgccgggatggcgattgcgcta

atcgccaccatttttggaccggatacgggtaacgttggctggatcttgctggcgatggtc

attggtggggcaattggtatccgtctggcgaagaaagttgaaatgaccgaaatgccagaa

ctggtggcgatcctgcatagcttcgtgggtctggcggcagtgctggttggctttaacagc

tatctgcatcatgacgcgggaatggcaccgattctggtcaatattcacctgacggaagtg

ttcctcggtatcttcatcggggcggtaacgttcacgggttcggtggtggcgttcggcaaa

ctgtgtggcaagatttcgtctaaaccgttgatgctgccaaaccgtcataaaatgaacctg

gcggctctggtcgtttccttcctgctgctgattgtatttgttcgcacagacagcgtcggc

ctgcaagtgctggcattgctgataatgaccgccattgcgctggtattcggctggcattta

gtcgcctccatcggtggtgctgatatgccagtggtggtgtcaatgctgaactcgtactcc

ggctgggcggctgcggctgcgggctttatgctcagcaacgacctgctgattgtgaccggt

gcgctggtcggttcttcgggcgctatcctttcttacattatgtgtaaggcgatgaaccgt

tcctttatcagcgttattgcgggtggtttcggcaccgacggctcttctactggcgatgat

caggaagtgggtgagcaccgcgaaatcaccgcagaagagacagcggaactgctgaaaaac

tcccattcagtgatcattactccggggtacggcatggcagtcgcgcaggcgcaatatcct

gtcgctgaaattaccgagaaactgcgcgctcgtggtatcaacgtgcgtttcggtatccac

ccggttgcggggcgtttgcctggacatatgaacgtattgctggctgaagcaaaagtaccg

tatgacatcgtgctggaaatggacgagatcaacgatgactttgctgataccgataccgta

ctggtgattggtgctaacgatacggttaacccggcggcgcaggatgatccgaagagtcct

attgctggtatgcctgtgctggaagtgtggaaagcgcagaacgtgattgtctttaaacgt

tcgatgaacactggctatgctggtgtgcaaaacccgttgttcttcaaggaaaacacccac

atgctgtttggtgacgccaaagccagcgtggatgcaatcctgaaagctctgtaaatgggt

aaaacccagcccttgccaatattaattactggcggaggtcgtcgcatcggcctcgccctc

gcatggcatttcattaatcaaaagcaaccggtgattgtcagctatcggacacactatcca

gccattgatggactgattaatgcaggtgcgcagtgtattcaggctgatttttcgaccaac

gacggtgtgatggcgtttgccgatgaagtactaaaaagcacccatggtctgcgtgctatt

ttgcataacgccagtgcgtggatggcggaaaaaccgggtgcgccactggccgacgtactg

gcttgcatgatgcagatccacgttaataccccatacctgctcaaccatgcgctggaaaga

ttactgcgtgggcacggacacgccgccagcgatatcattcactttaccgattatgtggtg

gagcgcggtagcgacaaacatattgcgtatgctgcaagcaaagcggcactggataatatg

acccgctcgtttgcccgcaagctggcaccggaagtgaaagtgaattctattgcgccatcg

ctgatcctgtttaatgaacatgatgatgccgaatatcgacaacaggcgctgaataaatca

ctgatgaaaaccgcgcctggcgagaaagaagtgatcgacctggtcgattacttacttacc

agctgctttgtcaccggacgcagtttcccacttgatggcggtcgtcatctgcgttaaatg

gctaccgccaaaaaaataaccattcatgatgttgcgctggctgcgggcgtgtcggtaagt

accgtttcgctggtgcttagtggcaaagggcgaatctctaccgccacaggagaacgcgtt

aacgccgccattgaagagctgggatttgtgcgcaatcgccaggcgtcggcgctgcgcggc

gggcaaagcggcgtcattggtttgatcgtccgtgatttatctgcgccgttttacgccgaa

ttgacggccggattgacggaagctctggaagcgcagggacggatggtttttttgcttcac

ggcggtaaagacggcgagcagctggcacagcggttttcactgttactgaatcagggtgtc

gatggtgtggtaattgccggggctgcaggaagcagcgatgacctgcgacggatggcagaa

gaaaaagctatcccggtgattttcgcttcccgtgccagttatcttgatgatgttgatacg

gttcgcccggacaacatgcaggctgcacagttgttgacggagcatctcattcgcaatggg

catcagcggatcgcctggctgggagggcaaagttcctcattaacccgtgcagaacgggtg

gggggctattgtgcaactctactaaaatttggccttccgtttcacagcgattgggtgttg

gagtgcacttccagccagaagcaagccgcggaagctatcacggcgcttttacgtcataac

ccgaccatcagtgccgtggtttgctataacgaaactattgcgatgggggcatggtttggt

ttgctgaaagcagggcggcaaagcggggaaagcggagtcgatcgttactttgagcaacag

gtttcgctggcggcatttaccgatgcgacaccaaccacacttgatgatatacccgttacc

tgggccagcacgcctgcgcgggaacttggtaccacacttgcggatcgcatgatgcaaaaa

atcacccatgaagagacgcattcacgcaatcttattattcccgcccggctcattgcggcg

aaataaatgattgataccaccctgccattaactgatatccatcgccaccttgatggcaac

attcgtccccagaccattcttgaacttggccgccagtataatatctcgcttcctgcacaa

tccctggaaacattgattccccacgttcaggtcattgccaacgaacccgatctggtgagc

tttctgaccaaacttgactggggcgttaaagttctcgcctctcttgatgcctgccgccgc

gtggcatttgaaaacattgaagatgcagcccgtcacggcctgcactatgtcgagctgcgt

ttttcaccaggctacatggcaatggcacataagctgcctgtagcgggtgttgtcgaagcg

gtgatcgatggcgtacgtgaaggttgccgcacctttggtgtgcaggcgaagcttatcggc

attatgagccgaaccttcggcgaagccgcctgtcagcaagagctggaggcctttttagcc

caccgtgaccagattaccgcacttgatttagccggtgatgaacttggtttcccgggaagt

ctgttcctttctcacttcaaccttgcgcgtgatgcgggctggcatattaccgtccatgca

ggcgaagccgctgggccggaaagcatctggcaggcgattcgtgaactgggtgcggagcgt

attggacatggcgtaaaagccattgaagatcgggtgctgatggattttctcgccgagcaa

caaattggtattgaatcctgtctgacctccaatattcagaccagcaccgtagcagagctg

gctgcacatccgctgaaaacgttccttgagcatggcattcgtgccagcattaacactgac

gatcccggcgtacagggagtggatatcattcacgaatataccgttgccgcgccagctgct

gggttatcccgcgagcaaatccgccaggcacagattaatggtcttgaaatggctttcctc

agcgcagaggaaaaacgcgcactgcgagaaaaagtcgctgcgaagtaaatgactgactac

ctgttactgtttgtcggaactgtactggtcaataactttgtactggtcaagtttctcggt

ctctgtccgtttatgggggtttccaaaaagctggaaaccgcgatgggcatggggctggca

acaacgtttgtgatgacgctggcgtctatttgcgcctggcttatcgatacgtggattttg

atcccacttaatctgatttacctgcgcaccctggcatttattctggtgattgctgtggtc

gtgcagttcaccgagatggtggtgcgcaaaaccagcccggtgctttaccgtttgctgggg

atttttttgccgcttatcaccaccaactgtgccgtgctcggcgtggcgttgctgaatatc

aatctcgggcacaatttcttgcagtcggcgctgtacggtttttccgccgctgtcggtttt

tcgctggtgatggtgctcttcgcagccatccgcgaacgccttgctgtggctgatgtcccg

gctccttttcgcggtaatgccattgcgttaattaccgcaggtcttatgtctctggccttt

atgggctttagtggtttggtgaagttgtaaatgaatgctatctggattgccgttgccgcc

gtgagcctgctgggcctggcgtttggcgccattctgggttatgcctcccgccgttttgcg

gtggaagacgatccggtcgttgaaaaaattgacgaaatcttaccgcagagccagtgtggt

cagtgcggttatcccggctgtcgcccctacgcggaagccatcagctgtaacggtgaaaaa

atcaaccgttgcgcccctggtggcgaagctgtgatgctaaaaattgccgagttgcttaat

gtcgaaccgcagccgctggatggcgaagcgcaagagctaacgcctgcgcggatggtggcg

gttattgatgaaaataactgtattggctgcactaaatgtattcaggcgtgtccggtagac

gccatcgttggcgctacccgagccatgcatacggtaatgagtgaactctgtacgggctgc

aatttatgtgttgatccgtgcccgacgcactgcatctcgttgcaaccggtcgcagaaaca

cctgactcctggaaatgggatctgaacaccattcccgtgcgtatcattcccgtggaacac

catgcttaaatggtattcagaatagctagctccccttatacccataaccagcgccagaca

tcgcgcattatgctgttggtgttgctcgcagccgtgccaggaatcgcagcgcaactgtgg

ttttttggttggggtactctcgttcagatcctgttggcgtcggtcagtgctctgttagcc

gaagctctcgtactcaaactacgcaaacagtcggtagccgccacgttgaaagataactca

gcattgctgacaggcttattgctggcggtaagtattccccccctcgcgccatggtggatg

gtcgtgctgggtacggtgtttgcggtgattatcgctaaacagttatatggcggtctggga

caaaacccgtttaatccggcaatgattggttatgtggtcttactgatctccttcccggtg

cagatgaccagctggttaccgccacatgaaattgcggtcaacctccctggttttatcgac

gccatacaggttattttcagcgggcataccgccagtggtggtgatatgaacacactacgc

ttaggtattgatggcattagtcaggcgacaccgctggatacatttaaaacctctgtccgt

gccggtcattcggttgaacagattatgcaatatccgatctacagcggtattctggcgggc

gctggttggcaatgggtaaatctcgcctggctggctggcggcgtgtggttgctatggcag

aaagcgattcgctggcatattcccctcagcttcttagtaacgctgacgttatgcgcaacg

ttgggctggttgttctcaccagaaacactggcagcaccgcaaattcatctgctgtctgga

gcgaccatgctcggcgcattctttattttgactgacccggttaccgcttctacgaccaat

cgtggtcgtcttattttcggcgcgcttgcgggcttattagtctggttgatccgcagtttc

ggcggctatcctgacggcgtggcttttgccgtcctgctggcgaacatcacggttcctctg

atcgattactacacgcgtccgcgcgtctacggccatcgcaaagggtaaatgctgaaaact

atccgaaaacacggcattacgttggcgctatttgcagcgggttcaacagggttaactgcg

gccatcaaccagatgaccaaaacgacgattgctgaacaggccagtctgcaacaaaaggct

ttatttgatcaggtgctgccagccgaacgctataacaatgcgctggcacagagttgctat

ctggtaactgcgccagagttaggtaaaggtgagcatcgggtttacatcgccaaacaggat

gacaaaccggtagccgccgttctggaagcaaccgcgccagatggctattccggtgcgatt

cagctgctggtgggagccgattttaacggcacggtacttggcacgcgcgtgacagagcac

cacgaaacgccagggcttggcgataaaatcgaactgcgcctttctgactggatcacccat

tttgcgggtaaaaaaatcagtggtgcggatgatgcgcactgggcggtgaagaaagatggt

ggtgatttcgaccagttcaccggcgcaacgattactccccgcgcggtggttaatgcggta

aaacgcgccggattgtacgctcagacgttaccggcacaactttctcaacttcctgcctgt

ggagaataagtgagcgaaattaaagacgttattgttcaggggttgtggaaaaacaactct

gcgctggtccagttgctcggcctttgtcctctgttggcggtcacgtccactgccactaac

gctctgggtttaggacttgcgactacgctggtactgacgctgaccaacctgaccatttcg

acgctgcgtcactggacgccagccgagatccgcattcccatttacgtgatgatcatcgcc

tcggtggtcagcgctgtacagatgctgatcaacgcctacgcctttggcctgtatcaatca

ttagggatttttattccgctgattgtcactaactgtatcgttgtgggccgcgctgaagcc

ttcgccgccaaaaaaggtccggcgctttcggcactggacggcttttcaattggtatgggc

gcaacctgcgccatgttcgtgctgggttcactacgcgaaattatcggcaatggcacattg

tttgacggtgcagatgcgctgttaggtagctgggcaaaagtattacgcgtggagattttc

cacaccgactcccctttcctgctggcgatgctgccaccaggtgcatttattggcctggga

ctgatgctggcaggaaaatacctgattgatgaaagaatgaaaaagcgccgtactgaagca

gctgccgaacgtgcattgccaaacggtgaaacagggaatgtctgaatgaacaaagcaaaa

cgcctggagatcctcactcgcctgcgtgagaacaatcctcatcccaccaccgagcttaat

ttcagttcgccttttgaattgctgattgccgtactgctttccgctcaggcgaccgatgtc

agtgttaataaggcgacggcgaaactctacccggtggcgaatacgcctgcagcgatgctt

gaactgggcgttgaaggggtgaaaacctatatcaaaacgattgggctttataacagcaaa

gcagaaaatatcatcaaaacctgccgtatcttactggagcagcataatggcgaggttccg

gaagatcgtgctgcgcttgaagccctgcccggcgtaggtcgtaaaacagccaacgtcgta

ttaaacactgcattcggctggccgactattgctgtcgacacgcacattttccgcgtttgt

aatcgtactcaatttgcgccggggaaaaacgtcgaacaggtagaagaaaagctactgaaa

gtggttccggcagagtttaaagtcgactgccaccattggttgatcctgcacgggcgttat

acctgcattgcccgcaagccccgctgtggctcttgtattattgaagatctttgtgaatac

aaagagaaagttgacatctgaatgtctgataacgacgaattgcagcaaatcgcgcatctg

cgccgtgaatacaccaaaggcgggttacgccgccgcgatcttcctgccgatccattaacc

ctttttgaacgctggctctctcaggcttgtgaagccaaactggcggaccctaccgcgatg

gtggtcgctaccgtggatgaacatggtcagccttatcagcgcatcgttttactcaaacat

tacgacgaaaaaggcatggtgttttacaccaacctcggcagccgtaaagcacatcaaatc

gaaaataatccgcgcgttagcctgctgttcccgtggcatacccttgagcgccaggtgatg

gtgatcggtaaagcggaacgactttcgactctcgaagtgatgaaatattttcatagccgc

ccgcgtgatagccagattggtgcatgggtttcgaagcagtccagtcgcatttctgcccgc

ggtatccttgaaagtaaattcctggagctgaagcagaagtttcaacagggcgaagtgcca

ttgccgagcttttggggcggttttcgcgtcagccttgaacagattgagttctggcagggg

ggtgagcatcgcctgcatgaccgctttttgtaccagcgtgaaaatgatgcgtggaagatt

gatcgtcttgcaccctgaatgattaaacgcgtattggttgtttcaatggtaggtctgtct

cttgtcggttgtgttaataacgacaccctgtcaggggatgtttataccgcttctgaagcg

aaacaagtacagaatgtcagctatggcaccatcgttaacgtacgtccggtacagattcag

ggcggtgatgattccaacgtgatcggtgcaattggcggtgctgttcttggtggtttcctg

ggaaatactgttggtggcggaaccgggcgttctctggctactgcagcaggcgctgttgca

ggtggcgtagccggtcagggcgtacagagtgcaatgaacaaaacgcagggtgtcgagctg

gaaattcgtaaagacgatggtaataccatcatggtggtacagaaacaaggcaacactcgt

ttctctccgggccaacgtgtcgtactggccagcaatggcagtcaggtgaccgtttctccg

cgctaaatgcgtcttcttcataccatgctgcgcgttggcgatttgcaacgctccatcgat

ttttataccaaagttctgggcatgaaactgctgcgtaccagcgaaaacccggaatacaaa

tactcactggcgtttgttggctacggcccggaaaccgaagaagcggtgattgaactgacc

tacaactggggcgtggataaatacgaactcggcactgcttatggtcacatcgcgcttagc

gtagataatgccgctgaagcgtgcgaaaaaatccgtcaaaacgggggtaacgtgacccgt

gaagcgggtccggtaaaaggcggtactacggttatcgcgtttgtggaagatccggacggt

tacaaaattgagttaatcgaagagaaagacgccggtcgcggtctgggcaactaaatgtcc

gataacgctcaacttaccggtctgtgcgaccgttttcgtggtttttatcctgttgtgatc

gatgttgaaacagctggatttaacgccaaaaccgatgcgctgcttgagattgccgccatc

accctgaaaatggatgaacaaggctggctgatgccggacaccacattacatttccacgtc

gaaccatttgtcggcgcaaatttgcagccagaggccctcgccttcaacggcattgacccg

aacgatcccgatcgcggcgcggtcagcgaatacgaggcgctgcacgaaatttttaaagtt

gtacgtaaaggtattaaagcgagcggctgtaaccgcgccattatggtggcacacaacgcc

aatttcgatcacagctttatgatggccgccgctgaacgcgcctcactgaaacgtaacccg

ttccaccctttcgccacttttgacactgctgcactggccgggctggcactcggacaaacc

gtattgtcaaaggcttgccagaccgctggcatggacttcgacagcacccaggcgcactcc

gcgctgtacgacaccgaacgcactgcggtgctgttttgtgaaatcgtcaaccgctggaaa

cgtctgggaggctggccgctacctgccgccgaagaggtgtaaatgagcaccactatcgaa

aaaatccaacgccagattgctgaaaacccgatcctgctgtacatgaaaggttcaccgaaa

ctgccgagctgcggtttctctgcccaggcagtccaggcgcttgccgcatgtggcgaacgt

tttgcctatgttgatattctgcagaatccggacattcgtgcggaactgccgaaatatgct

aactggccgaccttcccgcaactgtgggttgacggcgagctggtcggcggttgtgatatc

gtgatcgaaatgtatcagcgtggcgaactgcagcagctgatcaaagaaactgccgctaaa

tacaagtctgaagagccggacgcggaataaatgtcattcgaattacctgcactaccatat

gctaaagatgctctggcaccgcatatttctgcggaaactatcgagtatcactacggcaag

catcatcagacttatgtcactaacctgaacaacctgattaaaggtaccgcgtttgaaggt

aaatcactggaagagattattcgcagctctgaaggtggcgtattcaacaacgcagctcag

gtctggaaccatactttctactggaactgcctggcaccgaacgccggtggcgaaccgact

ggaaaagtcgctgaagctatcgccgcatcttttggcagctttgccgatttcaaagctcag

tttactgatgcagcgatcaaaaactttggttctggctggacctggctggtgaaaaacagc

gatggcaaactggctatcgtttcaacctctaacgcgggtactccactgaccaccgatgcg

actccgctgctgaccgttgatgtctgggaacacgcatattacatcgattatcgcaatgca

cgtcctggttatctggagcacttctgggcgctggtgaactgggaattcgtagcgaaaaat

ctcgctgcataaatgcaacctgggaaaagatttttagtctggctggcgggtttgagcgta

ctcggttttctggcaaccgatatgtatctgcctgctttcgccgccatacaggccgacctg

caaacgcctgcgtctgctgtcagtgccagccttagtctgttccttgccggttttgccgca

gcccagcttctgtgggggccgctctccgaccgttatggtcgtaaaccggtattattaatc

ggcctgacaatttttgcgttaggtagtctggggatgctgtgggtagaaaacgccgctatg

ctgctggtattgcgttttgtacaggctgtgggtgtctgcgccgcggcggttatctggcaa

gcgttagtaacggattattatccttcacagaaagttaaccgaatttttgcgaccatcatg

ccgctggtgggtctgtctccggccctggctcctctgttaggaagctggctgctggtccat

ttttcctggcaggcgattttcgccaccctgtttgccattaccgttgtgctgattctgcct

attttctggctcaaacccacgacgaaggcccgtaacaatagtcaggatggtctgaccttt

accgacctgctacgttctaaaacctatcgcggcaacgtgctgatatacgcagcctgttca

gccagtttttttgcatggctgaccggttcaccgttcatccttagtgaaatgggctacagc

ccggcagttattggtttaagttatgtcccgcaaactatcgcgtttctgattggtggttat

ggctgtcgcgccgcgctgcagaaatggcaaggcaagcagttattaccgtggttgctggtg

ctgtttgctgtcagcgtcattgcgacctgggctgcgggcttcattagccatgtgtcgctg

gtcgaaatcctgatcccattctgtgtgatggcgattgctaatggcgcgatctacccgatt

gttgtcgcccaggcgctgcgtcccttcccacacgcaactggtcgcgccgcagcgttgcag

aacactcttcaactgggtctgtgcttcctcgcaagtctggtagtttcctggctgatcagt

atcagcacgccattgctcaccaccaccagcgtgatgttatcaacagtagtgctggtcgcg

ctgggttacatgatgcaacgttgtaaagaagttggctgccagaatcatggcaatgccgaa

gtcgctcatagcgaatcacactgaatggctttattgccggataaagaaaaattgctgcgt

aattttttacgctgcgccaactgggaagagaaatatctctacattattgagctgggccag

cgtctgccagaattacgcgacgaagacagaagcccacaaaatagcattcagggttgccag

agtcaggtgtggattgtcatgcgccagaatgcgcagggaattattgaattgcaaggcgac

agcgatgcggcgattgtaaaagggcttattgcggtcgtatttattctctacgatcagatg

acgccgcaggatattgtcaatttcgatgtgcgtccgtggtttgaaaaaatggcgctcacc

caacatctcaccccatctcgttcacaaggtctggaagcgatgattcgcgcaattcgcgcc

aaagccgctgcacttagctaaatgatatggaaacgaaaaatcaccctggaagcactgaat

gccatgggtgaaggaaacatggtggggttactggatattcgctttgaacatattggtgat

gacacccttgaagcgacaatgccagtagactcgcggacaaagcagcctttcgggttgcta

catggaggtgcatccgtggtactggccgaaagtatcggttccgttgccggttatttatgt

accgaaggtgagcaaaaagtggttggtctggaaatcaatgctaaccacgtccgctcggca

cgagaagggcgggtacgcggcgtatgcaaaccgttgcatctcggttcgcgtcaccaggtc

tggcagattgaaatcttcgatgagaaagggcgtttgtgctgttcgtcacgattgacgacc

gccattttgtgaatggataatgctgttgatcgccacgttttttatatttctgatggtacg

gcaataactgcggaggtattaggacacgcagtaatgtcacaatttcccgtcactatcagc

agcatcacgctgccgtttgtcgaaaatgagagccgtgcacgggcagtgaaggatcagatt

gacgcaatttatcaccagacaggcgtgcgcccgctggtcttctactccatcgtgttgccg

gagattcgcgccatcatcttgcaaagtgaaggcttttgccaggatatcgttcaggcgctg

gttgccccgctacaacaagagatgaaactggatccaacgccgattgctcatcgtacccat

ggccttaaccctaataatctcaataaatatgatgcgcgcattgcggcgattgattacacc

ctcgcccacgatgacggcatttcgttgcgcaatctggatcaggctcaggtgatcctgctc

ggtgtttctcgctgtggtaaaacccccaccagtctgtatctggcaatgcaatttggtatc

cgcgcggcaaactacccctttattgccgacgatatggataatctggtgctacccgcgtcg

ctcaaaccgcttcagcataaattgttcggcctgactatcgacccggaacgtctggcggcg

attcgcgaggaacgtcgggagaacagtcgctatgcctcgcttcgtcagtgcaggatggaa

gtcgcggaagtggaagccttgtaccgtaaaaatcagatcccgtggattaacagtaccaat

tattcggtagaagagattgccaccaagatcctcgatatcatgggccttagtcgccgaatg

tactagatgcgtttctgccttattttgatcacagcactgtttctggccgggtgtagccac

cataaagcaccgccgccaaatgccagactttctgattcgattaccgttattgccggtttg

aacgaccagctacaaagctggcatggcacgccgtatcgttatggtggcatgacgcggcgc

ggtgtggactgttcgggatttgtggttgtgacgatgcgcgatcgtttcgatttgcagctg

ccccgagaaaccaaagaacaagcctctatcggcacgcaaattgataaagacgagttgctg

cctggtgacctggtctttttcaaaacgggttccggacaaaatggtttgcatgtaggtatt

tacgataccaacaaccaatttatccacgcctctaccagcaagggagtgatgcgttcctca

cttgataatgtctattggcagaaaaatttctggcaggcgagacgaatctagatggccagt

ggcgatcttgtccgttatgtcataaccgtcatgttgcatgaggatacattgactgaaatt

aacgagttgaataattacctgactcgcgacggttttttgctcaccatgacggatgatgag

ggaaatatccatgagctggggactaacacttttggacttatcagtacccaaagtgaagaa

gaaattagagaactggtttcggggcttacccaaagtgcaaccggcaaagatcctgaaatc

accatcacgacctgggaggaatggaatagcaacagaaaataaatgtggcaggcaatcagt

cgtcttttgagcgagcagttaggtgaaggcgaaatcgaactgcgtaatgaactgcctggc

ggagaagtccatgccgcatggcatttgcgctatgcaggacatgactttttcgtcaaatgt

gatgaaagggaactgcttcccggttttaccgccgaagccgaccaactggagttactgtcg

cgtagtaaaaccgtcaccgtgcctaaggtttgggcagtaggcgctgaccgtgactacagt

tttctggtgatggattatctcccacctcgtccgctggatgcgcatagcgcatttattctt

ggtcagcaaattgcgcgtttacatcaatggagtgaccaaccacaatttggcctcgatttc

gataacgcgctctccacaactccacagcccaacacctggcaacgtcgctggtcaacgttt

tttgctgaacaacggattggctggcagttggaactggcagcagagaaagggatcgctttc

ggcaatatcgacgccatcgtcgagcatattcagcagcgtctcgcctcacatcaaccgcag

ccttctctgttgcacggcgatttatggtccggcaactgtgcgctgggtccggatggcccg

tacattttcgacccggcctgctactggggtgaccgagagtgcgacctggcgatgttaccg

ctgcatactgaacagccgccacaaatctatgacggctatcagtcagtatccccgctacct

gccgatttccttgaacgtcaaccggtttaccaactctacacgctgttaaatcgtgcaagg

ttatttggcggtcagcatttggttattgctcagcagtcattggatagattattagcagca

tgaatgacgtatcaacaagctggacgcattgctgttttgaaaaggattttgggttgggtg

atttttatacctgcattgatctctacattgatttctttgctgaaatttatgaatacccgg

caggaaaaccaggaaggcattaatgcggtcatgctcgactttactcatgtcatgatcgat

atgatgcaggcgaatacgccttttttgaatctattctggtataactccccgacgcccaat

tttaatggtggcgtgaacgttatgttctgggtgattttcatcctgatttttgtcggactg

gcgttgcaggattccggtgcccggatgagtcgccaggcgcgcttcttgcgggaaggtgtt

gaagatcaactcattctggaaaaagccaaaggggaagaggggctaacgcgtgaacaaatt

gagtcccgtattgttgttccgcaccatactattttcctgcagtttttctcactgtatatc

ctgccggttatttgtattgctgcaggttatgtgttcttttctctgcttgggtttatttaa

atgtcaaccccgcgtcagattcttgctgcaatttttgatatggatggattacttatcgac

tcagaacctttatgggatcgagccgaactggatgtgatggcaagcctgggggtggatatc

tcccgtcgtaacgagctgccggataccttaggtttacgcatcgatatggtggtcgatctt

tggtacgcccggcaaccgtggaatgggccaagccgtcaggaagtagtagaacgggttatt

gcccgtgccatttcactggttgaagaaacacgtccattattaccaggcgtgcgcgaagcc

gttgcgttatgcaaagaacaaggtttattggtgggactggcctccgcgtcaccactacat

atgctggaaaaagtgttgaccatgtttgacttacgcgacagtttcgatgccctcgcctcg

gctgaaaaactgccttacagcaagccgcatccgcaagtatatctcgactgcgcagcaaaa

ctgggcgttgacccactgacctgcgtggcgctggaagactcggtaaatggcatgatcgct

tctaaagcagcccgcatgcgttccatcgtcgttcctgcgccagaagcgcaaaatgatcca

cgttttgtattagcaaacgtcaaactctcatcgctgacagaactcaccgcaaaagacctt

ctcggttaaatgaacaagaatatggcaggaattctgagtgcagcggcggtattaaccatg

ctggcgggttgtacggcttatgatcgtaccaaagaccagtttgtacagcctgtggtgaaa

gacgtcaaaaaaggcatgagccgggcgcaggttgcacaaattgcgggtaaaccttcgtct

gaagtgagcatgatccatgctcgtggtacttgccagacctacatcctgggtcaacgtgat

ggtaaagcagaaacctactttgtcgcgttagatgataccggacatgtcatcaactccggt

tatcagacctgtgctgaatacgacactgatccacaggctgcgaagtaagtgaccaaactc

aaacttctggcacttggagtgcttatcgcaacgtctgcaggcgtagcgcacgctgaaggt

aaattttccctgggcgcaggcgtaggtgtcgttgaacacccatataaagattacgatacc

gatgtttacccagtaccggtaatcaactatgaaggcgataacttctggttccgtggctta

ggtggtggttactacctgtggaatgacgcaacggataaactttcaattaccgcttactgg

tcgccgctttacttcaaagctaaagacagtggcgatcaccaaatgcgtcacctggatgac

cgtaagagcaccatgatggctggtctgtcttatgctcactttacccagtacggttacctg

cgtaccaccctggctggcgataccctggataacagcaacggcatcgtctgggatatggcc

tggttgtatcgttacaccaacggtggcctgaccgtgactccgggtattggtgtgcagtgg

aacagcgaaaaccagaacgaatactattatggcgtatcgcgcaaagagtccgctcgcagc

ggtctgcgtggctataacccgaacgacagctggagcccttacctggagctgagcgccagc

tacaacttcctcggcgactggagtgtttacggtaccgcgcgctacacccgtctgtctgat

gaagttactgacagcccgatggtggataaatcctggactggcctgatttctaccgggatc

acctacaaattctgagtggaataccgtagcctgacgcttgatgattttttatcgcgcttt

caacttttgcgcccacaaattaaccgggaaaccctaaatcatcgtcaggctgccgtgtta

atccccatcgtccgtcgaccgcaaccggggttgttgctgactcagcgttcaattcatctg

cgtaaacacgctggacaagtggcattccctggaggtgcagtcgatgacacggacgcatca

gttatcgccgccgcgctgcgcgaagctgaagaagaggttgctataccgccttccgccgtt

gaagttatcggcgtgctgccgcccgtcgatagcgtaactggctatcaggtaaccccagtg

gtcggcattatcccgcccgatctgccgtatcgcgccagtgaagatgaagtctcggcggtg

tttgaaatgccgctcgcccaggcattacatctgggtcgttatcaccctttagatatctac

cgccgtggcgattcacatcgggtatggctgtcctggtacgaacagtattttgtatgggga

atgaccgcaggcataattcgtgagctggcgctgcaaattggtgtgaaaccctgagtgacc

attgctattgttataggcacacatggttgggctgcagagcagttgcttaaaacggcagaa

atgctgttaggcgagcaggaaaacgtcggctggatcgatttcgttccaggtgaaaatgcc

gaaacgctgattgaaaagtacaacgctcagttggcaaaactcgacaccactaaaggcgtg

ctgtttctcgttgatacatggggaggcagcccgttcaatgctgccagccgcattgtcgtc

gacaaagagcattatgaagtcattgcaggtgttaacattccaatgctcgtggaaacgtta

atggcccgtgatgatgacccaagctttgatgaactggttgcgctggcagtagaaacaggc

cgtgaaggcgtgaaagcactgaaagccaaaccggttgaaaaagccgcgccagcacccgct

gccgcagcaccaaaagcggctccaactccggccaaaccaatgggaccaaacgactacatg

gttattggccttgcgcgtatcgacgaccgtctgattcacggtcaggtcgccacccgctgg

actaaagaaaccaatgtctcccgtattattgttgttagtgatgaagtggctgcggatacc

gttcgtaagacactgctcacccaggttgcacctccgggcgtaacagcacacgtagttgat

gttgccaaaatgattcgcgtctacaacaacccgaaatatgctggcgaacgt--gtaatgc

tgttatttaccaacccaacagatgtagagcgtctcgttgaaggcggcgtgaaaatcacct

ctgttaacgtcggtggtatggcattccgtcagggtaaaacccaggtgaataacgcggttt

cggttgatgaaaaagatatcgaggcgttcaaaaaactgaatgcacgcggtattgagctgg

aagtccgtaaggtttccaccgatccgaaactgaaaatgatggatctgatcagcaaaatcg

ataagtaaatgtcgttttcctgtccactttgccatcagcctctttcgcgtgaaaaaaaca

gctatatctgtccccaacgacatcagtttgatatggcgaaagaagggtatgtcaatctgc

tgcccgttcagcataaacggtcccgtgatccgggcgacagcgcggaaatgatgcaagcac

gccgcgcattcttagatgccggacattatcagccgctgcgtgatgcaattgtcgcccaac

tgcgggaacggcttgatgaaaaggccacggcggtgctggatattggctgtggtgaagggt

attacacacacgcatttgccgatgcgttgcccgaaatcaccacgtttggtctggatgttt

cgaaggtagcgataaaagcggcggcgaaacgctatccgcaggtcactctttgtgtcgctt

ccagccaccgtttgccgttttccgataccagtatggacgccataatacgtatttacgcgc

cgtgtaaagcagaagaattagcacgagtagtgaagcccggcggctgggtcattactgcca

cgccgggaccgcgacatttgatggagctgaaggggctgatttacaatgaagtacatcttc

atgcacctcatgcagaacaactggaaggttttacattacagcagagtgatgagttgtgtt

atccgatgcgtcttcgcggtgatgaagccgtcgcattattgcagatgacgccgtttgcct

ggcgtgcgaagccagaagtctggcaaacactggcagcaaaagaagtgttcgactgccaga

cggactttaatattcacctctggcagcgttcttattaaatgaacgaagttgtaaattcag

gcgtgatgaacattgcgtctttggttgtatcggtggtggttcttcttatcgggctcatct

tgtggttttttattaatcgtgccagttctcggactaacgaacagattgaactgcttgagg

cgttgctggatcagcaaaaacgtcaaaatgcactgttacgtcgtttgtgcgaagcaaacg

aaccagagaaagcagataaaaagaccattgagagtcaaaaatcggttgaagacgaagata

ttattcgcctggtcgccgaacgataaatgatgaaaaaaagtattctggcgtttctgttac

tcaccagttctgcagcggcgctggctgcaccgcaggtgattaccgtcagccgttttgaag

tgggtaaagacaaatgggcgtttaatcgcgaagaggtgatgctgacttgccgaccgggta

atgctttgtatgtcatcaacccaagtaccctcgtgcagtatcctttaaacgatatcgcac

aaaaggaagttgccagtgggaagactaaaacccaacccatttcggtgattcagattgatg

atcctaacaatcccggcgaaaaaatgagtctggcaccgtttatagaacgagctgaaaaac

tctgttaaatgaaaaactggaaaacaagtgcagaatcaatcctcaccaccggcccggttg

taccggttatcgtggtaaaaaaactggaacacgcggtgccgatggcaaaagcgttggttg

ctggtggggtgcgcgttctggaagtgactctgcgtaccgagtgtgcagttgacgctatcc

gtgctatcgccaaagaagtgcctgaagcgattgtgggtgccggtacggtgctgaatccac

agcagctggcagaagtcactgaagcgggtgcacagttcgcaattagcccgggtctgaccg

agccgctgctgaaagctgctaccgaagggactattcctctgattccggggatcagcactg

tttccgaactgatgctgggtatggactacggtttgaaagagttcaaattcttcccggctg

aagctaacggcggcgtgaaagccctgcaggcgatcgcgggtccgttctcccaggtccgtt

tctgcccgacgggtggtatttctccggctaactaccgtgactacctggcgctgaaaagcg

tgctgtgcatcggtggttcctggctggttccggcagatgcgctggaagcgggcgattacg

accgcattactaagctggcgcgtgaagctgtagaaggcgctaagctgtaaatgacaagtc

tggtttccctggaaaatgtctcggtttcttttggccaacgccgcgtcctctctgatgtgt

cgctggaacttaaacctggaaaaattttgactttacttgggccaaatggcgcaggtaagt

cgacactggtacgggtagtgctcgggctggtaacacccgatgaaggggttatcaagcgca

acggaaaactgcgcatcggctatgtaccgcagaagctgtatctcgacaccacgttgccac

tgaccgtaaaccgttttttacgcttacgccctggtacacataaagaagatattttgcctg

cactgaaacgtgtccaggccgggcacctgattaacgcaccgatgcaaaagctctctggtg

gcgaaacgcagcgtgtactgttagcgcgagcattgttaaatcgcccgcaattattagtgg

tggatgaacccactcaaggcgtggatgtaaatggccaggtggcgttatatgaccttattg

accaactgcgtcgtgaactggattgtggcgttttaatggtttctcacgatctgcatctgg

tgatggcaaaaaccgatgaagtgctgtgcctgaatcaccacatttgttgttccggcacac

cggaggttgtttccctgcatccggagtttatttcaatgtttggtcctcgtggtgctgaac

aactgggtatctatcgccatcatcataatcatcgtcacgatttacagggacgaattgttt

tgcgtcggggaaatgatcgctcatgaatgcaatttaatatccctacgttgcttacactgt

tccgtgtcatccttatcccattctttgtattggtcttttatctgcctgtcacctggtcgc

cgtttgccgccgcgctcattttctgcgtcgcggcggtgactgactggttcgatggttttc

tggcacgccgctggaaccagagtacccggtttggtgctttccttgaccctgtagcagata

aagttctcgtggctatcgccatggtgctggtaaccgagcattaccacagctggtgggtga

ccttaccggcggcaacgatgatcgcccgtgaaattattatttctgcgctacgcgaatgga

tggcggagttgggtaaacgcagtagcgtggctgtctcctggattgggaaagtgaaaacca

ctgcccagatggtggcgttggcatggctgctgtggcgtccgaacatttgggttgagtacg

ccggtattgcacttttctttgtggctgcggtactgactctgtggtcaatgttgcaatatt

tgagcgctgcgcgtgcagatttgcttgatcagtgaatgcgtctgtgtgaccgagatattg

aagcctggcttgatgaaggccgtttgtcgatcaacccacgtccgccagtggagcgtatta

acggcgcgacggtggatgtacgcctgggcaataaatttcgtaccttccgtggtcacacgg

cagcgtttatcgatctgagcggtcccaaagatgaagtgagcgccgcgcttgaccgcgtga

tgagcgatgagatcgttctcgacgagggcgaggcgttctatcttcacccaggagagctgg

cgctggcggtgacgctggagtcggtgacgctgccagccgatctggtgggctggctggacg

ggcgttcctcactggcgcgtctggggctgatggtgcacgtcaccgcgcaccgcatcgatc

cgggctggtctggttgcattgtgctggagttctacaactccggtaagctgccgctggcgc

tgcgtccgggcatgttaattggtgcgctgagctttgagccgctttccggcccggcggcgc

gaccttacaaccgccgtgaagatgcgaaatatcgcaaccagcagggcgcggtagccagcc

gaatcgataaagactaaatgaagatgttgcgcgatccgctgttctggctcattgctctgt

ttgtggcgctgattttctggctgccttacagccagccgctgtttgctgccttgttcccac

aactgccacgacccgtttatcagcaagaaagttttgcagctctggcactggctcatttct

ggctggtgggaatttcgagtttgtttgcggtgatcattggcactggtgccggaattgctg

tcactcgcccgtggggcgcggaatttcgcccactggtggaaactattgccgccgttggac

agacttttccgcccgtcgcagtgctggcgatcgccgttccggtgatcggctttggtctgc

aaccagcgattatcgccttgatcctttacggtgtgctgcccgtcctgcaggcgacacttg

ccgggctgggagcgattgatgccagcgtgacagaagttgcgaaaggtatgggaatgagtc

gtggtcagcgactgcgtaaggtcgagctaccgctggcggctccggtgattctggcgggcg

tgcgaacttcggtgattatcaacattggtacggcgacgatcgcctcaacggtaggggcca

gcacgctgggtacgcccatcatcatcgggcttagcggatttaataccgcgtatgtgatcc

agggggcgttactggtggcactggcggcgatcatcgcagaccgcctgtttgaaaggctgg

tgcaggcgcttagccagcacgcaaaataaatgttaaagcgcgtgttcctcagcctgttag

tcctgatcggcttgctgctgttgactgtgctcggcctcgatcgctggatgagctggaaaa

ccgcgccttatatctacgacgaattgcaggatctcccctaccgccaggtcggtgtggtgc

tcggaacagcaaaatattatcgtactggcgtaattaatcagtattatcgctaccgcattc

aaggagcgattaatgcctataacagcggtaaggtaaattatctattactgagcggcgata

acgcattgcaaagttataatgagccgatgaccatgcgcaaagatttaatcgctgctggtg

tcgacccatcagatattgttctcgattacgcaggctttcgtacgctggattccatcgtgc

gtacacgcaaagttttcgatactaatgatttcattattatcacccaacgtttccactgtg

agcgagcattatttattgcgctgcatatggggattcaggctcagtgttatgccgtaccgt

caccgaaagatatgctgtcagtacgtattcgtgaatttgccgcccgtttcggtgcgctgg

ctgacctttatatttttaaacgtgaaccgcgttttttagggccgctggtccctattccgg

ctatgcaccaggtaccggaagatgcgcaggggtatcccgccgtcacacccgaacagttac

ttgaattacaaaagaaacaaggaaagtagatgaataagaaggtgttaaccctgtctgctg

tgatggccagcatgttattcggtgccgctgcacacgctgctgaaactcgcattggtgtaa

caatctataagtacgacgataactttatgtctgtagtgcgcaaggctattgagcaagatg

cgaaagccgcgccagatgttcagctgctgatgaatgattctcagaatgaccagtccaagc

agaacgatcagatcgacgtattgctggcgaaaggggtgaaggcactggcaatcaacctgg

ttgacccggcagctgcgggtacggtgattgagaaagcgcgtgggcaaaacgtgccggtgg

ttttcttcaacaaagaaccgtctcgtaaggcgctggatagctacgacaaagcctactacg

ttggcactgactccaaagagtccggcattattcagggcgatttgattgctaaacactggg

cggcgaatcagggttgggatctgaacaaagacggtcagattcagttcgtactgctgaaag

gtgaaccgggccatccggatgcagaagcacgtaccacttacgtgattaaagaattgaacg

ataaaggcatcaaaactgaacagttacagttagataccgctatgtgggataccgctcagg

cgaaagataagatggacgcctggctgtctggcccgaacgccaacaaaatcgaagtggtta

tcgccaacaacgatgcgatggcaatgggcgcggtagaagcactgaaagcacacaacaagt

ccagcattccggtgtttggcgtcgatgctctgccagaagcgctggcgctggtgaaatccg

gtgcactggcgggcaccgtactgaacgatgctaacaaccaggcgaaagcgacctttgatc

tggcgaaaaacttggccgatggtaaaggtgcggctgatggcaccaactggaaaatcgaca

acaaagtggtccgcgtaccttatgttggcgtagataaagacaacctggctgaatttagca

agaaataaatgccatcactcagtaaagaagcggccctggttcatgaagcgttagttgcgc

gaggactggaaacaccgctgcgcccgcccgtgcatgaaatggataacgaaacgcgcaaaa

gccttattgctggtcatatgaccgaaatcatgcagctgctgaatctcgacctggctgatg

acagtttgatggaaacgccgcatcgcatcgctaaaatgtatgtcgatgaaattttctccg

gtctggattacgccaacttcccgaaaatcaccctcattgaaaacaaaatgaaggtcgatg

aaatggtcaccgtgcgcgatatcactctgaccagtacctgtgaacaccattttgttacca

tcgatggcaaagcgacggtggcttatatcccgaaagattcggtgatcggtctgtcaaaaa

ttaaccgtatcgtgcaattctttgcccagcgtccgcaggtacaggaacgtctgacgcagc

aaattctcattgcgctacaaacgctgctgggcaccaataacgtggctgtctctatcgacg

cggtgcattattgcgtgaaggcgcgtggcatccgcgatgcaaccagtgccacgacaacga

cctctcttggtggattgttcaaatccagtcagaatacgcgccacgagtttctgcgcgctg

tgcgtcatcacaactgaatgagcagacgtgttgctactatcacccttaatccggcttatg

accttgttggtttctgcccggaaattgaacgcggcgaagtgaacctggtgaaaaccaccg

gtctgcatgcggcgggtaaaggcatcaacgtggccaaagtattaaaagacctgggaattg

atgtcaccgttggcggcttcctgggtaaagacaatcaggatggttttcagcaactgttca

gcgagctgggcattgccaaccgtttccaggttgtacaggggcgcacccgaattaacgtta

agctgacggaaaaagacggcgaagtgaccgacttcaacttctcgggttttgaagtcactc

ccgccgactgggaacgctttgtgactgattctctgagctggctcggtcagttcgatatgg

tctgtgtcagcggaagcttaccgtcaggcgtcagcccggaagcgttcaccgactggatga

ctcgcctgcgtagtcagtgcccttgcattatctttgatagtagccgtgaagcgttagtag

caggtttgaaagcggcaccgtggctggtgaaacctaaccgccgcgagctggaaatctggg

caggccgtaaactgcctgaaatgaaagatgtgattgaagctgcgcatgcgctgcgtgaac

aaggtattgcgcatgtcgttatttcactgggtgccgaaggcgcgctttgggttaatgcct

ccggcgaatggatcgccaaaccaccgtcagtcgatgtcgtaagcaccgttggcgcagggg

attctatggttggtggcctgatttatggcttgctaatgcgtgaatccagtgaacacacac

tgcgtctggcgactgctgttgcagccctggcggtaagtcaaagcaatgtgggtattaccg

atcgtccgcagttggccgcaatgatggcgcgcgtcgacttacaaccttttaactgaatgt

tccagttatccgtacaggacatccatccgggcgaaaaggccggagacaaagaagaggcga

ttcgccaggtcgctgcggcgctggtgcaggccggtaatgtagcagaaggctacgtcaatg

gcatgctggcgcgcgaacagcaaacctcaacgttcctcggcaatggtattgctattccac

acggcactaccgacacccgcgatcaggtgctgaaaaccggcgttcaggtatttcagttcc

cggaaggcgtcacctggggtgacggtcaggtagcgtacgtagcgatcggtattgctgcca

gctcggatgagcatctgggcctgctacgccagctgacccacgtactgagcgatgattccg

ttgctgaacaactgaagtcagcaacaacagcagaagaacttcgcgcattgctgatgggcg

aaaagcagagtgagcagttgaagctcgacaacgaaatgctgacgctggatatcgtcgcca

gcgatctgctgactcttcaggcgctgaacgctgcgcgtctgaaagaggcgggggcagttg

acgcctctttcgtcaccaaagccatcaatgaacaaccgctgaatctcggacagggtatct

ggctgagcgatagcgccgaaggcaatctgcgtagcgcgattgcggtaagccgtgcggcaa

atgcttttgatgtggacggcgaaacggcagccatgctggtgagtgtggcgatgaatgacg

atcagcccattgcggttcttaagcgtctcgctgatttgttgctcgacaataaagctgacc

gcttgctgaaagcggatgcggcaacgttgctggcgctgctgaccagcgatgatgcgccga

ccgacgacgtgttaagcgcggagtttgtggtgcgcaatgaacacggcttgcatgctcgtc

caggtaccatgctggtcaataccattaaacaatttaacagtgatattaccgtgacaaatc

ttgatggcaccggcaaaccggcaaacggacgtagtctgatgaaagttgtggcacttggcg

ttaagaaaggtcatcgcctacgctttaccgcccagggtgcagatgctgaacaggcgctga

aagcaatcggcgacgctatcgctgctggtcttggggagggcgcataaatggtcaaatctc

aaccgattttgagatatatcttgcgcgggatccccgcgattgcagtagcggttctgcttt

ctgcatgtagtgcaaataacaccgcaaagaatatgcatcctgagacacgtgcagtgggta

gtgaaacatcatcactgcaagcttctcaggatgaatttgaaaacctggttcgtaatgtcg

acgtaaaatcgcgaattatggatcagtatgctgactggaaaggcgtacgctatcgtctgg

gcggcagcactaaaaaaggtatcgattgttctggtttcgtacagcgtacattccgtgagc

aatttggcttagaacttccgcgttcaacttacgaacagcaggaaatgggtaaatctgttt

ctcgcagtaatttgcgtacgggtgatttagttctgttccgtgccggttcaacaggacgcc

atgtcggtatttatatcggcaacaatcagtttgtccatgcttccaccagcagtggtgtta

ttatttccagcatgaatgaaccgtactggaagaagcgttacaacgaagcacgccgggttc

tcagccgcagctaaatgaatattcgccgtaaaaaccgcttgtggattgcctgtgccgtgt

tggcagggctggcgctgactatcggtctggtgctatatgcgctgcgctcgaatatcgatc

tcttttatacgccgggggaaattctctacggcaagcgtgaaactcagcaaatgccggaag

tcggtcagcgtctgcgcgttggcgggatggtgatgccgggtagtgtgcagcgcgatccca

attcgctgaaagtgaccttcaccatttacgatgctgaaggctcagtggatgtctcttacg

aaggcattttgccggatctgttccgtgaagggcagggcgttgtggtgcagggcgagctgg

aaaaaggcaatcatatcctcgcgaaagaagtgctggcgaaacacgacgaaaactacacgc

cgccagaagttgagaaagcgatggaagctaaccaccgtcgcccggcgagtgtttataagg

acccagcatcatgaatgggaaattctgaccgtaagcctggtctgattaagcgcctgtgga

aatggtggcgtacccccagccgtctggcgctggggacgctgctgttgatcggttttgtcg

gcggcatcgtcttctggggcggctttaacactgggatggaaaaagccaataccgaagagt

tctgcattagctgccacgaaatgcgtaacacggtgtatcaggaatacatggattccgtgc

actacaacaaccgtagcggcgtccgtgcgacctgtccggattgtcacgttccgcacgagt

ttgtgccgaagatgatacgcaagctcaaagcaagtaaagagctgtatggtaaaatttttg

gcgttattgacacgccgcagaaatttgaagctcatcgtctgacgatggcacagaatgagt

ggcggcgcatgaaggacaataactcgcaggagtgccgtaactgtcacaacttcgagtata

tggatacaaccgcccagaaatcggttgccgcgaagatgcatgaccaggcggtgaaagatg

ggcaaacctgtattgattgccataaagggatagcgcacaagctgcccgatatgcgtgaag

tcgagccaggtttttaaatgtcccggtcagcgaaacctcaaaatggtcgccgccgctttc

tgcgcgatgttgttcgcacagcaggcgggctggctgccgtgggtgtggcgctggggttac

aacagcaaaccgcacgcgcatctggcgtgcggttgcgcccgcccggagccataaacgaga

acgcctttgccagtgcctgtgtgcgttgtggtcagtgtgttcaggcttgcccttacgaca

ccttaaaactggcgacgctggcctctggtctgtcggcgggcacgccatattttgtcgcac

gggatattccttgcgaaatgtgtgaggacattccgtgcgccaaagtgtgcccgagcggtg

cgctggatcgtgagattgaatcgatcgacgacgcgcggatggggctggcggtactggtgg

accaggaaaactgtctcaactttcaggggctgcgctgcgatgtttgttatcgcgaatgcc

cgaaaattgatgaggccatcaccctggagctggagcgcaacacgcgtaccggtaagcacg

cccgctttctgccgacggttcacagcgacgcctgtactggttgcggtaagtgcgaaaaag

tgtgcgtgctggaacaaccggcaatcaaggtgttaccgctgtcactggcgaaaggggagt

taggtcaccattaccgcttcggctggctggaggggaacaatggcaaatcgtaaatggcta

gcgttaccctgcgcatcactggcacacaactgctgtgccaggatgaacacccttcccttc

tggcggcgctggaatcccacaatgtggcggttgagtaccagtgccgcgaaggttactgcg

gctcctgtcgcacccgactggtcgcaggccaggtggactggattgccgaaccgttagctt

ttattcagccgggggaaattttgccctgttgttgccgggcaaaaggcgatattgaaatcg

agttgtgaatgaccaaagtcggcttacgcattgatgtcgatacctttcgtggcacccgtg

aaggcgtgccgcgtctgctggaaatcttgagtaagcataatattcaggccagcatttttt

tcagcgtcggcccggacaatatgggccgccatctctggcgactggtgaagccacagtttt

tgtggaagatgctgcgctcaaacgcgacatcgctttatggctgggatattttactggcag

gtacggcctggccaggtaaagagattggtcatgccaatgccgatatcattcgtgaagcgg

ctaaacatcacgaagtcggcctgcacgcctgggatcaccatgcctggcaagcccatagcg

gtaactgggatcggcaaacaatgatcgacgatattgcactcggccttcgcaccctggaag

agattatcggtcaaccggtaacctgttctgccgctgcgggctggcgtgccgaccagcagg

tgatcgaagcaaaagaagcgttccatttgcgctacaacagcgattgtcgtggggccatgc

cgttccgtccactgctcgaatcaggaaaccctggcactgcgcaaattccggtgaccttac

ccacctgggatgaagtgattggtcgggatgtgaaagcagaagattttaacggttggttac

tcaaccgcatcctgcgagataaaggcacgccggtttataccattcatgcagaagttgaag

gctgcgcttatcagcataattttgtggatctcctcaaacgcgcagctcaggaaggcgtga

cattttgccctttaagcgaactgttatcagagacgttgccgctcggacaagttgttcgcg

gaaatattgccggacgtgaaggctggctgggttgccaacaaattgcgggtagtcgctgaa

tgatttatcctgatgaagcaatgctttacgcaccggttgaatggcacgactgctccgaag

gtttcgaggacattcgttatgaaaaatccaccgacggtatcgcaaaaatcaccattaatc

gtccgcaggtgcgcaatgccttccgtcctctgacggtaaaagagatgatccaggcactgg

cagatgcgcgttatgacgacaatatcggcgtgatcattctgactggcgcaggcgataaag

cgttctgctccggtggtgaccagaaagtgcgtggtgattacggcggctataaagatgatt

ccggcgtacatcacctgaacgtgctggacttccagcgtcagatccgtacctgtccgaaac

cggttgtcgcgatggtggctggctactccatcggcggtggtcacgttctgcacatgatgt

gcgacctgactatcgcggcagataatgccatctttggtcagactggcccgaaagtcggtt

ccttcgacggcggctggggcgcttcctacatggctcgtatcgtcgggcagaaaaaagcgc

gtgaaatctggttcctgtgccgtcagtacgacgcaaaacaggcgctggatatgggccttg

tcaacaccgtggtaccgctggcggatctggaaaaagaaaccgtccgttggtgccgtgaaa

tgctgcaaaacagcccgatggcgctgcgttgcctgaaagctgcgctgaacgccgactgtg

acgggcaggcggggctgcaggagctggcgggcaacgccaccatgctgttctacatgacgg

aagaaggtcaggaaggtcgcaacgccttcaaccagaaacgtcagcctgacttcagcaaat

tcaaacggaatccgtaaatgatcctgcacgcgcaggcaaaacacggcaaaccaggtttac

cctggctggtgtttttgcacggtttttccggcgattgccacgaatggcaagaagtaggcg

aggcgtttgccgactactcacggttgtatgttgatctcccaggtcacggtggttcggcgg

cgattagagtcgatggatttgatgatgtcactgacttactgcgtaaaaccctggttagtt

acaacatccttaacttctggttggcgggatactcacttggtggacgggtggcgatgatgg

cggcttgccaggggctggcggggctttgtggggttattgtcgaaggtgggcatccggggc

tgcaaaatgctgaacaacgtgcggaacgtcagcgttccgatcgccaatgggcgcagcgtt

ttcgctcagaaccgttaacggcggtgtttaccgactggtatcaacagcctgtttttgcct

cgctcaatgacgatcaacgccgggagctggtggcgctgcgcagcaacaataatggcgcaa

cccttgccgccatgctggaggcgacttctctcgccgttcagcctgatttacgtgctaacc

ttagcgcccgcacatttgcgttttattatttatgtggtgaacgtgacagcaaattccgcg

ccctggcggcggaactggctgccgactgccatgtcattcctcgcgccggacataacgcgc

atcgggaaaatcccgctggcgtaatcgcaagtctggcgcagatcttgcgtttctgaatgt

ctaatcagtttggtgatacacgtatcgatgacgacctgacgctgcttagtgaaacactgg

aagaggtgctccgctcctctggcgatcccgccgatcagaaatatgttgagctgaaagcgc

gtgcagaaaaagcgctggatgatgtgaaaaaacgggttagccaggcttcagacagttatt

actatcgggcgaagcaggctgtttatcgtgctgatgactacgtccacgaaaaaccctggc

agggaattggtgtgggtgcggccgttgggctggtactaggactgttgctggcacgccgtt

aaatgatccccttacaacatggactgatcctcgcggcaatcttattcgttcttggcttaa

ccggtctggttatccgtcgcaatctgctgtttatgctgattggtctggaaatcatgatta

acgcctccgcgctggcctttgtggtcgccggaagctactggggtcagaccgacggtcagg

tgatgtacattctcgccatcagcctcgcggcggcagaagcgagtatcggccttgcgctgc

tgctgcaacttcaccgtcgtcgccagaacctgaacatcgattcagtaagtgagatgcgcg

gatgaatgaccttaaaagaattgttagtaggtttcggcacccaggttcgtagtatctgga

tgatcggcctgcacgcgttcgccaaacgcgaaacgcgaatgtacccggaagagccggtct

atctgccgccccgttatcgtggtcgtatcgttctgacccgcgacccggacggcgaagagc

gttgcgtagcctgtaacctctgcgcggtagcctgcccggtcggctgtatctcgctgcaaa

aagcagaaaccaaagacggtcgctggtatccggaatttttccgcatcaacttctcacgct

gcattttctgtggtttgtgcgaagaagcctgtccgaccacggcgattcagttaaccccgg

atttcgaaatgggggaatacaagcgccaggatctggtttacgagaaagaggatctgctga

tctccggtccgggcaaatacccggagtataacttctaccggatggcaggtatggcaatcg

acggcaaagataagggcgaagcagagaacgaagccaagcctatcgacgtcaagagcctgt

taccgtaaatgcacgagaatcaacaaccacaaaccgaggcttttgagctgagtgcggcag

agcgtgaagcgattgagcacgagatgcaccactacgaagacccgcgtgcggcgtccattg

aagcgctgaaaatcgttcagaagcagcgtggctgggtgccggatggtgcgatccacgcga

tcgccgatgtgctgggtattccggcaagcgacgtcgaaggtgtggcaacgttctacagtc

agatcttccgccagccggttggtcgccatgtgatccgttattgcgacagcgtggtctgtc

atatcaacggttatcagggtattcaggcggcgcttgagaaaaagctgaacatcaaaccag

ggcaaacgacatttgatggccgctttacgctgctgccaacttgctgcctggggaactgtg

ataaagggccaaacatgatgatcgatgaggacactcacgcgcatctgaccccggaagcga

tccctgaactgctggagcggtataaatgaatggattatacgctcacccgcatagatccca

acggtgagaacgaccgttaccccctgcaaaagcaggagatcgtaaccgaccctctggagc

aagaagttaacaaaaacgtgtttatgggcaagctcaatgacatggttaactggggtcgta

aaaactcaatttggccgtataacttcggtctttcctgctgttacgttgagatggtgactt

cgtttaccgcggtgcatgacgtggcgcgttttggcgcagaagtattgcgtgcttcgccgc

gtcaggctgacctgatggtggttgcaggaacctgctttaccaaaatggcaccggttattc

agcgtctgtatgaccagatgctggaaccaaaatgggttatctcaatgggtgcctgtgcca

actctggtggtatgtacgatatttattccgttgtgcagggcgtcgataaattcatcccgg

ttgatgtgtatatcccgggctgcccgccgcgtcctgaagcgtacatgcaggcactgatgc

tgttgcaggaatctatcggcaaagaacgtcgtccgctctcctgggtggttggcgatcagg

gcgtttatcgcgccaatatgcaatcagagcgcgaacgcaagcgcggtgaacgcattgccg

taaccaacctgcgtacacctgacgagatttaaatgagtatgtcaacatccactgaagtca

tcgctcatcactgggcattcgctatctttcttatcgttgccattggcctgtgttgcctga

tgctggtaggcggttggtttttaggcggtcgcgcacgcgcgaggtcgaaaaacgtgccgt

ttgaatccggtatcgactcggtcggctccgcccgcttacgcctgtccgccaagttttatc

tggtggccatgttcttcgttatcttcgacgttgaagcgctgtatctgttcgcatggtcaa

cctctatccgcgaaagcggctgggtaggctttgtggaagctgcaatttttatttttgtgt

tactggcaggtctggtttatctggtgcgtattggcgcgctggactggacgcccgcgcgtt

cacgccgcgagcgtatgaacccggaaacgaacagtatcgctaatcgtcaacgctaaatgt

cccccattgaaaaatccagcaaattagagaatgtctgttatgacatccgtggtccggtgc

tgaaagaagcaaaacgcctggaagaagaaggtaacaaggtactgaaactgaacatcggca

acccagccccgttcggttttgacgcgccagatgaaatcctcgttgacgtgatacgcaacc

tgcctacagctcaagggtattgcgattccaaaggtctttactccgcgcgtaaagccatca

tgcagcactaccaggctcgtggcatgcgtgatgttaccgtggaagatatttacatcggca

atggtgtatcggagcttatcgttcaggcaatgcaggcattgctgaacagcggggacgaaa

tgttggttcctgcaccagattacccactatggaccgcggcggtttcgctttccagcggta

aagcggtgcattatctttgcgatgaatcctctgactggttcccggacctcgatgatattc

gcgctaaaattacgcctcgtacgcgtgggatcgttattatcaacccaaataacccaaccg

gcgcggtatattccaaagagcttttaatggagattgtggagattgcacgtcagcataatc

tcattatcttcgccgatgaaatttatgacaaaattctctacgacgacgctgagcatcact

caattgcgccgctggcacctgacctgctgaccattacctttaatggactgtcgaaaacgt

accgcgttgcaggcttccgtcaggggtggatggtgttgaacgggccgaaaaaacacgcca

aaggctacatcgaaggtctggaaatgctggcttcaatgcgcctgtgtgctaacgttcctg

cgcaacacgccattcagaccgcgctgggtggttatcagagcatcagtgaatttattaccc

ctggcggtcgtctttatgagcagcgtaaccgcgcgtgggaattgatcaacgatattccgg

gcgtttcctgcgtgaaacctcgtggtgcgctgtacatgttcccgaaaatcgacgccaaac

gctttaacattcacgacgatcagaaaatggtgctggatttcctgttgcaggaaaaagttc

tgttggtgcaagggacggcattcaactggccgtggccggatcacttccgcattgtcacgc

taccgcgtgtcgatgatatcgagctgtctttgagcaagttcgcgcgtttcctttctggtt

atcatcagctgtaaatgtcaacgccggataatcgttctgttaatttttttagcttgtttc

gccggggacagcattactcaaagacgtggccgctggaaaaacgccttgccccggtctttg

tcgaaaatcgcgttatcaagatgacgcgttatgcgatccgttttatgccgccgatcgccg

tatttactctctgctggcagattgccctgggcggtcagcttgggccggcagttgccactg

-cgctgttcgccttaagtttacccatgcagggattgtggtggctgggcaagcgttctgtc

acgccattaccccctgctatcctcaactggttttatgaagttcgcggtaaattgcaggag

tctggacaggtgttggcacccgttgaaggcaagcctgattaccaggcattagctgacacg

cttaagcgcgccttcaaacaactggataaaactttccttgatgatttgtaaatgtcgagt

aagttagtactggttctgaactgcggtagttcttcactgaaatttgccatcatcgatgca

gtaaatggtgaagagtacctttctggtttagccgaatgtttccacctgcccgaagcacgt

atcaaatggaaaatggacggcaataaacaggaagcggctttaggtgcaggcgccgctcac

agcgaagcgctcaactttatcgttaatactattctggcacaaaaaccagaactgtctgcg

cagctgactgctatcggtcaccgtatcgtacacggcggcgaaaagtataccagctccgta

gtgatcgatgagtctgttattcagggtatcaaagatgcagcttcttttgcaccgctgcac

aacccggctcacctgatcggtatcgaagaagctctgaaatctttcccacagctgaaagac

aaaaacgttgctgtatttgacaccgcgttccaccagactatgccggaagagtcttacctc

tacgccctgccgtacaacctgtacaaagagcacggcatccgtcgttacggcgcgcacggc

accagccacttctatgtaacccaggaagcggcaaaaatgctgaacaaaccggtagaagaa

ctgaacatcatcacctgccacctgggcaacggtggttccgtttctgctatccgcaacggt

aaatgcgttgacacctctatgggcctgaccccgctggaaggtctggtcatgggtacccgt

tctggtgatatcgatccggcgatcatcttccacctgcacgacaccctgggcatgagcgtt

gacgcaatcaacaaactgctgaccaaagagtctggcctgctgggtctgaccgaagtgacc

agcgactgccgctatgttgaagacaactacgcgacgaaagaagacgcgaagcgcgcaatg

gacgtttactgccaccgcctggcgaaatacatcggtgcctacactgcgctgatggatggt

cgtctggacgctgttgtattcaccggtggtatcggtgaaaatgccgcgatggttcgtgaa

ctgtctctgggcaaactgggcgtgctgggctttgaagttgatcatgaacgcaacctggct

gcacgtttcggcaaatctggtttcatcaacaaagaaggtacccgtcctgcggtggttatc

ccaaccaacgaagaactggttatcgcgcaagacgcgagccgcctgactgcctgaatggaa

cagcgtcgtttggcaagtactgaatgggtggatattgtcaatgaagagaacgaagtcatt

gcacaagccagccgggaacaaatgcgggcacagtgtctgcgtcatcgtgcaacttacatc

gtcgtgcatgatggcatgggcaaaattctggtccagcgtcgtaccgagacaaaagacttt

ttacccggcatgttagatgcgaccgcaggcggtgtagtccaggccgatgagcaactgctg

gaatccgcgcgtcgcgaagcggaagaagagttgggcattgccggtgtcccctttgccgag

cacgggcagttctatttcgaagataaaaattgccgtgtctggggcgcattgttcagctgc

gtctctcacggtcccttcgccctacaggaagatgaagtcagtgaagtttgctggctgacg

ccggaagaaatcaccgcacgctgcgatgagttcactccagactcgctgaaagcgctagcg

ttgtggatgaagcgcaatgccaaaaatgaagccgtagagactgaaacggcagaatgaatg

atcgatctctatttcgccccgacacccaatggccacaaaattacgctgtttctcgaagaa

gcagggctggattatcgcttgataaaggtagacctggggaaaggcggtcagtttcgcccg

gaatttttgcgcatttcgcctaacaacaaaattccggcaattgttgatcattctccagcc

gatggcggcgaaccgctaagcctctttgaatctggtgccattttgttgtatctggctgag

aaaacggggctctttttgagtcatgaaacgcgtgaacgcgccgccacattacagtggtta

ttctggcaggtaggcggactggggccgatgcttgggcaaaatcatcattttaatcacgca

gccccccaaaccattccttacgctattgaacgttatcaggttgaaactcagcgtctgtac

catgtactgaacaagcggctggaaaactcgccctggctgggaggcgagaactacagcatt

gcggatattgcctgctggccgtgggttaatgcctggactcgccagcgaattgacctcgca

atgtatccggcagtcaagaactggcatgagcggatccgttcgcgccctgccaccgggcag

gcactgctaaaagcacaactcggtgatgagcgttcggatagttaaatggtctggattgat

tacgccataatcgcggtgattgctttttcctctctggttagcctgatccgcggctttgtt

cgtgaagcgttatcgctggtgacatggggttgtgctttctttgttgccagtcattactac

acttacctgtcagtctggtttacgggctttgaagacgaactggttcgaaatgggattgcc

atcgcggtactgtttatcgctaccctgatcgttggtgctatcgtgaacttcgtgataggc

cagttggtggagaaaacggggttgtcaggcaccgatcgggtgctgggcgtctgtttcggt

gcgttgcgcggtgtgttgattgttgccgccattctcttctttctcgactcctttaccggg

gtgtcgaaaagcgaagactggagcaaatcacagctgatcccgcagttcagttttatcatc

agatggttttttgattatctgcaaagctcgtcaagtttcttgcccagagcgtaaatgatc

gcggagtttgaatcacgcattctggcattaatcgacggtatggttgaccatgccagtgat

gatgagttgtttgccagtgggtatttgcgtggccacctgacattagccatcgcagaactg

gaaagtggtgatgaccactccgctcaggcggtgcatacgaccgttagccagagtctggaa

aaagccattggtgccggtgaattgtcgccgcgtgaccaggcgctggtgaccgatatgtgg

gaaaacctgtttcagcaggcgtcacagcagtaaatgaacaaaaccgcgattgcgctgctg

gccctgcttgccagtagcgtcagcctggcagcgacgccgtggcaaaaaataacccaacct

gtgccgggtagcgcacaatcgataggcagtttttctaatggctgtatcgtcggcgctgac

acgctgccgatacagtccgaacattatcaggtcatgcgtaccgatcagcgtcgctatttc

ggtcacccggacctggtgatgtttatccagcgtctgagtagccaggtgagcaatctgggc

atgggtacggtgctgattggcgatatggggatgcccgctggtgggcgtttcaacggcggt

catgccagccaccagaccggactggatgtcgatatctttctgcaactgccgaaaactcgc

tggacctccgcgcagctcttgcgcccgcaagcactggacttagtatcctgcgacggtaaa

cacgttgtctccacgctgtggaagccagaaattttcagcttgatcaaactcgccgcccag

gacaaagacgtcacgcgcatttttgttaatccggcgattaaacaacaactttgccttgat

gcgggcaccgatcgcgactggttgcgcaaagtgcgaccctggttccagcatcgtgcgcat

atgcatgtacgattacgttgtcctgccgatagtctggagtgtgaagatcaacctttaccg

ccaccaggcgatggttgcggggcagaactgcaaagctggtttgaacctccaaaaccggga

acaacaaagcctgagaagaagacaccgcctccgttgccgccttcctgccaggcgctactg

gatgagcacgtgatctaaatgcaacttgaaaagatgattaccgaaggctcgaacgccgcc

tcggctgaaattgaccgcgtatcaacgttggaaatgtgccggattatcaacgatgaagat

aaaaccgtaccgcttgccgttgagcgcgtactgccggatatcgccgcggcgatcgatgtt

atccacgcccaggtcagcggcggcggtcgtctgatttatctcggtgcgggaacatccggt

cgtctggggattctggatgccagcgaatgtccgcccacctacggcgtgaaaccgggtttg

gtggttggtttgattgctggaggcgaatatgccattcagcacgcggtggaaggcgcggaa

gatagccgggaaggcggcattaacgatctgaaaaatattaatttaacggcacaggatgtg

gtggtcggtattgccgccagcggtcgtacgccgtatgtgattgccggactggaatatgca

cgccagctcggctgccgtacagtgggaatttcctgtaatccggggagcgccgtttccacc

accgctgagtttgctattacgccggtggttggggccgaagtggtcaccggttcttcgcgg

atgaaagcaggcacggcgcagaaactggtgctcaatatgctttccaccgggctgatgatt

aaatccggcaaagtgttcggcaacctgatggtcgatgtggtcgccaccaacgaaaaactg

catgtgcgccaggtcaatatcgttaaaaacgccaccggatgtagcgcagagcaagcggaa

gcggcgttaattgcctgcgagcgcaactgtaaaacggccattgtgatggtgctgaaaaat

ctcgatgccgctgaagctaaaaaacgcctgaatcaacacggcggatttattcgcaaggct

ctggaaaaggaataaatgagcacttttaaaccactaaaaacactcacttcgcgccgccag

gtgctgaaagccggtttggctgccctgacgttgtcaggaatgtcgcaagccatcgccaaa

gacgaacctttaaaaaccagcaacggacacagcaagccgaaagctaaaaaatctggcggc

aaacgtgtcgttgttctcgatccaggccacggcgggattgataccggagcgatcgggcgc

aacggttcgaaagaaaaacatgtggtgctggcgattgctaaaaacgtccgttccattttg

cgtaatcatgggattgatgcgcgtttaacgcgttctggcgatacgtttatcccactttac

gatcgcgttgaaatcgcccataaacatggcgcagatctgtttatgtcaattcatgccgat

ggctttaccaacccgaaagctgccggtgcttcggtatttgccctctctaaccgtggggca

agtagcgcaatggcgaaatacctgtctgaacgcgaaaaccgcgccgatgaagttgccggt

aaaaaggcgactgacaaggatcacctattgcagcaagtgctgtttgatctggtgcaaacc

gatactattaaaaacagcctgacgctcggctcgcatattctgaaaaagattaagccggtg

cataaactgcacagccgcaacaccgaacaagccgcgtttgtggtgttgaaatcgccgtcg

gttccttcggtgctggtggaaacctcgtttatcaccaacccggaagaagagcgactgtta

ggctcggcggcgtttcgtcagaaaatcgccacagcgattgctgaaggcgtgatcagttat

ttccactggttcgacaaccagaaagcacattcgaaaaagcgataaatggttacactttac

ggtatcaaaaattgtgacaccattaaaaaggctcgccgttggctggaagccaataacatc

gactatcgttttcatgattaccgcgtcgatgggctggacagcgaattattgaacggtttt

atcaacgaattaggctgggaagcgttactcaacacccgtggtacaacctggcgtaaactg

gacgaaaccacccgcaataaaatcaccgatgcggcctctgcggcggcattaatgactgaa

atgcctgcaattatcaaacgtccattgctctgcgcgcccggtaagcctatgctgctgggt

ttcagtgattccagttatcagcaatttttccatgaggtgtagatgaatccactgaaagcc

ggtgatatcgcaccgaaatttagcttgccggatcaagacggagaacaagttaatttgacc

gacttccagggacagcgtgttctggtttatttctacccgaaagccatgacccccggctgt

accgtacaggcctgcggcttacgcgataacatggatgagttgaaaaaagcgggcgttgat

gtgctgggtatcagcaccgataaacccgaaaaactctcccgttttgcggaaaaagagctg

cttaactttacgctcctgtctgatgaggaccacctggtgtgcgagcaattcggcgtctgg

ggggaaaagtccttcatgggcaaaacctacgatggtattcatcgcatcagcttcctgatt

gacgctgatggcaaaatcgaacatgtctttgacgatttcaaaaccagcaatcaccacgac

gttgtgctgaactggctgaaagaacacgcctgaatgctcgaaatgttgatgcaatggtat

cgccgccgttttagcgacccggaagcgattgccttgctggttattttagttgccggattt

ggcattatctttttctttagtggcctgcttgctccgttgctggtggctattgtgctggcc

tatttgctggaatggccaaccgtgcgcctgcaatctattggctgctcccgccgctgggcg

acgtcgattgtattggtggttttcgtcggtatattgctactgatggcgttcgtggtactg

cctatcgcctggcaacagggcatctacttaatccgcgatatgccggggatgctcaataag

ctttctgactttgccgccacgttgccgcgccgctatccggcgttaatggatgcgggcatt

attgatgcaatggccgaaaatatgcgcagtcggatgctgaccatgggcgattcggtggtg

aaaatttccctcgcctcgctggtcggtttgctgaccatagccgtctatctggtgctggtg

ccattgatggtcttcttcctgctgaaagacaaagagcagatgctgaacgccgttcgtcgg

gtgctgccgcgcaaccgtggactggcaggacaggtgtggaaggagatgaatcaacaaatc

accaactatatccgcggcaaagtgctggagatgatcgtggtggggatcgccacctggctg

gggttcttgctctttgggctgaactattcgctgctgctggcggtgctggtcggcttctcg

gtacttattccgtacattggcgcatttgtggtgaccattccggtggttggcgtggcgcta

ttccagtttggtgcaggcactgaattctggagctgtttcgcggtgtatctgattattcag

gcgcttgacggcaatctgttagtaccggtgttgttctccgaagcggttaacctgcatccg

ctggtgattattttatcggtggtgatcttcggtggtttgtggggattctggggcgtattc

ttcgccattccattggcgacgctgatcaaagccgtgatccacgcctggcccgatgggcaa

atagcgcaagaataaatgaatattgtggtgcttatttccggcaacggaagtaatttacag

gcaattattgacgcctgtaaaaccaacaaaattaaaggcaccgtacgggcagttttcagc

aataaggccgacgcgttcggccttgaacgcgcccgccaggcgggtattgcaacgcatacg

ctcatcgccagcgcgtttgacagtcgtgaagcctatgaccgggagttgattcatgaaatc

gacatgtacgcacccgatgtggtcgtgctggctggttttatgcgcattctcagcccggcg

tttgtctcccactatgccgggcgtttgctgaacattcacccttctctgctgccgaaatat

cccggattacacacccatcgtcaggcgctggaaaatggcgatgaagagcacggtacatcg

gtgcatttcgtcaccgatgaactggacggtggcccggttattttacaggcgaaagtcccg

gtatttgctggtgatacggaagatgacgtcaccgcccgcgtgcaaacccaggaacacgcc

atttatccactggtgattagctggtttgccgatggtcgtctgaaaatgcacgaaaacgcc

gcgtggctggatggtcaacgtctgccgccgcagggctacgctgccgacgagtaagtggaa

atttacgagaacgaaaacgaccaggtagaagcggttaaacgcttttttgctgaaaatggc

aaagcactggctgttggggtgattttgggcgttggcgcactgattggctggcgctactgg

aacagccatcaggttgattctgcacgctccgcttctcttgcctatcaaaatgcggttacc

gcagtgagcgaaggcaaaccggatagcatcccggcggcggaaaaatttgctgctgaaaat

aaaaatacttatggtgcgctggcttctttggaacttgcgcagcaatttgttgacaaaaat

gaactagagaaagctgccgcccagttacaacaggggctggcagacacgagcgatgaaaat

ctcaaagccgtgataaatctgcgtcttgctcgcgttcaggtacagctcaagcaggctgat

gccgcgctgaaaacccttgataccatcaaaggtgaagggtgggctgccattgttgccgac

ctgcgtggtgaagcattgctgagcaaaggtgataagcaaggtgcgcgtagtgcatgggaa

gcaggcgtgaaaagcgatgttactccggcactgagcgaaatgatgcagatgaaaattaat

aatttgtccatctgaatggctattgaacgtactttttccattatcaaaccgaacgcggta

gcaaaaaacgtcattggtaatatctttgcgcgctttgaagctgcagggttcaaaattgtc

ggcaccaaaatgctgcacctgaccgttgaacaggctcgtggcttttatgctgaacacgat

ggaaaaccgttctttgatggtctggttgaattcatgacctctggcccgatcgtggtttcc

gtgctggaaggtgaaaacgccgttcagcgtcaccgcgatctgctgggcgcgaccaatccg

gcaaacgcactggccggtactctgcgcgctgattacgctgacagcctgaccgaaaatggt

acccacggttctgactccgtcgaatctgccgctcgcgaaatcgcttatttcttcggcgaa

ggcgaagtgtgcccgcgcacccgttaaatggcttacagcgaaaaagttatcgaccattac

gagaatccgcgtaacgtgggttcctttgacaacaacgacgagaacgtcggcagcggcatg

gtgggtgcaccggcctgtggcgacgtgatgaagttgcagattaaagtcaacgatgaaggt

atcattgaagacgcgcgttttaaaacttacggctgcggttccgctatcgcttccagctcc

ctggtcaccgaatgggtgaaagggaagtctctcgacgaagcgcaggcgatcaaaaacacc

gatattgctgaagaacttgaactgccgccggtgaaaattcactgttctattctggcagaa

gacgcgatcaaagccgccattgcggactataaaagcaaacgtgaagcaaaataaatgaga

ctgacatctaaagggcgctatgccgtgaccgcaatgcttgacgttgcgctcaactctgaa

gcgggcccggtaccgttggctgatatttccgaacgtcagggaatttccctttcttatctg

gaacaactgttttcccgtctgcgtaaaaatggtctggtttccagcgtacgtggaccaggc

ggtggttatctgttaggcaaagatgccagcagcatcgccgttggcgaagtgattagcgcc

gttgacgaatctgtagatgccacccgttgtcagggtaaaggcggctgtcagggcggcgat

aaatgcctgacccacgcgctgtggcgtgatttgagcgaccgtctcaccggttttctcaac

aacattactttaggcgaactggttaataaccaggaagtgctggatgtgtctggtcgtcag

catactcacgacgcgccacgcacccgcacacaagacgcgatcgacgttaagttacgcgct

taaatgcatccgatgctgaacatcgccgtgcgcgcagcgcgcaaggcgggtaatttaatt

gccaaaaactatgaaaccccggacgctgtagaagcgagccagaaaggcagtaacgatttc

gtgaccaacgtagataaagctgccgaagcggtgattatcgacacgattcgtaaatcttac

ccacagcacaccatcatcaccgaagaaagcggtgaacttgaaggtactgatcaggatgtt

caatgggttatcgatccactggatggcactaccaactttatcaaacgtctgccgcacttc

gcggtatctatcgccgttcgtatcaaaggccgcaccgaagttgctgtggtatacgatcct

atgcgtaacgaactgttcaccgccactcgcggtcagggcgcacagctgaacggctaccgt

ctgcgcggcagcaccgctcgcgatctcgacggtactattctggcgaccggcttcccgttc

aaagcaaaacagtacgccactacctacatcaacatcgtcggcaaactgttcaacgaatgt

gcagacttccgtcgtaccggttctgcggcgctggatctggcttacgtcgctgcgggtcgt

gttgacggtttctttgaaatcggtctgcgcccgtgggacttcgctgcaggcgagctgctg

gttcgtgaagcgggcggcatcgtcagcgacttcaccggtggtcataactatatgctgacc

ggtaacatcgttgctggtaacccgcgtgttgttaaagccatgctggcgaacatgcgtgac

gagttaagcgacgctctgaagcgttaaatgcttgacgctcaaaccatcgctacagtaaaa

gccaccatccctttactggtggaaacggggccaaagttaaccgcccatttctacgaccgt

atgtttactcataacccagaactcaaagaaatttttaacatgagtaaccagcgtaatggc

gatcaacgtgaagccctgtttaacgctattgccgcctacgccagtaatattgaaaacctg

cctgcgctgctgccagcggtagaaaaaatcgcgcagaagcacaccagtttccagatcaaa

ccggaacagtacaacatcgtcggtgaacacctgttggcaacgctggacgaaatgttcagc

ccggggcaggaagtgctggacgcgtggggtaaagcctatggtgtactggctaatgtattt

atcaatcgcgaggcggaaatctataacgaaaacgccagcaaagccggtggttgggaaggt

actcgcgatttccgcattgtggctaaaacaccgcgcagcgcgcttatcaccagcttcgaa

ctggagccggtcgacggtggcgcagtggcagaataccgtccggggcaatatctcggcgtc

tggctgaagccggaaggtttcccgcatcaggaaattcgtcagtactctttgactcgcaaa

ccggatggcaaaggctatcgtattgcggtgaaacgcgaagagggtgggcaggtatccaac

tggttgcacaatcacgccaatgttggcgatgtcgtgaaactggtcgctccggcaggtgat

ttctttatggctgtcgcagatgacacaccagtgacgttaatctctgccggtgttggtcaa

acgccaatgctggcaatgctcgacacgctggcaaaagcaggccacacagcacaagtgaac

tggttccatgcggcagaaaatggcgatgttcacgcctttgccgatgaagttaaggaactg

gggcagtcactgccgcgctttaccgcgcacacctggtatcgtcagccgagcgaagccgat

cgcgctaaaggtcagtttgatagcgaaggtctgatggatttgagcaaactggaaggtgcg

ttcagcgatccgacaatgcagttctatctctgcggcccggttggcttcatgcagtttgcc

gcgaaacagttagtggatctgggcgtgaagcaggaaaacattcattacgaatgctttggc

ccgcataaggtgctgt--atggaaggctggcagcgcgcatttgtcctgcatagtcgcccg

tggagcgaaaccagcctgatgctggacgtcttcacggaggaatcggggcgcgtgcgtctg

gttgccaaaggcgcacgctctaaacgctctaccctgaaaggtgcattacagcctttcacc

cctctcttgctacgttttggcgggcgtggcgaagtcaaaacgctgcgcagtgctgaagcc

gtctcgctggcgctgccattaagcggtatcacgctttacagcggtctgtacatcaacgaa

cttctctcccgcgtactggaatacgagacgcgcttctctgaactttttttcgattacttg

cactgcattcagtctcttgcagggggcactggtacgccagaacccgcgctgcgccgcttt

gaactggcactgctcgggcatctgggttatggcgtcaattttacccattgtgcgggtagc

ggcgagccggtagatgacaccatgacgtatcgttatcgcgaagaaaaagggtttatcgca

agcgtcgttatcgataataaaacgttcaccggaaggcagttaaaagcgttaaacgcacgg

gaatttcctgacgcagacacactgcgcgccgcgaaacgctttacccgcatggcgcttaag

ccgtatcttggcggtaaacctttaaagagcagggaactgttccggcaatttatgcctaag

cgaacggtgaaaacacattatgaatgaatgaaccccatcgtaattaatcggcttcaacgg

aagctgggctacacttttaatcatcaggaactgttgcagcaggcattaactcatcgtagt

gccagcagcaaacataacgagcgtttagaatttttaggcgactctattctgagctacgtt

atcgccaatgcgctttatcaccgtttccctcgtgtggatgaaggcgatatgagccggatg

cgcgccacgctggtccgtggcaatacgctggcggaactggcgcgcgaatttgagttaggc

gagtgcttacgtttagggccaggtgaacttaaaagcggtggatttcgtcgtgagtcaatt

ctcgccgacaccgtcgaagcattaattggtggcgtattcctcgacagtgatattcaaacc

gtcgagaaattaatcctcaactggtatcaaactcgtctggacgaaattagcccaggcgat

aaacaaaaagatccgaaaacgcgcttgcaagaatatttgcagggtcgccatctgccgctg

ccgacttatctggttgtccaggtacgtggcgaagcgcacgatcaggaatttactatccac

tgccaggtcagcggcctgagtgaaccggtggttggcacaggttcaagccgtcgtaaggct

gagcaggctgccgccgaacaggcgttgaaaaaactggagctggaatgaatgaagcaactt

tggtttgccatgtcattagtgacaggtagcctgttattctctgctaacgcctcggccact

cccgcgtccggggcgttattacagcagatgaacctggccagtcagtcactgaattacgag

ctgtcattcatcagcatcaataaacagggtgttgagtctctgcgttatcgacatgcacgc

ctcgataaccgtcctcttgcacaattgttgcaaatggatggcccgcgccgggaagtggta

cagcgcggcaatgaaatcagctattttgaaccgggacttgaaccgttcacgcttaatggc

gattacattgttgattctctgccatcgctcatctataccgatttcaaacgcctttctcct

tactacgactttatctccgtcgggcgcacgcgtattgctgatcgtctttgcgaagtcatt

cgcgtggttgcccgagatggtacacgctacagctacatcgtgtggatggacaccgaatcg

aaattaccgatgcgggttgatcttcttgatcgcgatggtgaaacgctggaacaatttcgc

gtgattgcttttaacgtcaatcaggatatcagcagcagtatgcagacgctggcgaaggca

aatttgccgccgttgctttctgttcctgtaggtgaaaaagctaaattcagctggacgcca

acctggttgccacagggttttagcgaagtttccagtagtcgacgtccgctaccgacgatg

gacaacatgcctatcgaatcacgtctctattccgacggattattcagcttctcggtaaac

gttaaccgcgctacgccatcgagcaccgatcagatgttgcgcaccggacgcagaaccgtc

agtacaagcgtccgtgataacgccgaaatcaccattgtcggtgaactgccgccgcaaacg

gcgaaacgcattgccgagaatattaagttcggggcagcgcaatgaatgaataccgtttgt

acccattgtcaggccatcaatcgcattcccgacgatcggatcgaagatgcggcaaaatgc

ggacgctgcggtcacgacttgtttgacggagaggtgattaatgcgaccggtgaaacgctc

gacaaattgctgaaagatgatctaccagtggtgatcgacttctgggcaccgtggtgcggc

ccctgccgtaatttcgcaccaatttttgaagatgtcgcgcaagagcgtagcggtaaagtg

cgctttgtgaaagtgaataccgaagctgaacgtgaattaagcagccgctttggtattcgc

agtattccgacgatcatgattttcaaaaacggtcaggttgtcgacatgcttaatggcgca

gtgccgaaagcgccgttcgatagctggctgaacgaatctctttaaatggcacaacgagta

cagctcactgcaacggtgtccgaaaaccaactcggtcaacgcttagatcaggctttggcc

gaaatgttcccggattattcacgttcgcgaataaaagaatggatcctcgaccagcgcgtg

ctggtaaacggcaaagtttgtgataagccgaaagaaaaagtattgggtggcgagcaggtt

gccatcaacgctgagattgaagaagaagcgcgttttgaaccgcaggatatcccgctggat

atcgtctatgaagatgaagatatcatcgtcattaataaaccgcgcgatctggtggtacat

cctggcgcgggtaacccggatggcacggtactgaatgcgttacttcattactatccgccc

attgctgatgtaccgcgtgcaggcatcgtccatcgtctggataaagacaccactggcctg

atggttgtggcaaaaaccgttccggctcagacgcgtttagtcgaatctttgcaaaggcgt

gaaattactcgtgagtatgaagcggtggcaattggtcatatgaccgctggcggtacggtg

gatgagccaatcagtcgccacccgaccaaacgtactcacatggcggtgcatccgatgggc

aaaccagcggtgactcactatcgcatcatggaacacttccgtgtgcacacgcgtctgcgg

ttgcgtctggaaactggacgtacgcaccagatccgcgtgcatatggcccatatcactcat

ccgctggtgggcgatccggtttatggtggccgtccgcgtccgccaaaaggtgcttcggaa

gcgtttatctccacgctgcgtaagtttgaccgccaggcgcttcatgcaaccatgctgcgt

ctttatcacccgatctccggcatcgaaatggaatggcatgcgcctattccacaagatatg

gtggagctgattgaggcgatgcgtgccgatttcgaagaacataaggatgaagtggactgg

ttatgaatgacgcgcatgaaatatctggtggcagccgccacactaagcctgtttttggcg

ggttgctcggggtcaaaggaagaagtacctgataatccgccaaatgaaatttacgcgact

gcacaacaaaagctgcaggacggtaactggagacaggcaataacgcaactggaagcgtta

gataatcgctatccgtttggtccgtattcgcagcaggtgcagctggatctcatctacgcc

tactataaaaacgccgatttgccgttagcgcaggctgccatcgatcgttttattcgcctt

aacccgacccatccgaatatcgattatgtcatgtacatgcgtggcctgaccaatatggcg

ctggatgacagtgcgctgcaagggttctttggcgttgaccgtagcgatcgcgatcctcaa

catgcacgagctgcgtttagtgacttttccaaactggtgcgcggctatccgaacagtcag

tacaccaccgatgccaccaaacgtctggtattcctgaaagatcgtctggcgaaatatgaa

tactccgtagccgagtactatacagaacgtggcgcatgggttgccgtcgttaaccgcgta

gaaggcatgttgcgcgactacccggatacccaggctacgcgtgatgcgctgccgctgatg

gaaaatgcataccgtcagatgcagatgaatgcgcaagctgaaaaagtagcgaaaatcatc

gccgcaaacagcagcaatacataaatgaataatcatttcaagtgtattggcattgtggga

cacccacggcaccccactgcactgacaacacatgaaatgctctaccgctggctgtgcaca

aaaggttacgaggttatcgtcgagcagcaaatcgctcacgaactgcaactgaagaatgtg

aaaaccggcacactcgccgagattgggcaactggcagatctcgcagtagttgttggtggc

gacggtaatatgctcggcgcagcgcgcacgctcgcccgctacgatattaaagttattgga

atcaaccgtggcaacctgggtttcctgactgaccttgatcccgataacgcccagcaacag

ttagccgatgtgctggaaggtcactacatcagcgaaaaacgttttttgctggaagcgcaa

gtctgccagcaagattgccagaaacgcatcagcacggcgattaacgaagtggtacttcac

cctggcaaagtggcgcatatgattgagtttgaagtgtatatcgacgagatctttgcgttt

tcgcagcgatctgatggcctgattatttcgacgccaacaggctccaccgcctattccctc

tcagcaggcggtccaatactgacgccttctctggatgcgattaccctggtgcccatgttc

ccgcatacgttgtcagcacgaccactggtcataaacagcagcagcacgatccgtctgcgt

ttttcgcatcgccgtaacgacctggaaatcagttgcgacagccagatagcactgccgatt

caggaaggtgaagatgtcctgattcgtcgctgtgattaccatctgaatctgattcatccg

aaagattacagttatttcaacacattaagtaccaagctcggctggtcaaaaaaattattc

taaatgacgaagaaaaaagcacataaacctggttcagcgaccatcgcgcttaacaagcgc

gcccgtcacgaatactttatcgaagaagagttcgaagcgggacttgccctgcaaggctgg

gaagttaaatccctgcgcgcaggaaaagccaatatcagcgacagctacgtccttctgcgt

gacggagaggcatttctgtttggcgctaacatcacgccaatggccgtggcctccacgcat

gtggtgtgcgatcctacccgtacccgcaagttacttctcaaccagcgcgaactggactca

ttgtacggtcgcgtcaatcgagaaggctataccgtagtggcgctctccctgtactggaaa

aatgcctggtgcaaagtgaaaatcggcgtcgccaaaggtaagaaacagcacgataaacgt

tcagatatcaaagagcgcgaatggcaggtggataaagcgcgtatcatgaaaaacgcccac

cgttaaatgggtctgttcaattttgtgaaagatgccggagaaaaactctgggacgcggtt

acaggtcagcacgataaagacgatcaggcgaagaaggtgcaggagcatctgaacaaaacc

ggtatacctgatgccgataaagtgaatattcaaattgccgacggcaaagcgacggtcact

ggtgacggtcttagtcaggaggcgaaagagaaaatccttgttgcggtggggaatatttcc

ggcattgccagtgttgatgatcaggtgaaaacggcgacaccagccactgccagccagttt

tataccgttaagtctggcgacactctgagtgccatttccaaacaggtctacggtaacgct

aatctgtacaataaaatcttcgaagcgaataaaccgatgctaaaaagcccggataaaatt

tatccggggcaagtgttgcgtattccggaagagtagatgactgaactcgcgcaattacag

gctagtgccgaacaggcagcggccttattgaaagcaatgagccaccctaaacggttgctg

attctgtgcatgcttagcggttcccccggcaccagcgcgggagagctgacgcgcattacc

ggactgagtgcctctgcgacatcacagcatctcgcccgtatgcgggacgaagggcttatc

gacagccaacgggatgcccaacgcattctatattccattaaaaatgaggcggtaaatgcc

attatcgccaccctgaaaaatgtctattgtccgtaaatgtccgtaatgttacaaagttta

aataacattcgcaccctccgtgcgatggctcgcgaattctccattgacgttcttgaagaa

atgctcgaaaaattcagggttgtcactaaagaaagacgtgaagaagaagaacagcagcag

cgtgaactggcagagcgccaggaaaaaattagcacctggctggagctgatgaaagctgac

ggaattaacccggaagagttattgggtaatagctctgctgctgcaccacgcgctggtaaa

aaacgccagccgcgtccggcgaaatataaattcaccgatgttaacggtgaaactaaaacc

tggaccggtcagggccgtacaccgaagccaatcgctcaggcgctggcagaaggtaaatct

ctcgacgatttcctgatctaaatgttctcaccgcagtcacgcttgcgtcatgcagttgca

gatacgttcgcgatggttgtttactgttctgtcgtgaacatgtgtattgaagttttcctc

tccggaatgagcttcgaacagtctttttattccagattggtagcgattccggtgaacatc

ttaattgcatggccatacggtatgtaccgtgatctgtttatgcgcgcggcacgcaaagtt

agcccgtcgggctggataaaaaatctggcggatatcctggcttatgtgacgttccagtca

ccggtgtatgtggcgatcttgttagtggtgggcgcagactggcatcagattatggcggcg

gtcagttcaaacatcgttgtttcgatgttgatgggggcggtttatggctacttcctcgat

tattgccgccgactgtttaaagtcagccgttaccagcaggtaaaagcctgaatggaaagc

cctactacacagcctgctcctggttcggcgaccttcatggaaggatgcaaagacagttta

ccgattgttattagttatattccggtggcctttgcgttcggtctgaatgcgacccgtctg

ggattctctcctctcgaaagcgtttttttctcctgcatcatttatgcaggcgcgagccag

ttcgtcattaccgcgatgctggcagcagggagtagtttgtgggttgctgcactgaccgtc

atggcaatggatgttcgccatgttttgtatggcccgtcactgcgtagccgtattattcag

cgtctgcaaaaatcgaaaaccgccctatgggcgtttggcctgacggatgaggtttttgcc

gccgccaccgcaaaactggtacgcaataatcgccgctggagcgagaactggatgatcggc

attgccttcagttcatggtcatcgtgggtctttggtacggtaataggggcattctccggc

agcggcttgctgcaaggttatcccgccgttgaagcagcattaggttttatgcttccggca

ctctttatgagtttcctgctcgcctctttccagcgcaaacaatctctttgcgttaccgca

gcgttagttggtgcccttgcaggcgtaacgctattttctattcccgtcgccattctggca

ggcattgtctgtggctgcctcactgcgttaatccaggcattctggcaaggagcgcccgat

gagctatgaatgagctatgaggttctgctgcttgggttactggttggcgcggcgaattat

tgcttccgctatttgccgctgcgcctgcgtgtgggtaatgcccgcccaaccaaacgtggc

gcggtaggtattttgctcgacaccattggcatcgcctcgatatgcgctctgctggttgtc

tctaccgcaccagaagtgatgcacgatacacgccgtttcgtgcccacgctggtcggcttc

gcggtactgggtgccagtttctataaaacacgcagcattatcatcccaacactgcttagt

gcgctggcctatgggctcgcctggaaagtgatggcgattatataaatgccgttgttagat

agcttcacagtcgatcatacccggatggaagcgcctgcagttcgggtggcgaaaacaatg

aacaccccgcatggcgacgcaatcaccgtgttcgatctgcgcttctgcgtgccgaacaaa

gaagtgatgccagaaagagggatccataccctggagcacctgtttgctggttttatgcgt

aaccatcttaacggtaatggtgtagagattatcgatatctcgccaatgggctgccgcacc

ggtttttatatgagtctgattggtacgccagatgagcagcgtgttgctgatgcctggaaa

gcggcaatggaagacgtgctgaaagtgcaggatcagaatcagattccggagctgaacgtc

taccagtgtggcacttaccagatgcactcgttgcaggaagcgcaggatattgcgcgtagc

attctggaacgtgacgtgcgcatcaacagcaacgaagaactggcgctgccgaaagagaag

ttgcaggaactgcacatttagatgacagaatcaacgtcccgtcgcccggcatatgctcgc

ctgttggatcgtgcggtacgcattctggcggtgcgcgatcacagtgagcaagaactgcga

cgtaaactcgcggcaccgattatgggcaaaaatggcccagaagagattgatgctacggca

gaagattacgagcgcgttattgcctggtgccatgaacatggctatctcgatgacagccga

tttgttgcgcgctttatcgccagccgtagccgcaaaggttatggacctgcgcgtattcgc

caggaactgaatcagaaaggtatttcccgcgaagcgacagaaaaagcgatgcgtgaatgt

gacatcgactggtgcgcactggcgcgcgatcaggcgacgcgaaaatatggtgaacctttg

ccaactgtcttttcagaaaaagttaagatccagcgttttctgctctatcgtggctatctg

atggaagatatccaggatatttggcgaaattttgccgactgaatgtgcataggcgttccc

ggccagatccgcaccattgacggcaaccaggcgaaagtcgacgtctgcggcattcagcgc

gatgtcgatttaacgttagtcggcagctgcgatgaaaacggtcagccgcgcgtgggccag

tgggtactggtacacgttggctttgccatgagcgtaattaatgaagccgaagcacgcgac

actctcgacgccttacaaaacatgtttgatgttgagccggatgtcggcgcgctgttgtat

ggcgaggaaaaataaatgcgaattggacacggttttgacgtacacgcctttggcggtgaa

ggcccaattatcattggtggcgtacgcattccttacgaaaaaggattgctggcgcattct

gatggcgacgtggcgctccatgcgttgaccgatgcattgcttggcgcggcggcgctgggg

gatatcggcaaactgttcccggataccgatccggcatttaaaggtgccgatagccgcgag

ctgctacgcgaagcctggcgtcgtattcaggcgaagggttatacccttggcaacgtcgat

gtcactatcatcgctcaggcaccgaagatgttgccgcacattccacaaatgcgcgtgttt

attgccgaagatctcggctgccatatggatgatgttaacgtgaaagccactactacggaa

aaacttggatttaccggacgtggggaagggattgcctgtgaagcggtggcgctactcatt

aaggcaacaaaatgaatgggtaaactaacgctgctgttgctggctattctggtctggcta

cagtattcgctgtggttcggtaagaacggtatacatgactatacccgcgtcaatgatgat

gtggcggcacagcaagctacaaacgcgaaacttaaagcgcgaaacgatcaactttttgcc

gaaattgacgatctcaatggcggccaggaggcgctcgaagagcgtgcgcgtaatgaactc

agcatgaccaggccgggcgaaactttttatcgtctggtgcctgacgcgtcgaagcgcgca

cagtctgcggggcaaaacaatcgataaatgcgtaatagccataacattacactaacaaat

aacgacagccttactgaggatgaagaaaccacatggtcactgcctggtgccgtggtcggt

tttatctcttggttatttgcgctggcgatgccgatgttgatttatggctctaacacgctg

ttcttctttatctacacctggcctttctttcttgcgctgatgcccgtcgcggtagtggtg

gggattgcgctgcattcattgatggacggaaagctacgctacagtattgttttcaccctg

atgacagtaggcattatgtttggcgcactgtttatgtggctactgggctaaatggcgctg

catgacgaaaacgtcgtctggcatagccatccggtcactgtgcaacaacgcgagctacac

cacggtcatcgtggtgtagtgctgtggtttaccggcctctccgggtccggtaaatcaacg

gtcgccggggcgctggaggaggcgttacataaactcggcgtcagtacgtatctgctggat

ggcgacaatgttcgccacggattatgcagcgatctcggttttagcgatgccgatcgtaaa

gagaatatccgtcgcgtcggtgaagtggcgaatttgatggttgaagccggactggtggtg

ctgaccgcatttatctcgccacaccgcgccgaacgccagatggttcgtgaacgcgtagga

gaagggcgctttatcgaagtgtttgtcgatacgccgctggcgatttgcgaagcccgcgat

ccaaaaggcttatataagaaagcgcgtgccggtgaactgcgcaactttacgggaatagat

tccgtttacgaagcgcctgaatcggcagaaattcatctcaatggtgaacaattagtaaca

aatttggtacagcaattattagatctgttgagacagaacgatattatcagatcctgaatg

gaaacgactcaaaccagcacgattgcgtcgaaagactctcgtagtgcctggcgcaagaca

gacaccatgtggatgctgggcctttacggcacggcaatcggcgcgggcgtgctgttcctg

ccaatcaacgccggtgttggcggtatgatcccgctgatcatcatggctatccttgcgttc

ccgatgacgttttttgctcaccgcggcctgactcgcttcgtactgtctggtaaaaacccg

ggcgaagacatcaccgaggttgtagaagaacactttggtattggcgcaggtaaactgatt

accctgctctacttcttcgctatctacccgatcctgctggtttatagcgtggcaatcacc

aatacagttgaaagcttcatgtctcaccagctgggtatgacgccaccgccgcgtgcgatt

ctgtcgctgatcctgatcgtgggtatgatgaccatcgttcgcttcggtgagcagatgatc

gttaaagcaatgagtattctggtattcccgtttgttggcgtactgatgctgctggctctg

tacctgatcccgcagtggaacggcgcagcactggaaacgctgtctctggacactgcatct

gcaaccggaaacggtctgtggatgaccctgtggctggcaattccggtaatggtgttctcg

ttcaaccactctccgatcatctcttctttcgccgttgcgaagcgtgaagagtacggcgat

atggcagaacagaaatgctcgaagatcctggcattcgcacacatcatgatggtgctgacc

gtaatgttcttcgtcttcagctgcgtactgagcctgactccggcagacctggctgcggct

aaagagcagaacatctcgattctgtcttacctggctaaccactttaacgcaccggttatc

gcgtggatggctccgattatcgcgattatcgctatcaccaaatccttcctcggccactac

ctgggcgcacgtgaaggcttcaacggtatggtgattaaatctctgcgtggtaaaggtaag

tctatcgaaatcaacaagctgaaccgtatcactgcgctgttcatgctggtaacgacctgg

attgttgccaccctgaacccgagcatcctgggtatgattgaaaccctgggcggcccaatc

atcgcgatgatcctgttcctgatgccgatgtacgcaattcagaaagtaccggcaatgcgt

aagtacagcggtcacatcagcaacgtattcgttgtcgtgatgggtctgattgcaatctcc

gcaatcttctactctctgttcagctaaatggaacgaaataaacttgctcgtcagattatt

gacacttgcctggaaatgacccgcctgggactgaaccaggggacagcggggaacgtcagt

gtacgttatcaggatgggatgctgattacgcctacaggcattccatatgaaaaactgacg

gagtcgcatattgtctttattgatggcaacggtaaacatgaggaaggaaagctcccctca

agcgagtggcgtttccatatggcggcctatcaaagcagaccggatgccaatgcggttgtt

cacaatcatgccgttcattgcacagcagtttccattcttaaccgaccgatccccgctatt

cactacatgattgcggcggctggcggtaattctattccttgcgcgccttatgcgaccttt

ggaacacgcgaactttctgaacatgttgcgctggctctcaaaaatcgtaaggcaactttg

ttacaacatcatgggcttatcgcttgtgaggtgaatctggaaaaagcgttatggctggcg

catgaagttgaagtgctggcgcaactttacctgacgaccctggcgattacggacccggtg

ccagtgctgagcgatgaagagattgccgtagtgctggagaaattcaaaacctatgggtta

cggattgaagagtaaatgctgaaaacaatttcgccgttaatttctcccgaactattgaaa

gtgctggcagagatgggacatggagatgaaattattttttccgatgctcactttcccgcc

cattcgatgggaccgcaggtgatccgcgctgatggcctgttggtgagcgacttgctccag

gcgattatcccgttatttgagctggacagttatgcaccgccgctggtgatgatggcagcg

gtagaaggtgacactctcgatcctgaagtagaacgacgttaccgtaatgcgctttcacta

caagccccctgtcct----------------------------gacatcatccgcatcaa

tcgttttgcgttttatgaacgggcgcaaaaagcctttgcgatcgttatcacaggcgaacg

agcgaagtacgggaatattcttttaaaaaaaggggtaacaccgtaaatgtcccaacctcg

cccactgctctctcctcccgaaactgaagaacagttgttagcgcaagcacagcaactttc

tggttatacattgggagaactggcggcacttgccgggctggttacgccagagaatttaaa

acgcgataagggctggattggcgtgttactggagatctggctaggtgccagcgcagggag

taaacctgagcaagattttgctgctctgggcgtggaacttaaaactatccctgtggatag

tcttggtcgtccgctggaaacaacattcgtttgtgttgccccgttaacgggcaatagcgg

agtgacctgggaaaccagccacgtgcgccacaaactcaaacgcgtactgtggataccggt

tgaaggcgagcgcagcatcccgctggcgcagcgtcgcgttggatcaccgttgctgtggag

cccgaatgaagaggaagaccggcagctacgcgaagactgggaagaattaatggatatgat

tgttctcggtcaggttgagcggattaccgctcgtcacggggagtatttacagatacgacc

gaaagcagcgaatgcgaaagcgcttaccgaagccattggtgcccggggcgaacggattct

gacgctgccgcgcggcttttatttgaagaaaaatttcaccagtgcgctgctggcccgtca

ttttctgatccagtagatgttatttgcatggataaccgatcctaacgcctggcttgcgct

cggtacgctgacgctgctggagatcgttcttgggatcgacaatattattttcctttctct

ggtggtggcaaagctccccacagcacaacgtgctcatgcgcgccgtctggggttggcggg

agccatggttatgcgtctggcgctgctggcatcaatcgcctgggttacgcgcctgacgaa

tccgctttttacaatattcagtcaggaaatttccgcccgtgatttgattctgcttctggg

tggcttgttccttatctggaaagccagcaaggaaatccacgaatccattgaaggtgaaga

agaagggctgaaaacacgcgtttcatcattcctcggcgctatcgtgcagattatgctgct

ggatattatcttcagcctcgactcggtgattaccgctgtgggtctgtcagatcacctgtt

tattatgatggccgccgtggtaattgccgtaggcgtgatgatgttcgccgcgcgctcgat

tggtgattttgtcgagcgccatccttcggtaaaaatgctggcgctctctttcctgattct

ggtgggctttaccctgattctggaaagtttcgacatccacgtaccgaaaggttacatcta

cttcgcgatgttcttctctattgcggttgaaagcctcaacctgattcgcaacaaaaagaa

tccgctctgaatgaatatgggtcttttttacggttccagcacctgttacaccgaaatggc

ggcagaaaaaatccgcgatattatcggcccagaactggtgaccttacataacctcaagga

cgactccccgaaattaatggagcagtacgatgtgctcattctgggtatcccgacctggga

ttttggtgaaatccaggaagactgggaagccgtctgggatcagctcgacgacctgaacct

cgaaggtaaaattgttgcgctgtatgggcttggcgatcaactgggatacggcgagtggtt

cctcgatgcgctcggtatgctgcatgacaaactctcgaccaaaggcgtaaagttcgtcgg

ctactggccaacggaaggatatgaatttaccagcccgaaaccggtgattgctgacgggca

actgttcgtgggtctggcgctggatgaaactaaccagtatgaccttagcgacgagcgtat

tcagagctggtgcgagcaaatcctcaacgaaatggcagagcattacgcctgaatgaacga

tattgcgcataacctggcacaggtccgggacaaaatctcagcggctgcaacgcgttgcgg

ccgttctccagaagaaattacgctgcttgcagtcagtaaaacaaaacctgcgagcgccat

cgcagaagccattgatgccgggcagcgtcaatttggtgaaaactacgttcaggaaggggt

agataaaattcgccactttcaggaactgggcgtaacaggattagaatggcattttattgg

cccgttgcagtctaataaaagccgcctggtggcagagcatttcgactggtgtcataccat

cgaccgtttgcgcatcgctacccgcctcaacgatcagcgcccggcagaacttccccctct

taacgtgctgattcaaattaatattagtgatgaaaacagtaagtccgggattcaactggc

agaactggacgagctggcagctgcggtcgctgaactaccgcgtttacgtctgcgcgggct

gatggcaatccctgcgcctgagtcagaatatgtaaggcagtttgaagttgcacgccaaat

ggctgtagcatttgccggactgaaaacgcgctacccgcatatcgacacgctctctctggg

aatgtcggacgatatggaagccgccattgcggcaggtagcacgatggttcgtatcggcac

tgcaatttttggtgcgcgtgattactctaaaaaataaatgaatacgttgactttcctgct

ttcaacggtcattgagctgtataccatggtgctgttattacgcatctggatgcagtgggc

tcattgtgatttttacaaccccttctcacagtttgtagtgaaggtaacacagccaattat

cgggccactgcgccgcgttattccggcaatgggacccattgacagcgcctcgctgctggt

tgcctatattctcagttttatcaaagccattgtgctgtttaaagtggtgaccttcctgcc

aatcatctggattgccggtttactgattctgctgaaaaccatcggcctgctgattttctg

ggtcctgctggtgatggcgattatgagctgggtaagccagggtcgtagcccgattgaata

cgtgctgattcagctggccgatccgctgctgcgcccgattcgccgcctgctaccagcaat

gggtgggattgatttctcgccgatgatcctcgttctgctgctgtatgtcatcaatatggg

tgtcgcagaagtattacaggcaaccggaaacatgctgctgccggggctgtggatggcgtt

atgaatgcaaaaagttgtcctcgcaaccggcaatgccggtaaagtgcgtgagctggcgtc

gctgcttagcgacttcggtcttgatatcgtggcccaaacagacctcggcgtagattccgc

tgaagaaaccggcctgacctttatcgaaaacgcgattctgaaagcgcgccatgcggcaaa

agtgaccggtttaccggcaattgccgacgactccggtctggcggtagatgtacttggcgg

tgcgccggggatttactccgcgcgttattccggtgaagacgcgaccgatcaaaagaatct

gcaaaaactgctggaaacactgaaagacgtaccggacgaccaacgtcaggcgcgtttcca

ctgcgtgctggtatatctgcgtcacgcggaagatccgactccgctggtgtgccacggtag

ttggccgggcatgattactcgtgaaccagcgggtactggcggctttggttatgatccaat

cttcttcgtaccttccgaagggaaaaccgctgccgaactgacccgcgaagaaaagagcgc

catttcccaccgtggtcaggcattgaaactgctgctggacgctttacgtaatggttaaat

gaaaaacgacgtcatttcaccggaatttgatgaaaacggccgcccactgcgccgtatccg

tagttttgtgcgccgccaggggcgactgaccaaaggccaggaacatgcgctggaaaacta

ctggccggtgatgggcgttgagttcagcgaagatatgctggatttccccgcgctttttgg

ccgtgaagcgccggtgacgcttgagattggttttggcatgggggcgtcgctggtggcaat

ggctaaagatcgccccgagcaggacttcttcggcattgaagtgcattcaccgggcgttgg

tgcgtgcctggcttctgcgcatgaagagggcttaagcaacctgcgcgtgatgtgtcacga

tgcggttgaagtgctgcataaaatgattcctgacaattcattgcgcatggtgcagctctt

tttccctgacccgtggcacaaagcgcgccataataaacgccgtatcgttcaggtgccgtt

tgccgaactggtaaaaagcaaactgcagctggggggcgtattccatatggcgaccgactg

ggaaccttatgcggaacatatgcttgaagtgatgtcttctattgacggttataaaaacct

gtcagagagcaatgattacgtaccgcgtccggcatcacgtccggtgacgaaatttgaaca

acgtggtcatcgtcttggtcacggagtatgggacttaatgttcgagagggtgaaataagt

gaacagacgtaattttattaaagcagcctcctgcggggcattgctgacgggcgcgttgcc

gtctgtcagtcatgcggctgctgaaaaccgcccgccaattccgggatcgctggggatgtt

gtacgactcgaccttgtgcgtaggctgccaggcttgcgtcaccaagtgtcaggatatcaa

cttccctgaacgtaacccgcgaggggaacagacctggtcgaacaacgacaaactatcgcc

gtataccaataacatcattcaggtgtggaccagcggcactggcgtcaacaaagaccagga

ggagaacggctacgcgtacattaagaaacagtgtatgcactgcgtcgatccgaactgtgt

ctctgtgtgcccggtttctgcactgaaaaaagatccgaaaaccggcattgtccattacga

caaagacgtgtgcaccggctgccgttactgcatggtcgcctgtccgtacaacgtgccgaa

gtacgactacaacaacccgtttggtgcgctgcataagtgcgagctgtgcaaccagaaagg

tgtggaacgtctcgataaaggcggtctgcctggctgcgtagaagtgtgcccggcgggcgc
[truncated: 7,805,128 more chars]
