# Supplementary figures and images for: Comparative genomic analysis of a Shiga toxin-producing Escherichia coli (STEC) O145:H25 associated with a severe pediatric case of hemolytic uremic syndrome in Davidson County, Tennessee, US
Source: BMC Genomics. 2020 Aug 17;21:564. doi: 10.1186/s12864-020-06967-3 (PMC7437938; doi:10.1186/s12864-020-06967-3)

## Slide 1
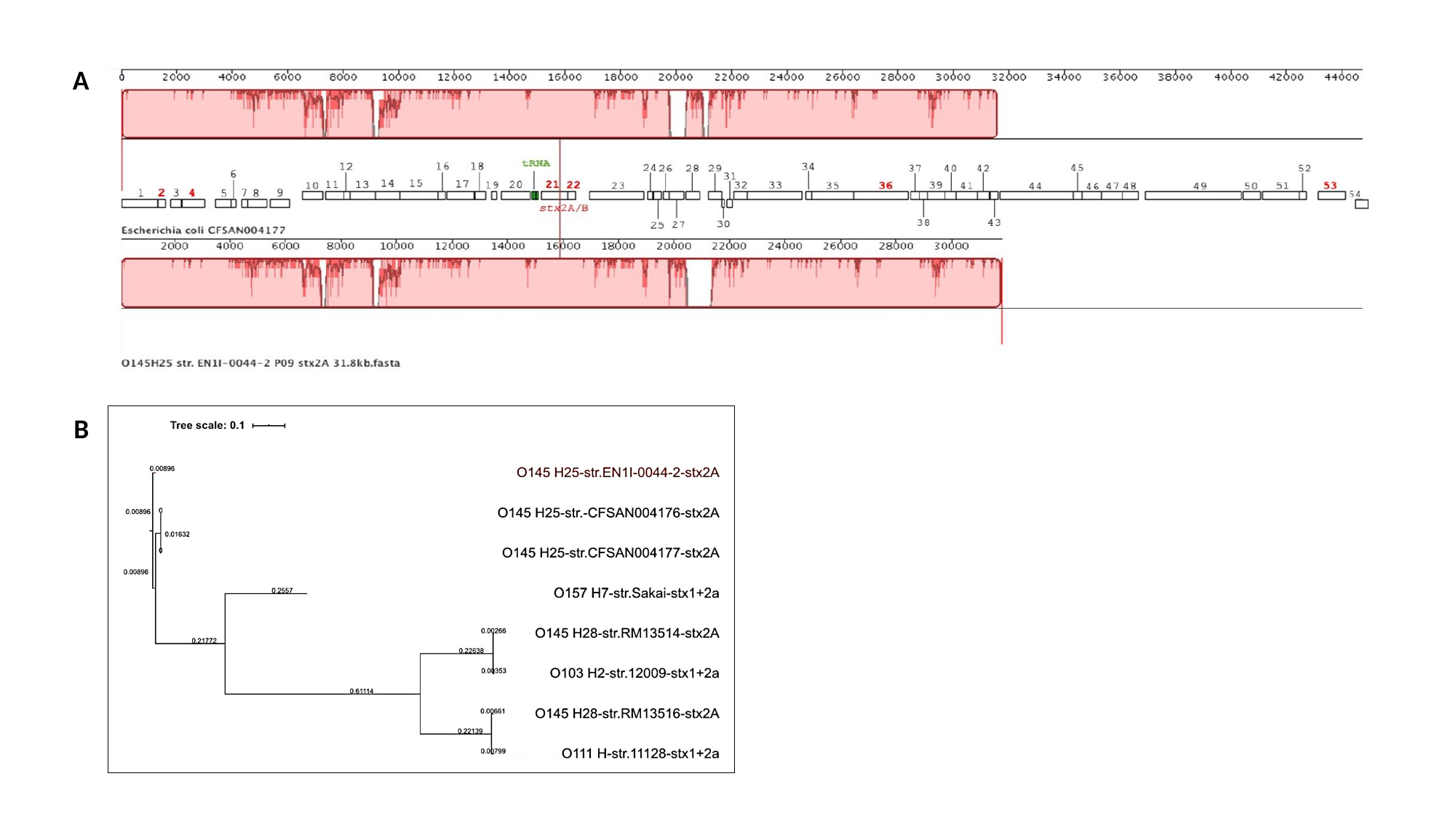

A
B

Supplement: Supplementary file 6 — Additional file 6: Figure S1. Stx2a prophages gene alignment and evolutionary relationships. Panel A, Diagram of gene alignment of Stx2a phage from STEC O145:H25 CFASN004177 and phage P09 from study strain STEC O145:H25 EN1I-0044-2. Annotated sequence of Stx2a prophage from strain CFSAN004177 was taken from GenBank: [CP014670.1], position start: 4,500,642 and position end: 4,545,386. Prophage alignment was performed using Mauve (Darling AE, Mau B, Perna NT., 2010). Numbers indicate ORFs. Numbers in read correspond to virulence genes, among them the stx2A and B genes. Panel B, Phylogenetic tree of P09 phage sequence compared with stx2a prophages from O145:H25, O145:H28, O157, and additional non-O157 serogroups. The maximum likelihood (ML)-based phylogenetic tree based on phage sequence comparisons. [file 12864_2020_6967_MOESM6_ESM.pptx]
